# Discovery of genes involved in anthocyanin biosynthesis from the rind and pith of three sugarcane varieties using integrated metabolic profiling and RNA-seq analysis

**DOI:** 10.1186/s12870-021-02986-8

**Published:** 2021-05-12

**Authors:** Yang Ni, Haimei Chen, Di Liu, Lihui Zeng, Pinghua Chen, Chang Liu

**Affiliations:** 1 Key Laboratory of Ministry of Education for Genetics, Breeding and Multiple Utilization of Crops, National Engineering Research Center of Sugarcane, College of Agriculture, Fujian Agriculture and Forestry University, 350002 Fuzhou, Fuzhou, Fujian Province P. R. China; 2 Institute of Medicinal Plant Development, Chinese Academy of Medical Sciences, Peking Union Medical College, 100193 Beijing, P. R. China

**Keywords:** Anthocyanins, Sugarcane, Transcriptome, Metabolome, DEGs

## Abstract

**Background:**

Sugarcane (*Saccharum officinarum*) is one of the most valuable feedstocks for sugar production. In addition to the production of industrial raw materials such as alcohol, papermaking, the fiber of livestock feed, respectively, sugarcane can produce bioactive compounds such as anthocyanins. Elucidation of the anthocyanin biosynthesis pathway is critical for the molecular breeding of sugarcane varieties with favorable traits. We aimed to identify candidate genes involved in anthocyanin biosynthesis by transcriptomic and metabolomic analyses.

**Results:**

Three varieties of sugarcane displaying different colors were used in this study: FN15 (greed rind), ROC22 (red rind), and Badila (purple rind). Sample materials were subjected to metabolomic analysis using UPLC-Q-TOF/MS and RNA-seq analysis. The metabolomic profiling results showed Cyanidin, Cyanidin (6’-malonylglucoside), Cyanidin O-glucoside, and Peonidin O-glucoside were the main components responsible for the rind color. Then, through RNA-seq analysis, we identified a total of 3137, 3302, 3014 differentially expressed genes (DEGs) between the rind and pith tissues for the corresponding varieties Badila rind, ROC22, and FN15. We then compared the expression levels of genes among the rind tissues from the three varieties. We identified 2901, 2821, and 3071 DEGs between Badila rind vs. ROC22 rind, Badila rind vs. FN15 rind, ROC22 rind vs. FN15 rind, respectively. We identified two enriched pathways, including phenylpropanoid biosynthesis and flavonoid biosynthesis. Sequencing similarity search identified a total of 50 unigenes belonging to 15 enzyme families as putative genes involved in anthocyanin biosynthesis in sugarcane rind. Seven of them were identified as candidate genes related to anthocyanin biosynthesis in the rind of sugarcane through co-localization analysis with the anthocyanin content in sugarcane. In total, 25 unigenes were selected and subjected to RT-qPCR analysis, and qRT-PCR results were consistent with those obtained with the RNA-Seq experiments.

**Conclusions:**

We proposed a pathway for anthocyanin biosynthesis in sugarcane rind. This is the first report on the biosynthesis of anthocyanin in sugarcane using the combined transcriptomic and metabolomic methods. The results obtained from this study will lay the foundation for breeding purple pith sugarcane varieties with high anthocyanin contents.

**Supplementary Information:**

The online version contains supplementary material available at 10.1186/s12870-021-02986-8.

## Background

Sugarcane (*Saccharum officinarum*) is one of the most valuable feedstocks for sugar production [1]. Sugar extracted from sugarcane represents 70 % of global sugar production. The processed by-products of sugarcane can be used as industrial raw materials such as alcohol, papermaking, fiber, and livestock feed, respectively [2]. Sugarcane is a proven biofuel feedstock and accounts for about 40 % of the biofuel production worldwide [3]. Besides, sugarcane can provide large numbers of bioactive compounds for human health. The anthocyanins extract from sugarcane peel (*Saccharum Officinarum*) shows that 51.2 % inhibition of the HT29 cell line at a concentration of 0.625 µg/ml reduces the risk of colon cancer [4]. Duarte-Almeida et al. found that the predominant phenolics in sugarcane culms were phenylpropanoids related to antioxidant activity [5]. However, the bioactive compounds in sugarcane, such as phenolic compounds, have not been utilized and developed adequately.

The flavonoids are an important kind of phenolic compounds containing anthocyanins, flavones, and proanthocyanidins widely existing in the plants’ leaves and fruits. Anthocyanins are naturally occurring polyphenols responsible for the colors in most flowers and fruits of plants. Dietary consumption of anthocyanins has been shown to reduce the risk of cardio- and cerebrovascular diseases, atherosclerosis, cancer, diabetes, and failing vision. Such a beneficial effect could be related to the potent antioxidant activity of anthocyanin compounds [6]. Cyanidin, peonidin, malvidin, pelargonidin, petunidin, and delphinidin are six common natural anthocyanins. Under normal circumstances, anthocyanins are mainly accumulated in plant organs, which give plants colorful colors and contribute to their ornamental and economic values [7].

Also, anthocyanins play an indispensable role in protecting the growth and development of plants. When plants are exposed to cold stress, CFBs transcription factors are activated, which affects the expression of anthocyanin synthetic genes, resulting in the increase of the anthocyanin contents and cold resistance in plants [8]. The anthocyanin content in sugarcane leaves increased significantly under cold stress, thereby compensating for the lack of antioxidants in a low-temperature environment [9]. Flavonoids’ production in plants changes in response to light intensity [10]. When plants are exposed to intense sunlight, anthocyanins are produced in large quantities to protect plant chloroplasts from oxidation [11].

Furthermore, anthocyanins have specific effects on the body’s antioxidant and anticancer aspects [12]. For example, anthocyanins can lower blood lipids and cholesterol [13]. Simultaneously, anthocyanins also have specific functions in treating glaucoma and protecting vision [14]. For the bioactive effects of anthocyanins described, increasing its contents in various plants has been one of the most popular research topics.

Sugarcane is an excellent breeding material and is grown on a large scale in China. Anthocyanins have high economic value, and anthocyanin-rich sugarcane can be obtained quickly by cultivating purple-hearted sugarcane. The naturally occurring anthocyanins and flavonoids have been found in *Saccharum* species such as *S*. *officinarum*, *S. robustum*, *S barberi*, and their inter-varietal, inter-generic, and interspecific crosses. For example, Mabry et al. used spectroscopic and chemical evidence to build two structures of flavonoids in *S*. *officinarum* [15]. Li et al. systematically isolated flavonoids and anthocyanins from Chinese sugarcane (*S. sinensis Roxb*) [16]. Li et al. determined the flavonoid content of different tissue parts of *S. sinensis Roxb* [17]. Zhao et al. find that the content and variation of anthocyanins in different cultivars of sugarcane were significant, and 13 anthocyanins and their glycosyl derivatives were identified [18].

The accumulation of 3-deoxyanthocyanidin in sugarcane has been identified to strengthen the resistance to the red rot pathogen *Colletotrichum falcatum* [19]. Ganesh et al. used HPLC analysis to reveal the mechanism of nine 3-deoxyanthocyanidin compounds against *Colletotrichum falcatum* resistance differentially from different sugarcane cultivars [20]. Ganesh et al. revealed the mechanism of differential expression of key genes in the anthocyanin metabolic pathway by studying the antifungal properties of 3-deoxyanthocyanidin [21]. In addition, the researchers have shown that 3-Deoxyanthocyanidin flavonoids increased the resistance to the attack of maize aphids in *Sorghum bicolor* [22]. In summary, increasing the anthocyanidin contents might increase the resistance to pathogen infection and insect attack.

Several naturally red-fleshed *Saccharum* clones have been described [23]. Chandran et al. reported nine germplasm clones resource of red-fleshed of Saccharum *robustum* (28 NG 219, NG 77 − 73, NG 77 − 75, NG 77 − 76, NG 77–78, NG 77–84, NG 77–88, NG 77–90 and NG 77–132). Their breeding value and applications of this sugarcane in terms of yield and morphological traits were discussed in detail [1]. These red-fleshed canes are of great breeding value. The presence of red-fleshed *S. robustum* clones led us to hypothesize that breeding for red-flashed *S*. *officinarum* is also possible.

The whole genome of wild-type sugarcane (*S. spontaneum L.*) has been sequenced and assembled by Jisen Zhang et al. [12]. However, very few studies have been reported on the biosynthesis of anthocyanins in sugarcane to date. This study aims to reveal anthocyanin-biosynthesis genes in sugarcanes by comparing gene expression levels sugarcane varieties with different rind colors. The results obtained from this study will provide a theoretical basis for the cultivation of purple-hearted sugarcane and provide a basis for subsequent gene cloning, gene function validation, molecular marker screening, and genetic improvement of sugarcane varieties.

## Materials and methods

### Plant materials

**Fig. 1:**
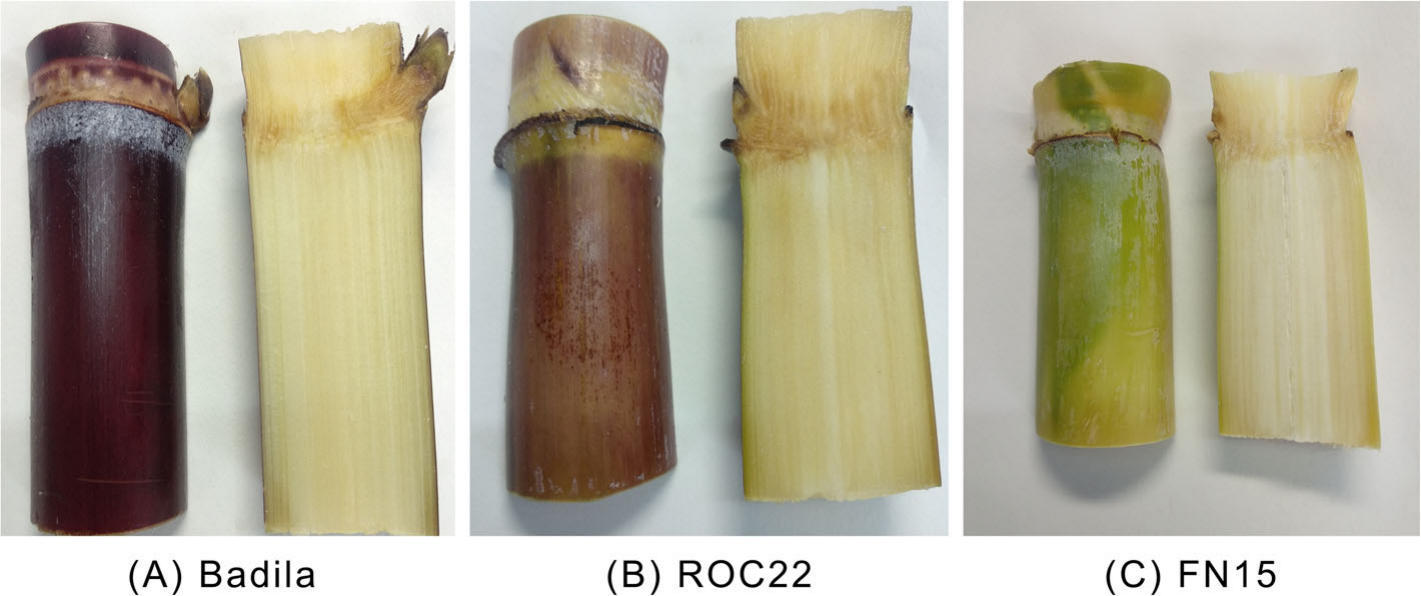
Three sugarcane varieties of different colors. Badila (**a**), ROC22 (**b**), and FN15 (**c**) are shown. The rind and pith are shown on the left and right, respectively

We used three sugarcane (*S. officinarum*) varieties: FN15, ROC22, and Badila with a green rind, red rind, and purple rind, respectively (Fig. 1). We collected the three varieties collected from Sugarcane Germplasm Resource Nursery of the National Sugarcane Engineering Research Center of Fujian Agricultural and Forestry University (26.0886 N, 119.2435 E), China. We cleaned the surface of the sugarcane rind repeatedly with DEPC water. The rind and inner pith of sugarcane samples were separated with a sharp knife, cut into small pieces, packed with tin foil paper, frozen in liquid nitrogen, stored at − 80 °C until use.

### Profiling of anthocyanins using UPLC-Q-TOF/MS

We prepared anthocyanins as described previously [17]. The process including the following steps: we weighed 200 mg sample, then added 1ml 1 % methanolic acetate solution, mixed them by shaking, let the solution stand at low temperature and dark overnight, collected the extract, extracted three times with 1ml 1 % methanolic acetate solution, transferred to a flask, made up to a final volume of 50 ml with a 1 % methanolic acetate solution. In summary, there are three varieties. Samples were taken from the rind and pith tissues of three individual plants of each variety. We then pooled the samples from the biological replicates for further analyses. There are three biological replicates for each sample. The standard compounds were cyanidin, Malvidin, Pelargonidin, Peonidin.

We performed LC-HRMS analyses on a Waters UPLC I-class system equipped with a binary solvent delivery manager and a sample manager, coupled with a Waters VION IMS Q-TOF Mass Spectrometer equipped with an electrospray interface (Waters Corporation, Milford, USA). We used the column Acquity BEH C18 column (100 mm × 2.1 mm i.d., 1.7 μm; Waters, Milford, USA). The separation was achieved using the following gradient: 5–20 % B over 0–2 min, 20–60 % B over 2–8 min, 60–100 % B over 8–12 min. The composition was held at 100 % B for 2 min, then at 100 to 5 % B for 14–14.5 min, and at 5 % B for 14.5–15.5 min at a flow rate of 0.40 mL/min. Here A is aqueous formic acid (0.1 % (v/v) formic acid) and B is acetonitrile (0.1 % (v/v) formic acid). The injection volume was 3.00 µL, and the column temperature was set at 45.0 °C. ESI ion source was used to ensure the data collected in a negative ion mode.

### RNA isolation and sequencing

We extracted total RNAs using the mirVana miRNA Isolation Kit (Cat. AM1561, Invitrogen, Thermo Fisher Scientific Inc., USA) following the manufacturer’s protocol. The RNA purity was assessed and quantified using a NanoDrop 2000 spectrophotometer (Thermo Fisher Scientific, Waltham, MA, USA). Then, RNA integrity was appraised using the Agilent 2100 Bioanalyzer (Agilent Technologies, Santa Clara, CA, USA). The transcriptome sequencing and analysis were conducted by OE Biotech Co., Ltd. (Shanghai, China). Briefly, the libraries were sequenced on an Illumina HiSeq X Ten platform, and 150 bp paired-end reads were generated. Raw data of FASTQ format were corrected with Trimmomatic to remove the adaptor sequences and filter out the low-quality reads [24]. The ploy-N and low-quality reads were filtered out of the raw data with the parameters “LEADING = 3”, “TRAILING = 3”, and “MINLEN = 50”.

### De novo assembly and function annotation

The clean reads were *de novo* assembled into transcripts by Trinity software (version: 2.4) according to the paired-end splicing method [25]. We selected the longest transcript of each unigene for subsequent analysis. The unigenes were compared with known sequences in the NR (ftp://ftp.ncbi.nlm.nih.gov/blast/db/FASTA/nr.gz ); SWISS-PROT (http://www.uniprot.org/ ), and KOG (ftp://ftp.ncbi.nih.gov/pub/COG/KOG/kyva) databases using BLAST with an E-value cutoff of 1e-5. The transcripts were categorized by mapping their sequences against those in the Kyoto Encyclopedia of Genes and Genomes (KEGG: http://www.genome.jp/kegg/) database [21]. The KO numbers and KEGG reference metabolic pathways were inferred based on those of their best hit sequences. Similarly, the unigenes were mapped to proteins from SwissProt. Their Gene Ontology (GO, http://www.geneontology.org/) classifications were inferred from those of their best hits.

### Gene expression quantification and differential gene expression analysis

The FPKM [26] (fragments Per kb per Million reads) of each unigene was calculated using software bowtie2 [27] and eXpress [28]. Differentially expressed genes (DEGs) were identified using the DESeq [29] with a model based on the negative binomial distribution. The results of all statistical tests were corrected by multiple tests using the Benjamini and Hochberg false discovery rate. Genes were determined to be significantly differentially expressed with |Log_2_FoldChange| ≥1 and the adjusted P-value of < 0.05 according to the default settings in DESEq. We conducted a hierarchical cluster analysis of DEGs with TBtools [30].

### Enrichment analysis

We performed Gene Ontology (GO) and KEGG enrichment analysis on differentially expressed genes to describe their functions. GO classification was performed by mapping our proteins to those in Swissprot using BLASTN. The related GO terms were then extracted from the annotations for the hit proteins in SwissProt. KEGG enrichment analysis was conducted using the KOBAS database (http://kobas.cbi.pku.edu.cn/kobas3). After that, we counted the number of differential genes included in each GO entry and KEGG pathway. Then we calculated the significance of the enrichment of differential genes in each GO entry and KEGG using the hypergeometric distribution test method. The resulting p-value was subjected to correction to calculate the False Discovery Rate (FDR) or adjusted p-value. An adjusted p-value < 0.05 was used as the cutoff for significant enrichment.

### Validation of RNA-seq experiments

We conducted a Reverse transcription-quantitative real-time PCR (RT-qPCR) analysis with the RNA samples used for the RNA-seq experiments. Each experiment had three technical replicates. In total, 1 µl total RNA was processed with the PrimeScript™ RT reagent Kit with gDNA Eraser. The reactions included two steps. Firstly, genomic DNA removal reaction included 1 µL RNA, 1 µL gDNA Eraser, 2 µL 5×gDNA Eraser Buffer, 16 µL RNase Free water. Reverse transcription reaction included 1 µL PrimeScript RT Enzyme Mix, 1 µL RT Primer Mix, 4 µL 5×PrimeScript Buffer 2, 4 µL RNase Free water. The Gene-specific primers were designed by the IDT (https://sg.idtdna.com/Primerquest/Home/Index) [31]. All the primers are shown in Table S1. We chose the NADPH gene as the endogenous control. Reverse transcription-quantitative real-time PCR reaction contained 10 µL 2 × Master Mix, 2 µL cDNA, 0.5 µL 10 μm PCR gene-specific forward primers, 0.5 µL 10 μm PCR gene-specific reverse primers, 7 µL RNase Free water. The cycling conditions were 95 ºC for 30 s with 40 cycles. To establish the melting curve of PCR products, after the amplification reaction is over, press (95 ºC, 10 s; 60 ºC, 60 s; 95 ºC, 15 s); and slowly heat from 60 ºC to 99 ºC. The target gene and internal reference of each sample were subjected to real-time PCR reaction, and three replicate wells were tested for each sample, and the data were analyzed by the 2^−△△Ct^ method [32].

### 2.8 Analysis of structural genes related to the biosynthesis of anthocyanins in the rind of sugarcane

The structure genes associated with the biosynthesis of anthocyanins in the rind of sugarcane were identified as described below. First, we downloaded the gene sequences from the KEGG pathway with id ko00941 (flavonoid biosynthesis) and ko00942 (anthocyanin biosynthesis) to construct a local reference database. Then, the unigene sequences obtained from the RNA-seq experiments from this study were searched against the local reference database using the BLASTX algorithm (v2.7.1+) [33], with an e-value cutoff of 1e-5. Thirdly, the selected unigenes were subjected to the open reading frame (ORF) identification using the TransDecoder program (v5.5.0) with default parameters. Lastly, the ORFs were used to search the CD-search database at https://www.ncbi.nlm.nih.gov/Structure/bwrpsb/bwrpsb.cgi with an e-value cutoff of 1e-5. We identified the full-length protein sequences based on the result of the CD-search. The Pearson correlation coefficients between the expression profiles of genes related to anthocyanin-biosynthesis and the content of the derivative of cyanidin in sugarcane were calculated using TBtools software (Version 1.05).

We performed multiple sequence alignment of full-length sequences using the MUSCLE [34]. We visualized the multiple sequence alignment results using GeneDoc software [35]. Phylogenetic analysis of full-length genes was conducted with the Maximum Likelihood Estimate method implemented in MEGA7 software [36]. We calculated the bootstrap score based on 1000 replications. We ran the weighted correlation network analysis (WGCNA) to determine the relationships between phenotypes and differential genes with the R package of WGCNA [37].

## Result

### Anthocyanins involved in sugarcane

**Fig. 2:**
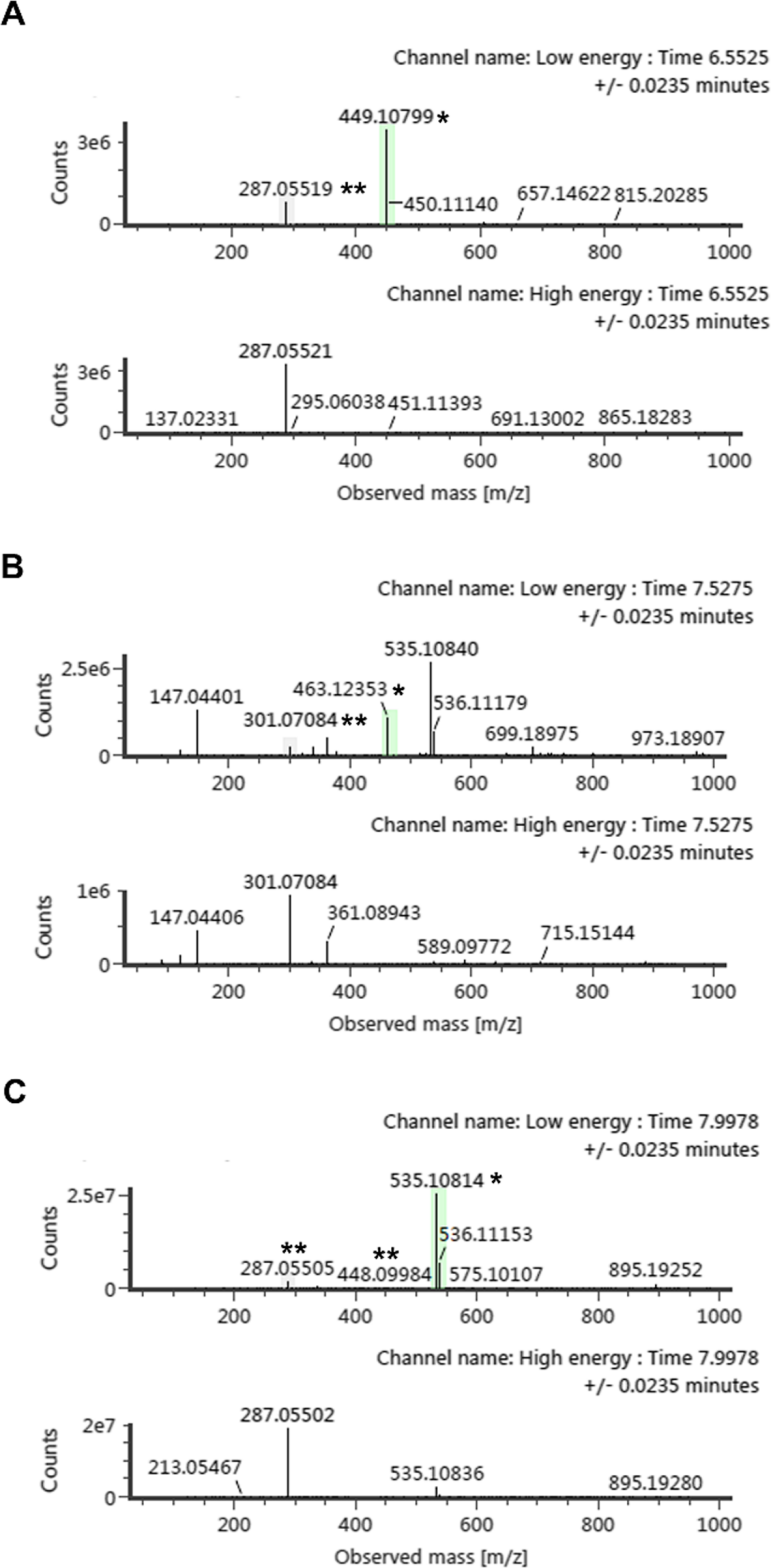
Spectra of product ions for anthocyanins in sugarcane samples by UPLC-Q-TOF/MS under low and high energy. The precursor of cyanidin O-glucoside (**a**), peonidin O-glucoside (**b**), cyanidin_C3C6 (**c**) were analyzed. The corresponding peaks of precursor and product ions were marked with a star

To compare the flavonoids and anthocyanin compounds among the three sugarcane varieties, we obtained the rind and pith sugarcane varieties’ total ion chromatograms from ultra-high-performance liquid chromatography-quadrupole time-of-flight mass spectrometry (UPLC-Q-TOF/MS). We identified the chemical components of sugarcane through retention time, exact relative molecular mass, cleavage fragments of MS/MS, and previously reported data. Figure 2 shows the spectra of cyanidin O-glucoside (A), peonidin O-glucoside (B), cyanidin (6’-malonylglucoside) (C) in sugarcane. Cyanidin O-glucoside showed a formula of C21H21O11 and retention time of 6.65 with m/z 449, which produced one fragment located at m/z 287. Transition 449 > 287 represented the loss of glucose (m/z 162) (Fig. 2 a). Peonidin O-glucoside showed a formula of C22H23O11 and retention time of 7.53 with m/z 463. The m/z 301 fragments were formed, which corresponds to peonidin C16H13O6 (m/z 301.07051) (Fig. 2b). Cyanidin (6’-malonylglucoside) showed a formula of C24H23O14 and a retention time of 7.99 with m/z 535.10814, which produced one fragment m/z 448 and 287 (Fig. 2 c). Transition 535 > 448 represented the loss of malonyl (m/z 87), and transition 535 > 287 produced cyanidin (m/z 287) due to the loss of both glucose and malonyl.

As shown in Table 1, a total of 7 anthocyanins were identified and quantified in the rind and pith of sugarcane. Most anthocyanins in the rind and pith of sugarcane are cyanidin, which included cyanidin, cyanidin (6’-malonylglucoside), and cyanidin O-glucoside. The total anthocyanidin contents in rind samples were higher than pith samples. The content of cyanidin, cyanidin (6’-malonylglucoside), cyanidin O-glucoside and peonidin O-glucoside peonidin_C6 in rind is higher than the content in pith. In the rind of Badila with purple color, the contents of peonidin, cyanidin (6’-malonylglucoside), cyanidin O-glucoside, peonidin O-glucoside are significantly higher than the contents in the rind of ROC22 and FN15. As a result, the derivative of cyanidin is the most abundant ingredient in the rind of sugarcane, which is the main factor affecting the rind color of sugarcane.

**Table 1:** Chemical components of anthocyanins and their concentrations in the rind and pith tissues of the three sugarcane varieties

Chemical component name	Formula	Retention time (min)	Abundance(µg/g)					
			Badila		ROC22		FN15	
			Rind	Pith	Rind	Pith	Rind	Pith
Cyanidin	C15H11O6	8.53	30.77 ± 0.01	ND	15.03 ± 2.40	ND	5.16 ± 0.32	0.66 ± 0.01
Malvidin	C17H15O7	10.17	6.30 ± 0.35	3.61 ± 0.11	4.61 ± 0.88	8.81 ± 1.06	12.85 ± 1.11	32.68 ± 1.42
Pelargonidin	C15H11O5	9.47	0.27 ± 0.03	ND	0.92 ± 0.26	ND	0.48 ± 0.05	0.08 ± 0.01
Peonidin	C16H13O6	9.92	2.13 ± 0.01	ND	1.25 ± 0.30	0.16 ± 0.05	0.79 ± 0.17	0.23 ± 0.04
Cyanidin (6’-malonylglucoside)	C24H23O14	7.99	10891.78 ± 815.03	1.26 + 0.47	1193.76 ± 371.68	ND	125.81 ± 36.94	ND
Cyanidin O-glucoside	C21H21O11	6.65	6819.82 ± 924.32	ND	335.26 ± 140.44	ND	30.80 ± 3.60	ND
Peonidin O-glucoside	C22H23O11	7.53	82.18 ± 14.14	ND	13.75 ± 4.11	ND	0.94 ± 0.07	ND

*ND* not detected

### RNA-seq analysis of the rind and pith tissues of three sugarcane varieties

We constructed six cDNA libraries from three sugarcane varieties with different rind colors to explore the molecular mechanism of anthocyanins biosynthesis and accumulation in the rind and pith tissues of sugarcane. The six samples were named Badila_rind (purple), Badila_pith; ROC22_rind (red), ROC22_pith; FN15_rind (green), FN15_pith. After removing the adapters, low-quality sequences, and reads shorter than 35 bp, we obtained 55.5, 57.1, 55.2, 59.9, 5 6.1, and 57.2 million clean reads for the six samples. These clean data were assembled using the Trinity, and we obtained 73,916 unigenes longer than 300 bp. The average length of those unigenes is 646 bp, and the length of N50 is 1398 bp. The length of distribution for the unigenes is shown in Fig. S1.

To understand these genes’ putative functions, we compared a total of 73,916 unigenes to five public databases using BLASTN and BLASTX. The databases included the following: NR, GO, SWISSPROT, KEGG, and KOG. Finally, 43,546, 21,210, 8,297, 27,966 and 23,821 unigenes were annotated by NR, KOG, KOG, KEGG, SWISSPROT and GO databases. There were 6,114 unigenes annotated by all the databases and 43,827 unigenes annotated only by one database (Fig. S2).

The 23,821 unigenes mapped with GO terms were divided into 64 function groups belonging to the three main GO classifications: biological process, cellular component, and molecular function (Fig. S3). In contrast, 16,133 unique sequences were assigned to KEGG pathways (Fig. S4).The top 10 most mapped pathways were “Transport and Catabolism”(1,013 sequences), “Cell growth and Death”(1,022 sequences), “Signal Transduction”(3,005 sequences), “Translation”(1,027 sequences), “Carbohydrate metabolism”(1,748 sequences), “Amino acid metabolism”(1,057 sequences), “Lipid metabolism”(994 sequences), “Folding, Sorting and Degradation”(944 sequences), “Replication and Repair”(776 sequences), “Energy Metabolism”(714 sequences) (Fig. S3).

### Real‐time PCR validation of the expression levels of anthocyanin‐related genes

**Fig. 3:**
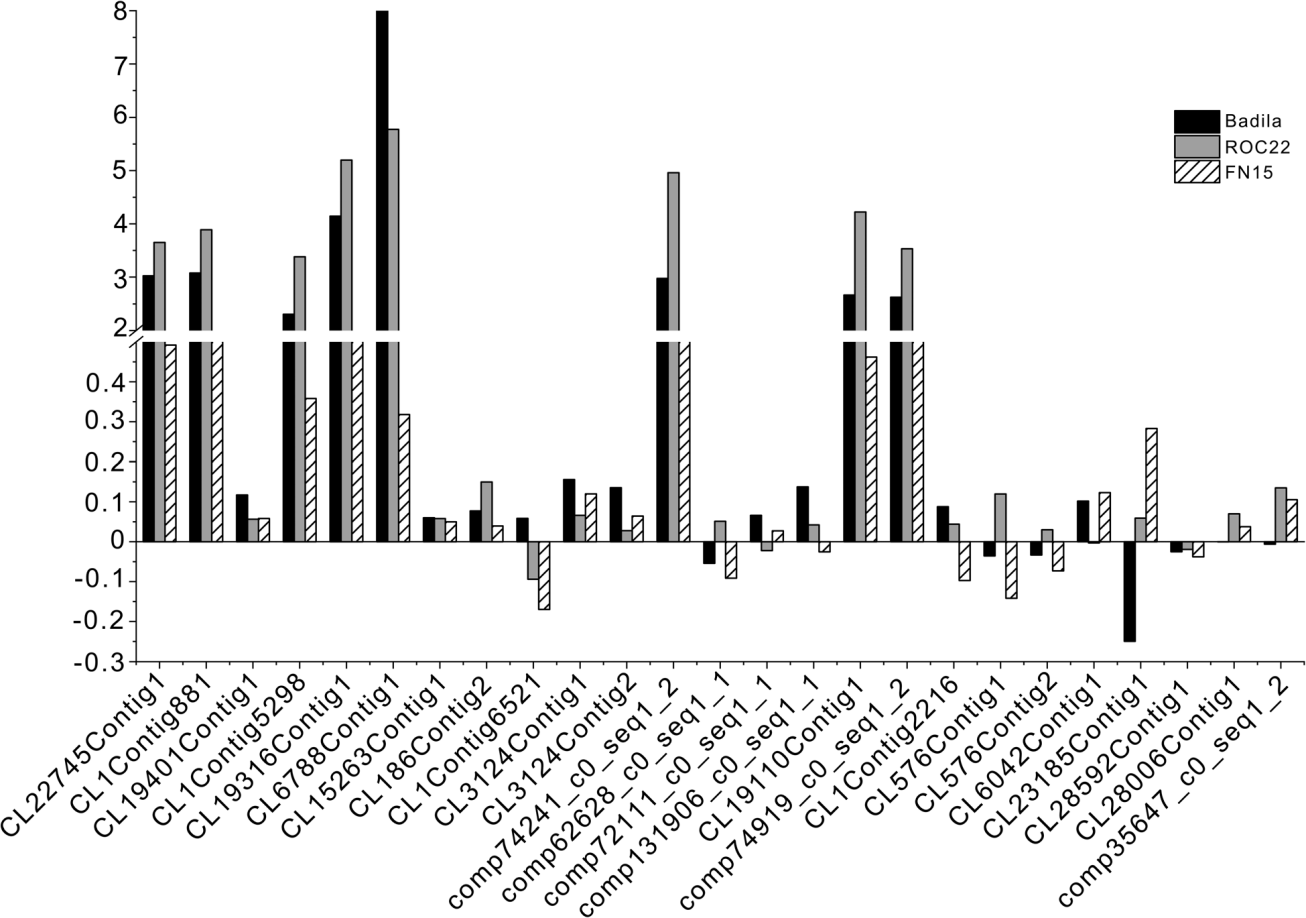
The expression pattern of twenty-five genes obtained from RT-qPCR experiments Relative expression values, normalized to NADPH, were shown as 2^−ΔΔCt^ relative to mean expression levels in the pith

To validate the RNA-seq data, we selected 25 unigenes for RT-qPCR analysis. Those genes belong to the anthocyanin and flavonoid biosynthetic pathways, and their sequences are presented in supplementary file1. Three genes (CL22745Contig1, CL1Contig881, CL19401Contig1) belong to the CHS family, two genes (CL1Contig5298, CL19316Contig1) belong to the CHI family, one gene (CL6788Contig1) belong to the LDOX family, three genes (CL15263Contig1, CL186Contig2, CL1Contig6521) belong to F3’5’H family, two genes (CL3124Contig1, CL3124Contig2) belong to the F3’H family, three genes (CL1Contig2216, CL576Contig1, CL576Contig2) belong to the FLS family, five genes ( CL6042Contig1, CL23185Contig1,CL28592Contig1, CL28006Contig1, comp35647_c0_seq1_2) belong to the LDOX family, four genes (comp62628_c0_seq1_1, comp72111_c0_seq1_1, comp74241_c0_seq1_2, comp131906_c0_seq1_1) belong to the UFGT family, one gene (CL19110Contig1) belong to the BZ2 family, one gene (comp74919_c0_seq1_2) belong to the MYB family (Table 2). We used the gene expression level in the rind minus that in the pith derived from the same variety of sugarcane as the relative expression level of genes. As shown in Figs. 3 and 64 % of qRT-PCR results were consistent with those obtained with the RNA-seq experiments. These data suggested that the expression patterns deduced from the FPKM values in our transcriptome analyses were reliable and can be used in downstream gene expression analyses.

**Table 2:** Putative unigenes involved in anthocyanin and flavonoid biosynthesis between the rind and pith

Unigene_id	Short Gene name	Full Gene Name	Status
CL22745Contig1	ScCHS1	Chalcone synthase	complete
CL1Contig881	ScCHS2	Chalcone synthase	complete
CL19401Contig1	ScCHS3	Chalcone synthase	partial
CL1Contig5298	ScCHI1	Chalcone–flavonone isomerase	complete
CL19316Contig1	ScCHI2	Chalcone–flavonone isomerase	complete
comp30564_c0_seq2_1	ScF3H	Flavanone 3-hydroxylase	complete
CL3124Contig1	ScF3’H1	Flavonoid 3’-monooxygenase	partial
CL3124Contig2	ScF3’H2	Flavonoid 3’-monooxygenase	partial
CL15263Contig1	ScF3’5’H1	Flavonoid 3’,5’-hydroxylase	partial
CL186Contig2	ScF3’5’H2	Flavonoid 3’,5’-hydroxylase	partial
CL1Contig6521	ScF3’5’H3	Flavonoid 3’,5’-hydroxylase	partial
CL6788Contig1	ScLDOX	Leucoanthocyanidin dioxygenase	complete
comp74919_c0_seq1_2	ScMYB	Myeloblastosis	complete
CL19110Contig1	ScBZ2	Glutathione S-transferase Bronze2	complete
CL6042Contig1	ScANR1	Anthocyanidin reductase	Partial
CL23185Contig1	ScANR2	Anthocyanidin reductase	Complete
CL28592Contig1	ScANR3	Anthocyanidin reductase	partial
CL28006Contig1	ScANR4	Anthocyanidin reductase	partial
comp35647_c0_seq1_2	ScANR5	Anthocyanidin reductase	partial
CL15295Contig1	ScANR6	Anthocyanidin reductase	partial
CL1Contig4941	ScANR7	Anthocyanidin reductase	partial
CL1Contig2216	ScFLS1	Flavonol synthase	Partial
CL576Contig1	ScFLS2	Flavonol synthase	Partial
CL576Contig2	ScFLS3	Flavonol synthase	partial
CL29339Contig1	ScBZ1_1	Anthocyanidin 3-O-glucosyltransferase	complete
comp74241_c0_seq1_2	ScBZ1_2	Anthocyanidin 3-O-glucosyltransferase	complete
CL18056Contig1	ScBZ1_3	Anthocyanidin 3-O-glucosyltransferase	complete
comp62628_c0_seq1_1	ScBZ1_4	Anthocyanidin 3-O-glucosyltransferase	partial
comp73769_c1_seq6_2	ScBZ1_5	Anthocyanidin 3-O-glucosyltransferase	complete
comp132391_c0_seq1_2	ScBZ1_6	Anthocyanidin 3-O-glucosyltransferase	partial
comp59267_c1_seq1_2	ScBZ1_7	Anthocyanidin 3-O-glucosyltransferase	partial
comp72111_c0_seq1_1	ScBZ1_8	Anthocyanidin 3-O-glucosyltransferase	partial
comp131906_c0_seq1_1	ScBZ1_9	Anthocyanidin 3-O-glucosyltransferase	partial
CL150Contig1	ScGT1_1	Anthocyanidin 5,3-O-glucosyltransferase	complete
CL1Contig3595	ScGT1_2	Anthocyanidin 5,3-O-glucosyltransferase	complete
CL8117Contig1	ScGT1_3	Anthocyanidin 5,3-O-glucosyltransferase	complete
comp53603_c2_seq1_1	ScGT1_4	Anthocyanidin 5,3-O-glucosyltransferase	Partial
CL1Contig4549	ScGT1_5	Anthocyanidin 5,3-O-glucosyltransferase	partial
CL16968Contig1	ScGT1_6	Anthocyanidin 5,3-O-glucosyltransferase	partial
comp43983_c0_seq1_2	ScGT1_7	Anthocyanidin 5,3-O-glucosyltransferase	partial
comp55939_c2_seq1_1	ScGT1_8	Anthocyanidin 5,3-O-glucosyltransferase	partial
CL3948Contig1	ScGT1_9	Anthocyanidin 5,3-O-glucosyltransferase	partial
comp111837_c0_seq1_2	ScGT1_10	Anthocyanidin 5,3-O-glucosyltransferase	partial
comp103236_c0_seq1_2	ScGT1_11	Anthocyanidin 5,3-O-glucosyltransferase	partial
CL1Contig2451	Sc5MaT_1	Malonyl-CoA: anthocyanidin 5-O-glucoside-6’’-O-malonyltransferase	complete
CL1Contig4024	Sc5MaT_2	Malonyl-CoA: anthocyanidin 5-O-glucoside-6’’-O-malonyltransferase	complete
CL7057Contig1	Sc5MaT_3	Malonyl-CoA: anthocyanidin 5-O-glucoside-6’’-O-malonyltransferase	complete
CL3290Contig1	Sc3MaT_1	Malonyl-coenzyme A: anthocyanin 3-O-glucoside-6’’-O-malonyltransferase	complete
CL1Contig5025	ScMF_1	O-methyltransferase	partial
CL1379Contig2	ScMF_2	Caffeoyl-CoA O-methyltransferase	partial

### Differentially expressed genes in the rind and pith of sugarcane

To identify the Differentially Expressed Genes (DEGs) in the rind and pith of sugarcane, we first analyzed the DEGs between the rind and the pith tissues for each variety. There were 2,559 up and 703 down-regulated unigenes between Badila_rind vs. Badila_pith. In contrast, there were 2,138 up and 999 down-regulated unigenes between ROC22_rind vs. ROC22_pith. Lastly, there were 1,687 up and 1,732 down-regulated unigenes between FN15_rind vs. FN15_pith. A total of 1872 DEGs between rind and pith of sugarcane have expression profiles that correlate with the anthocyanin’s contents according to the Pearson correlation coefficient is a threshold value of 0.9 (Table S2).

**Fig. 4:**
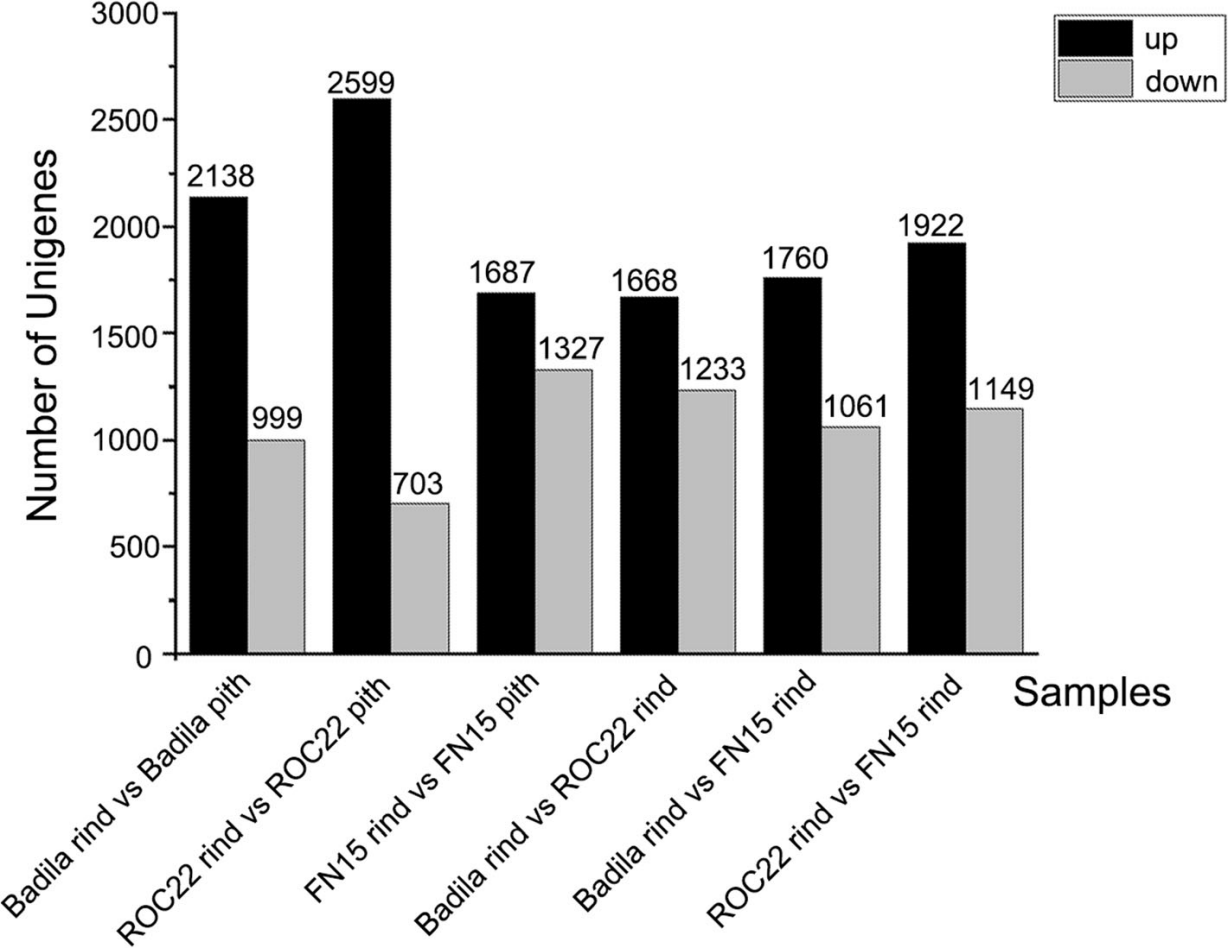
Statistics of differentially expressed genes (DEGs) The “X” axis the alignment combination between sugarcane rind and pith, and the “Y” axis shows the transcript number

Then, we identified DEGs in the rind tissues of the three varieties. There were 1,760 up-regulated transcripts and 1,061 down-regulated transcripts between Badila (purple rind) and FN15 (green rind); 1,922 up-regulated transcripts and 1,149 down-regulated transcripts between ROC22 (red rind) and Badila (purple rind); 1,668 up-regulated transcripts and 1,233 down-regulated transcripts between FN15 (green rind) and ROC22 (red rind) (Fig. 4). A total of 1746 DEGs between rinds of sugarcane have expression profiles that correlate with the anthocyanin’s contents according to the Pearson correlation coefficient is a threshold value of 0.9 (Table S3). Among those related genes, ScLDOX (CL6788Contig1), ScF3H (comp30564_c0_seq2_1), ScGT1_7 (comp43983_c0_seq1_2) are related to anthocyanin biosynthesis.

Next, we identified the DGEs between the rind and pith tissues of the three varieties. The comparison results of these DGEs are shown in the Venn diagram (Fig. S5). As shown, 637 DGEs were shared among all three sugarcane varieties. And there are 200, 250, and 426 DEGs shared between Badila and ROC22, Badila and FN15, ROC22, and FN15, respectively (Fig. S5).

Lastly, we compared gene expression levels in the rind tissues of the three varieties. Using the expression level in the rind of FN15 sugarcane as a control, we determine DGEs between Badila and FN15, ROC22, and FN15. The results are shown in Fig. S6. There are 2821 DGEs for Badila/FN15. In contrast, there are 2901 DGEs for ROC22/FN15. Lastly, there are 574 DGEs shared between the two sets.

### Enrichment analysis of DEGs

**Fig. 5:**
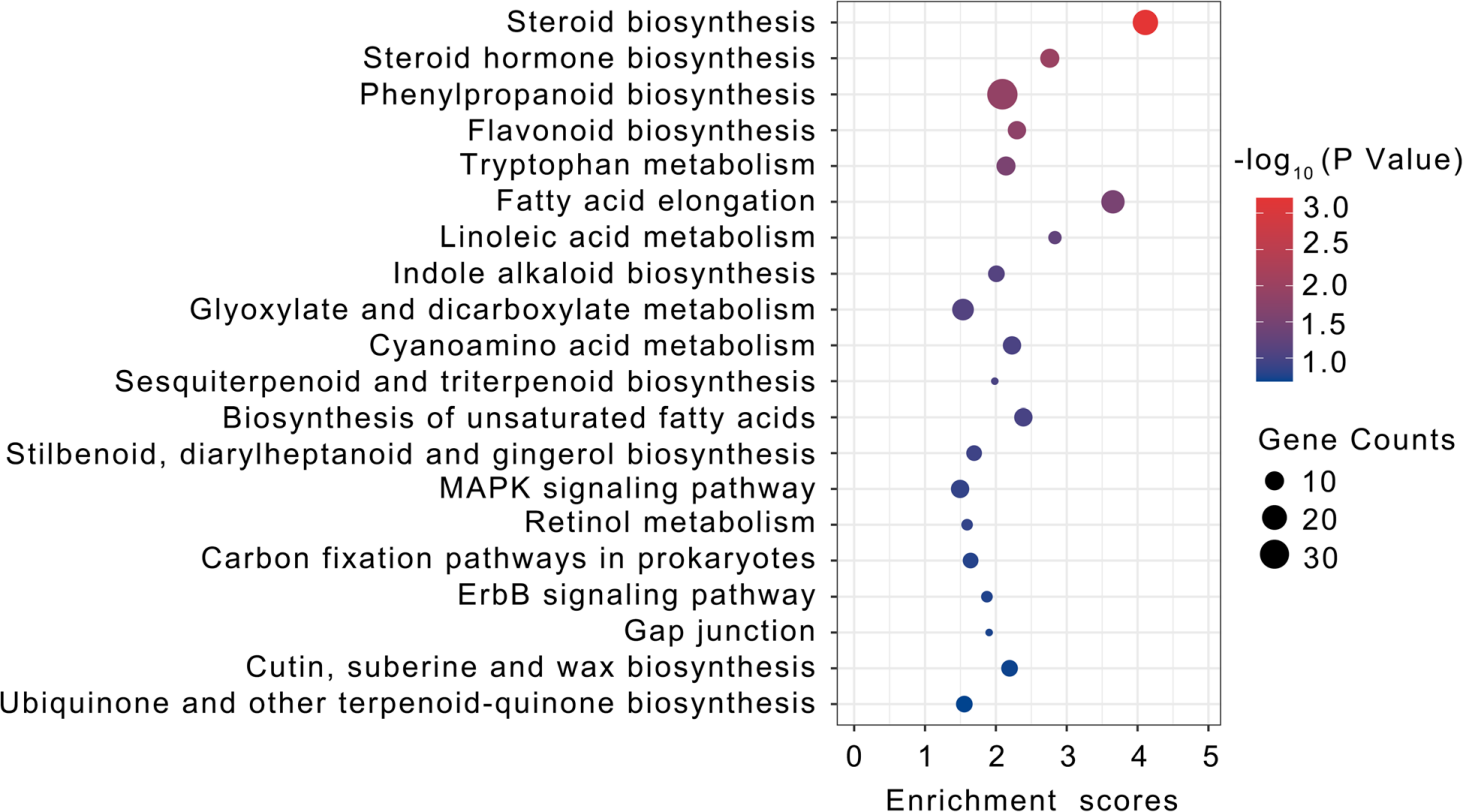
KEGG enrichment of DEGs between the rind and pith of different sugarcane varieties The “X” axis is the enrichment score; the “Y” axis the number of DEGs mapped to each KEGG pathway

The DEGs identified from the six pairs of comparisons were further subjected to KEGG pathway enrichment analyses to screen genes associated with anthocyanin biosynthesis in the rind and pith tissues. The top 20 enriched pathways included the following: steroid biosynthesis, steroid hormone biosynthesis, phenylpropanoid biosynthesis, flavonoid biosynthesis, tryptophan metabolism, fatty acid elongation, linoleic acid metabolism, indole alkaloid biosynthesis, glyoxylate, and dicarboxylate metabolism, cyanoamino acid metabolism, sesquiterpenoid, and triterpenoid biosynthesis, biosynthesis of unsaturated fatty acids, stilbenoid, diarylheptanoid, and gingerol biosynthesis, MAPK signaling pathway, retinol metabolism, carbon fixation pathways in prokaryotes, ErbB signaling pathway, gap junction, cutin, suberine, and wax biosynthesis, ubiquinone and another terpenoid-quinone biosynthesis (Fig. 5). In particular, phenylpropanoid biosynthesis is the most enriching pathway. Anthocyanin biosynthesis and flavonoid biosynthesis were all enriched in all the above comparisons. The result showed the DEGs were enriched in many metabolic processes that included flavonoid and anthocyanin biosynthesis pathways.

### Identification of candidate genes related to anthocyanin biosynthesis in the rind

We identified the putative genes related to anthocyanin biosynthesis based on the sequence similarity to those genes in the KEGG pathways for flavonoid biosynthesis (ko00941) and anthocyanin biosynthesis (ko00942). As shown in Tables 2, we identified a total of 51 genes. These included the following: three CHS (chalcone synthase), two CHI (chalcone–flavanone isomerase), one F3H (Flavanone 3-hydroxylase), two F3’H (flavanone 3’-hydroxylase), three F3’5’H (flavonoid-3’,5’-hydroxylase), one LDOX (leucoanthocyanidin dioxygenase), one MYB (myeloblastosis), one BZ2 (Bronze 2), seven ANR (anthocyanidin reductase), three FLS (flavonol synthase), eight BZ1 (anthocyanidin 3-O-glucosyltransferase), eleven GT1(anthocyanidin 5,3-O-glucosyltransferase), three 5MaT (malonyl-CoA: anthocyanidin 5-O-glucoside-6’’-O-malonyltransferase), one 3MaT (malonyl-coenzyme A: anthocyanin 3-O-glucoside-6’’-O-malonyltransferase) and two MF (O-methyltransferase). To validate the full-length coding sequences, we conducted multiple sequence alignment and phylogenetic analysis for these genes: CHS (Fig. S7), CHI (Fig. S8), F3H (Fig. S9), LDOX (Fig. S10), BZ2 (Fig. S11), MYB (Fig. S12), ANR (Fig. S13), BZ1 (Fig. S14), GT1 (Fig. S15), 5MaT (Fig. S16), 3MaT (Fig. S17). As shown in the multiple sequence alignment, these genes are highly conserved among sugarcane and other plants.

**Fig. 6:**
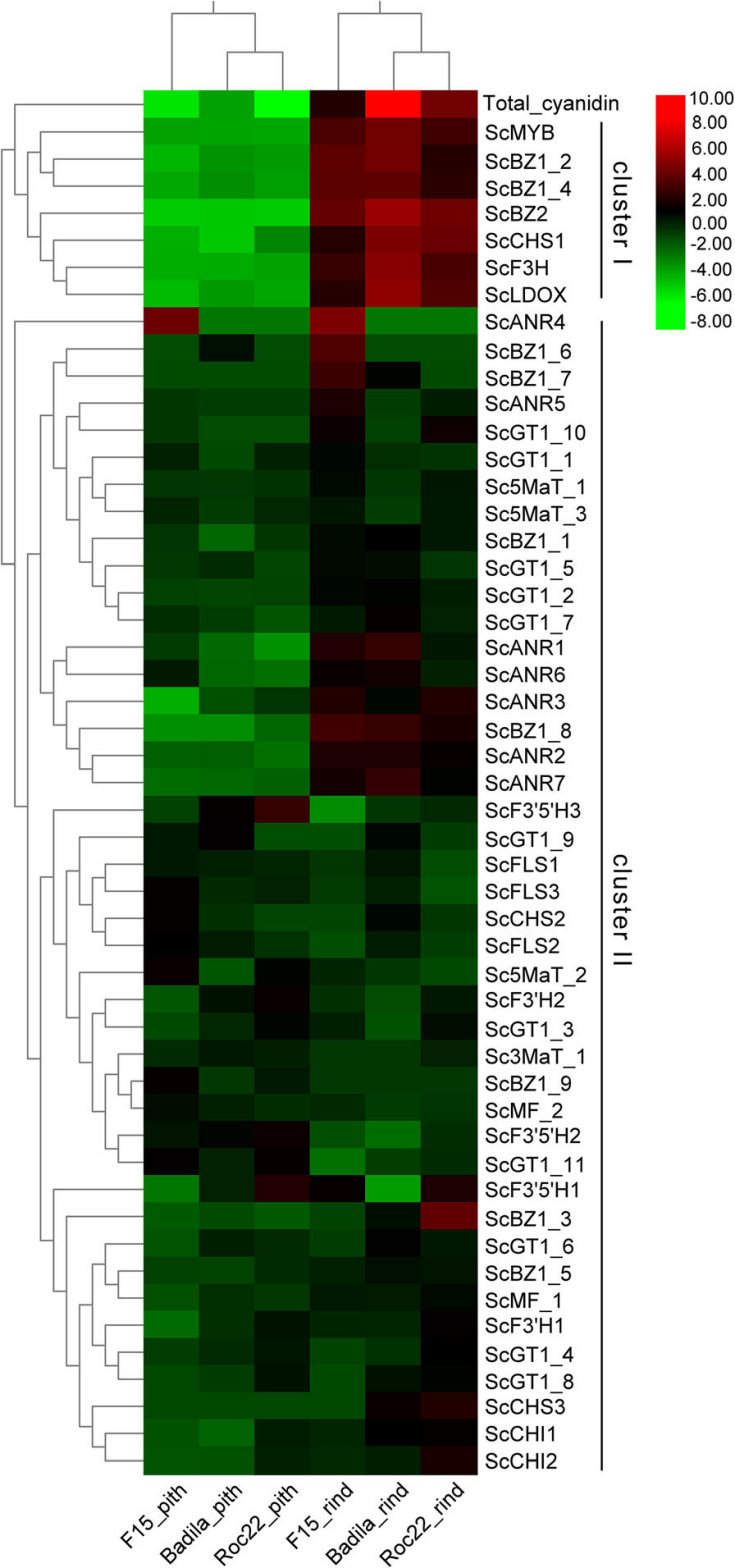
Hierarchical clustering of 50 flavonoid and anthocyanin biosynthesis genes. The scale bar denotes the log2 (FPKM + 1)/ (mean expression levels across the three treatment groups). The color represents relative gene expression levels. While the horizontal direction shows the sample names, the vertical direction shows the names of genes involved in the biosynthesis of flavonoids and anthocyanins

To study the co-expression patterns of these putative genes related to anthocyanin biosynthesis, we performed hierarchical clustering of these 51 genes’ expression profiles and the content of derivative of cyanidin using the Euclidean distance as the metric and Ward’s method. As shown in Fig. 6, two main clusters were readily discernable, which were named C1 and C2. The clusters C1 containing seven unigenes showed the highest expression levels in the rind, which had the highest levels of cyanidin derivatives. The seven unigenes were ScCHS1, ScF3H, ScLDOX, ScMYB, ScBZ2, ScBZ1_2, ScBZ1_4. All of them except ScBZ1_4 appeared to have the full length of the coding sequences. These genes are likely to play important roles in anthocyanins biosynthesis, and their exact functions will be the subject of future investigation.

**Fig. 7:**
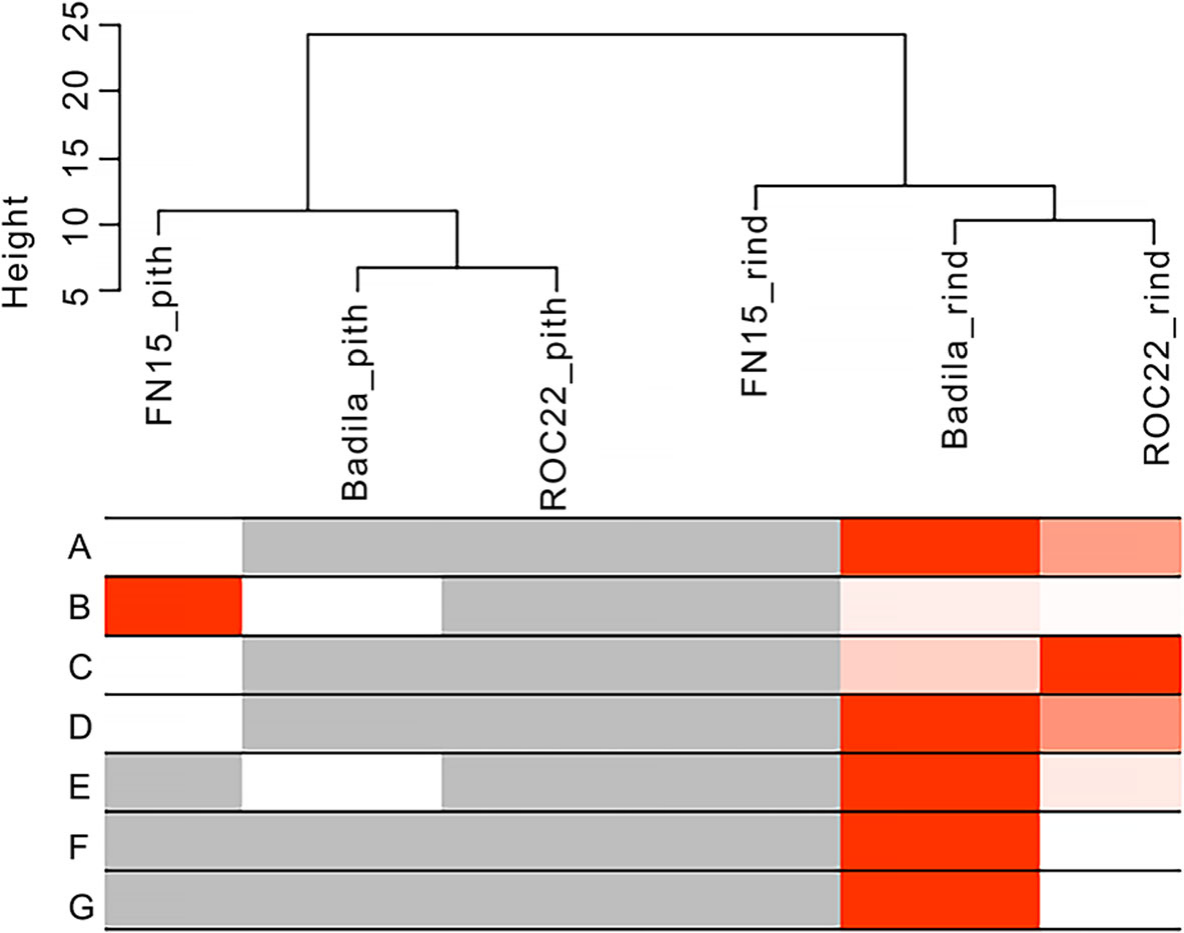
Weighted correlation network analysis of sugarcane anthocyanin biosynthesis genes and anthocyanins. Hierarchical clustering of the samples is shown on the top. The height represents the relative distance among the samples based on the gene expression profile [37]. The correlations of various anthocyanin compounds and different samples are shown at the bottom. The grey means a low value, red means a high value, and white means a missing entry. Cyanidin (**a**); Malvidin (**b**); Pelargonidin (**c**); Peonidin (**d**); Cyanidin (6’-malonylglucoside) (**e**); Cyanidin O-glucoside (**f**); Peonidin O-glucoside (**g**)

To further investigate the relationship between DEGs and the abundance of anthocyanin compounds, we performed WGCNA analysis. As shown in Fig. 7, all the rind samples were clustered together, and all the pith samples were clustered together. In the purple rind, the expression levels of the members of the gene module were upregulated. The gene modules have the highest correlation with the abundance of those anthocyanin compounds, including cyanidin, pelargonidin, peonidin, cyanidin (6’-malonylglucoside), cyanidin O-glucoside, and peonidin O-glucoside. Interestingly, the abundance of malvidin was highly correlated with the gene expression profiles in the green rind sugarcane.

## Discussion

### Anthocyanin identification in the rind and pith of sugarcane

Anthocyanins are secondary metabolites distributed widely in plants such as vegetables, fruits, and medical plants. Several efforts have been made to increase anthocyanin content in particular plant tissues. For example, the anthocyanin contents have been increased in the purple heart cabbage[38]. Anthocyanin glycosylation modifications affect the stability of anthocyanins in cells. The first step after anthocyanin biosynthesis in both purple potato and Arabidopsis is glycosylation to form anthocyanin-3-O-glucoside. However, the downstream glycosylation modifications become entirely different. In Arabidopsis, a xylose group is transferred to the C2 position of anthocyanin-3-O-glucoside catalyzed by glycosyltransferase At3GGT (UGT79B1), whereas in purple potato, a glucose molecule is presumably transferred to the C2 position of anthocyanin-3-O-glucoside catalyzed by glycosyltransferase (Ib3GGT) to form anthocyanin-3-O-sophoroside [39].

Our long-term goal is to breed purple-hearted sugarcane with high anthocyanin content. Here, we conducted a combined metabolomic and transcriptomic analysis to identify genes involved in anthocyanins’ biosynthesis and regulation. A total of 7 anthocyanins were identified in the rind and pith of sugarcane by UPLC-Q-TOF/MS (Table 1). We found that the derivative of cyanidin is the determinant of the rind color of sugarcane.

The results from this study might be related to other anthocyanins’ effects. Firstly, Increased expression of genes involved in the phenylalanine pathway leads to increased levels of polyphenols [40]. Polyphenolic compounds such as anthocyanins may be subject to browning by the action of polyphenol oxidase during sugar extraction [41, 42]. However, previous reports have found that sucrose concentrations of around 20 % were protective against anthocyanin browning. [43]. Besides, the browning of anthocyanin-rich fruit juice was affected by pH, and MA (monomeric anthocyanin content). And exogenous application of ascorbic acid had a preventive effect on phloem discoloration. [42, 44]. In conclusion, the enrichment of anthocyanins during the extraction of sugar cane can cause browning. Still, at the same time, effective measures can be taken to reduce the degree of browning significantly.

Three issues need attention. Firstly, there are six natural anthocyanins. However, we detected four of them and their glycosyl derivatives, as we did not detect two natural anthocyanins, delphindin and petunidin. There are two possible reasons: (1) the sample preparation method is not optimal for the extraction of the substance, and the substance is not in the extraction solution; (2) the substance was extracted successfully, but the contents were so low that they might be below the detection limitations of the instrument.

Secondly, it is generally considered that the content of malvidin correlates with the tissue color. However, the content of malvidin is higher in the pith (less colorful) than rind (more colorful), especially for FN15 and ROC22 (Table 1) on the contrary to the general thoughts. This may be due to the complex color-forming mechanism of plants. For example, co-pigmentation of anthocyanins in plant tissues under different anthocyanin combinations or PH values could show different results [45]. The exact reason is currently unclear and needs further investigation.

Thirdly, the mass spectrometry analysis did not deduce the anthocyanin’s accurate structure because there are many isomers of anthocyanins with different glucoside forms lacking standard compounds. Therefore, we can only make a preliminary qualitative and quantitative analysis of anthocyanins’ composition and content by analyzing anthocyanins’ mass spectrum information.

### Candidate genes involved in anthocyanins biosynthesis of sugarcane

Through correlation analysis with anthocyanin content in sugarcane, we found that the expression profiles of 7 genes correlate well with those of the anthocyanin abundance. The seven genes were ScCHS1, ScF3H, ScLDOX, ScMYB, ScBZ2, ScBZ1_2, ScBZ1_4. Overexpression of the CHS gene in rice causes anthocyanin accumulation [46]. A transcriptional activation complex composed of R2R3-MYB, basic-helix-loop-helix (bHLH), and WD40 proteins (named MBW complex) has been shown to control the expression of anthocyanin structural genes[47]. F3H mutant cells cannot synthesize anthocyanins and remain white [48]. The Arabidopsis TDS4 gene encodes leucoanthocyanidin dioxygenase (LDOX). It is essential for proanthocyanidin synthesis and vacuole development [49]. The glutathione S-transferase encoded by Bronze2 (BZ2) performs the last genetically defined step in maize anthocyanin biosynthesis, being required for pigment sequestration into vacuoles [50]. Based on the above results, we speculate that the different expression patterns of anthocyanin biosynthesis and related regulatory genes contribute to sugarcane color. This information sheds light on the evolution of anthocyanin glycosylation among other plants, which will provide new ideas for producing specific anthocyanin compounds through genetic engineering.

### Putative pathway of biosynthesis of anthocyanin in the sugarcane rind

**Fig. 8:**
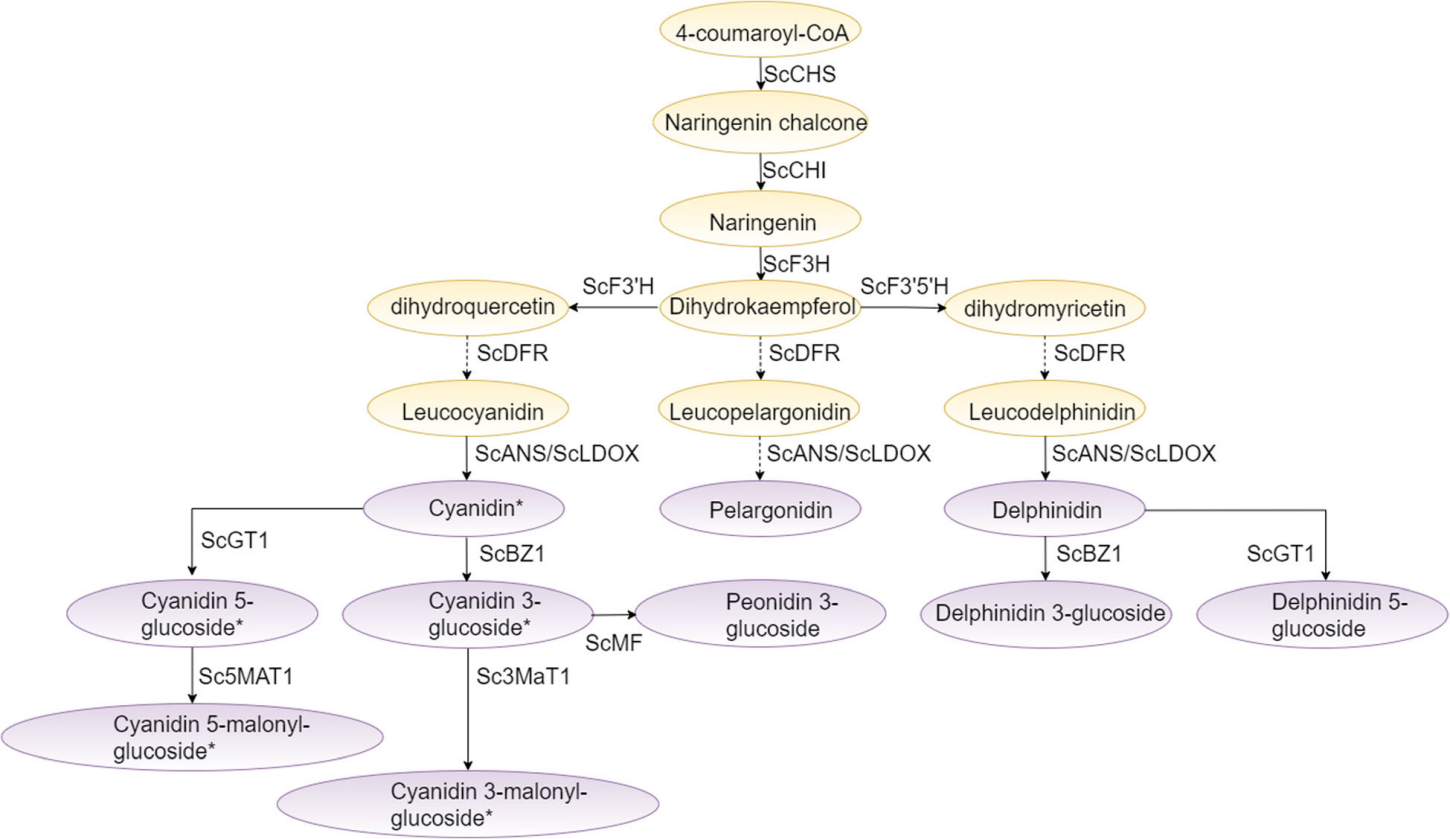
A proposed biosynthetic pathway of anthocyanin in the rind of sugarcane. Compounds are shown in ovals. The genes that catalyze each chemical reaction is shown above or to the right of the arrows. The compounds shown in yellow are those found in wide varieties of plant species. They represent the common upstream pathway. The compounds shown in peak are those specific for different plant species. The cyanidin and glycosylated cyanidin were found to be most abundant in the rind of sugarcane samples studied. All the compounds shown in peak were detected in this study. The cyanidin and its derivatives are the most abundant in sugarcane (indicated with *)

The anthocyanin biosynthesis pathway is conserved in higher plants (Naing and Kim, 2018). We identified several enzyme-coding structural genes involved in anthocyanins biosynthesis [31]. They include the following: phenylalanine-ammonia lyase (PAL), 4-coumaryl: CoA ligase (4CL), chalcone synthase (CHS), chalcone isomerase (CHI), flavonoid-3′-hydroxylase (F3′H), flavonoid-3′,5′-hydroxylase (F3′5′H), flavanone 3-hydroxylase (F3H), dihydroflavonol 4-reductase (DFR), anthocyanidin synthase (ANS), and UDP-glucose: flavonoid 3-O-glucosyltransferase (UFGT) [51]. So far, the biosynthesis mechanism of anthocyanin in the rind of sugarcane is not clear. In this study, we discovered that cyanidin’s derivative is the main factor affecting sugarcane’s rind color. By analyzing the transcriptome data of three different sugarcane varieties with different rind colors, we hypothesized the putative pathway of biosynthesis of anthocyanin in the rind of sugarcane (Fig. 8). The putative pathway contains 15 protein families, including CHS, CHI, F3H, F3’H, F3`5’H, ANR, BZ2, MYB, GT1, FLS, BZ1, ANS, 5MaT, 3MaT, and MF. In this study, we did not identify the DRX gene in the rind and pith of sugarcane, which may be due to the gene’s tissue-specific expression [52]. These results will further improve understanding of this anthocyanin biosynthesis pathway in the rind of sugarcane.

## Conclusions

This study investigated anthocyanin and flavonoid biosynthesis in sugarcane rind and pith using the combined transcriptomic and metabolomic methods. Through UPLC analysis of anthocyanin compounds in sugarcane, we found that cyanidin derivatives were the main factor for the color difference of sugarcane rind. Secondly, we conducted the comparative transcriptome analysis to identify the DEGs between the rind and pith of sugarcane varieties. Thirdly, we identified 51 putative genes related to anthocyanin and flavonoid biosynthesis based on the sequence similarity. Fourthly, seven genes were identified as candidate genes related to anthocyanin and flavonoid biosynthesis through the correlation analysis with cyanidin derivatives content. Finally, we proposed a hypothetical molecular model to explain anthocyanin’s biosynthesis and its glycoside derivatives in sugarcane. These results lay the foundation for improving anthocyanin production in sugarcane through genetic engineering and molecular breeding. This research provides valuable resources for the study of sugarcane anthocyanins and provides a molecular basis for improving sugarcane anthocyanin genetic breeding.

## Supplementary Information

**Additional file 1.**

**Additional file 2.**

**Additional file 3.**

## Data Availability

The raw reads generated for this study have been deposited in BioProject with the accession number: PRJNA573557 (https://www.ncbi.nlm.nih.gov/bioproject/PRJNA666228).

## Abbreviations

RNA-seq: RNA-seq sequencing
UPLC-Q-TOF/MS: Ultra-high-performance liquid chromatography-quadrupole time-of-flight mass spectrometry
DEGs: Differentially expressed genes; RT-qPCR: Real-time quantitative reverse transcription-polymerase chain reaction
ORF: Open reading frame
WGCNA: Weighted correlation network analysis
