# Supplementary material for: Discovery of genes involved in anthocyanin biosynthesis from the rind and pith of three sugarcane varieties using integrated metabolic profiling and RNA-seq analysis
Source: BMC Plant Biol. 2021 May 12;21:214. doi: 10.1186/s12870-021-02986-8 (PMC8117289; doi:10.1186/s12870-021-02986-8)

## Title:

Discovery of genes involved in anthocyanin biosynthesis from the rind and pith of three sugarcane varieties using integrated metabolic profiling and RNA-seq analysis

**Author list**

Yang Ni^#1^, Haimei Chen^#2^, Di Liu^1^, Lihui Zeng^1^, Pinghua Chen^1^*, Chang Liu^2^*

**Affiliations**

^1^Key Laboratory of Ministry of Education for Genetics, Breeding and Multiple Utilization of Crops, National Engineering Research Center of Sugarcane, College of Agriculture, Fujian Agriculture and Forestry University, Fuzhou, Fujian Province, Fuzhou 350002, P. R. China;

^2^Key Laboratory of Bioactive Substances and Resource Utilization of Chinese Herbal Medicine from Ministry of Education, Engineering Research Center of Chinese Medicine Resources from Ministry of Education, Institute of Medicinal Plant Development, Chinese Academy of Medical Sciences, Peking Union Medical College, Beijing 100193, P. R. China.

^#^These authors contributed equally to this work.

*****Correspondence: Pinghua Chen: [phcemail@126.com](mailto:phcemail@126.com) (PHC); Tel: +86-0591-83789177, Fax: +86-0591-83768242; Chang Liu: [cliu6688@yahoo.com](mailto:cliu6688@yahoo.com) (CL); Tel: +86-10-57833111, Fax: +86-10- 62899715;

E-mails:

Yang Ni: niyang_work@126.com

Haimei Chen: [hmchen@implad.ac.cn](mailto:hmchen@implad.ac.cn)

Di Liu: [fafuld@126.com](file:///C:\\Users\\Administrator\\AppData\\Roaming\\Microsoft\\Word\\%20fafuld@126.com)

Lihui Zeng: [lhzeng@hotmail.com](file:///C:\\Users\\administrator1\\Documents\\WeChat%20Files\\wxid_hyuarnhoaqq922\\FileStorage\\File\\2020-09\\lhzeng@hotmail.com)

Pinghua Chen: [phcemail@126.com](mailto:phcemail@126.com)

Chang Liu: [cliu6688@yahoo.com](mailto:cliu6688@yahoo.com)

# Supplementary figure

## Fig S1. Sequence length distribution of the unigene sequences. The “X” axis shows the lengths of the transcript sequences. The “Y” axis shows the number of unigenes.


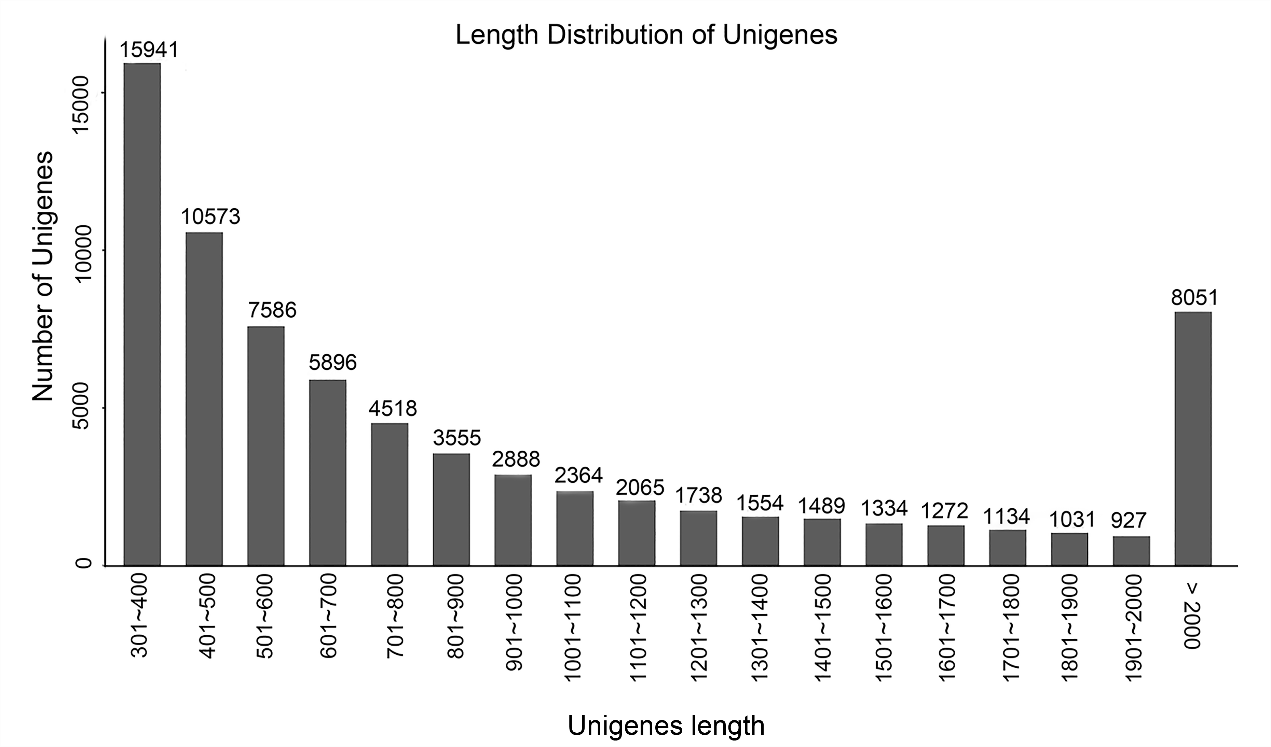


## Fig S2. Venn diagram indicating the results of unigenes annotated from NR, SWISSPROT, KOG, GO, KEGG databases.

**
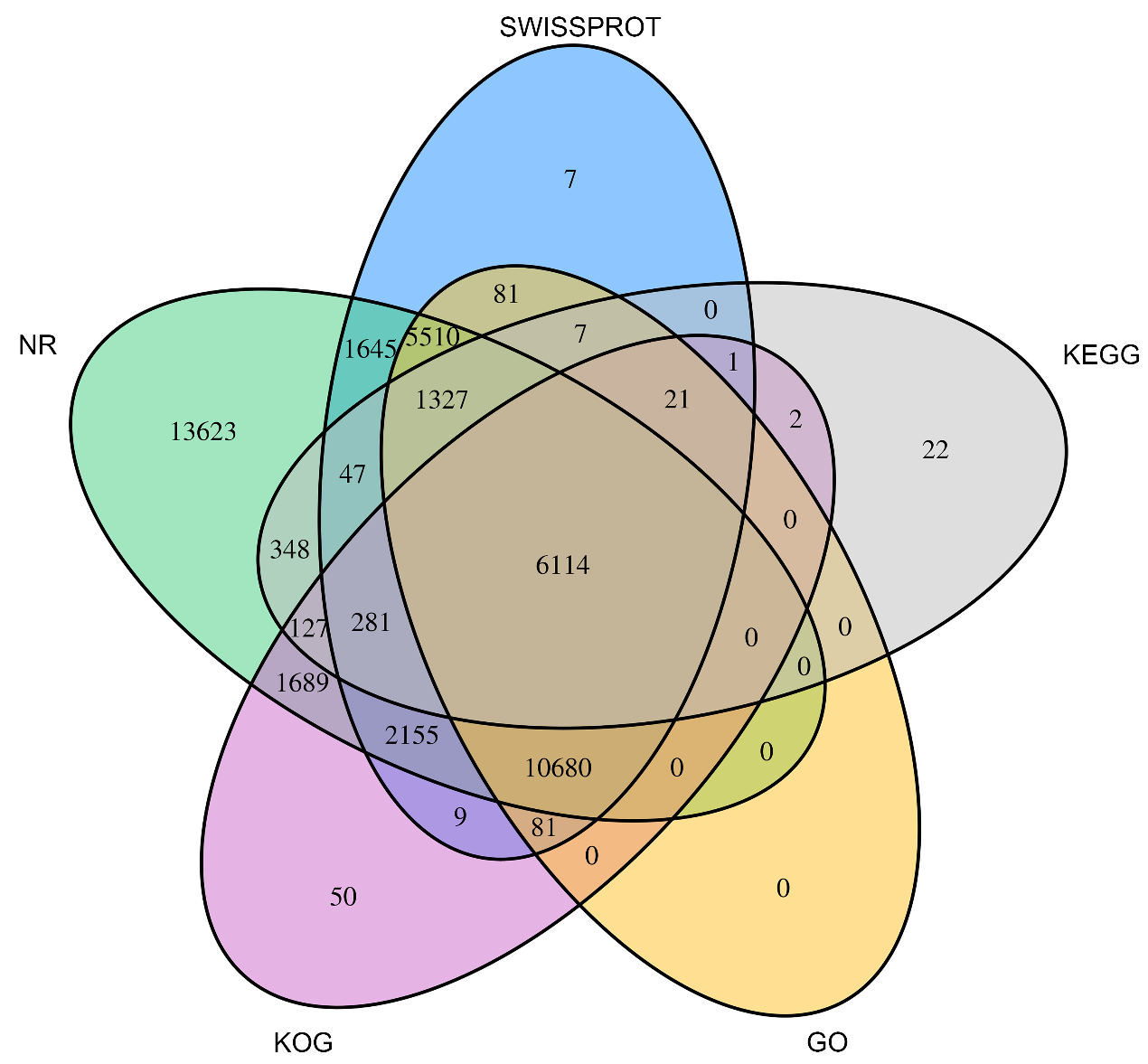
**

## Fig S3. GO classifications of the unigene sequences. Annotated unique sequences were classified into “Biological process” “cellular component” and “Molecular function” respectively. The “X” axis shows classification of genes, the “Y” axis shows the percentage and number of unigenes.


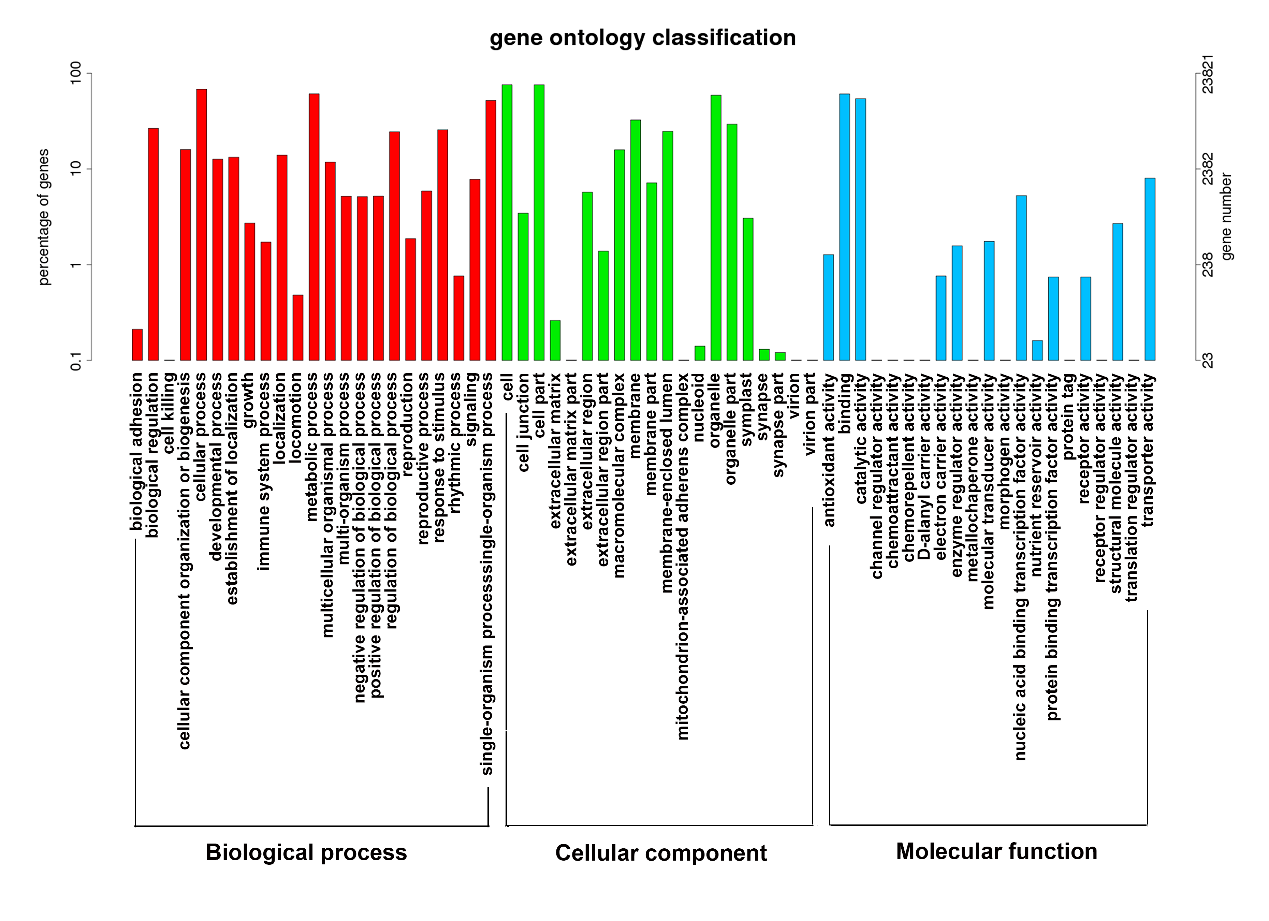


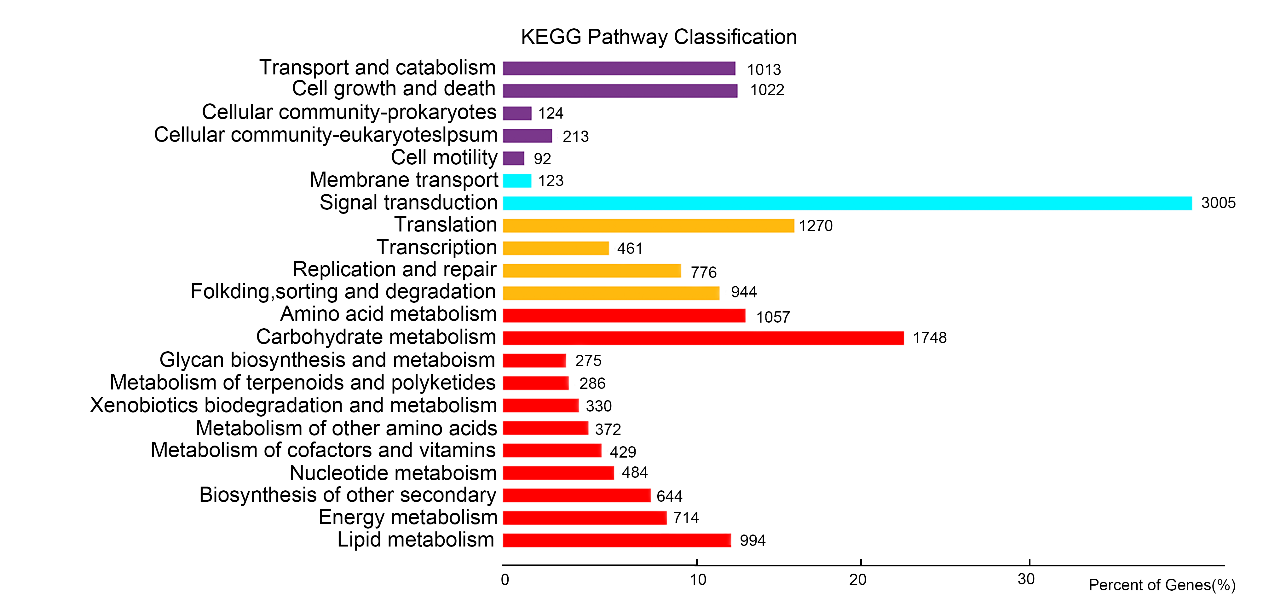


## Fig S4. KEGG classifications of all unique sequences. Purple is Cellular Processes; Blue is Environmental Information Processing; Orange is Genetic Information Processing; D: Red is Metabolism. “X” axis is the percentage of total genes mapped to each KEGG pathway; the “Y” axis is the KEGG pathway. The numbers to the right of each bar represent the number of genes mapped to the pathway.


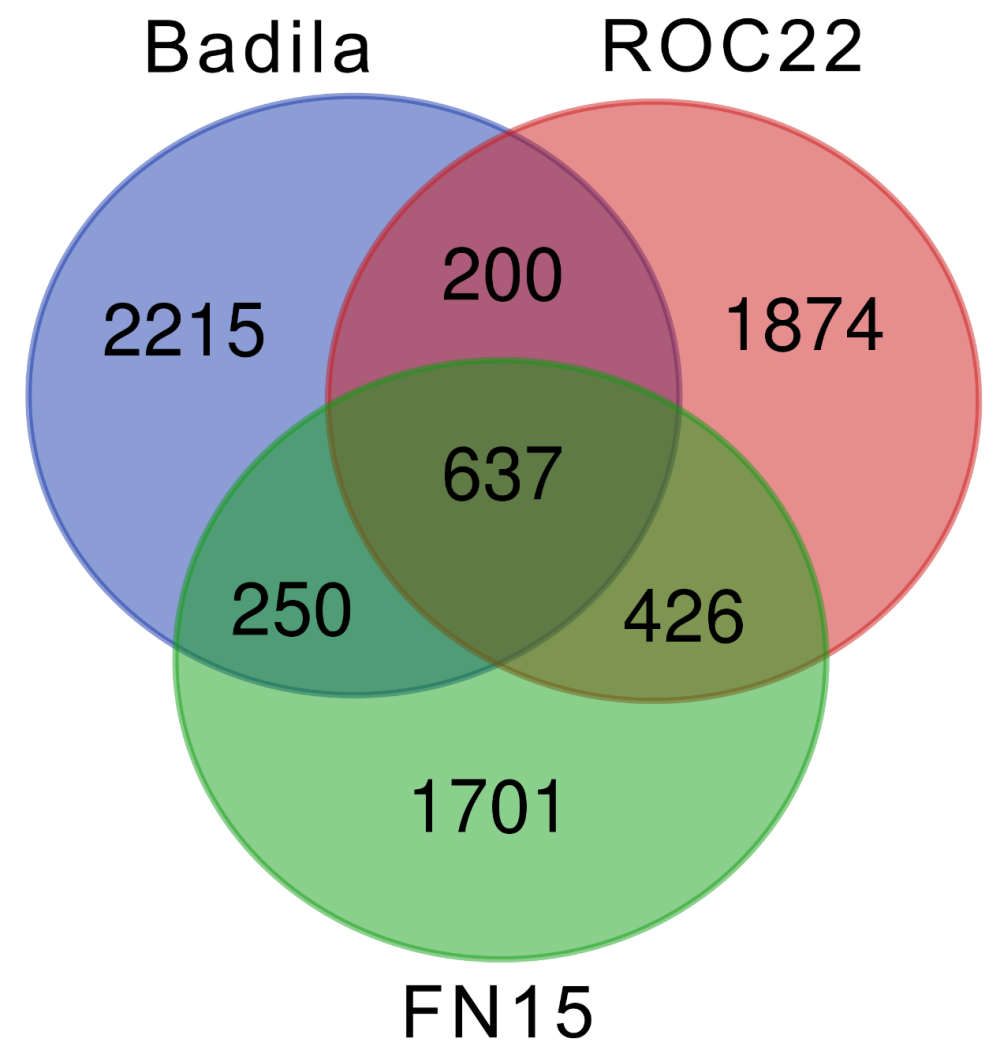


## Fig S5. Venn diagrams of DEGs between the rind and pith tissues of the three varieties.

##
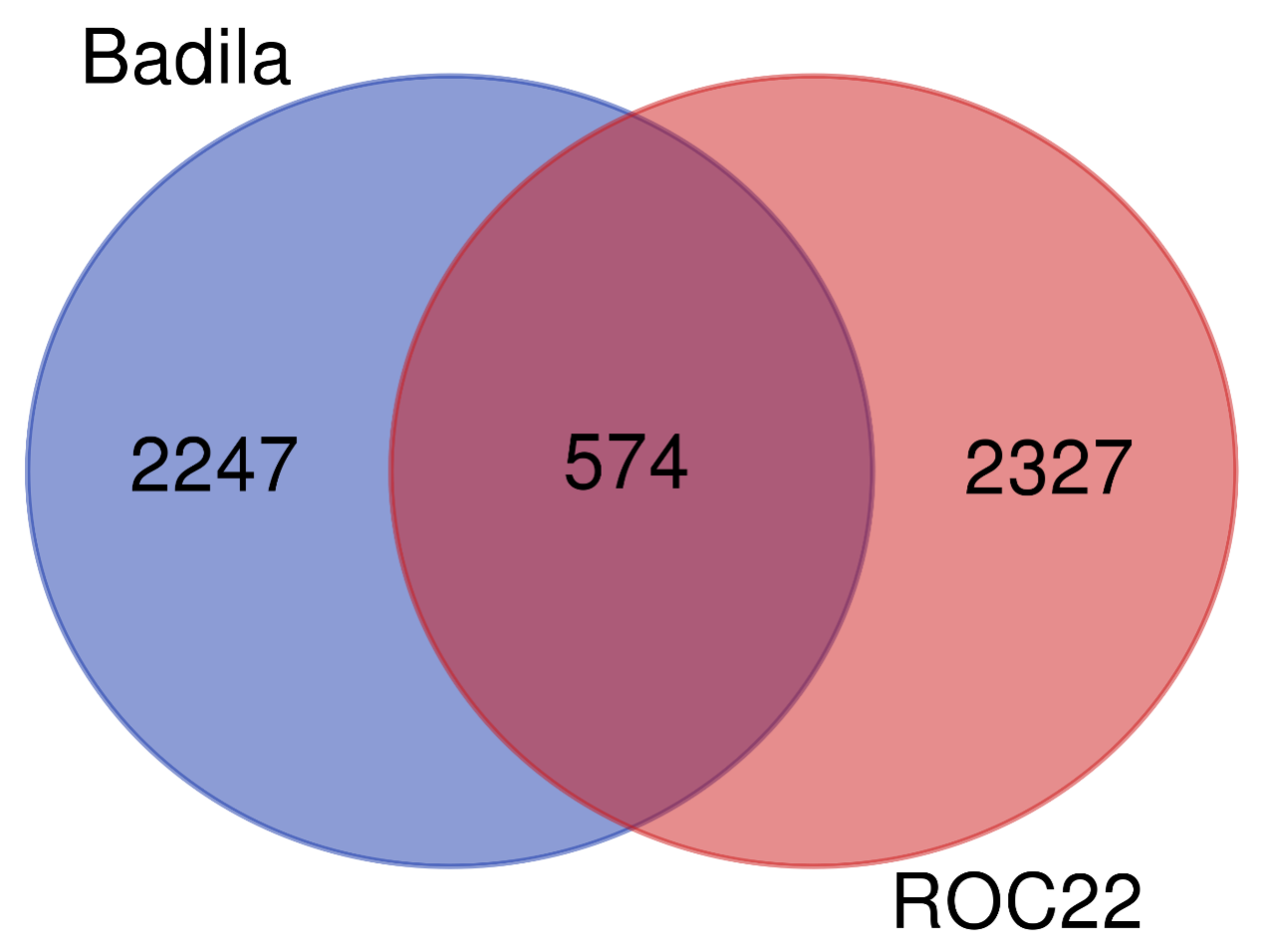
Fig S6. Venn diagrams of DEGs of sugarcane rind.

## Fig S7. Sequence alignment of chalcone synthase (CHS) proteins from sugarcane and various other plants, and phylogenetic relationships of chalcone synthase

### (A). Multiple sequence alignment of ScCHS1 gene.

* 20 * 40 * 60
Q9XGX2.1 : MAGATVTVEEVRKAQRATGPATVLAIGTATPANCVHQADYPDYYFRITKSEHMTELKEKF : 60
Q9SBL6.1 : MAAATVTVEEVRKAQRATGPATVLAIGTATPANCVHQADYPDYYFRITKSEHMTDLKEKF : 60
Q9SBL3.1 : MAGATVTVEEVRKAQRATGPATVLAIGTATPANCVHQADYPDYYFRITKSEHMTELKEKF : 60
Q9SBL7.1 : MAGATVTVEEVRKAQRATGPATVLAIGTATPANCVHQADYPDYYFRITKSEHMTELKEKF : 60
Q9SBL4.1 : MAAATVTVEEVRKAQRATGPATVLAIGTATPANCVHQADYPDYYFRITKSEHMTELKEKF : 60
Q9SBL5.1 : MAAATVTVEEVRKAQRATGPATVLAIGTATPANCVHQADYPDYYFRITKSEHMTELKEKF : 60
Q9XGX1.1 : MAGATVTVEEVRKAQRATGPATVLAIGTATPANCVHQADYPDYYFRITKSEHMTDLKEKF : 60
P24825.1 : MAGATVTVEEVRKAQRATGPATVLAIGTATPANCVYQADYPDYYFRITKSEHLTDLKEKF : 60
Q2R3A1.1 : -MAAAVTVEEVRRAQRAEGPATVLAIGTATPANCVYQADYPDYYFRITKSEHMVELKEKF : 59
A2ZEX7.1 : -MAAAVTVEEVRRAQRAEGPATVLAIGTATPANCVYQADYPDYYFRITKSEHMVELKEKF : 59
ScCHS1 : MAGATVTVEEVRKAQRAAGPATVLAIGTATPANCVYQADYPDYYFRITKSEHMTDLKEKF : 60
 ma AtVTVEEVR4AQRA GPATVLAIGTATPANCV QADYPDYYFRITKSEH6t LKEKF

 * 80 * 100 * 120
Q9XGX2.1 : KRMCDKSQIRKRYMHLTEEYLAENPNMCAYMAPSLDARQDIVVVEVPKLGKAAAQKAIKE : 120
Q9SBL6.1 : KRMCDKSQIRKRYMHLTEEYLAENPNMCAYMAPSLDARQDIVVVEVPKLGKAAAQKAIKE : 120
Q9SBL3.1 : KRMCDKSQIRKRYMHLTEEYLAENPNMCAYMAPSLDARQDIVVVEVPKLGKAAAQKAIKE : 120
Q9SBL7.1 : KRMCDKSQIRKRYMHLTEEYLAENPNMCAYMAPSLDARQDIVVVEVPKLGKAAAQKAIKE : 120
Q9SBL4.1 : KRMCDKSQIRKRYMHLTEEYLAENPNMCAYMAPSLDARQDIVVVEVPKLGKAAAHKAIKE : 120
Q9SBL5.1 : KRMCDKSQIRKRYMHLTEEYLAENPNMCAYMAPSLDARQDIVVVEVPKLGKAAAQKAIKE : 120
Q9XGX1.1 : KRMCDKSQIRKRYMHLTEEYLAENPNMCAYMAPSLDARQDIVVVEVPKLGKAAAQKAIKE : 120
P24825.1 : KRMCDKSMIRKRYMHLTEEFLAENPSMCAYMAPSLDARQDVVVVEVPKLGKAAAQKAIKE : 120
Q2R3A1.1 : KRMCDKSQIRKRYMHLTEEILQENPNMCAYMAPSLDARQDIVVVEVPKLGKAAAQKAIKE : 119
A2ZEX7.1 : KRMCDKSQIRKRYMHLTEEILQENPNMCAYMAPSLDARQDIVVVEVPKLGKAAAQKAIKE : 119
ScCHS1 : KRMCDKSQIRKRYMHLTEEYLAENPNMCAYMAPSLDARQDIVVVEVPKLGKAAAQKAIKE : 120
 KRMCDKSqIRKRYMHLTEE LaENPnMCAYMAPSLDARQD6VVVEVPKLGKAAAqKAIKE

 * 140 * 160 * 180
Q9XGX2.1 : WGQPKSKITHLVFCTTSGVDMPGADYQLTKMLGLRPSVNRLMMYQQGCFAGGTVLRVAKD : 180
Q9SBL6.1 : WGQPKSKITHLVFCTTSGVDMPGADYQLTKMLGLRPSVNRLMMYQQGCFAGGTVLRVAKD : 180
Q9SBL3.1 : WGQPKSKITHLVFCTTSGVDMPGADYQLTKMLGLRPSVNRLMMYQQGCFAGGTVLRVAKD : 180
Q9SBL7.1 : WGQPKSKITHLVFCTTSGVDMPGADYQLTKMLGLRPSVNRLMMYQQGCFAGGTVLRVAKD : 180
Q9SBL4.1 : WGQPKSKITHLVFCTTSGVDMPGADYQLTKMLGLRPSVNRLMMYQQGCFAGGTVLRVAKD : 180
Q9SBL5.1 : WGQPKSKITHLVFCTTSGVDMPGADYQLTKMLGLRPSVKRLMMYQQGCFAGGTVLRVAKD : 180
Q9XGX1.1 : WGQPKSKITHLVFCTTSGVDMPGADYQLTKMLGLRPSVNRLMMYQQGCFAGGTVLRVAKD : 180
P24825.1 : WGQPKSRITHLVFCTTSGVDMPGADYQLTKALGLRPSVNRLMMYQQGCFAGGTVLRVAKD : 180
Q2R3A1.1 : WGQPRSRITHLVFCTTSGVDMPGADYQLAKMLGLRPNVNRLMMYQQGCFAGGTVLRVAKD : 179
A2ZEX7.1 : WGQPRSRITHLVFCTTSGVDMPGADYQLAKMLGLRPNVSRLMMYQQGCFAGGTVLRVAKD : 179
ScCHS1 : WGQPKSKITHLVFCTTSGVDMPGADYQLTKMLGLRPSVNRLMMYQQGCFAGGTVLRVAKD : 180
 WGQP4S4ITHLVFCTTSGVDMPGADYQLtKmLGLRPsVnRLMMYQQGCFAGGTVLRVAKD

 * 200 * 220 * 240
Q9XGX2.1 : LAENNRGARVLVVCSEITAVTFRGPSESHLDSMVGQALFGDGAAAVIVGADPDERVERPL : 240
Q9SBL6.1 : LAENNRGARVLVVCSEITAVTFRGPSESHLDSMVGQALFGDGAAAVIVGADPDERVERPL : 240
Q9SBL3.1 : LAENNRGARVLVVCSEITAVTFRGPSESHLDSMVGQALFGDGAAAVIVGADPDERVERPL : 240
Q9SBL7.1 : LAENNRGARVLVVCSEITAVTFRGPSESHLDSMVGQALFGDGAAAVIVGADPDERVERPL : 240
Q9SBL4.1 : LAENNRGARVLVVCSEITAVTFRGPSESHLDSMVGQALFGDGAAAVIVGADPDERVERPL : 240
Q9SBL5.1 : LAENNRGARVLVVCSEITAVTFRGPSESHLDSMVGQALFGDGAAAVIVGADPDERVERPL : 240
Q9XGX1.1 : LAENNRGARVLVVCSEITAVTFRGPSESHLDSMVGQALFGDGAAAVIVGADPDKRVECPL : 240
P24825.1 : LAENNRGARVLVVCSEITAVTFRGPSESHLDSLVGQALFGDGAAAVVVGADPDDRVERPL : 240
Q2R3A1.1 : LAENNRGARVLAVCSEITAVTFRGPSESHLDSMVGQALFGDGAAAVIVGSDPDEAVERPL : 239
A2ZEX7.1 : LAENNRGARVLAVCSEITAVTFRGPSESHLDSMVGQALFGDGAAAVIVGSDPDEAVERPL : 239
ScCHS1 : LAENNRGARVLVVCSEITAVTFRGPSESHLDSMVGQALFGDGAAAVIVGADPDERVERPL : 240
 LAENNRGARVLvVCSEITAVTFRGPSESHLDS6VGQALFGDGAAAV6VGaDPDerVErPL

 * 260 * 280 * 300
Q9XGX2.1 : FQLVSASQRILPDSEGAIDGHLREVGLTFHLLKDVPGLISKNIERALEEAFKPLGITDYN : 300
Q9SBL6.1 : FQLVSASQTILPDSEGAIDGHLREVGLTFHLLKDVPGLISKNIERSLEEAFKPLGITDYN : 300
Q9SBL3.1 : FQLVSASQTILPDSEGAIDGHLREVGLTFHLLKDVPGLISKNIERSLEEAFKPLGITDYN : 300
Q9SBL7.1 : FQLVSASQTILPDSEGAIDGHLREVGLTFHLLKDVPGLISKNIERSLEEAFKPLGITDYN : 300
Q9SBL4.1 : FQLVSASQTILPDSEGAIDGHLREVGLTFHLLKDVPGLISKNIERSLEEAFKPLGITDYN : 300
Q9SBL5.1 : FQLVSASQTILPDSEGAIDGHLREVGLTFHLLKDVPGLISKNIERSLEEAFKPLGITDYN : 300
Q9XGX1.1 : FQLVSASQTILPDSEGAIDGHLREVGLTFHLLKDVPGLISKNIERSLEEAFKPLGITDYN : 300
P24825.1 : FQLVSAAQTILPDSEGAIDGHLREVGLTFHLLKDVPGLISKNIGRALDDAFKPLGISDWN : 300
Q2R3A1.1 : FQMVSASQTILPDSEGAIDGHLREVGLTFHLLKDVPGLISKNIERALGDAFTPLGISDWN : 299
A2ZEX7.1 : FQMVSASQTILPDSEGAIDGHLREVGLTFHLLKDVPGLISKNIERALGDAFTPLGISDWN : 299
ScCHS1 : FQLVSAAQAILPDSEGAIDGHLREVGLTFHLLKDVPGLISKNIERALEEAFKPLGITDYN : 300
 FQ6VSAsQtILPDSEGAIDGHLREVGLTFHLLKDVPGLISKNIeR L AFkPLGI3D5N

 * 320 * 340 * 360
Q9XGX2.1 : SIFWVAHPGGPAILDQVEAKVGLEKERMRATRHVLSEYGNMSSACVLFILDEMRKRSAED : 360
Q9SBL6.1 : SIFWVAHPGGPAILDQVEAKVGLEKERLRATRHVLSEYGNMSSACVLFILDEMRKRSAED : 360
Q9SBL3.1 : SIFWVAHPGGPAILDQVEAKVGLKKERMRATRHVLSEYGNMSSACVLFILDEMRKRSAED : 360
Q9SBL7.1 : SIFWVAHPGGPAILDQVEAKVGLEKERMRATRHVLSEYGNMSSACVLFILDEMRKRSAED : 360
Q9SBL4.1 : SIFWVAHPGGPAILDQVEAKVGLEKERMRATRHVLSEYGNMSSACVLFILDEMRKRSAED : 360
Q9SBL5.1 : SIFWVAHPGGPAILDQVEAKVGLKKERMRATRHVLSEYGNMSSACVLFILDEMRKRSAED : 360
Q9XGX1.1 : SIFWVAHPGGPAILDQVEAKVGLKKERMRATRHVLSEYGNMSSACVLFILDEMRKRSAEE : 360
P24825.1 : SIFWVAHPGGPAILDQVEAKVGLDKARMRATRHVLSEYGNMSSACVLFILDEMRKRSAED : 360
Q2R3A1.1 : SIFWVAHPGGPAILDQVEAKVGLDKERMRATRHVLSEYGNMSSACVLFILDEMRKRSAED : 359
A2ZEX7.1 : SIFWVAHPGGPAILDQVEAKVGLDKERMRATRHVLSEYGNMSSACVLFILDEMRKRSAED : 359
ScCHS1 : SIFWVAHPGGPAILDQVEAKVGLDKERMRATRHVLSEYGNMSSACVLFILDEMRKRSAED : 360
 SIFWVAHPGGPAILDQVEAKVGL KeR6RATRHVLSEYGNMSSACVLFILDEMRKRSAEd

 * 380 * 400
Q9XGX2.1 : GQTTTGEGFDWGVLFGFGPGLTVETVVLHSVPITTG-AAITA : 401
Q9SBL6.1 : GQATTGEGFDWGVLFGFGPGLTVETVVLHSVPITTG-AAITA : 401
Q9SBL3.1 : GQATTGEGFDWGVLFGFGPGLTVETVVLHSVPITTG-ATITA : 401
Q9SBL7.1 : GRATTGEGFEWGVLFGFGPGLTVETVVLHSVPITTG-AAITA : 401
Q9SBL4.1 : GQATTGEGFDWGVLFGFGPGLTVETVVLHSVPITTG-AAITA : 401
Q9SBL5.1 : GQATTGEGLDWGVLFGFGPGLTVETVVLHSVPITTG-AAITA : 401
Q9XGX1.1 : GQATTGEGFDWGVLFGFGPGLTVETVVLHSVPITI--AAITA : 400
P24825.1 : GQATTGEGLDWGVLFGFGPGLTVETVVLHSVPITTG--AATA : 400
Q2R3A1.1 : GHATTGEGMDWGVLFGFGPGLTVETVVLHSVPITAG-AAA-- : 398
A2ZEX7.1 : GHATTGEGMDWGVLFGFGPGLTVETVVLHSVPITAG-AAA-- : 398
ScCHS1 : GQATTGEGFDWGVLFGFGPGLTVETVVLHSVPITTGAAAITA : 402
 G aTTGEG dWGVLFGFGPGLTVETVVLHSVPIT g aa ta

### (B). Molecular Phylogenetic analysis by Maximum Likelihood method (ScCHS1)


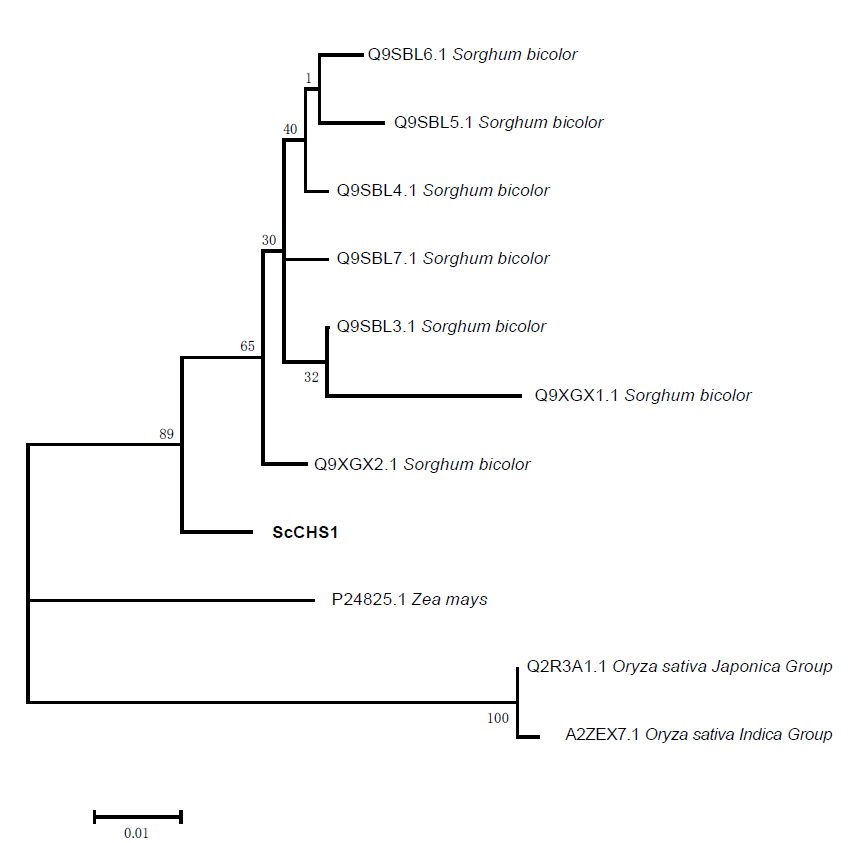


### (C). Multiple sequence alignment of ScCHS2 gene.

* 20 * 40 * 60
O23731.1 : ---------MAPAMEEIRQAQRAEGPAAVLAIGTSTPPNALYQADYPDYYFRITKSEHLT : 51
O23729.1 : ---------MAPAMEEIRQAQRAEGPAAVLAIGTSTPPNALYQADYPDYYFRITKSEHLT : 51
O23730.1 : ---------MAPAMEEIRQAQRAEGPAAVLAIGTSTPPNALYQADYPDYYFRITKSEHLT : 51
Q8LIL0.2 : MAPTTTMGSALYPLGEMRRSQRADGLAAVLAIGTANPPNCVTQEEFPDFYFRVTNSDHLT : 60
Q8RVK9.1 : ----------MVTVEEFRKAQRAEGPATIMAIGTATPANCVLQSEYPDYYFRITNSEHKT : 50
Q9FUB7.1 : ----------MVTVEEVRKAQRAEGPATVMAIGTAVPPNCVDQATYPDYYFRITNSEHKA : 50
P51090.1 : ----------MVSVAEIRKAQRAEGPATVLAIGTATPANCVYQADYPDYYFRITNSEHMT : 50
Q9ZRR8.1 : ----------MVTVEEVRKAQRAEGPATVLAIGTATPPNCLDQSTYPDYYFRITNSEHKT : 50
P08894.1 : ----------MVTVEEYRKAQRAEGPATVMAIGTATPTNCVDQSTYPDYYFRITNSEHKT : 50
Q43163.1 : ----------MVTVEEYRKAQRAEGPATILAIGTSTPSNCVDQSTYPDYYFRITNSEHKT : 50
ScCHS2 : ------MGSAPVNVCQFRRTQQAEGPAAVLAIGTANPPHSVPQDEFPDYFFRISKSEHLT : 54
 6 2 R aQrAeGpA 66AIGT P n 6 Q 5PD55FR63 SeH t

 * 80 * 100 * 120
O23731.1 : ELKEKFKRMCDKSMIKKRYMYLTEEILKENPNICAFMAPSLDARQDIVVTEVPKLAKEAA : 111
O23729.1 : ELKEKFKRMCDKSMIKKRYMYLTEEILKENPNICAFMAPSLDARQDIVVTEVPKLAKEAA : 111
O23730.1 : ELKEKFKRMCDKSMIKKRYMYLTEEILKENPNICAFMAPSLDARQDIVVTEVPKLAKEAA : 111
Q8LIL0.2 : ALKDKFKRICQEMGVQRRYLHHTEEMLSAHPEFVDRDAPSLDARLDIAADAVPELAAEAA : 120
Q8RVK9.1 : ELKEKFKRMCDKSMIRKRYMHLTEEILKENPNLCAYEAPSLDARQDMVVVEVPKLGKEAA : 110
Q9FUB7.1 : ELKEKFQRMCDKSQIKKRYMYLNEEVLKENPNMCAYMAPSLDARQDIVVVEVPKLGKEAA : 110
P51090.1 : ELKEKFKRMCEKSMINKRYMHLTEEILKENPNVCAYMAPSLDARQDMVVVEVPKLGKEAA : 110
Q9ZRR8.1 : ELKEKFQRMCDKSMIKKRYMYLTEEILKEHPNMCAYMAPSLDARQDMVVVEIPKLGKEAA : 110
P08894.1 : DLKEKFKRMCEKSMIKKRYMHLTEEILKENPSMCEYMAPSLDARQDIVVVEVPKLGKEAA : 110
Q43163.1 : ELKEKFKRMCDKSMIKKRYMHLTEEILKENPNMCAYMAPSLDARQDIVVVEVPKLGKEAA : 110
ScCHS2 : DLKVKLKRICEKSGIKKRFFHINEEILGAHPDFNDREKPSLEARIEMTATEVPKLAACAA : 114
 LKeKfkR6C ks 6 4R5 tEE6Lke P c aPSLdARqd6vv e6PkL keAA

 * 140 * 160 * 180
O23731.1 : ARAIKEWGHPKSRITHLIFCTTSGIDMPGADYQLTRLLGLRPSVNRFMLYQQGCFAGGTV : 171
O23729.1 : VRAIKEWGHPKSRITHLIFCTTSGIDMPGADYQLTRLLGLRPSVNRFMLYQQGCFAGGTV : 171
O23730.1 : VRAIKEWGHPKSRITHLIFCTTSGIDMPGADYQLTRLLGLRPSVNRFMLYQQGCFAGGTV : 171
Q8LIL0.2 : KKAIAEWGRPAADITHLVVTTNSGAHVPGVDFRLVPLLGLRPSVRRTMLHLNGCFAGCAA : 180
Q8RVK9.1 : TKAIKEWGQPKSKITHLVFCTTSGVDMPGADYQLTKLLGLRPSVKRLMMYQQGCFAGGTV : 170
Q9FUB7.1 : VKAIKEWGQPKSKITHLVFCTTSGVDMPGADYQLTKLLGLRPSVKRLMMYQQGCFAGGTV : 170
P51090.1 : AKAIKEWGQPKSKITHLVFCTTSGVDMPGADYQLTKLLGLKPSVKRLMMYQQGCFAGGTV : 170
Q9ZRR8.1 : VKAIKEWGQPKSKITHLVFCTTSGVDMPGADYQLTKLLGLRPSVKRLMMYQQGCFAGGTV : 170
P08894.1 : QKAIKEWGQPKSKITHLVFCTTSGVDMPGCDYQLTKLLGLRPSVKRLMMYQQGCFAGGTV : 170
Q43163.1 : QKAIKEWGQPKSKITHLVFCTTSGVDMPGCDYQLAKLLGLRPSVKRLMMYQQGCFAGGTV : 170
ScCHS2 : ANAIAEWGRPATDITHIVFSTYSGARTPSADLRLASLLGLRPSVCRTTLSLHGCSGGVRA : 174
 AIkEWG Pk ITH66fcTtSG d Pg D qL LLGL4PSV R m6yqqGCfaGgtv

 * 200 * 220 * 240
O23731.1 : LRLAKDLAENNAGARVLVVCSEITAVTFRGPSESHLDSLVGQALFGDGAAAIIVGSDPDS : 231
O23729.1 : LRLAKDLAENNAGARVLVVCSEITAVTFRGPSESHLDSLVGQALFGDGAAAIIVGSDPDS : 231
O23730.1 : LRLAKDLAENNAGARVLVVCSEITAVTFRGPSESHLDSLVGQALFGDGAAAIIVGSDPDS : 231
Q8LIL0.2 : LRLAKDLAENSRGARVLVVAAELTLMYFTGPDEGCFRTLLVQGLFGDGAAAVIVGAD-AD : 239
Q8RVK9.1 : LRLAKDLAENNKGARVLVVCSEITAVTFRGPNDTHLDSLVGQALFGDGSAALIVGSDPIP : 230
Q9FUB7.1 : LRLAKDLAENNKGARVLVVCSEITAVTFRGPTDTHLDSLVGQALFGDGAAAIIIGSDPIP : 230
P51090.1 : LRLAKDLAENNAGSRVLVVCSEITAVTFRGPSDTHLDSLVGQALFGDGAAAVIIGADPDT : 230
Q9ZRR8.1 : LRLAKDLAENNRGARVLVVCSEITAVTFRGPSDTHLDSLVGQALFGDGAAAIIVGADPLP : 230
P08894.1 : LRLAKDLAENNKGARVLVVCSEITAVTFRGPNDTHLDSLVGQALFGDGAGAIIIGSDPIP : 230
Q43163.1 : LRLAKDLAENNKGARVLVVCSEITAVTFRGPSESHLDSLVGQALFGDGAAAIIMGSDPII : 230
ScCHS2 : LHLAKDIAENNHGARVLVVCAEISLISYRGPTDGCVDSVLGPGLFGDGAGAVILGAGPVA : 234
 LrLAKD6AENn GaRVLVVcsE63a6 5rGP h d366gqaLFGDGaaA6I6G dp

 * 260 * 280 * 300
O23731.1 : ATERPLFQLVSASQTILPESEGAIDGHLREIGLTFHLLKDVPGLISKNIQKCLLDAFK-P : 290
O23729.1 : ATERPLFQLVSASQTILPESEGAIDGHLREIGLTFHLLKDVPGLISKNIQKCLLDAFK-P : 290
O23730.1 : ATERPLFQLVSASQTILPESEGAIDGHLREIGLTFHLLKDVPGLISKNIQKCLLDAFK-P : 290
Q8LIL0.2 : DVERPLFEIVSAAQTIIPESDHALNMRFTERRLDGVLGRQVPGLIGDNVERCLLDMFGPL : 299
Q8RVK9.1 : EVEKPIFELVSAAQTILPDSDGAIDGHLREVGLTFHLLKDVPGLISKNIEKSLNEAFK-P : 289
Q9FUB7.1 : EVEKPLFELVSAAQTILPDSEGAIDGHLREVGLTFHLLKDVPGLISKNVEKSLTEAFK-P : 289
P51090.1 : KIELPLFELVSAAQTILPDSEGAIDGHLREVGLTFHLLKDVPGLISKNIEKSLVEAFT-P : 289
Q9ZRR8.1 : EVEKPLFEVVSTAQTILPDSDGAIDGHLREVGVTFHLLKDVPGLISKNIEKSLVEAFQ-P : 289
P08894.1 : GVERPLFELVSAAQTLLPDSHGAIDGHLREVGLTFHLLKDVPGLISKNIEKSLEEAFK-P : 289
Q43163.1 : GVERPLFELVSAAQTLVPDSEGAIDGHLREVGLTFHLLKDVPGLISKNIEKSLLEAFQ-P : 289
ScCHS2 : GTERPLFETLCATQTTIPMTEDAITTQFARGGMDYHIGKQVPTIVEQTIKQCLLDATG-S : 293
 E P6F2 6sa QT 6P 3 gA6 ghlre g6t h6l4dVPg66skn6 L af p

 * 320 * 340 * 360
O23731.1 : LG---VHDWNSIFWIAHPGGPAILDQVEIKLGLKAEKLAASRNVLAEYGNMSSACVLFIL : 347
O23729.1 : LG---VHDWNSIFWIAHPGGPAILDQVEIKLGLKAEKLAASRNVLAEYGNMSSACVLFIL : 347
O23730.1 : LG---VHDWNSIFWIAHPGGPAILDQVEIKLGLKAEKLAASRSVLAEYGNMSSACVLFIL : 347
Q8LIL0.2 : LGGDGGGGWNDLFWAVHPGSSTIMDQVDAALGLEPGKLAASRRVLSDYGNMSGATVIFAL : 359
Q8RVK9.1 : LG---ISDWNSLFWIAHPGGPAILDQVESKLALKTEKLRATRHVLSEYGNMSSACVLFIL : 346
Q9FUB7.1 : LG---ISDWNSLFWIAHPGGPAILDQVEAKLSLKPEKLRATRHVLSEYGNMSSACVLFIL : 346
P51090.1 : IG---ISDWNSLFWIAHPGGPAILDQVELKLGLKEEKLRATRHVLSEYGNMSSACVLFIL : 346
Q9ZRR8.1 : LG---ISDWNSLFWIAHPGGPAILDQVEEKLALKPEKLGATRHVLSEYGNMSSACVLFIL : 346
P08894.1 : LG---ISDWNSLFWIAHPGGPAILDQVEIKLGLKPEKLKATRNVLSDYGNMSSACVLFIL : 346
Q43163.1 : LG---ISDWNSLFWIAHPGGPAILDQVELKLGLKQEKLRATREVLSNYGNMSSACVLFIL : 346
ScCHS2 : LGIDVVT-WNDLFWAVHPGGRAILDSVEAALRLETEKLAASRHVLSEYGNMSSATVVFVL : 352
 6G dWNs6FWiaHPGgpaI6DqVe kL Lk eKL A3R VL YGNMSsAcV6F L

 * 380 * 400
O23731.1 : DEMRRRSAEAGQATTGEGLEWGVLFGFGPGLTVETIVLRSVPIAGAE- : 394
O23729.1 : DEMRRRSAEAGQATTGEGLEWGVLFGFGPGLTVETIVLRSVPIAGAE- : 394
O23730.1 : DEMRRRSAEAGQATTGEGLEWGVLFGFGPGLTVETIVLRSVPIAGAE- : 394
Q8LIL0.2 : DELRRQRKEAAAAG--EWPELGVMMAFGPGMTVDAMLLHATSHVN--- : 402
Q8RVK9.1 : DEMRRKCVEDGLNTTGEGLEWGVLFGFGPGLTVETVVLHSVAIV---- : 390
Q9FUB7.1 : DEMRRKSKEDGLKTTGEGIEWGVLFGFGPGLTVETVVLHSVAIN---- : 390
P51090.1 : DEMRKKSIEEGKGSTGEGLEWGVLFGFGPGLTVETVVLHSVSAPAAHL : 394
Q9ZRR8.1 : DEMRRKSAEKGLKTTGEGLDWGVLFGFGPGLTVETVVLHSLTTP---- : 390
P08894.1 : DEMRKASAKEGLGTTGEGLEWGVLFGFGPGLTVETVVLHSVATP---- : 390
Q43163.1 : DEMRKASTKEGLGTTGEGLEWGVLFGFGPGLTVETVVLHSVATP---- : 390
ScCHS2 : KELHR-RLTAKDDGDEETAEWGVMIAFGPGITVEIMVLRATTNLKEN- : 398
 dE6r4 g tgEg ewGV6fgFGPG6TVet66L s

### (D). Molecular Phylogenetic analysis by Maximum Likelihood method (ScCHS2)


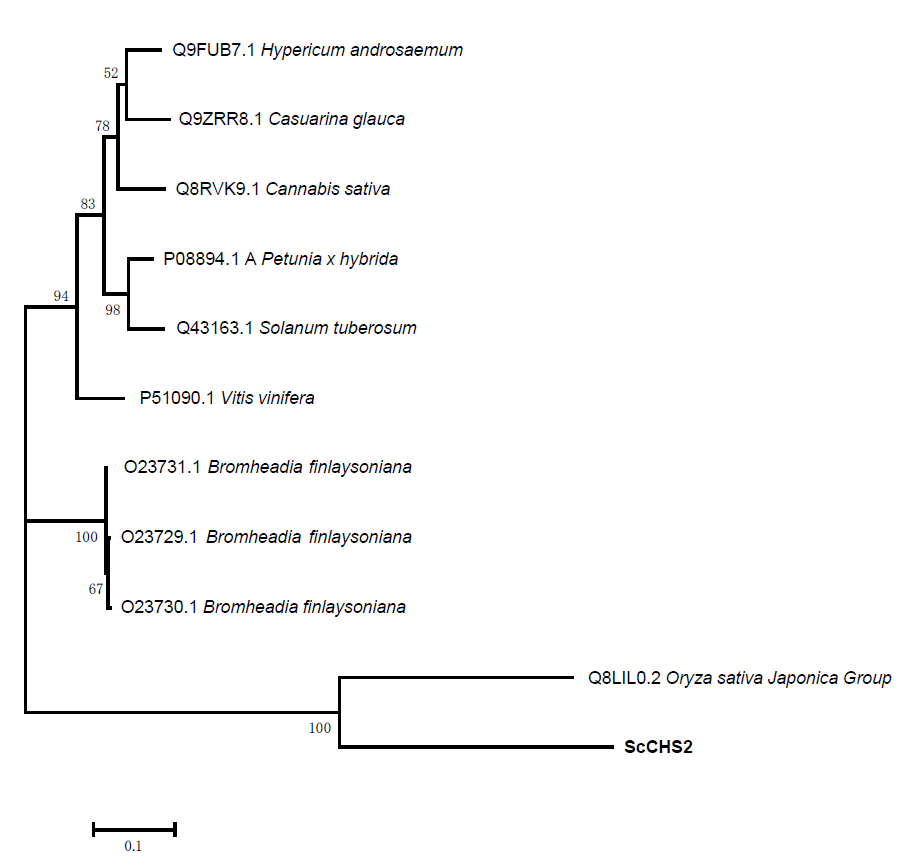


Fig S8. Sequence alignment of chalcone isomerase (CHI) proteins from sugarcane and various other plants, and phylogenetic relationships of chalcone isomerase

### Multiple sequence alignment of ScCHI1 gene.

* 20 * 40 * 60
XP_021301918.1 : ----------------------------------------------------------MG : 2
XP_034602805.1 : ----------------------------------------------------------MG : 2
XP_022684325.1 : ----------------------------------------------------------MG : 2
ACG25205.1 : ----------------------------------------------------------MG : 2
NP_001151452.1 : ----------------------------------------------------------MG : 2
RCV36830.1 : ----------------------------------------------------------MG : 2
ONM42252.1 : ---MDDCDEEQRRC-------------------------------------------AVG : 14
PWZ33927.1 : MAELFEWHRQQHRSDRSSTARVTASSSVFFLACSPAYLSVPHSRPLPYVRASNTHQVKMG : 60
XP_025827917.1 : ----------------------------------------------------------MG : 2
ScCHI1 : ----------------------------------------------------------MG : 2
 6G

 * 80 * 100 * 120
XP_021301918.1 : SET-KTITFEGIPFPAEITAAGN---PLSLLATGITDIEIHFLQIKYNAIGVYLHSNDDS : 58
XP_034602805.1 : SET-ETVTVEGIPFPAEITVG----NPLSLLANGITDIEIHFLQIKYNAIGIYLHSN--D : 55
XP_022684325.1 : SET-ETVTVEGIPFPAEITVG----NPLSLLANGITDIEIHFLQIKYNAIGIYLHNN--H : 55
ACG25205.1 : SET-ETVNVEGIPFPAEIT-VGN---PLSLLGTGITDIEIHFLQIKYNAIGVYLHNAGGG : 57
NP_001151452.1 : SET-ETVNVEGIPFPAEIT-VGN---PLSLLGTGITDIEIHFLQIKYNAIGVYLHNAGGG : 57
RCV36830.1 : SETTETVTVEGIPFPAEITVG----NPLSLLANGITDIEIHFLQIKYNAIGIYLHSN--D : 56
ONM42252.1 : SET-ETVNVEGIPFPAEIT-VGN---PLSLLGTGITDIEIHFLQIKYNAIGVYLHNAGGG : 69
PWZ33927.1 : SET-ETVNVEGIPFPAEIT-VGN---PLSLLATGITDIEIHFLQIKYNAIGVYLHNAGGG : 115
XP_025827917.1 : SET---ETVEGIPFPAEITAGSG--NPLSLLANGITDIEIHFLQIKFNAIGIYLHNN--D : 55
ScCHI1 : SET-ETITFEGIPFPAEITAAGAGGKPLSLLANGITDIEIHFLQIKYNAIGVYLHTT-DS : 60
 SET et vEGIPFPAEIT PLSLL GITDIEIHFLQIK5NAIG6YLH

 * 140 * 160 * 180
XP_021301918.1 : DLLTT----HLGAWKGKT-AEDLLADAAFWSALVSSPVEKLLRVVVIKEIKGSQYGVQLE : 113
XP_034602805.1 : ALLH-----HLQSWKGKT-ADELLGDDAFFQALVSAPVEKLFRVVVIKEIKGSQYGVQLE : 109
XP_022684325.1 : ALLH-----HLQSWKGKT-ADELLGDDAFFQALVSAPVEKLFRVVVIKEIKGSQYGVQLE : 109
ACG25205.1 : DSTT--LLGHLGAWKGKT-AEELLADAAFWAALVAAPVEKLFRVVVIKEIKGSQYGVQLE : 114
NP_001151452.1 : DSTTPTLLGHLGAWKGKT-AEELLADAAFWAALVAAPVEKLFRVVVIKEIKGSQYGVQLE : 116
RCV36830.1 : ALLH-----HLQSWKGKTTADELLGDDAFFQALVSAPVEKLFRVVVIKEIKGSQYGVQLE : 111
ONM42252.1 : DSTTPTLLGHLGAWKGKT-AEELLADAAFWAALVAAPVEKLFRVVVIKEIKGSQYGVQLE : 128
PWZ33927.1 : DSTT--LLGHLGAWKGKT-AEELLADTAFWAALVAAPVEKLFRVVVIKEIKGSQYGVQLE : 172
XP_025827917.1 : VLLG-----HLQSWKGKT-ADDLLGDDAFFQALVSAPVEKLFRVVVIKEIKGSQYGVQLE : 109
ScCHI1 : TLLG-----HLGAWKGKT-AEELLADAAFWSALVSSPVEKLLRVVVIKEIKGSQYGVQLE : 114
 HL WKGKT A eLL D AF5 ALV aPVEKLfRVVVIKEIKGSQYGVQLE

 * 200 * 220 * 240
XP_021301918.1 : SSVRDRLAAVDLYEDDEEEALEKVAEFFQAKYFKPGSVITFHFP----ATP--GP-ADIT : 166
XP_034602805.1 : SSVRDRLVAADKYDDDEEEVLEKVAEFFQSKYFKPSSVITFHFP----ATP---GAAEIS : 162
XP_022684325.1 : SSVRDRLVAADKYDDEEEEVLEKVAEFFQSKYFKPSSVITFHFP----ATP---GAAEIS : 162
ACG25205.1 : SSVRDRLAAADLYEDDEEEALEKVADFFQSKYFKPGSVVTFHFPAPASASP--GPAAEIT : 172
NP_001151452.1 : SSVRDRLAAADLYEDDEEEALEKVADFFQSKYFKPGSVVTFHFPAPASASP--GPAAEIT : 174
RCV36830.1 : SSVRDRLVAADKYDDDEEEVLEKVAEFFQSKYFKPGSVITFHFP----ATP---GAAEIS : 164
ONM42252.1 : SSVRDRLAAADLYEDDEEEALEKVADFFQSKYFKPGSVVTFHFPAPASASP--GPAAEIT : 186
PWZ33927.1 : SSVRDRLAAADLYEDEEEEALEKVADFFQSKYFKPGSVVTFHFPAPASASP--GPAAEIT : 230
XP_025827917.1 : SSVRDRLVAADRYDDDDEEALEKVADFFQAKYFKPASVITFHFP----ATPPAGAAAEIS : 165
ScCHI1 : SSVRDRLAAADLYEDEEEEALEKVAEFFQSKYFKPGSVVTFHFP----ATP--GP-AEIT : 167
 SSVRDRL AaD Y D eEE LEKVA FFQsKYFKP SV6TFHFP A3P aAeI3

 * 260 * 280 *
XP_021301918.1 : FVTEGKADAKITVENEHVAGMIQKWYLGGDNAVSPTTVRSLADRFAALLAA--- : 217
XP_034602805.1 : FATEGKNEAKMRVDNENVAAMIQKWYLGGESAASPTTVRSLADRFAALLSAAAA : 216
XP_022684325.1 : FATEGKDEAKMRVDNENVAAMIQKWYLGGESAASPTTVQSLADRFAALLSAAAA : 216
ACG25205.1 : FATEGKGDARIAVENGNVAGMIQTWYLGGDSAVSPSTVRSLADRFAALLAVAA- : 225
NP_001151452.1 : FATEGKGDARIAVENGNVAGMIQTWYLGGDSAVSPSTVRSLANRFAALLAVAA- : 227
RCV36830.1 : FATEGKDEAKMRVDNDNVAAMIQKWYLGGDSAASPTTVRSLADRFAALLSAAN- : 217
ONM42252.1 : FATEGKGDARIAVENGNVAGMIQTWYLGGDSAVSPSTVRSLANRFAALLAVAA- : 239
PWZ33927.1 : FATEGKGDARIAVENGNVAGMIQTWYLGGDSAVSPSTVRSLADRFAALLAVAA- : 283
XP_025827917.1 : FATEGKDEAKMRVENENVAGMIQKWYLGGESAVSPTTVRSLADRFAALLSAAP- : 218
ScCHI1 : FVTEGKGDAKITVENEHVAGMIQKWYLGGENAVSPTTVRSLADRFAALLAVAA- : 220
 FaTEGK A46 V N nVA MIQ WYLGG sA SP3TVrSLA1RFAALL a

### Molecular Phylogenetic analysis by Maximum Likelihood method (ScCHI1)


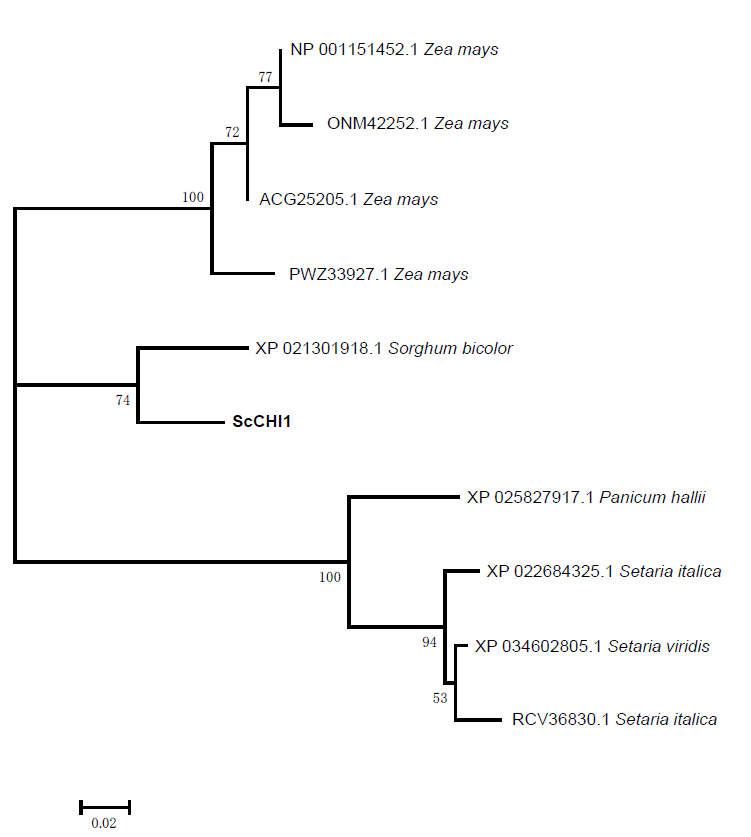


### Multiple sequence alignment of ScCHI2 gene.

* 20 * 40 * 60
Q08704.1 : ------MACRRWWSTAVVFPPVARPPGSAGSHFLGGAGVRGVEIGGNFIKFTAIGVYLED : 54
A2XNF0.1 : -----MAAVSEVEVDGVVFPPVARPPGSGHAHFLAGAGVRGVEIAGNFIKFTAIGVYLEE : 55
Q8S3X0.1 : ------MAVSELEVDGVVFPPLARPPGSAHAHFLAGAGVRGMEIGGHFIKFTAIGVYLQA : 54
Q45QI7.2 : MSPSQSPSVAQVQIESHVFPPTVKPPGTSKPFFLGGAGERGLEIQGKFIKFTAIGVYLED : 60
Q6QHK0.1 : -----MEAVTKLDVEGTAFDSVIIPPGSSKTHFLGGAGVRGLEIGGKFIAFTAIGIYLET : 55
A5HBK6.1 : MAP--PPSLAGLQVEATAFPPSVKPPGSSNTLFLGGAGVRGLEIQGNFVKFTAIGVYLED : 58
P51117.1 : MSQ--VPSVTAVQVENVLFPPSVKPPGSTNDLFLGGAGVRGLEIQGKFVKFTAIGVYLEN : 58
A5ANT9.1 : MSP--VPSVTAVQVENVLFPPSVKPPGSTNDLFLGGAGVRGLEIQGKFVKFTAIGVYLES : 58
A2IBF8.1 : MST--SLSVTELQVENFTFPPTVKPPGSTKTLFLGGAGERGLEIQGKFIKFTAIGVYLED : 58
O65333.1 : MAPF-TKSVTEVQVESVIFPPEVKPPGSSKTLFLGGAGVRGIEIQGKFIKFTAIGVYLED : 59
ScCHI2 : ------MAVPEVVVEGVVFPSVARPPGSAGSHFLGGAGVRGLEIGGNFIKFTAIGVYLED : 54
 Fpp PPG3 FLgGAGvRG6EI G F6kFTAIG6YL2

 * 80 * 100 * 120
Q08704.1 : -AAVPALAKKWGGKTADELASDAAFFRDVVTGDFEKFTRVTMILPLTGEQYAEKVTENCV : 113
A2XNF0.1 : GAAVPALAKKWAGKSADELAADAAFFRDVVTGDFEKFTRVTMILPLTGEQYSDKVTENCV : 115
Q8S3X0.1 : DAAVSALAAKWAGKPAADLASDAAFFRDVVTGEFEKFTRVTMILPLTGAQYSDKVTENCV : 114
Q45QI7.2 : -SAIPSLAVKWKGKTAEELTDSVDFFRDIVSGPFEKFTQVTMILPLTGQQYSEKVTENCV : 119
Q6QHK0.1 : -DSIPFLADKWKGKTGEELAGSLDFFRDICTGPFEKFTNVTMILPLTGEQYSEKVTENCV : 114
A5HBK6.1 : -NAVPLLAVKWKGKTAEELSESVEFFRDIVTGPFEKFIQVTTILPLTGQQYSEKVSENCV : 117
P51117.1 : -SAVPTLAVKWKGKTVEELADSVDFFRDVVTGPFEKFTKVTTILPLTGRQYSDKVSENCV : 117
A5ANT9.1 : -SAVPTLAVKWKGKTVEELADSVDFFRDVVTGPFEKFTKVTTILPLTGRQYSDKVSENCV : 117
A2IBF8.1 : -SAVNCLGVKWKGKSAVELTESVEFFRDVVTGDFEKFIRVTMILPLTGQQYSEKVSENCV : 117
O65333.1 : -NAVPSLAVKWKGKSAQELTESVEFFRDIVTGPMEKFTRVTTILPLTGQQYSEKVSENCV : 118
ScCHI2 : -AAVSALANKWAGKTADELASDAAFFRDVVKGDFEKFTRVTMIRPLTGQQYAEKVTENCV : 113
 a6 La KW GK eL FFRD6v G fEKFt VT IlPLTG QYs KV3ENCV

 * 140 * 160 * 180
Q08704.1 : AFWKAAGLYTDAEGVAVEKFREVFKPETFAPGRSILFTHSPAGVLTVAFSKDSSVPA--A : 171
A2XNF0.1 : AAWKAAGVYTDAEGAAADKFKEAFKPHSFPPGASILFTHSPPGVLTVAFSKDSSVPEGAV : 175
Q8S3X0.1 : AYWKAAGVYTDAEAAAVDKFKEAFGPHSFAPGASILFTHSPAGVLTVAFSKDSSVPE--S : 172
Q45QI7.2 : AYWKAVGTYTDAEAKAIEKFIEVFKDETFPPGGSILFTQSPLGSLTIAFSKDGSLPE--T : 177
Q6QHK0.1 : AYWKAIGIYTDAEASAVDKFKQAFKPESFPPGSSILFTHTPSGTLKIAFSKDGSVSK--D : 172
A5HBK6.1 : AFWKSVGIYTDAEGKAIEKFIEVFKDQNFPPGASILFTQSPKGSLTICFSKDASVPE--A : 175
P51117.1 : AFWKSVGIYTDAEAKAIEKFNEVLKDETFPPGNSILFTHSPLGALTMSFSKDGSLPE--V : 175
A5ANT9.1 : AFWKSVGIYTDAEAKAIEKFNEVLKDETFPPGNSILFTHSPLGALTMSFSKDGSLPE--V : 175
A2IBF8.1 : AIWKSLGIYTDAEAKAIEKFIEVFKDENFPPGSSILFTISGQGSLTIGFSKDSSVPE--G : 175
O65333.1 : AAWKSLGIYSDAEAKAIEKFIEIFKDQTFPPAASNLFTQSPLGSLTMSFSKDGSIPE--V : 176
ScCHI2 : AFWKAVGLYTDAEGVAVEKFKEVFKPETFPPGASILFTHSPTGILTVAFSKDSSVPA--A : 171
 A WK G Y3DAE A KF 2 fk FpPg SiLFT 3p G Lt6 FSKD S6p

 * 200 * 220 * 240
Q08704.1 : GGVAIENKRLCEAVLESIIGERGVSPAAKLSLAARVSELLAKETA--------------A : 217
A2XNF0.1 : AAAAIENRALCEAVLDSIIGEHGVSPAAKRSIAARVSQLLK--AE--------------S : 219
Q8S3X0.1 : GGVAIENARLCEAVLESIIGEHGVSPAAKLSLANRVAELLKG-AA--------------H : 217
Q45QI7.2 : GTVVMENKQLSEAVLESIIGKHGVSPAAKKSLAARMSELLKEKPE--------------- : 222
Q6QHK0.1 : EGVLIENKALTQAVLESIIGEHGVSPAAKLSIASRLSEIMN------------------K : 214
A5HBK6.1 : ANAVIENKLLSEAVLESILGKHGVSPAAKRSLAARLSELLNG------------------ : 217
P51117.1 : GNAVIENKLLTEAVLESIIGKHGVSPEAKKSLAARLSELFCKEAG-------------DE : 222
A5ANT9.1 : GNAVIENKLLTEAVLESIIGKHGVSPEAKKSLAARLSELFCKEAG-------------DE : 222
A2IBF8.1 : GKVVIENKLLANSVLESVIGKNGVSPAAKESLASRLSPLFNDCG---------------- : 219
O65333.1 : GNAVLENKLLSEAVLESIIGKHGVSPEAKQNLATRLVQLLNENSTTDLNESENEKLNSNE : 236
ScCHI2 : GGVAIENKPLCEAVLESIIGEHGVSPAAKLSLAARVSELLTKGTA--------------A : 217
 6EN L aVLeS66G hGVSP AK s6A R6s 6

 * 260
Q08704.1 : AADAPQAEPVSITA------ : 231
A2XNF0.1 : TGDVAAAEPAPVSA------ : 233
Q8S3X0.1 : AGGEPAAEPVPVSVS----- : 232
Q45QI7.2 : -AQTAAAAEV---------- : 231
Q6QHK0.1 : VGNVEEKLPVLSA------- : 227
A5HBK6.1 : -CKE---------------- : 220
P51117.1 : KIEAEKVAPVAC-------- : 234
A5ANT9.1 : KIEAEKVAPVAC-------- : 234
A2IBF8.1 : -ADSEKPQSK---------- : 228
O65333.1 : VSKEEKPLQVEKSAFKEVEV : 256
ScCHI2 : AADAPQVEPFSVTV------ : 231

### Molecular Phylogenetic analysis by Maximum Likelihood method (ScCHI2)


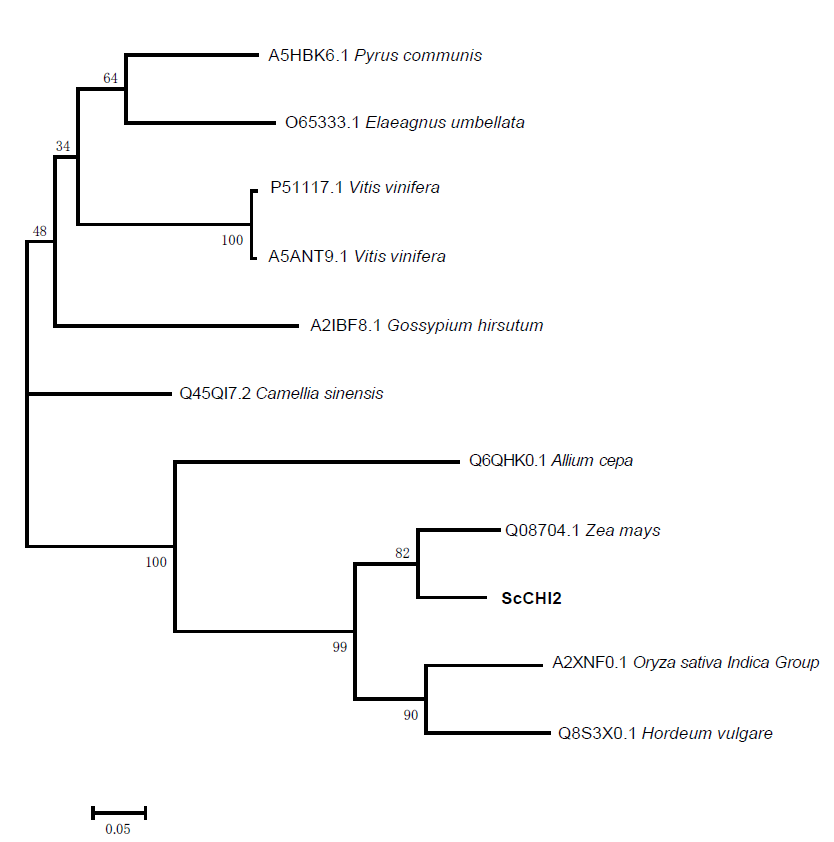


## S9 Fig. Sequence alignment of flavanone 3-hydroxylase (F3H) proteins from sugarcane and various other plants, and phylogenetic relationships of flavanone 3-hydroxylase.

### (A). Multiple sequence alignment of ScF3H gene.

* 20 * 40 * 60
P28038.1 : MAPVS--NETFLPTEAWGEATLRPSFVRDEDERPKVAHDRFSDAVPLISLHGIDGA---R : 55
A0A4D6Q4T7.1 : MAPVAT-ATPFLPTISN-ETTLRQTFVRDEDERPKVAYNVFSSEIPVISLEGIDEVDG-R : 57
Q7XM21.2 : MAPV---ATTFLPTASN-EATLRPSFVRDEDERPRVAYNQFSDAVPVISLQGIDEA---A : 53
A0A4D6Q9B0.1 : MAPGAT-ATPFLPTVSN-ETTLRESFVRDEDERPKVAYNVFSSEIPVISLEGIDEIEG-R : 57
Q07353.1 : --IPRV-TPSTLTALAE-EKTLQTSFIRDEDERPKVAYNQFSNEIPIISLEGIDDETG-K : 55
Q06942.1 : ----MA-PATTLTSIAH-EKTLQQKFVRDEDERPKVAYNDFSNEIPIISLAGIDEVEG-R : 53
Q05965.1 : -------APGTLTELAG-ESKLNSKFVRDEDERPKVAYNEFSDEIPVISLAGIDDVDG-K : 51
Q7XZQ7.1 : -----M-APSTLTALAQ-EKTLNSKFVRDEDERPKIAYNKFSDEIPVISLAGIDDDSVDK : 53
Q9S818.1 : -----M-APGTLTELAG-ESKLNSKFVRDEDERPKVAYNVFSDEIPVISLAGIDDVDG-K : 52
Q05964.1 : --MVAE-KPKTLTSLEG-DDKLNSNFVRDEDERPKVAYNEFSNDIPVISLAGIDGE---K : 53
ScF3H : MAPVSISAVPFLPTAASRESTLRASFVREEDERPKVPHDRFSDEVPVVSLQGIDGA---R : 57
 L e L F6RdEDERP46ay1 FS 6P66SL GID

 * 80 * 100 * 120
P28038.1 : RAQIRDRVAAACEDWGIFQVIDHGVDADLIADMTRLAREFFALPAEDKLRYDMSGGKKGG : 115
A0A4D6Q4T7.1 : RAEICKKIVAACEDWGVFQVVDHGVDAGLISDMTRLAREFFALPPEDKLRFDMTGGKKGG : 117
Q7XM21.2 : RAEIRARVAGACEEWGIFQVVDHGVDAGLVADMARLARDFFALPPEDKLRFDMSGGKKGG : 113
A0A4D6Q9B0.1 : RVEICKKIVAACEDWGVFQVVDHGVDAGVIADMTRLAREFFSLPPEDKLRFDMSGGKKGG : 117
Q07353.1 : RAEICDKIVKACEDWGVFQVVDHGVDAEVISQMTTFAKEFFALPPEEKLRFDMSGGKKGG : 115
Q06942.1 : RGEICKKIVAACEDWGIFQIVDHGVDAELISEMTGLAREFFALPSEEKLRFDMSGGKKGG : 113
Q05965.1 : RGEICREIVEACENWGIFQVVDHGVDTSLVADMTRLARDFFALPPEEKLRFDMSGGKKGG : 111
Q7XZQ7.1 : RSQICRKIVEACEDWGIFQVVDHGIDIDLISEMTRLARQFFALPAEEKLRFDMTGGKKGG : 113
Q9S818.1 : RGEICRQIVEACENWGIFQVVDHGVDTNLVADMTRLARDFFALPPEDKLRFDMSGGKKGG : 112
Q05964.1 : RGEICRKIVEACEDWGIFQVVDHGVGDDLIADMTRLAREFFALPAEEKLRFDMSGGKKGG : 113
ScF3H : RAEIRDRVAAACEDWGIFQVVDHGVDAALVADMARLARDFFALPAEDKLRFDMSGGKKGG : 117
 R 2I 6 ACE WG6FQ66DHG6d 66 MtrlA4 FFaLP E KLR5DM3GGKKGG

 * 140 * 160 * 180
P28038.1 : FIVSSHLQGEAVQDWREIVTYFSYPVKARDYGRWPEKPAGWCAVVERYSERLMGLSCNLM : 175
A0A4D6Q4T7.1 : FIVSSHLQGEAVQDWREIVTYFSYPIRARDYSRWPDKPEDWRSVTKTYSEKLMELACKLL : 177
Q7XM21.2 : FIVSSHLQGEAVKDWREIVTYFSYPVKSRDYSRWPDKPAGWRAVVEQYSERLMGLACKLL : 173
A0A4D6Q9B0.1 : FIVSSHLQGEAVQDWREIVTYFSYPIRARDYSRWPDKPEDWRSVTEKYSETLMELACKLL : 177
Q07353.1 : FIVSSHLQGEVVQDWREIVTYFSYPTRARDYSRWPDKPEGWIAVTQKYSEKLMELACKLL : 175
Q06942.1 : FIVSSHLQGEAVQDWREIVTYFSYPIRHRDYSRWPDKPEAWREVTKKYSDELMGLACKLL : 173
Q05965.1 : FIVSSHLQGEAVQDWREIVTYFSYPVRNRDYSRWPDKPQGWAKVTEEYSEKLMGLACKLL : 171
Q7XZQ7.1 : FIVSSHLQGEAVQDWREIVTYFSYPIQARDYSRWPDKPEGWRSITEMYSDELMALACKLL : 173
Q9S818.1 : FIVSSHLQGEAVQDWREIVTYFSYPVRNRDYSRWPDKPEGWVKVTEEYSERLMSLACKLL : 172
Q05964.1 : FIVSSHLQGEVVQDWREIVTYFSYPTNSRDYTRWPDKPEGWIKVTEEYSNKLMTLACTLL : 173
ScF3H : FIVSSHLQGEVVQDWREIVTYFSYPVKARDYSRWPDKPAAWRAVVERYSEQLMGLACKLL : 177
 FIVSSHLQGE VqDWREIVTYFSYP RDY RWPdKP W 6 YS LM LaCkL6

 * 200 * 220 * 240
P28038.1 : GVLSEAMGLETEALAKACVDMDQKVVVNFYPRCPQPDLTLGLKRHTDPGTITLLLQDLVG : 235
A0A4D6Q4T7.1 : GILSEAMGLDTEALTKACVDMDQKVVVNFYPKCPQPDLTLGLKRHTDPGTITLLLQDQVG : 237
Q7XM21.2 : GVLSEAMGLDTNALADACVDMDQKVVVNFYPKCPQPDLTLGLKRHTDPGTITLLLQDLVG : 233
A0A4D6Q9B0.1 : GVLSEAMGLDTEAITKACIDMDQKVVVNFYPKCPQPDLTLGLKRHTDPGTITLLLQDQVG : 237
Q07353.1 : DVLSEAMGLEKEALTKACVDMDQKVVVNFYPKCPEPDLTLGLKRHTDPGTITLLLQDQVG : 235
Q06942.1 : GVLSEAMGLDTEALTKACVDMDQKVVVNFYPKCPQPDLTLGLKRHTDPGTITLLLQDQVG : 233
Q05965.1 : EVLSEAMGLEKESLTNACVDMDQKIVVNYYPKCPQPDLTLGLKRHTDPGTITLLLQDQVG : 231
Q7XZQ7.1 : EVLSEAMGLEKEGLTKACVDMDQKVIVNYYPKCPQPNLTLGLKRHTDPGTITLLLQDQVG : 233
Q9S818.1 : EVLSEAMGLEKESLTNACVDMDQKIVVNYYPKCPQPDLTLGLKRHTDPGTITLLLQDQVG : 232
Q05964.1 : GVLSEAMGLELEALTKACVDMDQKIVVNYYPKCPQPDLTLGLKRHTDPGTITLLLQDQVG : 233
ScF3H : GVLSEAMGLDTDALANACVDMDQKVVVNFYPRCPQPNLTLGLKRHTDPGTITLLLQDLVG : 237
 6LSEAMGL e 6 AC6DMDQK66VN5YP4CP2P1LTLGLKRHTDPGTITLLLQD VG

 * 260 * 280 * 300
P28038.1 : GLQATRDGGKNWITVQPISGAFVVNLGDHGHFMSNGRFKNADHQAVVNGESSRLSIATFQ : 295
A0A4D6Q4T7.1 : GLQATKDGGKTWITVQPVEGAFVVNLGDHGHFLSNGRFKNADHQAVVNSNTSRLSIATFQ : 297
Q7XM21.2 : GLQATRDAGKTWITVQPIPGSFVVNLGDHAHYLSNGRFKNADHQAVVNSDCCRLSIATFQ : 293
A0A4D6Q9B0.1 : GLQATKDGGKTWITVQPVEGAFVVNLGDHGHFLSNGRFKNADHQAVVNSNTSRLSIATFQ : 297
Q07353.1 : GLQATKDNGKTWITVQPVEGAFVVNLGDHGHFLSNGRFKNADHQAVVNSNSSRLSIATFQ : 295
Q06942.1 : GLQATRDDGKTWITVQPVEGAFVVNLGDHGHLLSNGRFKNADHQAVVNSNSSRLSIATFQ : 293
Q05965.1 : GLQATRDDGNTWITVQPVEGAFVVNLGDHGHFLSNGRFKNADHQAVVNSNSSRLSIATFQ : 291
Q7XZQ7.1 : GLQATRDGGKTWITVQPVEGAFVVNLGDHGHYLSNGRFKNADHQAVVNSNSSRMSIATFQ : 293
Q9S818.1 : GLQATRDNGKTWITVQPVEGAFVVNLGDHGHFLSNGRFKNADHQAVVNSNSSRLSIATFQ : 292
Q05964.1 : GLQATRDGGKTWITVQPVPGAFVVNLGDHGHFLSNGRFKNADHQAVVNSECSRLSIATFQ : 293
ScF3H : GLQATRDGGRTWITVQPVEGAFVVNLGDHGHLLSNGRFKNADHQAVVNSECSRLSIATFQ : 297
 GLQAT4D G tWITVQP6 GaFVVNLGDHgH 6SNGRFKNADHQAVVNs sR6SIATFQ

 * 320 * 340 * 360
P28038.1 : NPAPDARVWPLAVREGEEPILEEPITFTEMYRRKMERDLDLAKRKKQAKDQLMQQQLQLQ : 355
A0A4D6Q4T7.1 : NPAPDAIVYPLAIREGEKPVLDEPITFAEMYRRKMSRDLELANLKKLAKTENQ---QVAE : 354
Q7XM21.2 : NPAPDAMVYPLAVRDGEEPILEEPITFAEMYRRKMARDLELAKLKKKAKEQRQLQQAALP : 353
A0A4D6Q9B0.1 : NPAPDAIVYPLAIREGEKPVLDEPITFAEMYRRKMSRDLELAKIKKLAKVENQ---EVLE : 354
Q07353.1 : NPAPEAIVYPLKIREGEKSIMDEPITFAEMYRRKMSKDLELARLKKQAKEQQLQAEVAAE : 355
Q06942.1 : NPAQEAIVYPLSVREGEKPILEAPITYTEMYKKKMSKDLELARLKKLAKEQQSQ---DLE : 350
Q05965.1 : NPAPEATVYPLKVREGEKAIMEEPITFAEMYKRKMGRDLELARLKKLAKEEHN--HKEAA : 349
Q7XZQ7.1 : NPAPNATVYPLKIREGEKAVMEEPITFAEMYKRKMSRDIEMATLKKLAKEKVLQ-DQEVE : 352
Q9S818.1 : NPAPDATVYPLKVREGEKAILEEPITFAEMYKRKMGRDLELARLKKLAKEERD--HKEVD : 350
Q05964.1 : NPSPDATVYPLAIREGENSIMEEPITFADLYRRKMAKDLEIARHKRLAKEEMP--FKELD : 351
ScF3H : NPAPDATVYPLAVREGEAPILDQPITFAEMYRRKMARDIELARLKKQAKAEKQLQKSAKE : 357
 NPap A V5PL 6ReGE 66 PIT5ae6Y44KM 4D6e6A K4 AK

 * 380 *
P28038.1 : QQQA--------VAAAPMPTATKPLNEILA : 377
A0A4D6Q4T7.1 : KAEV------------EAVVQPKGLSEILA : 372
Q7XM21.2 : PPPP------TQVAAELAAQKPKSLDEILA : 377
A0A4D6Q9B0.1 : KTKV------------EAVAQPKGLGEILA : 372
Q07353.1 : KAKL------------ESK--P--IEEILA : 369
Q06942.1 : KAKV------------DTK--P--VDDIFA : 364
Q05965.1 : KP----------------------LDQILA : 357
Q7XZQ7.1 : KAKL------------QMTPKS--ADEIFA : 368
Q9S818.1 : KP----------------------VDQIFA : 358
Q05964.1 : EAKF------------ESK--S--IDQILA : 365
ScF3H : FAAPNVKEFTVPTAKEFAVPNAKPLDDILA : 387
 I A

### (B). Molecular Phylogenetic analysis by Maximum Likelihood method (ScF3H)


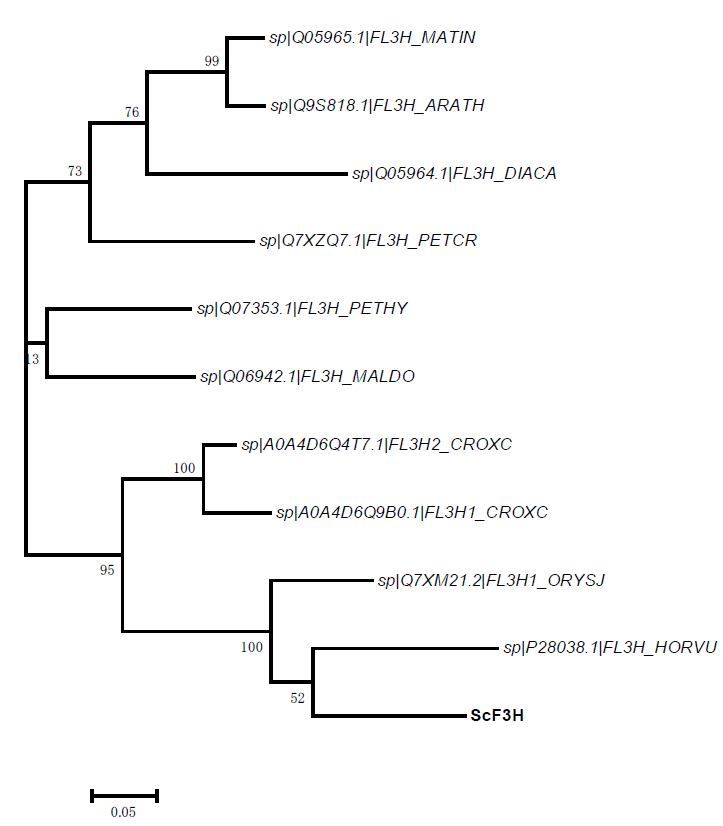


## S10 Fig. Sequence alignment of Leucoanthocyanidin dioxygenase (LDOX) proteins from sugarcane and various other plants, and phylogenetic relationships.

### Multiple sequence alignment of ScLDOX gene.

* 20 * 40 * 60
Q93VC3.1 : -MTD----VELRVEALSLSGVSAIPPEYVRPEEERAD-LGDALELARAASDDDATARIPV : 54
A2WQ39.1 : -MTD----VELRVEALSLSGVSAIPPEYVRPEEERAD-LGDALELARAASDDDATARIPV : 54
Q67VR7.1 : -MTD----VELRVEALSLSDVSAIPPEYVRLEEERTD-LGDALEVARAASDDADAARIPV : 54
P51091.1 : -MVSSDS-VNSRVETLAGSGISTIPKEYIRPKDELVN-IGDIFEQEKNN----EGPQVPT : 53
Q96323.1 : -MVAVE-----RVESLAKSGIISIPKEYIRPKEELES-INDVFLEEKKE----DGPQVPT : 49
O04274.1 : -MVTSAMGPSPRVEELARSGLDTIPKDYVRPEEELKSIIGNILAEEKSS----EGPQLPT : 55
P51092.1 : -MVNAVVTTPSRVESLAKSGIQAIPKEYVRPQEELNG-IGNIFEEEKKD----EGPQVPT : 54
A0A2R6PI27.1 : MVATVVG---TRVESLASSGIEAIPKEYVRPQEELTS-IGNIFEEEKKE----NGPQVPT : 52
P51093.1 : -MVTSVA---PRVESLSSSGIQSIPKEYIRPQEELTS-IGNVFEEEKKD----EGPQVPT : 51
ScLDOX : -MASPSPLLQLPARVEALSGLSAIPLEYVRPADERAG-LGDAFDLARTHANDHAAPRIPV : 58
 6 rve l Sg6 IP eY6Rp eE 6g1 4 6P

 * 80 * 100 * 120
Q93VC3.1 : VDISAFDND-----GDGRHACVEAVRAAAEEWGVIHIAGHGLPGDVLGRLRAAGEAFFAL : 109
A2WQ39.1 : VDISAFDND-----GDGRHACVEAVRAAAEEWGVIHIAGHGLPGDVLGRLRAAGEAFFAL : 109
Q67VR7.1 : VDISAFD-------GDGRRACVEAVRAAAEEWGVMHIAGHGLPGDVLDRLRAAGEAFFAL : 107
P51091.1 : IDLKEIESDNE----KVRAKCREKLKKAAVDWGVMHLVNHGISDELMDKVRKAGKAFFDL : 109
Q96323.1 : IDLKNIESDDE----KIRENCIEELKKASLDWGVMHLINHGIPADLMERVKKAGEEFFSL : 105
O04274.1 : IDLEEMDSRDE----EGRKKCHEELKKAATDWGVMHLINHGIPEELIDRVKAAGKEFFEL : 111
P51092.1 : IDLKEIDSEDK----EIREKCH-QLKKAAMEWGVMHLVNHGISDELINRVKVAGETFFDQ : 109
A0A2R6PI27.1 : IDLEDLVSEDE----EKRVRCHEELKRAATEWGVMQVVNHGIPIELMERVRAAGAEFFNQ : 108
P51093.1 : IDLKDIESEDEVVRREIRERCREELKKAAMEWGVMHLVNHGISDDLINRVKVAGETFFNL : 111
ScLDOX : VDISPFLDATAS-SQQQRDACVEAVRAAAADWGVMHIAGHGIPAELMDRLRAAGAAFFAL : 117
 6D6 R C e 64 Aa WGV6h6 HG6 66 464 AG FF l

 * 140 * 160 * 180
Q93VC3.1 : PIAEKEAYANDPAAGRLQGYGSKLAANASGKREWEDYLFHLVHPDHLADHSLWPANPPEY : 169
A2WQ39.1 : PIAEKEAYANDPAAGRLQGYGSKLAANASGKREWEDYLFHLVHPDHLADHSLWPANPPEY : 169
Q67VR7.1 : PIAEKEAYANDPAAGRLQGYGSKLAANASGKREWEDYLFHLVHPDHLADHSLWPANPPEY : 167
P51091.1 : PIEQKEKYANDQASGKIQGYGSKLANNASGQLEWEDYFFHCVYPEDKRDLSIWPQTPADY : 169
Q96323.1 : SVEEKEKYANDQATGKIQGYGSKLANNASGQLEWEDYFFHLAYPEEKRDLSIWPKTPSDY : 165
O04274.1 : PVEEKEAYANDQAAGNVQGYGSKLANNASGQLEWEDYFFHCVYPEHKTDLSIWPTKPPDY : 171
P51092.1 : PVEEKEKYANDQANGNVQGYGSKLANSACGQLEWEDYFFHCAFPEDKRDLSIWPKNPTDY : 169
A0A2R6PI27.1 : SVEEKEKYANDHASGNIQGYGSKLANNASGQLEWEDYFFHLVYPEDKRDMSIWPKTPSDY : 168
P51093.1 : PMEEKEKYANDQASGKIAGYGSKLANNASGQLEWEDYFFHLIFPEDKRDMTIWPKTPSDY : 171
ScLDOX : PIQDKEAYANDPAAGRLQGYGSRLATNASGQREWEDYLFHLVQPDGLADHALWPAHPPDY : 177
 p6 KE YAND A G 6qGYGS4LA nAsG EWEDY FH P D 6WP P Y

 * 200 * 220 * 240
Q93VC3.1 : VPVSRDFGGRVRTLASKLLAILSLGLGLP--EETLERRLRGHELAGVDD----------- : 216
A2WQ39.1 : VPVSRDFGGRVRTLASKLLAILSLGLGLP--EETLERRLRGHELAGVDD----------- : 216
Q67VR7.1 : VPVSRDFGGRVRTLASKLLAILSLGLGLP--EETLERRLRRHDQHGVDD----------- : 214
P51091.1 : IEATAEYAKQLRELATKVLKVLSLGLG-------LDEGRLEKEVGGLEE----------- : 211
Q96323.1 : IEATSEYAKCLRLLATKVFKALSVGLG-------LEPDRLEKEVGGLEE----------- : 207
O04274.1 : IPATSEYAKQLRALATKILSVLSIGLG-------LEKGRLEKEVGGAED----------- : 213
P51092.1 : TPATSEYAKQIRALATKILTVLSIGLG-------LEEGRLEKEVGGMED----------- : 211
A0A2R6PI27.1 : IPATSAYAEHLRGLATKILSALSLGLG-------LEEGRLEKEVGGMEE----------- : 210
P51093.1 : VPATCEYSVKLRSLATKILSVLSLGLG-------LEEGRLEKEVGGMEE----------- : 213
ScLDOX : VPATRDFGRRTRELTSTLLAILSMGLLGPDRGDALEKALITHSQAEAGDEHEQKQQQQDL : 237
 p 3 5 R La3k6l LS6GLg Le e g

 * 260 * 280 * 300
Q93VC3.1 : DLLLQLKINYYPRCPRPDLAVGVEAHTDVSALSFILHNGVPGLQVHHAGSWVTARPEPGT : 276
A2WQ39.1 : DLLLQLKINYYPRCPRPDLAVGVEAHTDVSALSFILHNGVPGLQVHHAGSWVTARPEPGT : 276
Q67VR7.1 : DLLLQLKINYYPRCPRPDLAVGVEAHTDVSALSFILHNGVPGLQAHHAGTWVTARSEQGT : 274
P51091.1 : -LLLQMKINYYPKCPQPELALGVEAHTDVSALTFILHNMVPGLQLFYEGKWVTAKCVPNS : 270
Q96323.1 : -LLLQMKINYYPKCPQPELALGVEAHTDVSALTFILHNMVPGLQLFYEGKWVTAKCVPDS : 266
O04274.1 : -LIVQMKINFYPKCPQPELALGWEAHTDVSALTFILHNMVPGLQLFYEDKWVTAKCVPNS : 272
P51092.1 : -LLLQMKINYYPKCPQPELALGVEAHTDVSALTFILHNMVPGLQLFYEGQWVTAKCVPNS : 270
A0A2R6PI27.1 : -LLLQMKINYYPKCPQPELALGVEAHTDVSALTFILHNMVPGLQLFYEGKWVTAKCVPDS : 269
P51093.1 : -LLLQKKINYYPKCPQPELALGVEAHTDVSALTFILHNMVPGLQLFYEGKWVTAKCVPNS : 272
ScLDOX : DLLLQFKINYYPRCPQPELAIGVEAHTDVSALSFILHNGVPGLQVLHGGRWVTARDEPGT : 297
 L66Q KIN5YP4CP P LA6GvEAHTDVSAL3FILHN VPGLQ g WVTA4 p 3

 * 320 * 340 * 360
Q93VC3.1 : IVVHVGDALEILTNGRYTSVLHRGLVSRDAVRLSWVVFCEPPPESVLLQPVPELLAD--- : 333
A2WQ39.1 : IVVHVGDALEILTNGRYTSVLHRGLVSRDAVRLSWVVFCEPPPESVLLQPVQELLAD--- : 333
Q67VR7.1 : IVVHVGDALEILTNGRYTSVLHRSLVSRDAVRVSWVVFCEPPPESVLLQPLPELLAN--- : 331
P51091.1 : IVMHIGDTLEILSNGKYKSILHRGMVNKEKVRISWAVFCEPPKEKIILKPLPET----VS : 326
Q96323.1 : IVMHIGDTLEILSNGKYKSILHRGLVNKEKVRISWAVFCEPPKDKIVLKPLPEM----VS : 322
O04274.1 : IIMHIGDTLEILSNGKYKSILHRGLVNKEKVRISWAVFCEPPKEKIVLQPLPET--V--S : 328
P51092.1 : IIMHIGDTIEILSNGKYKSILHRGVVNKEKVRFSWAIFCEPPKEKIILKPLPET--V--T : 326
A0A2R6PI27.1 : LVMHIGDTIEILSNGKYKSILHRGLVNKEKVRISWAVFCEPPKEKIILKPLPET--V--S : 325
P51093.1 : IIMHIGDTIEILSNGKYKSILHRGLVNKEKVRISWAVFCEPPKEKIILKAHCQRRCLRLS : 332
ScLDOX : IIVHVGDALEILSNGRYTSVLHRGLVNREAVRISWVVFAEPPPDAVVLRPLPELVT---- : 353
 666H6GD 6EIL3NG4Y S6LHRg6V 4 VR SW 6FcEPP e 66L p p2

 * 380 * 400 * 420
Q93VC3.1 : GADKPLFAPRTFKQHVQRKLFEKLK------------DQQDNNAAAASNGMRTK------ : 375
A2WQ39.1 : GAGKPLFAPRTFKQHVQRKLFKKLK------------DQQDNNAAAASNGMITK------ : 375
Q67VR7.1 : GAGKPLFAPRTFKQHVQRKLFKKLK------------DQQDNNAAAASNGIIPK------ : 373
P51091.1 : EDEPAMFPPRTFAEHIQHKLFRKSQE------------ALLPK----------------- : 357
Q96323.1 : VESPAKFPPRTFAQHIEHKLFGKEQE------------ELVSEKND-------------- : 356
O04274.1 : EVEPPRFPPRTFAQHLKHKLFRKTDG------------DLDEKPTY-------------- : 362
P51092.1 : EAEPPRFPPRTFAQHMAHKLFRKDDKDAAVEHKVFNEDELDTAAEHKVLKKDNQDAVAEN : 386
A0A2R6PI27.1 : EAEPPLYPPRTFAQHIHHKLFRKTQE-------------LGAK----------------- : 355
P51093.1 : HHSSHLAPFPNIFSTSSSGRPRRLYS-------------PNEL----------------- : 362
ScLDOX : ADQPARFTPRTFKQHLDNKLFKKKQQ-----------EQQKAKEEEDGNGVHHHDEPPPQ : 402
 prtf h klf 4

 * 440 * 460
Q93VC3.1 : -------------------------------------------- : -
A2WQ39.1 : -------------------------------------------- : -
Q67VR7.1 : -------------------------------------------- : -
P51091.1 : -------------------------------------------- : -
Q96323.1 : -------------------------------------------- : -
O04274.1 : -------------------------------------------- : -
P51092.1 : KDIKEDEQCGPAEHKDIKEDGQGAAAENKVFKENNQDVAAEESK : 430
A0A2R6PI27.1 : -------------------------------------------- : -
P51093.1 : -------------------------------------------- : -
ScLDOX : TQTN---------------------------------------- : 406

### (B) Molecular Phylogenetic analysis by Maximum Likelihood method (ScLDOX)


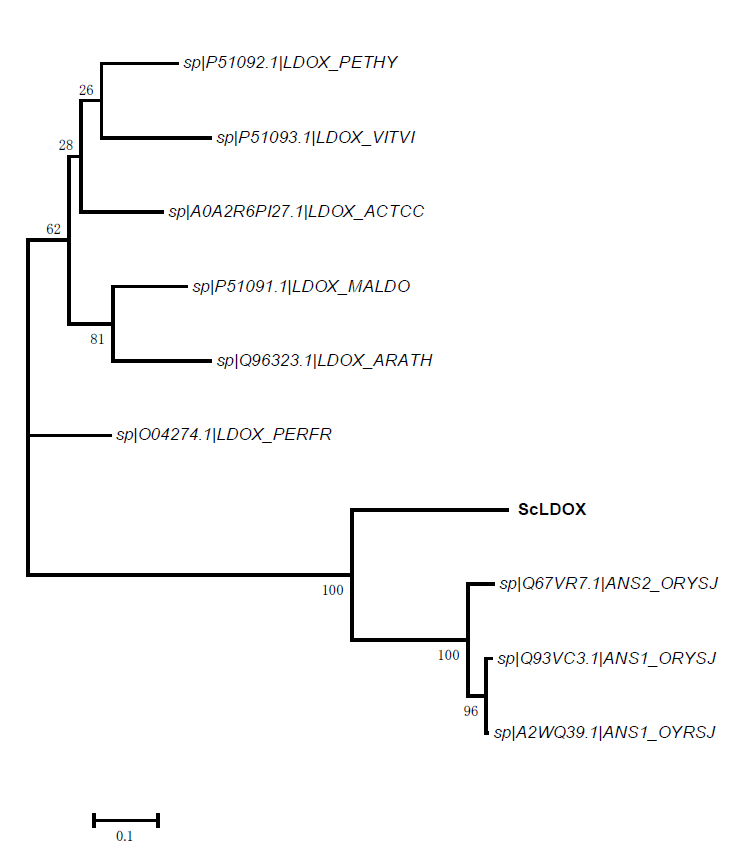


## Fig S11. Sequence alignment of BZ2 proteins from sugarcane and various other plants, and phylogenetic relationships of BZ2 from sugarcane and various other plants.

### (A). Multiple sequence alignment of ScBZ2 gene.

* 20 * 40 * 60
XP_0024672 : MDGAAAAPCCSAGCLGRLAAAESRIGDAETESIAAGGVRDEERNCGGRGGSGWESESGAL : 60
XP_0024672 : ------------------------------------------------------------ : -
AAV64226.1 : ------------------------------------------------------------ : -
AAV64188.1 : ------------------------------------------------------------ : -
CAA57496.1 : ------------------------------------------------------------ : -
NP_0011836 : ------------------------------------------------------------ : -
ONM06458.1 : ------------------------------------------------------------ : -
P50472.1_R : ------------------------------------------------------------ : -
XP_0257980 : ------------------------------------------------------------ : -
XP_0049866 : -----------------------------------------------------MAATTAT : 7
ScBZ2 : ------------------------------------------------------------ : -


 * 80 * 100 * 120
XP_0024672 : SVLGRARELLKSQEEERRGAQHVGTSRPACISSSSMTAG---TMRLLGGEVSPFAARARL : 117
XP_0024672 : -----------------------------------MTAG---TMRVLGGEVSPFTARARL : 22
AAV64226.1 : -----------------------------------MTAG---TMRVLGGEVSPFTARARL : 22
AAV64188.1 : -----------------------------------MTAG---TMRVLGGEVSPFTARARL : 22
CAA57496.1 : -----------------------------------MTAG---TMRVLGGEVSPFTARARL : 22
NP_0011836 : ---------------MPTEVTSTPASRPDCSSSSSMTAG---TMRVLGGEVSPFTARARL : 42
ONM06458.1 : ---------------MPTEVTSTPASRPDCSSSSSMTAG---TMRVLGGEVSPFTARARL : 42
P50472.1_R : -------------------------------------------MRVLGGEVSPFTARARL : 17
XP_0257980 : -----------------------------------MTAGTAPAVRVLGGEVSPFTARARL : 25
XP_0049866 : LEASARLRSTHELRHVGTCSRPACIDLVTSSPSSSMTAGRTTTVRVLGGELSPFTARARL : 67
ScBZ2 : -----------------------------------MTAG---TMRVLGGEVSPFTARARL : 22
 mtag t6R6LGGE6SPFtARARL

 * 140 * 160 * 180
XP_0024672 : ALELRGVAYELLDEPLGPKKSDRLLAANPVYGKIPVLLLPDGRAICESAVIVQYVEDVAR : 177
XP_0024672 : ALELRGVAYELLDEPLGPKKSDRLLAANPVYGKIPVLLLPDGRAICESAVIVQYVEDVVR : 82
AAV64226.1 : ALDLRGVAYELLDEPLGPKKSDRLLAANPVYGKIPVLLLPDGRAICESAVIVQYIEDVAR : 82
AAV64188.1 : ALDLRGVAYELLDEPLGPKKSDRLLAANPVYGKIPVLLLPDGRAICESAVIIQCIEDVAR : 82
CAA57496.1 : ALDLRGVAYELLDEPLGPKKSDRLLAANPVYGKIPVLLLPDGRAICESAVIVQYIEDVAR : 82
NP_0011836 : ALDLRGVAYELLDEPLGPKKSDRLLAANPVYGKIPVLLLPDGRAICESAVIIQCIEDVAR : 102
ONM06458.1 : ALDLRGVAYELLDEPLGPKKSDRLLAANPVYGKIPVLLLPDGRAICESAVIIQCIEDVAR : 102
P50472.1_R : ALDLRGVAYELLDEPLGPKKSDRLLAANPVYGKIPVLLLPDGRAICESAVIVQYIEDVAR : 77
XP_0257980 : ALELRGVAHELLEEPLGPAKSARLLAANPVYGKIPVLLLPDGRAICESAVIVQYVDDAAR : 85
XP_0049866 : ALELRGVAYELLDEPLGPRKSDRLLAANPAYGKIPVLLLPDGRAICESAVIVQYVEDVAR : 127
ScBZ2 : ALELRGVAYELLDEPLGPKKSDRLLAANPVYGKIPVLLLPDGRAICESAVIVQYVEDVAR : 82
 AL LRGVAyELLdEPLGP KSdRLLAANPvYGKIPVLLLPDGRAICESAVI6Q 6eDvaR

 * 200 * 220 * 240
XP_0024672 : GSGGA----EAGGLLLPDDPYERAMHRFWTVFIDDKFWPALDAVSLAPTADARAQAVVDT : 233
XP_0024672 : GTGGA----EAGGLLLPDDPYERAMHRFWTVFIDDKFWPALDAVSLAPTADERAQAVVDT : 138
AAV64226.1 : ESGGA----EAGSLLLPDDPYERAMHRFWTAFIDDKFWPALDAVSLAPTPGARAQAAEDT : 138
AAV64188.1 : GSGGA----EASSLLLPDDPYERAMHRFWTAFIDDKFWPALDAVSLAPTPGARAQAVEDT : 138
CAA57496.1 : ESGGA----EAGSLLLPDDPYERAMHRFWTAFIDDKFWPALDAVSLAPTPGARAQAAEDT : 138
NP_0011836 : GSGGA----EASSLLLPDDPYERAMHRFWTAFIDDKFWPALDAVSLAPTPGARAQAVEDT : 158
ONM06458.1 : GSGGA----EASSLLLPDDPYERAMHRFWTAFIDDKFWPALDAVSLAPTPGARAQAVEDT : 158
P50472.1_R : ESGGA----EAGSLLLPDDPYERAMHRFWTAFIDDKFWPALDAVSLAPTPGARAQAAEDT : 133
XP_0257980 : STASAGGEDD--CLLLPEDPYERAMHRFWTAYIDDKFWPALDAVSLGPTPAAQAQAADDT : 143
XP_0049866 : AAAGAGGEGEGAALLLPEDPYERAMHRFWTAYIDDKFWPAIDAVSLGPTPEARAQATADA : 187
ScBZ2 : GSG--------GGLLLPDDPYERAMHRFWTVFIDDKFWPALDAVSLAPTADARAQAVVDT : 134
 gga e LLLPdDPYERAMHRFWT 5IDDKFWPA6DAVSLaPT arAQA Dt

 * 260 * 280 * 300
XP_0024672 : RAALNRLEEVFKDRSNGAAFFSGRDAAPGLLDLALGCFLPALRACERLHGLSLIDAS--T : 291
XP_0024672 : RAALNRLEEVFKDRSSGAAFFSGRDAAPGLLDLALGCFLPALRACERLHGLSLIDAS--A : 196
AAV64226.1 : RAALSLLEEAFKDRSNGRAFFSGGDAAPGLLDLALGCFLPALRACERLHGLSLIDAS--A : 196
AAV64188.1 : RAALSLLEVAFKDRSNGRAFFSGGDAAPGLLDLALGCFLPALRACERLHGLSLIDAS--A : 196
CAA57496.1 : RAALSLLEEAFKDRSNGRAFFSGGDAAPGLLDLALGCFLPALRACERLHGLSLIDASATA : 198
NP_0011836 : RAALSLLEVAFKDRSNGRAFFSGGDAAPGLLDLALGCFLPALRACERLHGLSLIDAS--A : 216
ONM06458.1 : RAALSLLEVAFKDRSNGRAFFSGGDAAPGLLDLALGCFLPALRACERLHGLSLIDAS--A : 216
P50472.1_R : RAALSLLEEAFKDRSNGRAFFSGGDAAPGLLDLALGCFLPALRACERLHGLSLIDAS--A : 191
XP_0257980 : RAALRLLEEAFRDRSGGAAFFSGRDASPGLLDLALGCFLPALRACERLHGLSLVDAS--A : 201
XP_0049866 : RAALRLLEGAFKDCSSGAGFFSGSDAAPGLLDLALGCFLPALRACERLHGLSLIDAS--A : 245
ScBZ2 : RAALNRLEEAFKDRSNGAAFFSGRDAAPGLLDLALGCFLPALRACERLHGLSFIDAS--T : 192
 RAAL LE aF4DrS G aFFSG DAaPGLLDLALGCFLPALRACERLHGLSl6DAS a

 * 320 * 340
XP_0024672 : TPLLDGWSRRFAAHPAAKRVLPDTDKVVQFTRFL--QEKFGVH-VS-- : 334
XP_0024672 : TPLLDGWSRRFAAHPAAKRILPDTDKVVQFTKFL--QEMFGVH-VS-- : 239
AAV64226.1 : TPLLDGWSQRFAAHPAAKRVLPDTEKVVQFTRFL--QAQFRVH-VS-- : 239
AAV64188.1 : TPLLDGWSQRFAAHPAAKRVLPDTEKVVQFTRFL--QGQFRVH-VS-- : 239
CAA57496.1 : TPLLDGWSQRFAAHPAAKRVLPDTEKVVQFTRFL--QAQFRVH-VS-- : 241
NP_0011836 : TPLLDGWSQRFAAHPAAKRVLPDTEKVVQFTRFL--QGQFRVH-VS-- : 259
ONM06458.1 : TPLLDGWSQRFAAHPAAKRVLPDTEKVVQFTRFL--QGQFRLF-FHHV : 261
P50472.1_R : TPLLDGWSQRFAAHPAAKRVLPDTEKVVQFTRFLQVQAQFRVH-VS-- : 236
XP_0257980 : TPLLDGWSRRFAAHPAARRVLPDTEKVVRFTRFL--QEKFGADDVSTK : 247
XP_0049866 : TPLLDRWSHRFAAHPATRRVLPDTDKVVEFTRFL--QAKFGVD-VSK- : 289
ScBZ2 : TPLLDGWSRRFAAHPAAKRILPDTDKVVQFTRFL--QAKFGVR-VS-- : 235
 TPLLDgWS RFAAHPAa4R6LPDT KVV FT4FL Q F vs

### (B). Molecular Phylogenetic analysis by Maximum Likelihood method (ScBZ2)


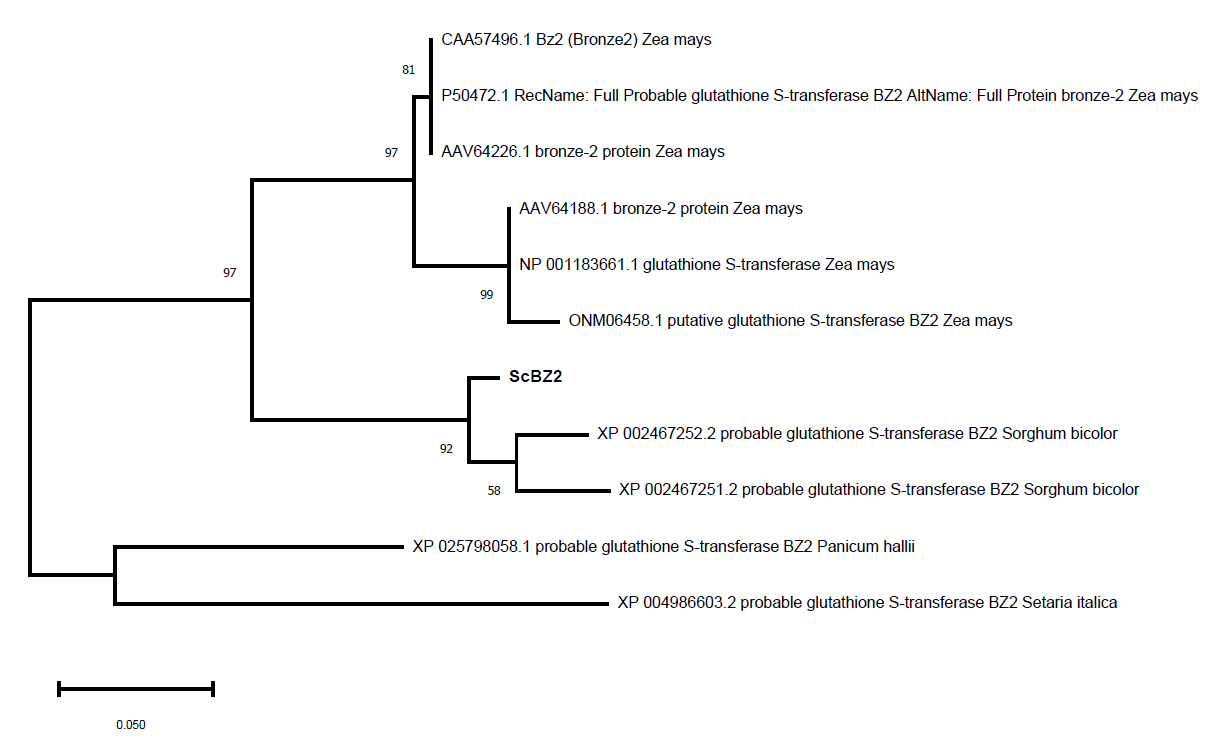


## Fig S12. Sequence alignment of MYB proteins from sugarcane and various other plants, and phylogenetic relationships of MYB from sugarcane and various other plants.

### (A). Multiple sequence alignment of MYB gene.

* 20 * 40 *
XP_0024419 : ----------MEKSSRVRP------SRGHWRPGEDDKLRQLVDKYGPQNW : 34
RLM99791.1 : ---MAP-SSTTTTSDKANSRSPSSCPRGHWRPGEDEKLRQLVEQYGPQNW : 46
XP_0049782 : ---MAPSSSTTATPVRANSRPPSSCPRGHWRPGEDDKLRQLVENYGPQNW : 47
RLN28584.1 : ---MAP-SSTTTTSDRANSRSPSSCPRGHWRPGEDEKLRQLVEQYGPQNW : 46
OEL26485.1 : MRDMVPSSSTTTTSGRANSRSSSSCSRGHWRPGEDEKLRQLVEQYGPQNW : 50
OEL14395.1 : ----MASSSTTNTSDGAKS---SSCPRGHWRPGEDEKLRQLVDKYGPQNW : 43
XP_0273439 : -----------------------MCSRGHWRPAEDDKLRELVEHYGPHNW : 27
XP_0202521 : --------MEENMEASGSEDQTKSCPRGHWRPGEDEKLRQLVEQYGPQNW : 42
XP_0239272 : -----------------------MCTRGHWRPAEDEKLRELVERYGPHNW : 27
AKE81092.1 : -----------------------MCTRGHWRPAEDEKLKELVEKYGPHNW : 27
ScMYB : ----------MEKS-RARP------SRGHWRPGEDDKLRQLVDKYGPQNW : 33
 c RGHWRP ED KL42LV YGP NW

 60 * 80 * 100
XP_0024419 : NSIAENLEGRSGKSCRLRWFNQLDPRINRRPFTAAEEELLLQAHRAHGNR : 84
RLM99791.1 : NSIAEKLDGRSGKSCRLRWFNQLDPRINRRPFTAAEEERLLQAHRAHGNR : 96
XP_0049782 : NSIAEKLEGRSGKSCRLRWFNQLDPRINRRPFTAAEEERLLQAHRAHGNR : 97
RLN28584.1 : NSIAEKLEGRSGKSCRLRWFNQLDPRINRRPFTAAEEERLLQAHRAHGNR : 96
OEL26485.1 : NSIAEKLEGRSGKSCRLRWFNQLDPRINRRPFTAAEEERLLQAHRAHGNR : 100
OEL14395.1 : NSIAEKLEGRSGKSCRLRWFNQLDPRINKRPFTEEEEERLLNAHRAHGNK : 93
XP_0273439 : NAIAEKLRGRSGKSCRLRWFNQLDPRINRSPFTEEEEERLVASHRIHGNR : 77
XP_0202521 : NSIAEKLQGRSGKSCRLRWFNQLDPRINRKPFTEEEEDRLLAAHRVHGNK : 92
XP_0239272 : NAIAEKLQGRSGKSCRLRWFNQLDPRINRSPFTEEEEERLLASHRIHGNR : 77
AKE81092.1 : NAIAEKLQGRSGKSCRLRWFNQLDPRINRSPFTEEEEERLLASHRIHGNR : 77
ScMYB : NSIAENLEGRSGKSCRLRWFNQLDPRINRRPFTAAEEELLLQAHRAHGNR : 83
 N IAEkL GRSGKSCRLRWFNQLDPRIN4 PFT EEerL6 HR HGN4

 * 120 * 140 *
XP_0024419 : WALISRFFPGRTDNAVKNHWHVVTARRRRGHHHHHRTLVGDGEQTATSTG : 134
RLM99791.1 : WALISRLFPGRTDNAVKNHCHVVMARRR---RSHQRSAG--TLLAASATG : 141
XP_0049782 : WALISRLFPGRTDNAVKNHWHVVMARRS---HHHHRSAGTLALLAG--SV : 142
RLN28584.1 : WALISRLFPGRTDNAVKNHWHVVMARRR---RSHQRSAG--TLLAGPGTG : 141
OEL26485.1 : WALISRLFPGRTDNAVKNHWHVVMARRRR-MRSHHRSAG--TFLAG--AG : 145
OEL14395.1 : WALIARLFPGRTDNAVKNHWHVVMARRSR-ERSRLLARASSSPSSAYPFG : 142
XP_0273439 : WAVIARLFPGRTDNAVKNHWHVMMARVR---RERSKLYAKHPALPRTNPH : 124
XP_0202521 : WAHIARLFPGRTDNAVKNHWHVIMARRQ---RERSRQLGKSSRSSSIYDP : 139
XP_0239272 : WAVIARLFPGRTDNAVKNHWHVIMARRC---RERSKLYAKRAAQTLMNDQ : 124
AKE81092.1 : WAIIARFFPGRTDNAVKNHWHVIMARRY---RERSRLHAKRAAQTLVNDN : 124
ScMYB : WALISRLFPGRTDNAVKNHWHVVMARRRR-RRQYHCSGSGGTLTGDPTTG : 132
 WA I RlFPGRTDNAVKNHwHV6mARr r

 160 * 180 * 200
XP_0024419 : YVAVVPPRHQPPFHHQYFHFGSCCLPATTTKMTTTRSLCFAVPAGSGPLG : 184
RLM99791.1 : ---CSPPPRRPPF--QCFHFGAA---PASAKTTTG-NLCFATP-GSGPSS : 181
XP_0049782 : ---YSPPPRRPPF--QCFHFGAP---PAAMKTTGSLSLCFATP-GSGPSS : 183
RLN28584.1 : ---FSPPPRRPPF--QCFHFSAP---PASAKTTTG-NLCFATP-GSGPSS : 181
OEL26485.1 : ---CSPPPRRPPF--QCFHFGAP---PAATKTTG--SLCFAMP-SSGPSS : 184
OEL14395.1 : --TGAPATSSLCFGFSKLGGGGRGGLFRSPAAAAPTSLFKSFGTATGSNG : 190
XP_0273439 : --HNS--------NYDTTTFAS---SFVDKCSPFQ-------------FT : 148
XP_0202521 : --TSVHQERNHISNHRMFEFRNPLKGFAPSSSSQF---------SWGFPS : 178
XP_0239272 : --KSSLKQEVQMMNCEKRITTN--ASFVEKCRERY-QYPFMYN-YCPPFP : 168
AKE81092.1 : --KLSSKQDHMHVDCETRNFS----SFAKKYCEKYGHYPMVTHSSLPAFC : 168
ScMYB : --SVVPPR-QPSF--QYFRFGSC--PPATIAKTTTHSLCFAVPAGPGPLG : 175
 f

 * 220 * 240 *
XP_0024419 : LSSSRAGTYGVVRNCNVPTA--------AAAVALLD-GHRRHDMSKDHGR : 225
RLM99791.1 : LISSSSGTFS-VRNCHVPIN-VAFSSPSE-AAAAPD-DHR-HGMGKDEHG : 226
XP_0049782 : SLSSP-GTFSVIRNCNVPTT-VAFSSSRE-VAATPD-DHR-HDMGRDDHG : 228
RLN28584.1 : LVSSS-GTFS-VRNCHVPTNVVTFSSTREAAAAAPD-DHR-HGMVKDEHG : 227
OEL26485.1 : LVSSS-GTCN-VRNHNVPAT-IAFSSSRE-VAATPN-DHR-RDMGKDDDN : 228
OEL14395.1 : LLGPSYEAVRYSYSGKQPAAPVSITFSSPREALAMDTGHR----GRHEQH : 236
XP_0273439 : NNLHFRVPSYCSTMLQDRSQ-----SVEFYDFLQVNTDSNKSEVTDNVKR : 193
XP_0202521 : SSQFSDRLPYYHRYRGLGSA-----SPFSIGFSFVDDECKFVKIGDNS-- : 221
XP_0239272 : KDLFSQHLAPCINPRQEKKQ-----PIEFYDFLQVNTESNGSEVIDNPRR : 213
AKE81092.1 : KEFYNEDPSHCE----DQSR-----PVEFYDFLQVNTDSNKSEVIDNARR : 209
ScMYB : LG--RPGTSYGVSNSNVP----------AAAAVVLD-GHR-HDMSKYLLG : 211
 1

 260 * 280
XP_0024419 : --RGGDDGGAGAVANRKD--VQFFDFLGVGI---- : 252
RLM99791.1 : -GNEDDEDGGAAAAKRKD--VPFIDFLGVGI---- : 254
XP_0049782 : HRKDDDEDGDGTASKRKDVDVPFFDFLGVGI---- : 259
RLN28584.1 : -GNDDDEDGGGAAAKRKA--VPFIDFLGVGI---- : 255
OEL26485.1 : GGNDDDEDGGGAAAKRKD-DVLFFDFLGVGI---- : 258
OEL14395.1 : -QKDYHASDGEEPLKRKD--VPFIDFLGVGVSS-- : 266
XP_0273439 : DDEEVN-QDAAGS-KNKED-VSFIDFLSVG--SS- : 222
XP_0202521 : -NEVMMKHEDNES--LKKD-VPFIDFLGVENR--- : 249
XP_0239272 : DDEEVD-QEAKEQ-QSKAV-VPFIDFLSAG--SS- : 242
AKE81092.1 : DDEEVDQQEAMENDQSKGD-VPFIDFFSVNGKSSS : 243
ScMYB : --RGDDNG--GAVAKRKDD-VQFFDFLGVGI---- : 237
 K V F DFl vg

### (B). MYB Molecular Phylogenetic analysis by Maximum Likelihood method


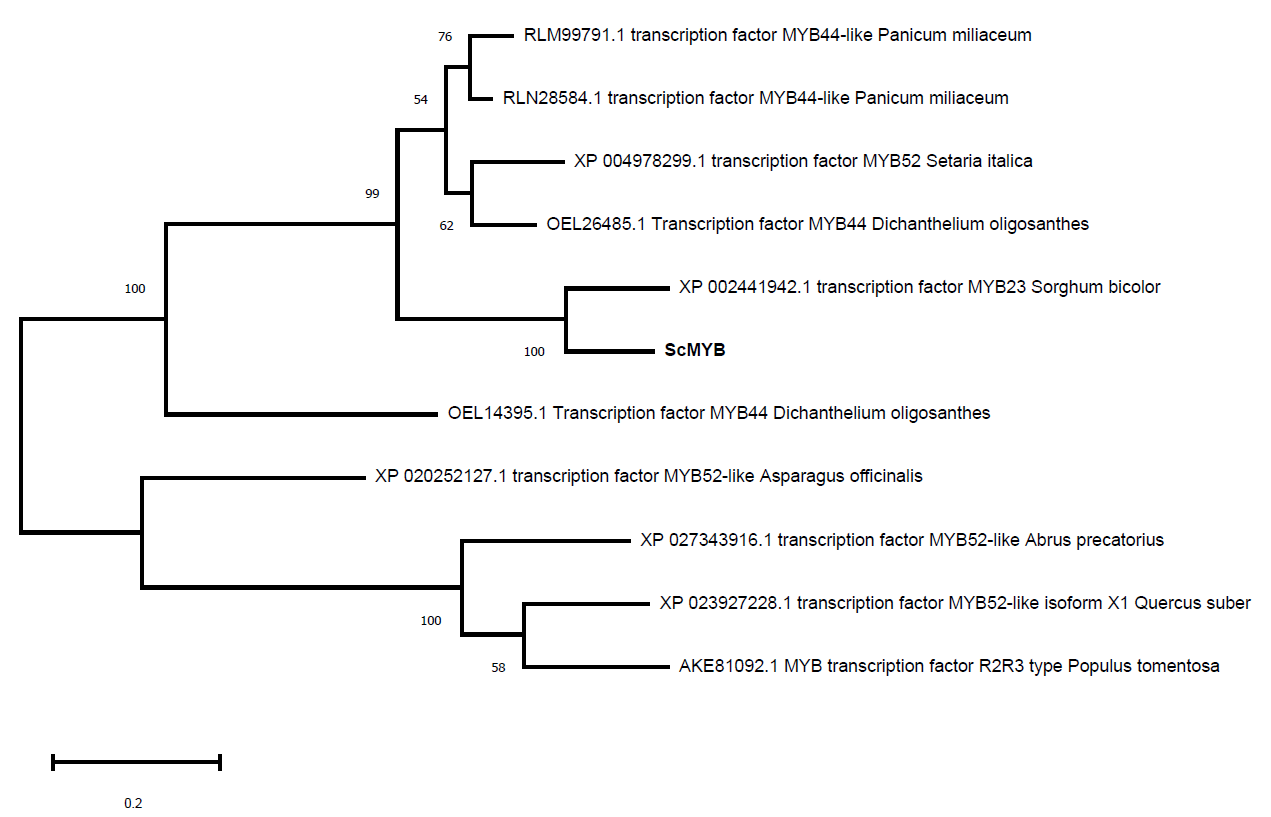


## Fig S13. Sequence alignment of Anthocyanidin reductase (ANR) proteins from sugarcane and various other plants, and phylogenetic relationships of Anthocyanidin reductase from sugarcane and various other plants.

### (A). Multiple sequence alignment of ScANR2 gene.

* 140 * 160 * 180
Q5FB34.1_R : CTRAKSVKRVILTSSAAAVTIN-QLDGTGLVVDEKNWTDIEFLTSAKPPTWG-------- : 167
D7U6G6.1_R : CTRAKSVKRVILTSSAAAVTIN-QLDGTGLVVDEKNWTDIEFLTSAKPPTWG-------- : 167
Q7PCC4.1_R : CTRAKSVKRVILTSSAAAVTIN-QLDGTGLVVDEKNWTDIEFLTSAKPPTWG-------- : 167
Q9SEV0.2_R : CLKSKSVKRVIYTSSAAAVSIN-NLSGTGIVMNEENWTDVEFLTEEKPFNWG-------- : 168
Q5XLY0.1_R : CTKVDSIKRVVVTSSAATVSIN-NSSEQNQYIDESCWTDVNFLTSQKPPGWA-------- : 171
ScANR2 : CVKAGTVRRVILTSSVAGVYIRPDLQGDGHVLDEASWSDVEYLRANKPPTWVRRPICTHS : 178
 C 4 364RV6 TSSaA V In l g g v61E W3D6e5Lt KPp W

 * 200 * 220 * 240
Q5FB34.1_R : ---------------------------YPASKTLAEKAAWKFAEENNIDLITVIPTLMAG : 200
D7U6G6.1_R : ---------------------------YPASKTLAEKAAWKFAEENNIDLITVIPTLMAG : 200
Q7PCC4.1_R : ---------------------------YPASKTLAEKAAWKFAEENNIDLITVIPTLMAG : 200
Q9SEV0.2_R : ---------------------------YPISKVLAEKTAWEFAKENKINLVTVIPALIAG : 201
Q5XLY0.1_R : ---------------------------YPVSKTLAEQAALKYAEEHSLDVVTVIPVLVVG : 204
ScANR2 : PWDALSSVDHGEDSISLMVTWIACLQGYCVSKVLLENEASRFAAEHGISLVTVCPVITVG : 238
 Yp SK LaE A 5A E 6 66TViP 6 G

 * 260 * 280 * 300
Q5FB34.1_R : SSLTSDVPSSIGLAMSLITGNEFLINGMKGMQMLSGSVSIAHVEDVCRAHIFVAEKESAS : 260
D7U6G6.1_R : SSLTSDVPSSIGLAMSLITGNEFLINGMKGMQMLSGSVSIAHVEDVCRAHIFVAEKESAS : 260
Q7PCC4.1_R : SSLTSDVPSSIGLAMSLITGNEFLINGMKGMQMLSGSVSIAHVEDVCQAHIFVAEKESAS : 260
Q9SEV0.2_R : NSLLSDPPSSLSLSMSFITGKEMHVTGLKEMQKLSGSISFVHVDDLARAHLFLAEKETAS : 261
Q5XLY0.1_R : PAVTPTVPSSVELALSLITGDEFKMGALKGMQFVSGSISLVHIDDVCSAQIFLMEKPSAQ : 264
ScANR2 : AAPAPKVRTSIIDSLSMLSGDEAGLAVLRGIETTSGALPLVHIDDLCRAELFLAEAAAAD : 298
 vp3S6 l 6S 63G E 6 64g62 SGs6s H6 D6c A 6F6aEk A

 * 320 * 340 * 360
Q5FB34.1_R : GRYICCAANTSVPELAKFLSKRYPQYKVPTDFGD---FPPKSKLIISSEKLVKEGFSFKY : 317
D7U6G6.1_R : GRYICCAANTSVPELAKFLSKRYPQYKVPTDFGD---FPSKSKLIISSDKLVKEGFSFKY : 317
Q7PCC4.1_R : GRYICCAANTSVPELAKFLSKRYPQYKVPTDFGD---FPPKSKLIISSEKLVKEGFSFKY : 317
Q9SEV0.2_R : GRYICCAYNTSVPEIADFLIQRYPKYNVLSEFEE---GLSIPKLTLSSQKLINEGFRFEY : 318
Q5XLY0.1_R : GRYICFPVNTGIPQLAEFLSKRYPQYKVPTKFDD---VPATPKLTISSQKLLDCGFSFKY : 321
ScANR2 : GRYICCSLNTTVVELARFLAHKYPQYGVKTNFDDDKQLLERPRVIMSSEKLVREGFQYRH : 358
 GRYICc NT 6p26A FL 4YPqY V 3 F d 46 6SS KL6 eGF 5 y

 * 380
Q5FB34.1_R : G-IEEIYDESVEYFKAKGLLQN- : 338
D7U6G6.1_R : G-IEEIYDESVEYFKAKGLLQN- : 338
Q7PCC4.1_R : G-IEEIYDESVEYFKAKGLLQN- : 338
Q9SEV0.2_R : G-INEMYDQMIEYFESKGLIKAK : 340
Q5XLY0.1_R : G-IEDIYDQAIEYMKTKGLLTC- : 342
ScANR2 : NTLDDIYDNVVEYGKALGILPN- : 380
 g 6 6YD 6EY k kG66

### (B). Molecular Phylogenetic analysis by Maximum Likelihood method (ScANR2)


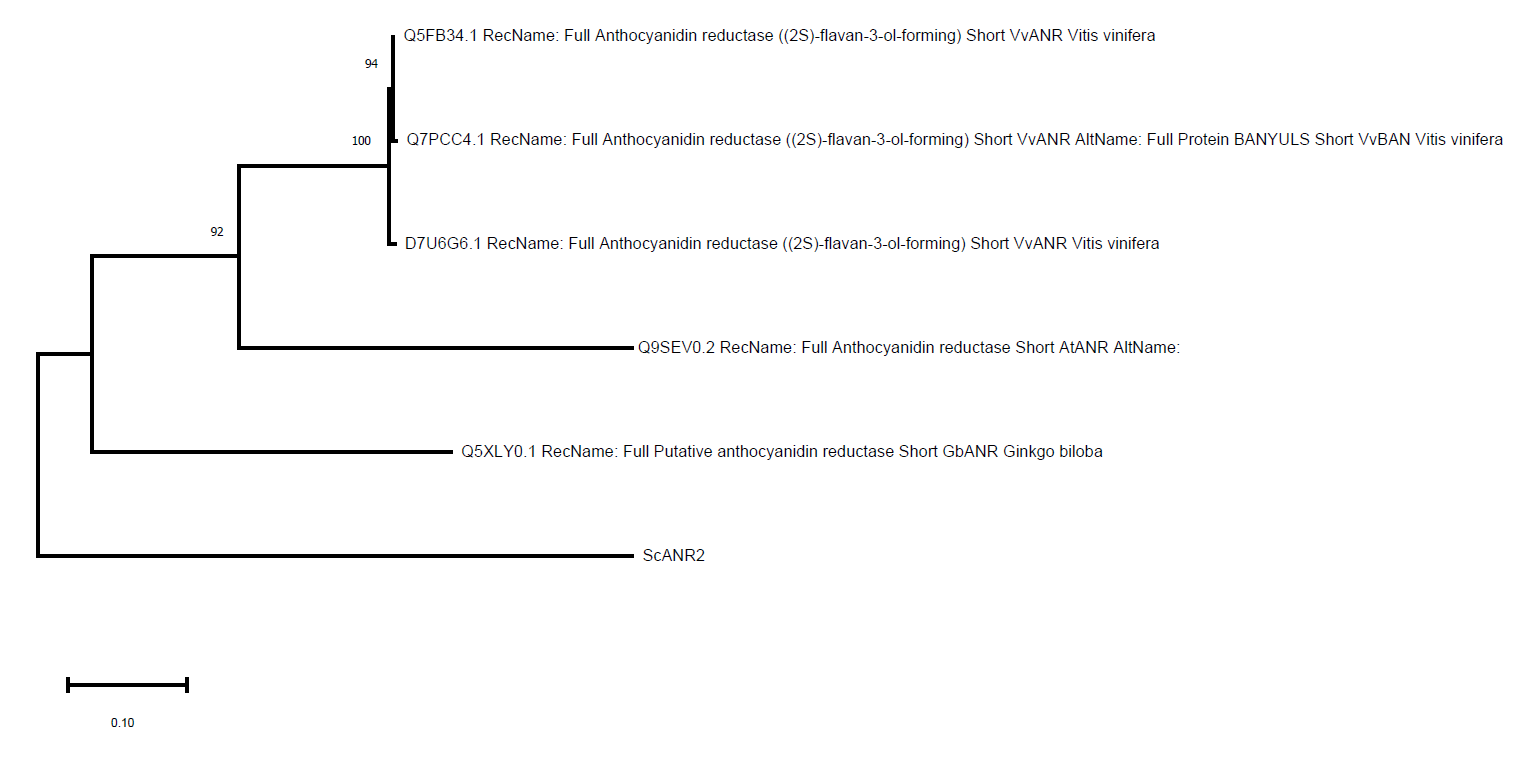


## Fig S14. Sequence alignment of Anthocyanidin 3-O-glucosyltransferase (BZ1) proteins from sugarcane and various other plants, and phylogenetic relationships of Anthocyanidin 3-O-glucosyltransferase.

### (A). Multiple sequence alignment of ScBZ1_1 gene.

* 20 * 40 * 60
P16167.1 : MAPADGESSPPPHVAVVAFPFSSHAAVLLSIARALAAAAAPSGATLSFLSTASSLAQLRK : 60
P16166.1 : MAPADGESSPPPHVAVVAFPFSSHAAVLLSIARALAAAAAPSGATLSFLSTASSLAQLRK : 60
P14726.1 : MAP------PPPHIAVVAFPFSSHAAVLFSFARALAAAAP-AGTSLSFLTTADNAAQLRK : 53
P16165.1 : MAPADGESSPPPHVAVVAFPFSSHAAVLLSIARALAAAAAPSGATLSFLSTASSLAQLRK : 60
Q9LFJ8.1 : MTKPS-DPTRDSHVAVLAFPFGTHAAPLLTVTRRLASASP--STVFSFFNTAQSNSSLFS : 57
A0A2Z5CVA1 : MDS-----ASRQHVALIAFPFASHPGNLFAFARALAAAAP--DITFSFLTTTFAAATLPP : 53
ScBZ1_1 : -RRVRRVAMGSPHVAVVAFPFASHAPKLLMVARALATAAP--SATFSFISTADSLARLG- : 56
 m H6A66AFPF 3Ha L aRaLA Aa SF Ta a L

 * 80 * 100 * 120
P16167.1 : ASSASAGHGLPGNLRFVEVPDGAP-AAEESVPVP-RQMQLFMEAAEAGGVKAWLEAARAA : 118
P16166.1 : ASSASAGHGLPGNLRFVEVPDGAP-AAEETVPVP-RQMQLFMEAAEAGGVKAWLEAARAA : 118
P14726.1 : AG------ALPGNLRFVEVPDGVP-PGETSCLSPPRRMDLFMAAAEAGGVRVGLEAACAS : 106
P16165.1 : ASSASAGHGLPGNLRFVEVPDGAP-AAEETVPVP-RQMQLFMEAAEAGGVKAWLEAARAA : 118
Q9LFJ8.1 : SGDE---ADRPANIRVYDIADGVP-EGYVFSGRPQEAIELFLQAAPE-NFRREIAKAETE : 112
A0A2Z5CVA1 : AP-------PAANLRLCHVADGVPEGGLPPGTTIHGRIGMFLRATPG-NFRDGVRAAEEE : 105
ScBZ1_1 : VS------AVPGNLRFVEVP--AA-GGDDKGILPWRRMELFVEAAEAGGLRQALEMARAA : 107
 p N6R 6 dg p p 6 6F6 Aa 4 6 A

 * 140 * 160 * 180
P16167.1 : AGGARVTCVVGDAFVWPAADAAASAGAPWVPVWTAASCALLAHIRTDALREDVG---DQA : 175
P16166.1 : AGGARVTCVVGDAFVWPAADAAASAGAPWVPVWTAASCALLAHIRTDALREDVG---DQA : 175
P14726.1 : AGGARVSCVVGDAFVW-TADAASAAGAPWVAVWTAASCALLAHLRTDALRRDVG---DQA : 162
P16165.1 : AGGARVTCVVGDAFVWPAADAAASAGAPWVPVWTAASCALLAHIRTDSLREDVG---DQA : 175
Q9LFJ8.1 : VG-TEVKCLMTDAFFWFAADMATEINASWIAFWTAGANSLSAHLYTDLIRETIG--VKEV : 169
A0A2Z5CVA1 : VG-VKVSCVVSDAFLWMTADVAEEIGAQWLPLWTCAPAALLAHVSTDQLRERFGVEKQAT : 164
ScBZ1_1 : AGGARVTCVVGDAFMS----MAAEAGVPWVAVWTGGPCALLAHLIGDAIREDIS---DHA : 160
 G V C66 DAF w ad A ga W6 WT aLlAH6 tD 6Re g

 * 200 * 220 * 240
P16167.1 : ANRVDEPLISHPGLASYRVRDLPDG--VVSGDFNYVINLLVHRMGQCLPRSAAAVALNTF : 233
P16166.1 : ANRVDGLLISHPGLASYRVRDLPDG--VVSGDFNYVINLLVHRMGQCLPRSAAAVALNTF : 233
P14726.1 : ASRADELLVAHAGLGGYRVRDLPDG--VVSGDFNYVISLLVHRQAQRLPKAATAVALNTF : 220
P16165.1 : ANRVDEPLISHPGLASYRVRDLPDG--VVSGDFNYVISLLVHRMGQCLPRSAAAVALNTF : 233
Q9LFJ8.1 : GERMEETIGVISGMEKIRVKDTPEG--VVFGNLDSVFSKMLHQMGLALP-RATAVFINSF : 226
A0A2Z5CVA1 : AGWADELVDFIPGLSCLRIRDIPDE---IVTNWHSDLSILLHRMGNQLT-SATAVALNTF : 220
ScBZ1_1 : ANRAEELLTSHPGLGSFRVRDLPFGGVGASGDMHRVMSLLLSRLAQRLPRAATAVALNAF : 220
 a r e 6 G6 R64D P g g1 v 66hr Lp A AVa6N F

 * 260 * 280 * 300
P16167.1 : PGLDPPDVTAALAEILPNCVPFGPYHLLL-AEDDADT-AAPADPHGCLAWLGRQPARGVA : 291
P16166.1 : PGLDPPDVTAALAEILPNCVPFGPYHLLL-AEDDADT-AAPADPHGCLAWLGRQPARGVA : 291
P14726.1 : PGLDPPDLIAALAAELPNCLPLGPYHLLPGAEPTADTNEAPADPHGCLAWLDRRPARSVA : 280
P16165.1 : PGLDPPDVTAALAEILPNCVPFGPYHLLL-AEDDADT-AAPADPHGCLAWLGRQPARGVA : 291
Q9LFJ8.1 : EDLDPT-LTNNLRSRFKRYLNIGPLGLLS---STLQQ--LVQDPHGCLAWMEKRSSGSVA : 280
A0A2Z5CVA1 : DGLDTT-IDAALASLFKKTLPIGPLNLLS--SPPPLQ---PGDEK-CLSWLDGQEDATVA : 273
ScBZ1_1 : PGLFPQDVSAALANALPNSLPIGPYHLLP--GAAAPT----DDPHDCLAWLAQRPAGTVA : 274
 gLdp 6 aaLa 6p GP LL Dph CLaW6 VA

 * 320 * 340 * 360
P16167.1 : YVSFGTVACPRPD-ELRELAAGLEASGAPFLWSLREDSWT-LLPPGFLDRAAGTGSGLVV : 349
P16166.1 : YVSFGTVACPRPD-ELRELAAGLEDSGAPFLWSLREDSWP-HLPPGFLDRAAGTGSGLVV : 349
P14726.1 : YVSFGTNATARPD-ELQELAAGLEASGAPFLWSLR-GVVA-AAPRGFLERAP----GLVV : 333
P16165.1 : YVSFGTVACPRPD-ELRELAAGLEASAAPFLWSLREDSWT-LLPPGFLDRAAGTGSGLVV : 349
Q9LFJ8.1 : YISFGTVMTPPPG-ELAAIAEGLESSKVPFVWSLKEKSLV-QLPKGFLDRTRE--QGIVV : 336
A0A2Z5CVA1 : YVSFGTMVLMPTQSDVSEIAQGLESSGVRFLWSLREEARAGLLPPGFLERTAG--RGLVV : 331
ScBZ1_1 : YVSFGTVAALPPD-ELRELASGLEASGAPFLWSLREDAWP-LLPPGFVDRAKANGSGLLV : 332
 Y6SFGT p e6 e6A GLE S pF6WSL4e lP GF6 R G66V

 * 380 * 400 * 420
P16167.1 : PWAPQVAVLRHPSVGAFVTHAGWASVLEGVSSGVPMACRPFFGDQRMNARSVAHVWGFGA : 409
P16166.1 : PWAPQVAVLRHPSVGAFVTHAGWASVLEGLSSGVPMACRPFFGDQRMNARSVAHVWGFGA : 409
P14726.1 : PWAPQVGVLRHAAVGAFVTHAGWASVMEGVSSGVPMACRPFFGDQTMNARSVASVWGFGT : 393
P16165.1 : PWAPQVAVLRHPSVGAFVTHAGWASVLEGVSSGVPMACRPFFGDQRMNARSVAHVWGFGA : 409
Q9LFJ8.1 : PWAPQVELLKHEATGVFVTHCGWNSVLESVSGGVPMICRPFFGDQRLNGRAVEVVWEIGM : 396
A0A2Z5CVA1 : PWAPQVRVLGHRAVGAFVTHCGWNAVMESVTSGVPMACLPSFADQKTNARMVSAAWGIGE : 391
ScBZ1_1 : PWTPQAAVLRHPAVGAFVTHSGWGAVVEGMSGGVPMACRPFFGDQQMNARAVAHLWCFGT : 392
 PWaPQv 6L H vGaFVTH GW V6E 63 GVPMaCrPfFgDQ NaR V W G

 * 440 * 460 * 480
P16167.1 : AF--EGAMTSAGVAAAVEELLRGEEGARMRARAKVLQALVAEAFGPGGECRKNFDRFVEI : 467
P16166.1 : AF--EGAMTSAGVATAVEELLRGEEGARMRARAKELQALVAEAFGPGGECRKNFDRFVEI : 467
P14726.1 : AF--DGPMTRGAVANAVATLLRGEDGERMRAKAQELQAMVGKAFEPDGGCRKNFDEFVEI : 451
P16165.1 : AF--EGAMTSAGVAAAVEELLRGEEGAGMRARAKELQALVAEAFGPGGECRKNFDRFVEI : 467
Q9LFJ8.1 : TII-NGVFTKDGFEKCLDKVLVQDDGKKMKCNAKKLKELAYEAVSSKGRSSENFRGLLDA : 455
A0A2Z5CVA1 : ALR-GEKVTKEEVVRSMEIVMMGEEGRRMRERIGNLREKAAEAVGPGGSSSENFKSVLEM : 450
ScBZ1_1 : AFGDDTPMTSGVVAEVVTSLLTGAEGARMRATARDLRARVVEAFGPDGGSVNNFHKFVEV : 452
 a T v 6 66 g G M4 a L eA p G NF 6e


P16167.1 : VCRA-- : 471
P16166.1 : VCRA-- : 471
P14726.1 : VCRV-- : 455
P16165.1 : VCRA-- : 471
Q9LFJ8.1 : VVNII- : 460
A0A2Z5CVA1 : VRGTAN : 456
ScBZ1_1 : VCARV- : 457

### (B). Molecular Phylogenetic analysis by Maximum Likelihood method (ScBZ1_1)


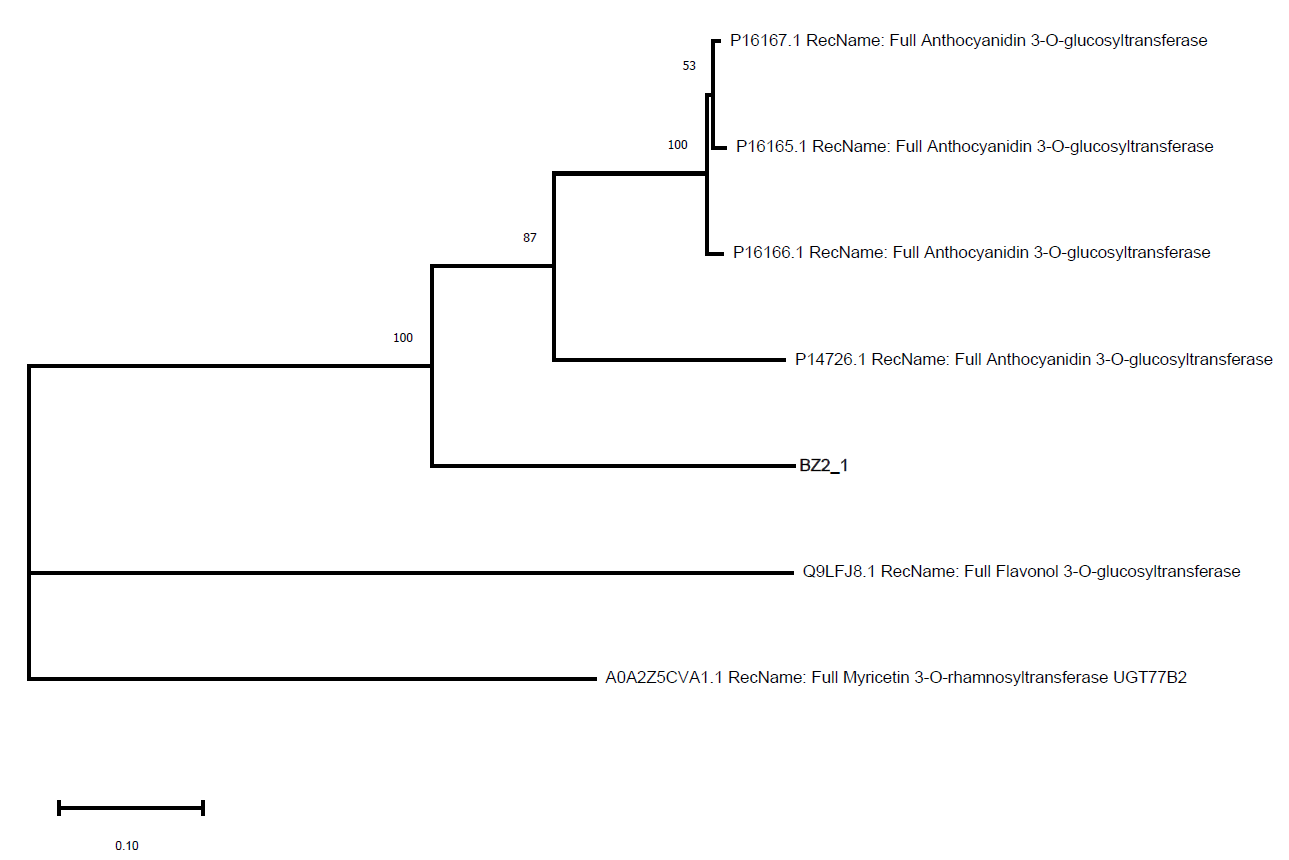


### (C). Multiple sequence alignment of ScBZ1_2 gene.

* 20 * 40 * 60
P16167.1 : ------------------MAP--------ADGESSPPPHVAVVAFPFSSHAAVLLSIARA : 34
P16165.1 : ------------------MAP--------ADGESSPPPHVAVVAFPFSSHAAVLLSIARA : 34
P16166.1 : ------------------MAP--------ADGESSPPPHVAVVAFPFSSHAAVLLSIARA : 34
P14726.1 : ------------------MAP--------------PPPHIAVVAFPFSSHAAVLFSFARA : 28
Q9LFJ8.1 : ---------------MTKPSD------------PTRDSHVAVLAFPFGTHAAPLLTVTRR : 33
A0A2Z5CVA1 : ------------------MDS-------------ASRQHVALIAFPFASHPGNLFAFARA : 29
P51094.2 : ------------------MSQ------------TTTNPHVAVLAFPFSTHAAPLLAVVRR : 30
ScBZ2_2 : HTCALPIFSSHRIACAADASPSLAEAKTMAPAASSPPPHVAVVAFPFSSHAAVLLSFARA : 60
 m pH6A66AFPFs3Haa Ll aRa

 * 80 * 100 * 120
P16167.1 : LAAAAAPSGATLSFLSTASSLAQLRKASS---ASAGHGLPGNLRFVEVPDGAPAAEES-- : 89
P16165.1 : LAAAAAPSGATLSFLSTASSLAQLRKASS---ASAGHGLPGNLRFVEVPDGAPAAEET-- : 89
P16166.1 : LAAAAAPSGATLSFLSTASSLAQLRKASS---ASAGHGLPGNLRFVEVPDGAPAAEET-- : 89
P14726.1 : LA-AAAPAGTSLSFLTTADNAAQLRKAGA---------LPGNLRFVEVPDGVPPGETSC- : 77
Q9LFJ8.1 : LA-SASPS-TVFSFFNTAQSNSSLFSSGD------EADRPANIRVYDIADGVPEGYVFS- : 84
A0A2Z5CVA1 : LA-AAAPD-ITFSFLTTTFAAATLPPAPP----------AANLRLCHVADGVPEGGLPPG : 77
P51094.2 : LA-AAAPH-AVFSFFSTSQSNASIFHDS-------MHTMQCNIKSYDISDGVPEGYVFA- : 80
ScBZ2_2 : LAVAAAPSGATLSFLSTASSLAQLRKAAAGGGGSAGHGLPGNLRFVEVPDGAPAATEGP- : 119
 LA aAaP SFl Ta s a 6 a p N64 6 DG P

 * 140 * 160 * 180
P16167.1 : VPVPRQMQLFMEAAEAGGVKAWLEAARAAAGGARVTCVVGDAFVWPAADAAASAGAPWVP : 149
P16165.1 : VPVPRQMQLFMEAAEAGGVKAWLEAARAAAGGARVTCVVGDAFVWPAADAAASAGAPWVP : 149
P16166.1 : VPVPRQMQLFMEAAEAGGVKAWLEAARAAAGGARVTCVVGDAFVWPAADAAASAGAPWVP : 149
P14726.1 : LSPPRRMDLFMAAAEAGGVRVGLEAACASAGGARVSCVVGDAFVW-TADAASAAGAPWVA : 136
Q9LFJ8.1 : GRPQEAIELFLQAAPE-NFRREIAKAETEVG-TEVKCLMTDAFFWFAADMATEINASWIA : 142
A0A2Z5CVA1 : TTIHGRIGMFLRATPG-NFRDGVRAAEEEVG-VKVSCVVSDAFLWMTADVAEEIGAQWLP : 135
P51094.2 : GRPQEDIELFTRAAPE-SFRQGMVMAVAETG-RPVSCLVADAFIWFAADMAAEMGLAWLP : 138
ScBZ2_2 : VPVPRQMQLFMAAAEAGGVKAGLEAARAAAGGARVSCVVGDAFVWPAADAAAAAGAPWVP : 179
 6 6F Aa 4 6 aA a G V C66 DAF W aAD A ga W6p

 * 200 * 220 * 240
P16167.1 : VWTAASCALLAHIRTDALREDVG---DQAANRVDEPLISHPGLASYRVRDLPDGVVSGDF : 206
P16165.1 : VWTAASCALLAHIRTDSLREDVG---DQAANRVDEPLISHPGLASYRVRDLPDGVVSGDF : 206
P16166.1 : VWTAASCALLAHIRTDALREDVG---DQAANRVDGLLISHPGLASYRVRDLPDGVVSGDF : 206
P14726.1 : VWTAASCALLAHLRTDALRRDVG---DQAASRADELLVAHAGLGGYRVRDLPDGVVSGDF : 193
Q9LFJ8.1 : FWTAGANSLSAHLYTDLIRETIG--VKEVGERMEETIGVISGMEKIRVKDTPEGVVFGNL : 200
A0A2Z5CVA1 : LWTCAPAALLAHVSTDQLRERFGVEKQATAGWADELVDFIPGLSCLRIRDIPDEIVT-NW : 194
P51094.2 : FWTAGPNSLSTHVYIDEIREKIG--VSGIQGREDELLNFIPGMSKVRFRDLQEGIVFGNL : 196
ScBZ2_2 : VWTAASCALLAHLRTDALREDVG---DQAANRADELLITHPGLARYRVRDLPDGVVSGDF : 236
 WTaa aLlaH6 tD 6Re G a r de 6 pG6 R 4D pdg6V g1

 * 260 * 280 * 300
P16167.1 : NYVINLLVHRMGQCLPRSAAAVALNTFPGLDPPDVTAALAEILPNCVPFGPYHLLL-AED : 265
P16165.1 : NYVISLLVHRMGQCLPRSAAAVALNTFPGLDPPDVTAALAEILPNCVPFGPYHLLL-AED : 265
P16166.1 : NYVINLLVHRMGQCLPRSAAAVALNTFPGLDPPDVTAALAEILPNCVPFGPYHLLL-AED : 265
P14726.1 : NYVISLLVHRQAQRLPKAATAVALNTFPGLDPPDLIAALAAELPNCLPLGPYHLLPGAEP : 253
Q9LFJ8.1 : DSVFSKMLHQMGLALP-RATAVFINSFEDLDPT-LTNNLRSRFKRYLNIGPLGLLS---- : 254
A0A2Z5CVA1 : HSDLSILLHRMGNQLT-SATAVALNTFDGLDTT-IDAALASLFKKTLPIGPLNLLS---- : 248
P51094.2 : NSLFSRMLHRMGQVLP-KATAVFINSFEELDDS-LTNDLKSKLKTYLNIGPFNLIT---- : 250
ScBZ2_2 : NYVISLLLHRMGQRLPRSAAAVALNTFPGLDPPEVTAALAEILPNCLPFGPYHLLL-PKD : 295
 s 66Hrmgq Lp A AVa6N3F gLDp 6taaLa l 6p GP L6

 * 320 * 340 * 360
P16167.1 : DADT-AAPADPHGCLAWLGRQPARGVAYVSFG-TVACPRPDELRELAAGLEASGAPFLWS : 323
P16165.1 : DADT-AAPADPHGCLAWLGRQPARGVAYVSFG-TVACPRPDELRELAAGLEASAAPFLWS : 323
P16166.1 : DADT-AAPADPHGCLAWLGRQPARGVAYVSFG-TVACPRPDELRELAAGLEDSGAPFLWS : 323
P14726.1 : TADTNEAPADPHGCLAWLDRRPARSVAYVSFG-TNATARPDELQELAAGLEASGAPFLWS : 312
Q9LFJ8.1 : -STLQQLVQDPHGCLAWMEKRSSGSVAYISFG-TVMTPPPGELAAIAEGLESSKVPFVWS : 312
A0A2Z5CVA1 : SPPP-LQPGDEK-CLSWLDGQEDATVAYVSFGTMVLMPTQSDVSEIAQGLESSGVRFLWS : 306
P51094.2 : -P--PPVVPNTTGCLQWLKERKPTSVVYISFG-TVTTPPPAEVVALSEALEASRVPFIWS : 306
ScBZ2_2 : DADT-AAPADPHGCLAWLDRHPERGVAYVSFG-TVASPRPDELRELAAGLEASGAPFLWS : 353
 p 1phgCLaW6 VaY6SFG tv p p e6 e6a gLE S pF6WS

 * 380 * 400 * 420
P16167.1 : LREDSWT-LLPPGFLDRAAGTGSGLVVPWAPQVAVLRHPSVGAFVTHAGWASVLEGVSSG : 382
P16165.1 : LREDSWT-LLPPGFLDRAAGTGSGLVVPWAPQVAVLRHPSVGAFVTHAGWASVLEGVSSG : 382
P16166.1 : LREDSWP-HLPPGFLDRAAGTGSGLVVPWAPQVAVLRHPSVGAFVTHAGWASVLEGLSSG : 382
P14726.1 : LR-GVVA-AAPRGFLERAP----GLVVPWAPQVGVLRHAAVGAFVTHAGWASVMEGVSSG : 366
Q9LFJ8.1 : LKEKSLV-QLPKGFLDRTRE--QGIVVPWAPQVELLKHEATGVFVTHCGWNSVLESVSGG : 369
A0A2Z5CVA1 : LREEARAGLLPPGFLERTAG--RGLVVPWAPQVRVLGHRAVGAFVTHCGWNAVMESVTSG : 364
P51094.2 : LRDKARV-HLPEGFLEKTRG--YGMVVPWAPQAEVLAHEAVGAFVTHCGWNSLWESVAGG : 363
ScBZ2_2 : LREDAWP-LLPPGFLDRTAGAGSGLVVPWAPQVPVLRHPSVGAFVTHAGWASVLEGVSSG : 412
 L4e lP GFL 4 g G6VVPWAPQv 6L H vGaFVTH GW s6 E 6 sG

 * 440 * 460 * 480
P16167.1 : VPMACRPFFGDQRMNARSVAHVWGFGAAFEG-AMTSAGVAAAVEELLRGEEGARMRARAK : 441
P16165.1 : VPMACRPFFGDQRMNARSVAHVWGFGAAFEG-AMTSAGVAAAVEELLRGEEGAGMRARAK : 441
P16166.1 : VPMACRPFFGDQRMNARSVAHVWGFGAAFEG-AMTSAGVATAVEELLRGEEGARMRARAK : 441
P14726.1 : VPMACRPFFGDQTMNARSVASVWGFGTAFDG-PMTRGAVANAVATLLRGEDGERMRAKAQ : 425
Q9LFJ8.1 : VPMICRPFFGDQRLNGRAVEVVWEIGMTIINGVFTKDGFEKCLDKVLVQDDGKKMKCNAK : 429
A0A2Z5CVA1 : VPMACLPSFADQKTNARMVSAAWGIGEALRGEKVTKEEVVRSMEIVMMGEEGRRMRERIG : 424
P51094.2 : VPLICRPFFGDQRLNGRMVEDVLEIGVRIEGGVFTKSGLMSCFDQILSQEKGKKLRENLR : 423
ScBZ2_2 : VPMACRPFFGDQRMNARSVAHVWGFGAAFEA-GMTRAGVAAAVEELLRGEEGARMRARAQ : 471
 VP6aCrPfFgDQ NaR V vwg G a g T g 66 ge G 64 a

 * 500 *
P16167.1 : VLQALVAEAFGPGGECRKNFDRFVEIVCRA--- : 471
P16165.1 : ELQALVAEAFGPGGECRKNFDRFVEIVCRA--- : 471
P16166.1 : ELQALVAEAFGPGGECRKNFDRFVEIVCRA--- : 471
P14726.1 : ELQAMVGKAFEPDGGCRKNFDEFVEIVCRV--- : 455
Q9LFJ8.1 : KLKELAYEAVSSKGRSSENFRGLLDAVVNII-- : 460
A0A2Z5CVA1 : NLREKAAEAVGPGGSSSENFKSVLEMVRGTAN- : 456
P51094.2 : ALRETADRAVGPKGSSTENFITLVDLVSKPKDV : 456
ScBZ2_2 : ELQAAVAEAFGPGGACRKNFDEFVQIVCRP--- : 501
 L eA gp G NF 6 V

### (D). Molecular Phylogenetic analysis by Maximum Likelihood method (ScBZ1_2)


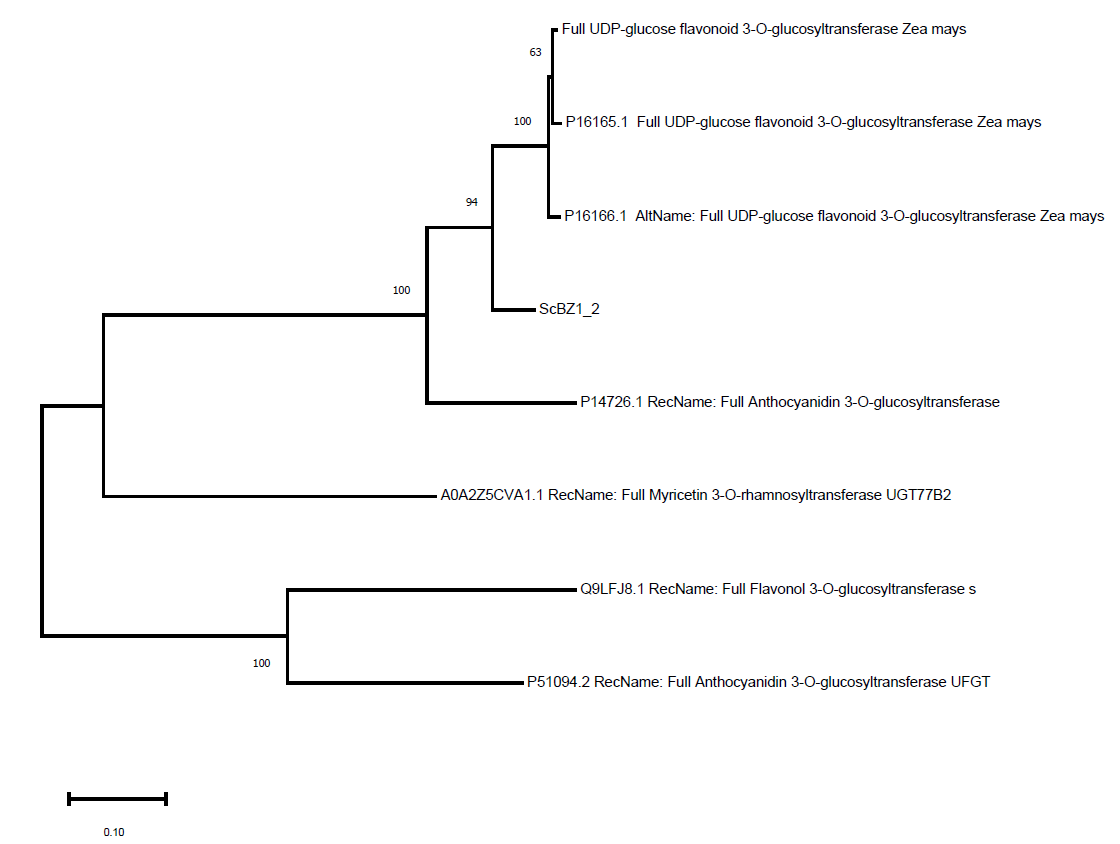


### (E). Multiple sequence alignment of ScBZ1_3 gene.

* 20 * 40 * 60
XP_0024378 : ----------------------------------------------------------MA : 2
XP_0024365 : ----------------------------------------------------------MA : 2
XP_0345794 : --------------------------------------------------------MASA : 4
XP_0049577 : --------------------------------------------------------MASA : 4
XP_0258039 : --------------------------------------------------------MASA : 4
XP_0035601 : ----------------------------------------------------------MA : 2
RLM87236.1 : --------------------------------------------------------MASA : 4
RLN34493.1 : MLLNQRFTLILVYHSKIHFKCNISFATHRGWKINTPPLSCFPRIHDDAGGLNPHPSMATA : 60
RLM86688.1 : ----------------------------------------------------------MA : 2
XP_0156454 : ----------------------------------------------------MAPAMASS : 8
BZ1_3 : ---------------------------------------SPNHHIILTINRTHESQLRMA : 21
 a

 * 80 * 100 * 120
XP_0024378 : YPTVVLIPLCVPGHLTSMLEAGKRLLAT--------SRCPMSLTVLVTQMTMSANLMSDV : 54
XP_0024365 : YPTLVLIPFCVPGHLTSMLEAGKRLLAT--------SRCAMSLTVLVTQMTMSANLMSDV : 54
XP_0345794 : SPTIVLVPLCVPGHLPPLFEAGKRLL----------SSGAMSLTVLFMQMTMAANLMSDV : 54
XP_0049577 : SPTIVLVPLCVPGHLPPLFEAGKRLL----------SSGAMSLTVLFMQMTMAANLMSDV : 54
XP_0258039 : APTIVLVPLCVPGHLPSLFEAGKRLLRT--------SGGAMSLTVLFMQMTMEANLMSDV : 56
XP_0035601 : IPTIVLIPFCVTGHLTSMVEAGKRLLSS--------SSRPLSLTMLVTPMSMDK-LTSEL : 53
RLM87236.1 : APTIVLVPLCVPGHLPSLFEAGKRLLGT--------SGGAMSLTVLFMQMTMEANLMSDV : 56
RLN34493.1 : SPTLVLLPLWSAGHLMSLLEAGKRLLTR--------AGGKLSLTVLVIPPPTEH-LAAEV : 111
RLM86688.1 : TPTVVLLPLWSAGHLMSLLEAGKRLLAR--------AGGRLSLTVLVIPPPTEK-LAAEV : 53
XP_0156454 : AATVVLIPFCVSGHLTPMLEVGKRMLRSRCCGDDDDGRPAMSLTVLLAQLPESH-RAPEI : 67
BZ1_3 : YPTVVLIPLCVPGHLTSMLEAGKRLLGR--------SRCPMSLTVLVTQMTMSANLMSDV : 73
 pT6VL6P cv GHL 6 EaGKR6L 6SLT6L l 6

 * 140 * 160 * 180
XP_0024378 : ADIIRREAA----DSAFDIRFVHLPAVELPTAADGLEDFMMRFIQLHDTYVKEAVSGISS : 110
XP_0024365 : ADIIRREEA----DSGFDIRFVHLPAVELPTAADGLEAFMMRFIQLHDTYVKEAISGMSS : 110
XP_0345794 : TDLIRRES-----ESGLDIRFHHLPAVELPTDSHGTEDFIMRFIQLHAPHVKAALSGLAS : 109
XP_0049577 : TDLIRRES-----ESGLDIRFHHLPAVELPTDSHGTEDFIMRFIQLHAPHVKAALSGLAS : 109
XP_0258039 : ADLIRREA-----GSGLDIRFHHLPAVELPTDSHGTENFIMHFIQLHAPHVKATLSGLAS : 111
XP_0035601 : ADIIRRET-----ESGFEIRFHHLPAVELPQDFHGAEDFISRFVQLHAPGAKAAISGLAS : 108
RLM87236.1 : ADLIRREA-----GSGLDIRFHHLPAVELPTDSHGTENFIMHFIQLHAPHVKAALSGLAS : 111
RLN34493.1 : QGHIRREE-----ASGLDVRFVHLPAMDPPTDFRGIEEFLSRFVQMHAPRVRAAVADLPC : 166
RLM86688.1 : EGHIRREE-----ASGLDVRFVRLPAVEPPTDFRGIEEFLSRLVQMHAPDVRAAVAALPC : 108
XP_0156454 : DEIIRREAAGASEHSGFDVRFHCLPAEELP-DFRGGEDFISRFMQQHASHAREAIAGLES : 126
BZ1_3 : ADIIRREA-----DSGLDIRFVHLPAVELPTADHGLEDFMVRFIQLHGTYVKEAVSGMSS : 128
 IRRE Sg d6RF hLPA elPt G E F6 rf6Q H v4 a6 g6 s

 * 200 * 220 * 240
XP_0024378 : -PVAAVVIDYFCTTLLDVTRELALPVYVYLTSGASMLALLLRLPALDEEIAGDFEAMGEA : 169
XP_0024365 : SPVAAVVVDFFCTALFDVTRELALPVYVYFTSGASMLALMLRLPALDGEIAGDFEAMG-E : 169
XP_0345794 : -PVAAVVVDYFCTTLFDVTRELALPVYAYLPCSASMLALILRLPALDEEVSGDLGDM--E : 166
XP_0049577 : -PVAAVVVDYFCTTLFDVTRELALPVYAYLPCSASMLALILRLPALDEEVSGDLGDM--E : 166
XP_0258039 : -PVAAVVVDYFCTTLFDAIRELALPVYVYVPCSAAMLAIILRLPALDEEVAGDLGDM--E : 168
XP_0035601 : -PVSAVVMDYFCTTLFDVTRELGLPAYVYFTSAASMLALMLRLPSLDKEVAVGFEEL--D : 165
RLM87236.1 : -PVAAVVVDYFCTTLFDAIRELALPVYVYVPCSAAILAIILRLPALDEEVAGDLGDM--E : 168
RLN34493.1 : -PVAALVLDFFCTALIDVARDLDVPAYVYFTTNAAMMALMLRLPALHEEVTVEFEEM--E : 223
RLM86688.1 : -PMAALVLDFFCTALIDVARDLAVPAYVYFTTNAAMLALMLRLPALHEEVTVEFEEM--E : 165
XP_0156454 : -RVAAVVLDWFCTTLLDVTRDLGLPGYVFFTSAASMLSLLLRLPALDKEVAVDFEEMG-- : 183
BZ1_3 : -PVAAVVMDYFCTTLFDVTRELALPVYVYLTSGASMLALLLRLPALDEEISGDFEAM--E : 185
 p6aA6V6D5FCT L Dv R L 6P Yv5 A 66a66LRLPaLd E6 6

 * 260 * 280 * 300
XP_0024378 : AAVDLPGMPPVPARLMPTPIMTK-NANFTWLVYHGNRFMEAAGIIVNTVAELEQSILAAI : 228
XP_0024365 : GAVDLPGMPPVPARLMPSPIMRK-DPNYAWFVYHGNRFMEAAGIIVNTVAKLEPSILAAI : 228
XP_0345794 : -AVDVPGMPPVPAALLPTPLMTR-GPNYAWLVYHGKRIMEAAGVIVYTVAELEPNVLAAI : 224
XP_0049577 : -AVDVPGMPPVPAALLPTPLMTR-GPNYAWLVYHGKRIMEAAGVIVYTVAELEPNVLAAI : 224
XP_0258039 : GAVEVPGMPPVPAALLPTPLLKK-GPNYAWMVYHGRRIMEAAGIIVYTVAELEPPVLAAI : 227
XP_0035601 : GPVNVPGMPPVPAASMPKPMMKK-DANYAWFVYHGNRFMDAAGIIVNTVAGLEPAILEAI : 224
RLM87236.1 : GAVEVPGMPPVPAALLPTPLLKK-GPNYAWTVYHGRRIMEAVGIIVHTVDELEPPVLAAI : 227
RLN34493.1 : GTVDVPGLPPVPSSSLPKPVMDKKNPNYTWFVYHGRRFAEADGIIVNTAAELERSALAAI : 283
RLM86688.1 : GAVDVPGLPPVPPSSLPKPVMDKKNPNYTWFVYHGRRFAEADGIIVNTAAELERSALAAI : 225
XP_0156454 : GAVDLPGLPPVPAALLPTPVMKK-GCNYEWLVYHGSRFMEAAGIIVNTVAELEPAVLEAI : 242
BZ1_3 : GAVDLPGMPPVPARLMPTPVMTK-GPNFAWLVYHGTRFMEAAGIIVNTVAELEPYILAAI : 244
 aV 6PG6PPVPa 6P P66 4 N5 W VYHG R meA G6IV TvaeLE LaAI

 * 320 * 340 * 360
XP_0024378 : ADGLCVPG-RRAPTVYPIGPVVMPVNKQPAAGGDGEQLQHECVRWLDAQPPASVVLLCFG : 287
XP_0024365 : ADGLCVPG-RRAPTVYPIGPVM-AFKPLAGDDDDEQLQLHECVRWLDAQPPASVVLLCFG : 286
XP_0345794 : AEGRCVPG-RRAPTVYLIGPALS---VKAPGK-----QPHECVTWLDAQPSASVVLLCFG : 275
XP_0049577 : AEGRCVPG-RRAPTVYLIGPALS---VKAPGK-----QPHECVTWLDAQPSASVVLLCFG : 275
XP_0258039 : ADG-------RFPTVYPIGPALSS--VKAPRE-----QPHRCVVWLDAQPPASVVLLCFG : 273
XP_0035601 : EGGRCVPGERRVPTVYPIGPVMS--FKKPTAK----EPPHECVRWLEAQPRASVVLLCFG : 278
RLM87236.1 : ADG-------RSPTVYPIGPALSS--VKAPRE-----QPHRCVAWLDAQPPASVVLLCFG : 273
RLN34493.1 : ADGRCTPG-VRPPTVYPIGPVIA---FEPPPE-----QPHECVRWLDAQPPASVALLCFG : 334
RLM86688.1 : ADGRCTPG-VRPPTVYPIGPVIS---FAPPSE-----QPHECVRWLDAQPPASVALLCFG : 276
XP_0156454 : ADGRCVPG-RRVPAIYTVGPVLS---FKTPPE-----KPHECVRWLDAQPRASVVFLCFG : 293
BZ1_3 : AGGLCVPG-RRAPTVYPIGPVV-P--VKPPGDGEQLQQLHECVRWLDAQPPASVVLLCFG : 300
 a G c pg R Pt6Y 6GP 6 HeCV WLdAQP ASVvlLCFG

 * 380 * 400 * 420
XP_0024378 : SMGGSFPSPQVREIADALEHSGHRFLWVLRGPIPADS--KYPTDADLDELLPEGFLERTK : 345
XP_0024365 : SMGGSFPSPQVREIADALEHSGHHFLWVLRGPLSPGS--KCPTDANVDELLPEGFLERTK : 344
XP_0345794 : SMGGSFPAPQVREIADALERSGHRFLWVLRGPVPAGG-APYPTDANVDELLPEGFLERTK : 334
XP_0049577 : SMGGSFPAPQVREIADALERSGHRFLWVLRGPVPAGG-APYPTDANVDELLPEGFLERTK : 334
XP_0258039 : SMGGSFPAPQVSEIADALERSGHRFLWVLRGPVPAGARSPYPSDANVDELLPEGFLERTK : 333
XP_0035601 : SMG-TFAPPQVLEIAEALDRSGHRFLWVLRGPPPGNS--PYPTDANLGELLPEGFLERTK : 335
RLM87236.1 : SMGGSFPAPQVSEIADALERSGHRFLWVLRGPVPAGARSPYPSDANVDELLPEGFLERTK : 333
RLN34493.1 : SQG-FFAAPQAHEIAHGLERSGHRFLWVLRGPPAPGA--RHPADANLAELLPDGFLERTK : 391
RLM86688.1 : SQG-FFAAPQAHEIAHGLERSGHRFLWVLRGPPAAGA--RHPTDANLAELLPDGFLERTK : 333
XP_0156454 : SMG-SFAPPQVLEIAAGLERSGHRFLWVLRGRPPAGS--PYPTDADADELLPEGFLERTK : 350
BZ1_3 : SMGGSFPSPQVREIANALDHSGHRFLWVLRGP-PADS--KYPTDANLDELLPEGFLERTK : 357
 SmG F PQv EIA Le SGHrFLWVLRGp P DA1 ELLPeGFLERTK

 * 440 * 460 * 480
XP_0024378 : GRGLVWPKWAPQKDILASPAVGGFVTHCGWNSILESLWHGVPLVPWPLFAEQHLNAFELV : 405
XP_0024365 : GRGLVWPKWAPQKDILANPAVGGFVTHCGWNSILESLWHGVPMAPWPLYAEQHLNAFELV : 404
XP_0345794 : DRGLVWPKWAPQKDIIAHPAVGGFVTHCGWNSVLESLWNGVPLAPWPLFAEQHLKAFELV : 394
XP_0049577 : DRGLVWPKWAPQKDIIAHPAVSGFVTHCGWNSVLESLWNGVPLAPWPLFAEQHLKAFELV : 394
XP_0258039 : DRGLVWPTWAPQKAIIAHAAVGGFVTHCGWNSVLESMWHGVPLAPWPQHADQHLNAFQLV : 393
XP_0035601 : EKGLVWPKWAPQQEILAHPAVGGFVTHCGWNSTLESLWHGVPLVPWPLYAEQHLNAFELV : 395
RLM87236.1 : DRGLVWPTWAPQKAIIAHAAVGGFVTHCGWNSVLESMWHGVPLAPWPQHADQHLNAFQLV : 393
RLN34493.1 : DRGLVWPTWAPQREILAHAAVGGFVTHGGWNSTLESLWHGVPLAPWPLYAEQHMNAFTLV : 451
RLM86688.1 : DRGLVWPTWAPQREILAHAAVGGFVTHGGWNSTLESLWFGVPLAPWPLYAEQHLNAFTLV : 393
XP_0156454 : GRGMVWPTWAPQKDILAHAAVGGFVTHGGWNSTLESLWHGVPMAPWPLYAEQHLNAFELV : 410
BZ1_3 : DRGLVWPKWAPQKDILANPAVGGFVTHCGWNSILESLWHGVPMVPWPLFAEQHLNAFELV : 417
 4G6VWP WAPQ I6A AVgGFVTH GWNS LES6W GVP6 PWPl AeQH6nAF LV

 * 500 * 520 * 540
XP_0024378 : SVMGVAVAMKVDRKRGNLVEAAELERVVRSLMGGSEEEGRKAREKATEAKDLCRKAVADG : 465
XP_0024365 : SVMGVAVAMQVDRKRGNFVEAAELERVVRCLMGGSEE-GRKAGKKAAEAKALCRKAVADG : 463
XP_0345794 : SVMGVAVAMEVDRKRGNFVEAAELERAVRSLMGGS-EEGRKARVKAAEAKALCRNAVEEG : 453
XP_0049577 : SVMGVAVAMEVDRKRGNFVEAAELERAVRSLMGGS-EEGRKARVKAAEAKALCRNAVEEG : 453
XP_0258039 : SMAGVAVAMEVDRKRGNFVEAAELERAVRSLMGGESEEGRKAREKAAEAKALFRRAVEEG : 453
XP_0035601 : SVMGVAVAMAVDTKRDNFVEATELERALRSLMDDGSEEGSKAREKAMEAQALCRSAVEEG : 455
RLM87236.1 : SVAGVAVAMEVDRKRGNFVEAAELERAVRSLWAASRRRGGRRGRRPP------RRRPCSG : 447
RLN34493.1 : AAMGVAVAMKVDRKRDNFVEAAELEREVRELMGGG-EEGRKAREKAMEMKAACRNAVEEG : 510
RLM86688.1 : AAMGVAVAMRVDRKRDNFVEAAELERAVKELMGGG-EEGRKAREKAMEMKAACRNAVEGG : 452
XP_0156454 : RDMGVAVEMEVDRKRGNLVEAAELERAVRCLMDEGSEEGRMAREKAAAAKAACRNAVDGG : 470
BZ1_3 : SVMGVAVAMQVDRKRDNFVEAAELERVVRSLMGGSEEEGRKAREKATEAKALCRKAVADG : 477
 mGVAVaM VDrKR NfVEAaELER 64 Lm eeGr ar 4a e ka cR av G

 * 560 * 580
XP_0024378 : GSSAASLQELAREIGEHREHQGRTEATPPLPQQVRYAAATLGAARHG : 512
XP_0024365 : GSSEASLQKLAREILHNHNDKWCGVA--------------------- : 489
XP_0345794 : GSSYVSLQELAREMLQHCGREAEDSASL------------------- : 481
XP_0049577 : GSSYVSLQELAREMLQHCGREAEDSASL------------------- : 481
XP_0258039 : GSSDVAMKKLAREMLEHRGCEAEASATASLVR--------------- : 485
XP_0035601 : GSSYTAWHKLAREVSRD------------------------------ : 472
RLM87236.1 : APWRRAVRRTWR----------------------------------- : 459
RLN34493.1 : GSSDAALHRLAEELCKGAVVSTGK----------------------- : 534
RLM86688.1 : GSSDAALLRLAEELYKGAVVSTTK----------------------- : 476
XP_0156454 : GSSIAALRKLTQEMAHMSSI--------------------------- : 490
BZ1_3 : GSSEVSLQQLAREIGEHREYQARTTEATPLPQHVHYPAATLGAASHG : 524
 gss la e

### (F). Molecular Phylogenetic analysis by Maximum Likelihood method (ScBZ1_3)


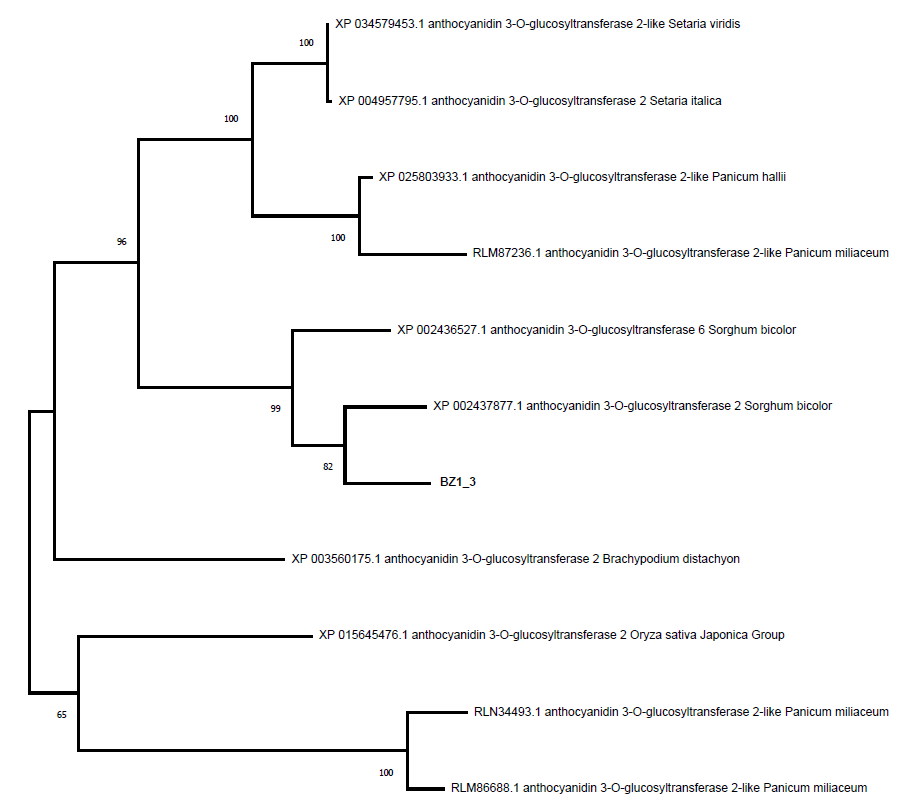


### (H). Multiple sequence alignment of ScBZ1_5 gene.

* 20 * 40 * 60
XP_0024480 : ----------------------------------------------------------MA : 2
XP_0345790 : ----------------------------------------------------------MA : 2
XP_0049554 : ----------------------------------------------------------MA : 2
XP_0024607 : ----------------------------------------------------------MA : 2
NP_0011484 : ----------------------------------------------------------MA : 2
ACG31601.1 : ----------------------------------------------------------MA : 2
XP_0024607 : ----------------------------------------------------------MA : 2
RLN34493.1 : MLLNQRFTLILVYHSKIHFKCNISFATHRGWKINTPPLSCFPRIHDDAGGLNPHPSMATA : 60
RLM86688.1 : ----------------------------------------------------------MA : 2
XP_0257998 : ----------------------------------------------------------MA : 2
BZ1_5 : ----------------------------------------------------------MA : 2
 mA

 * 80 * 100 * 120
XP_0024480 : TPTVVLLPVWGAGHLMPMVEAGKRLLAS--GSRRALSVTVLVMRPPTEQHASELEGDIRR : 60
XP_0345790 : TPTVVLLPVWGAGHLMPMLEAGKRLLAS--SGGRALSVTVLVMRPPTEQQASEVEGHIRR : 60
XP_0049554 : TPTVVLLPVWGAGHLMPMLEAGKRLLAS--SGGRALSVTVLVMRPPTEQQASEVEGHIRR : 60
XP_0024607 : APTVVLLPVWGAGHLMPMLEAGKRLLTR---GGRALSLTVLLMPAPTEEVAAELDGHVRR : 59
NP_0011484 : APTVVLLPVWGAGHLMSMLDAGKRLLTR---GGRALSLTVLVMRAPTEQLAADLDAHIRR : 59
ACG31601.1 : APTVVLLPVWGAGHLMSMLDAGKRLLTR---GGRALSLTVLVMRAPTEQLAADLDAHIRR : 59
XP_0024607 : APTVVLLPVWGAGHLMPMLEAGKRLLTH---SGRALSLTVLLMPAPTEEVAAELDGHVRR : 59
RLN34493.1 : SPTLVLLPLWSAGHLMSLLEAGKRLLTR---AGGKLSLTVLVIPPPTEHLAAEVQGHIRR : 117
RLM86688.1 : TPTVVLLPLWSAGHLMSLLEAGKRLLAR---AGGRLSLTVLVIPPPTEKLAAEVEGHIRR : 59
XP_0257998 : TPTVVLLPVWGAGHLTPMLEAGKRLLASGAGGGRALSVTVLVMRPPTEQQASEIEGHIRR : 62
BZ1_5 : TPTVVLVPVWGAGHLMPMLEAGKRLLAT--NGGRALSVTVLVMRPPTEQHASELETHIRR : 60
 PT6VL6P6WgAGHLm 66eAGKRLL graLS6TVL66 PTE A e6 h6RR

 * 140 * 160 * 180
XP_0024480 : AEE--AAAGLDVRFHRLPAVKPPTDHAGPVEFISRVVQLHAPHVRAAVSSLS--CPVAAL : 116
XP_0345790 : AEE--AAAGLDVRFHRLPAVEPPTDHEGPVEFISRTVELHEPHVRAAISGLLPASPVAAL : 118
XP_0049554 : AEE--AAAGLDVRFHRLPAVEPPTDHEGPVEFISRTVELHEPHVRAAISGLLPASPVAAL : 118
XP_0024607 : -EE---ASGLDIRFVRLPAVEPPMDSRGIEEFVSRLVQLHAPHVRAAMSSLA--SPVAAF : 113
NP_0011484 : -EE---ASGLDVRFVRLPAVQPPTDFHGIEEFISRLVQLHAPHVRAAISSLA--SPVAAV : 113
ACG31601.1 : -EE---ASGLDVRFVRLPAVQPPTHFHGIEEFISRLVQLHAPHVRAAISSLA--SPVAAV : 113
XP_0024607 : -EE---ASGLDIRFVRLPAVEPPMDSRGIEEFVSRLVQLHAPHVRAAMSSLS--SPVAAF : 113
RLN34493.1 : -EE---ASGLDVRFVHLPAMDPPTDFRGIEEFLSRFVQMHAPRVRAAVADLP--CPVAAL : 171
RLM86688.1 : -EE---ASGLDVRFVRLPAVEPPTDFRGIEEFLSRLVQMHAPDVRAAVAALP--CPMAAL : 113
XP_0257998 : AEEEAAAAGLDVRFRRLPAVEPPTDHEGPVEFISRAVELHVPHVRAAVSGLP---SVAAL : 119
BZ1_5 : AQE--AAGGLDVRFHRLPAVEPPTDSAGPVEFISRVVQLHAPHVRAAVSSLA--CPVAAL : 116
 2E A GLD6RF rLPA6 PPtd G EF6SR V26H PhVRAA6s L p6AA

 * 200 * 220 * 240
XP_0024480 : VLDLFCTPFVDVARELAVPAYVYFTCNAAALSFFLRLPALCEEVAGEFGEMDGAADIPGL : 176
XP_0345790 : VLDLFCTPFIDVARELAVPAYIYFTCNAAALSFFLRLPALCEEVAGEFEDMDGAADIPGL : 178
XP_0049554 : VLDLFCTPFIDVARELAVPAYIYFTCNAAALSFFLRLPALCEEVAGEFEDMDGAADIPGL : 178
XP_0024607 : VIDFFCTTLLDVSRELAVPAYVYFTASAGMLALKLRLPSLHEEVTVQFEEMEGAVDVPGL : 173
NP_0011484 : VMDFFCTALLDVTRELAVPAYVYFTASAGMLAFFLRLPSLHEEVTVQFEEMEGAVDVPGL : 173
ACG31601.1 : VMDFFCTALLDVTRELAVPAYVYFTASAGMLAFFLRLPSLHEEVTVQFEEMEGAVDVPGL : 173
XP_0024607 : VIDFFCTTLLDVSRELAVPAYVYFTASAGMLALFLRLPSLHEEVTVQFEEMEGAVDVPGL : 173
RLN34493.1 : VLDFFCTALIDVARDLDVPAYVYFTTNAAMMALMLRLPALHEEVTVEFEEMEGTVDVPGL : 231
RLM86688.1 : VLDFFCTALIDVARDLAVPAYVYFTTNAAMLALMLRLPALHEEVTVEFEEMEGAVDVPGL : 173
XP_0257998 : VLDLFCTPLIDVARELAVPAYVYFTCNAATLSFFLRLPALLEEVAGEFEEMEGAADIPGL : 179
BZ1_5 : VLDLFCTPFVDVARELSVPAYVYFTCNAAALSFFLRLPALCEEVAGEFGEMDGAADIPGL : 176
 V6D FCT 6DV ReLaVPAY6YFT A 6 LRLP L EEV 2FeeM Ga D6PGL

 * 260 * 280 * 300
XP_0024480 : PPVPALSLPTPIMERKKPDCAWYACHGRRFMDADGIIVNTAAELEQGVLSAIAKGRCTRG : 236
XP_0345790 : PPVPPLSLPTPVMERTKPECAWYAYHGRRFADADGIVVNTAAELEQGVLSAIADGRCAR- : 237
XP_0049554 : PPVPPLSLPTPVMERTKPECAWYAYHGRRFADADGIVVNTAAELEQGVLSAIADGRCAR- : 237
XP_0024607 : PPVPPSSLPNPVMDKNHPNYTWFAYHGRRFAEADGIIVNTAAELEQSVLSAIADGRCTP- : 232
NP_0011484 : PPVPPSSLPVPVMDKNHPNYTWFMYHGRRFAEADGIIVNTAAELEQSVLAAIADGRCTP- : 232
ACG31601.1 : PPVPPSSLPVPVMDKNHPNYTWFMYHGRRFAEADGIIVNTAAELEQSVLAAIADGRCTP- : 232
XP_0024607 : PPVPPSSLPDPLMDKNHPNCTWFAYHGRRFVEADGIIVNTAAEIEQSVLAAIADGRCTR- : 232
RLN34493.1 : PPVPSSSLPKPVMDKKNPNYTWFVYHGRRFAEADGIIVNTAAELERSALAAIADGRCTP- : 290
RLM86688.1 : PPVPPSSLPKPVMDKKNPNYTWFVYHGRRFAEADGIIVNTAAELERSALAAIADGRCTP- : 232
XP_0257998 : PPVPPLWLPMPVMERARPECAWYAYHGRRFADADGIIVNTATELDRGALSAIAGGRCTR- : 238
BZ1_5 : PPVPPLSLPTPIMERKKPECTWYAYHGRRFMDADGIIVNTAAELEQSVLAAIADGRCTP- : 235
 PPVPp sLP P6M 4 P W5 yHGRRF ADGI6VNTAaE6e L AIAdGRCt

 * 320 * 340 * 360
XP_0024480 : TGSRPAPTLYPIGPVISFPPPAA--EPPHECLRWLESQPPASVVFLCFGSGGFFTAPRAH : 294
XP_0345790 : -GGRPPPTLYPIGPVISFPPPTA--PPPHDCVRWLETQPPASVAFLCFGSGGFFTAAQAH : 294
XP_0049554 : -GGRPPPTLYPIGPVISFPPPTA--PPPHDCVRWLETQPPASVAFLCFGSGGFFTAAQAH : 294
XP_0024607 : --GVRAPTVYPIGPVISFTPPAE--E-PHECVRWLDTQPVASVVLLCFGSVGFFTAPQAH : 287
NP_0011484 : --GVRAPTVYPIGPVISFSPPPTNTEHPHECVRWLDTQPAASVVLLCFGSQGFSAAPQAH : 290
ACG31601.1 : --GVRAPTVYPIGPVISFSPPPTNTEHPHECVRWLDTQPAASVVLLCFGSQGFSAAPQAH : 290
XP_0024607 : --GARAPTLYPIGPVISFIPPTE--RRPDECVQWLDTQPPASVVLLCFGSGGFFTAPQAH : 288
RLN34493.1 : --GVRPPTVYPIGPVIAFEPPPE---QPHECVRWLDAQPPASVALLCFGSQGFFAAPQAH : 345
RLM86688.1 : --GVRPPTVYPIGPVISFAPPSE---QPHECVRWLDAQPPASVALLCFGSQGFFAAPQAH : 287
XP_0257998 : -GG-PAPALYPIGPVISFPPP-A--EPPHDCVRWLEAQPPASVVFLCFGSGGFFTAAQAH : 293
BZ1_5 : --GVRAPTVYPIGPVISFSPR-T--EQSHECVRWLDTQPPASVVLLCFGSGGFFTAPQAH : 290
 g Pt6YPIGPVIsF Pp ph C6rWL QP ASV LCFGS GFf A qAH

 * 380 * 400 * 420
XP_0024480 : EAAHGLERSGHRFLWVLRGAPAPDTRSPTDADLAELLPEGFVERTRNRGLVWPTAVPQKE : 354
XP_0345790 : EVAGGLERSGHRFLWVLRGPPAPGSRQPTDADLAELLPEGFVERTRGRGLVWPTAVPQKE : 354
XP_0049554 : EVAGGLERSGHRFLWVLRGPPAPGSRQPTDADLAELLPEGFVERTRGRGLVWPTAVPQKE : 354
XP_0024607 : ELAHGLERSGHRFLWVLRGPPAPGERHPSDANLDELLPDGFLERTKGRGLVWPTKAPQKE : 347
NP_0011484 : EIAHGLERSGHRFLWVLRGPPAPGERHPSDANLSELLPDGFLERTKGRGLVWPTKAPQKE : 350
ACG31601.1 : EIAHGLERSGHRFLWVLRGPPAPGERHPSDANLSELLPDGFLERTKGRGLVWPTKAPQKE : 350
XP_0024607 : EIAHGLERSGHRFLWVLRGPPAPGERLPSDANVAELLPDGFLERTNGRGLVWPTKAPQKE : 348
RLN34493.1 : EIAHGLERSGHRFLWVLRGPPAPGARHPADANLAELLPDGFLERTKDRGLVWPTWAPQRE : 405
RLM86688.1 : EIAHGLERSGHRFLWVLRGPPAAGARHPTDANLAELLPDGFLERTKDRGLVWPTWAPQRE : 347
XP_0257998 : EVARGLERSGHRFLWVLRGPPAPGSRQPTDANLAELLPDGFLERTRGRGLVWPTAAPQKE : 353
BZ1_5 : EIAHGLERSGHRFLWVLRGPPAPGQRHPSDANLAELLPDGFLERTKGRGLVWPTKAPQKE : 350
 E A GLERSGHRFLWVLRGpPApg R P DA16 ELLP GF6ERT RGLVWPT PQ4E

 * 440 * 460 * 480
XP_0024480 : ILAHAAVGGFVTHCGWNSVLESLWFGVPMAPWPLYAEQHLNAFALVAAMGVAVAMEVDRK : 414
XP_0345790 : ILAHAAVGGFVTHCGWNSMLESLWFGVPMAAWPLYAEQHLNAFALVAAMGAAVAVEVDRA : 414
XP_0049554 : ILAHAAVGGFVTHCGWNSMLESLWFGAPMAAWPLYAEQHLNAFALVAAMGAAVAVEVDRA : 414
XP_0024607 : VLAHAAMGGFVTHGGWNSVLESLWFGVPMAPWPLYAEQHLNAFTLVAYMGVAVAMKVDRK : 407
NP_0011484 : ILAHAAVGGFVTHGGWNSVLESLWFGVPMAPWPLYAEQHLNAFTLVAYVGVAVAMKVDRK : 410
ACG31601.1 : ILAHAAVGGFVTHGGWNSVLESLWFGVPMAPWPLYAEQHLNAFTLVAYVGVAVAMKVDRK : 410
XP_0024607 : ILAHAAMGGFVTHGGWNSVLESLWFGVPMAPWPLYAEQHLNAFTLVAYMGVAVAMKVDRK : 408
RLN34493.1 : ILAHAAVGGFVTHGGWNSTLESLWHGVPLAPWPLYAEQHMNAFTLVAAMGVAVAMKVDRK : 465
RLM86688.1 : ILAHAAVGGFVTHGGWNSTLESLWFGVPLAPWPLYAEQHLNAFTLVAAMGVAVAMRVDRK : 407
XP_0257998 : ILARAAVGGFVTHCGWNSILESLWCGVPMAPWPLYAEQHLNAFALVAAAGAAVAMGVDRR : 413
BZ1_5 : ILAHAAMGGFVTHGGWNSVLESLLFGVPMATWPLYAEQRENAVMLCEETRAALRPQA-RG : 409
 6LAhAA6GGFVTH GWNS LESLwfGvP6A WPLYAEQh NAf Lva g A6a vdR

 * 500 * 520 * 540
XP_0024480 : RDNFVEAAELERAVKALMGDGEE-GRKAREKAAVVQAACRNAVDDGGSSTATLSKLCNDI : 473
XP_0345790 : RGNFVEAAEVERAVRELMGGGEEAGRRVREKAAEMKAACRNAVGDGGSSTATLKRLANDI : 474
XP_0049554 : RGNFVEAAEVERAVRELMGGGEEAGRRVREKAAEMKAACRNAVGDGGSSTATLKRLANDI : 474
XP_0024607 : RNNFVEAAELERAVKELMGGGEE-GRKAREKAMEMKAAFRNAVEEGGSSYAALRRLSEEI : 466
NP_0011484 : RNNFVEASELERAVKELMGGGEE-GRKAREKAMEMRDACRNAVEEGGSSYSSLRRLSEKI : 469
ACG31601.1 : RNNFVEASELERAVKELMGGGEE-GRKAREKAMEMRDACRNAVEEGGSSYSSLRRLSEKI : 469
XP_0024607 : RNNFVDAAELERAVKELMGGGEE-GRKAREKAMEMKAAFRNAVEEGGSSYAALWRLSEEI : 467
RLN34493.1 : RDNFVEAAELEREVRELMGGGEE-GRKAREKAMEMKAACRNAVEEGGSSDAALHRLAEEL : 524
RLM86688.1 : RDNFVEAAELERAVKELMGGGEE-GRKAREKAMEMKAACRNAVEGGGSSDAALLRLAEEL : 466
XP_0257998 : RGNFVEAAELERAVRALMGGGEE-GRRARERAAEMKAACRNAVEDGGSSAAALKRLSNDI : 472
BZ1_5 : ADGMILAEDVAEVVRDMMHGDK--GAAARAKVAELREAAASALRPGGVSYETLAQVMSKW : 467
 r nf6eA e6eraV4 6Mgggee Gr aRe4a e6 A rnA6 GGsS L 6

 *
XP_0024480 : ---CRQ--------- : 476
XP_0345790 : ---CQQLRRDPAK-- : 484
XP_0049554 : ---CQQLRRDPAK-- : 484
XP_0024607 : M--CKVDTNQ----- : 474
NP_0011484 : ---CKVDKNL----- : 476
ACG31601.1 : ---CKVDKNL----- : 476
XP_0024607 : M--CKVDTNH----- : 475
RLN34493.1 : CKGAVVSTGK----- : 534
RLM86688.1 : YKGAVVSTTK----- : 476
XP_0257998 : L--CRQLPSSSLGTS : 485
BZ1_5 : K--GDTY-------- : 472

### (I). Molecular Phylogenetic analysis by Maximum Likelihood method (ScBZ1_5)


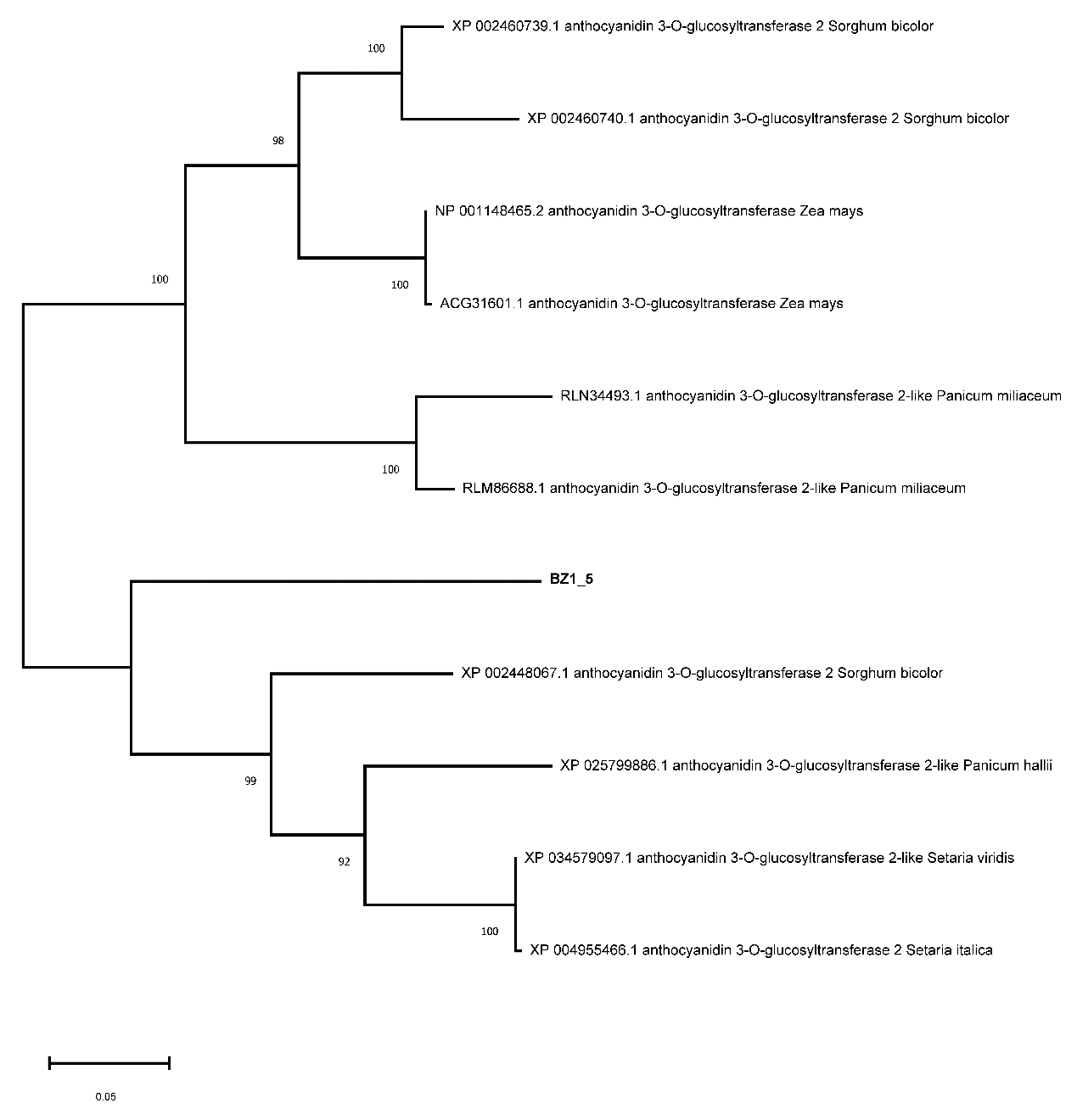


## Fig S15. Sequence alignment of Anthocyanidin 5,3-O-glucosyltransferase (GT1) proteins from sugarcane and various other plants, and phylogenetic relationships of Anthocyanidin 5,3-O-glucosyltransferase.

### (A). Multiple sequence alignment of ScGT1_1 gene.

* 20 * 40 * 60
XP_0024607 : ----------------------------------------------------------MA : 2
XP_0049577 : ----------------------------------------------------------MA : 2
XP_0345786 : ----------------------------------------------------------MA : 2
XP_0345787 : --------------------------------------------MAIISTPSSQSGEPAA : 16
XP_0049577 : --------------------------------------------MAIISTPSSQSGEPAA : 16
XP_0024449 : ---------------------------------------------------MTTSADADA : 9
XP_0147525 : ----------------------------------------------------------MA : 2
RLN34493.1 : MLLNQRFTLILVYHSKIHFKCNISFATHRGWKINTPPLSCFPRIHDDAGGLNPHPSMATA : 60
EMS62308.1 : ----------------------------------------------------------MA : 2
XP_0024607 : ----------------------------------------------------------MA : 2
ScGT1_1 : ----------------------------------------------------------MA : 2
 A

 * 80 * 100 * 120
XP_0024607 : TPTVVLVPVWGIGHFVPMLEVGKRLLARSARALTITVLVMPEPDATRASEITEHIRQEQA : 62
XP_0049577 : TPTVVLVPVWGIGHFVPMLEAGKRLLARSARPLTITVLVMPEPMSKRASEIAEHIRQEEA : 62
XP_0345786 : TPTVVLVPVWGIGHFVPMLEAGKRLLARSARPLTITVLVMPEPMSKRASEIAEHIRQEEA : 62
XP_0345787 : KPTVVLVALWGVSHFAPMVEAGKRLLDLSGRSLTVNVVLVPSPTGKWEDAIFERIRREEA : 76
XP_0049577 : KPTVVLVALWGVSHFAPMVEAGKRLLDLSGRSLTVNVVLVPSPTGKWEDAIFERIRREEA : 76
XP_0024449 : DPTVVLVPIWGVGHFVPMIEAGKRLLARSTRRLTLTVLIMPAPSEKRASEIADHIREVEA : 69
XP_0147525 : TPTVVLLPVWGAGHLMSMLDAGKRLLARSRGALSLTVLVMQAPAENYRSEVAGHIRREEA : 62
RLN34493.1 : SPTLVLLPLWSAGHLMSLLEAGKRLLTRAGGKLSLTVLVIPPPTEHLAAEVQGHIRREEA : 120
EMS62308.1 : NPTIVLLPVWGAGHFMPMIQAGKQLLASSSRPLSLTVLLLPAPTAQAASDICEHVRREEA : 62
XP_0024607 : APTVVLLPVWGAGHLMPMLEAGKRLLTRGGRALSLTVLLMPAPTEEVAAELDGHVRREEA : 62
ScGT1_1 : TPTVVLVPVWGIGHFMPMLEVGKRLLARSARPLTITVLVMPEPEATRASEITEHIRQEEA : 62
 PT6VL6p6Wg gH p66 aGKrLL s r L36tV666p P 6 h6R e2A

 * 140 * 160 * 180
XP_0024607 : TGL----AIRFHHLPLVAPP------TDTSGIEEYVSRYVQLYSPHVKAAVAGLTCPVAG : 112
XP_0049577 : SGLG---IIRFHHLPAVDPP------TDHSGIEEYISRYAQLYAPQVKAAVAALTCPVAG : 113
XP_0345786 : SGLG---IIRFHHLPAVDPP------TDHSGIEEYISRYAQLYAPQVKAAVAALTCPVAG : 113
XP_0345787 : SGQ----GIRFHHPAAVEPP------TDHTGIEEFVSRYVQLYVADLKAIVSGLACPVAA : 126
XP_0049577 : SGQ----GIRFHHPAAVEPP------TDHTGIEEFVSRYVQLYVADLKAIVSGLACPVAA : 126
XP_0024449 : ESAALGLAIRFHHLPAAAEPPPAVATTYYSGPEEFISLNVHPYVPHVTAAVAGLTCPVAA : 129
XP_0147525 : SG----LDIRFHHLPTVELP------TDYVGIEEFISRFVQLHAPHVKAAISGLACPVAA : 112
RLN34493.1 : SG----LDVRFVHLPAMDPP------TDFRGIEEFLSRFVQMHAPRVRAAVADLPCPVAA : 170
EMS62308.1 : AGA---VDIRFHHLPDVKLP------TDHSGIEEFISRAVQLHVPHLRAAIIGLTCPVAA : 113
XP_0024607 : SG----LDIRFVRLPAVEPP------MDSRGIEEFVSRLVQLHAPHVRAAMSSLASPVAA : 112
ScGT1_1 : SGL----AIRFHHLPLVAPP------TDTSGIEEYVSRYVQLYSPHVKAAVAGLTCPVAG : 112
 g 6RFhhlp P td GiEE56Sr vq p 6 Aa6 L cPVA

 * 200 * 220 * 240
XP_0024607 : VVVDIFCTTLFDAAHELGVPAYVYLIASAAMCALLLRSPALHEEVAG--DVEFEDVEGGG : 170
XP_0049577 : VVVDIFCTTLFDAAHELRVPAYVYLITSAAMCALLLRSPALDEELEA--EVEFEESEEGG : 171
XP_0345786 : VVVDIFCTTLFDAVHELRVPAYVYLITSAAMCALLLRSPALDEELEA--EVEFEESEEGG : 171
XP_0345787 : VVVDIFCTTLLDAPLELGVPAYVFLTCGAAMAAVLLRSPSLDEEVAA--EVEFRELEGG- : 183
XP_0049577 : VVVDIFCTTLLDAPLELGVPAYVFLTCGAAMAAVLLRSPSLDEEVAA--EVEFRELEGG- : 183
XP_0024449 : VVVDIFCTPLLDAAHGLAVPAYVYLICSAAMCALLLHSPALDDEAAAAGDVEFEEMDGGV : 189
XP_0147525 : LVVDFFATTLFDVSRELAVPAYVYFTASAAAYALFLRLPALQEEVTC----EFEELDGM- : 167
RLN34493.1 : LVLDFFCTALIDVARDLDVPAYVYFTTNAAMMALMLRLPALHEEVTV----EFEEMEGT- : 225
EMS62308.1 : LVVDIFCTPALDVSGELAVPSYVYFTSSAAMLSLLLRSPALDDEVVV----EFEEMGGA- : 168
XP_0024607 : FVIDFFCTTLLDVSRELAVPAYVYFTASAGMLALKLRLPSLHEEVTV----QFEEMEGA- : 167
ScGT1_1 : VVVDIFCTTLFDAAHELGVPAYVYLISSAAMCALLLRSPALDEEVAG--DVEFEEVEGG- : 169
 V6D FcT l D eL VPaYV5 Aam a6 Lr P L eE 2Fee g

 * 260 * 280 * 300
XP_0024607 : VDVPGLPPVPASCLPTGLENRKITTYRWFLYNGRRYMEAGGIVLNTVAEAEPRVLAAIAD : 230
XP_0049577 : VDVPGLPPVPASCLPTGLENRKIPTYKWFVYNGRRYMEASGIVVNTVAELEPRVLGAIAD : 231
XP_0345786 : VDVPGLPPVPASCLPTGLENRKIPTYKWFVYNGRRYMEASGIVVNTVAELEPRVLGAIAD : 231
XP_0345787 : VDVPGLPPVPATNLPSGLDNRKIRTYKWFLYNGRRYLETNGILINTIAELEESVLAAITD : 243
XP_0049577 : VDVPGLPPVPATNLPSGLDNRKIRTYKWFLYNGRRYLETNGILINTIAELEESVLAAITD : 243
XP_0024449 : VHVPGLPPVPASCLPSGLVDRNVPTYRWFLYNGRRYTEAAGIIVNTVAELEPHVLAAIAD : 249
XP_0147525 : VDVPGLPPVPPSSLPSPLMDKKNPNYTWFVYHGRRFMEANGVMINTAAALEQSVLAAIAD : 227
RLN34493.1 : VDVPGLPPVPSSSLPKPVMDKKNPNYTWFVYHGRRFAEADGIIVNTAAELERSALAAIAD : 285
EMS62308.1 : LDLPGLPPVPPSVLPGTLLERKSPTYKWFVYTGRRYVEASGIIVNTADDLEPRVLAAIAE : 228
XP_0024607 : VDVPGLPPVPPSSLPNPVMDKNHPNYTWFAYHGRRFAEADGIIVNTAAELEQSVLSAIAD : 227
ScGT1_1 : VDVPGLPPVPASCLPTGLENRKITTYNWFLYNGRRYMEAGGIVLNTVAEAEPRVLAAIAD : 229
 6d6PGLPPVP 3 LP 6 4k Y WF Y GRR5 Ea G666NT aelE vL AIad

 * 320 * 340 * 360
XP_0024607 : G-RCTRGVPAPPVYSIGPVIP----FTPPAAAG---EQARHECVRWLDSQPPGSVVFLCF : 282
XP_0049577 : G-QCTRGTRAPSVYTIGPVIP----FTPSTGE----KAAAHECVRWLDSQPPASVVFLCF : 282
XP_0345786 : G-QCTRGTRAPSVYTIGPVIP----FTPSTGE----KAAAHECVRWLDSQPPASVVFLCF : 282
XP_0345787 : G-RCTRGIPTPPVYTIGPVVA----IAPPSEE-------KDECVRWLDSQPAASVLFLCF : 291
XP_0049577 : G-RCTRGIPTPPVYTIGPVVA----IAPPSEE-------KDECVRWLDSQPAASVLFLCF : 291
XP_0024449 : GRCCTRGNRAPTVYTIGPVLAATITTTPPPADAEKQQEEEHECVRWLDTQPPASVLFLCF : 309
XP_0147525 : G-RCTPGIPAPTVYPVGPVIS----FNPPA------EQGGHECLRWLDTQPPASVVLLCF : 276
RLN34493.1 : G-RCTPGVRPPTVYPIGPVIA----FEPPP------EQP-HECVRWLDAQPPASVALLCF : 333
EMS62308.1 : G-RCTRGARAPVVYPIGPVLA----LTPPAHAEE--EEQTHECVRWLDAQPPASVLFLCF : 281
XP_0024607 : G-RCTPGVRAPTVYPIGPVIS----FTPPA------EEP-HECVRWLDTQPVASVVLLCF : 275
ScGT1_1 : G-WCTRGVPAPPVYSIGPVIP----FTPPAGE----QQARHECVRWLDSQPPGSVVFLCF : 280
 G CT G P VY 6GPV6 Pp hEC6RWLD QP aSV LCF

 * 380 * 400 * 420
XP_0024607 : GGKG-CFTAPQAHEIAHGLDRSGRRFLWVLRGLPEP---GTKMPADGNLAELLPAGFLER : 338
XP_0049577 : GGTG-SFTAPQAHEIAHGLERSGHRFLWVLRGQPEP---GTKLPTDGNLVELLPAGFLEE : 338
XP_0345786 : GGTG-SFTAPQAHEIAHGLERSGHRFLWVLRGQPEP---GTKLPTDGNLVELLPAGFLEE : 338
XP_0345787 : GGGGGYFSVPQVHEIAQGLERSGHRFLWVLRGQPEP---GMKVPTDANLAELLPDGFLER : 348
XP_0049577 : GGGGGYFSVPQVHEIAQGLERSGHHFLWVLRGQPEP---GMKVPTDANLAELLPDGFLER : 348
XP_0024449 : GSAR-FFSARQAHEAAHALDRSGHRFLWVLRGPPEH---GTKLSSDGDLAELLPPGFLER : 365
XP_0147525 : GSGG-FSTAPQAHEIAHGLERSGHRFLWVLRGPPAA---GAQQPADANLEELLPEGFLER : 332
RLN34493.1 : GSQG-FFAAPQAHEIAHGLERSGHRFLWVLRGPPAP---GARHPADANLAELLPDGFLER : 389
EMS62308.1 : GSKG-FLTKPQAHEIARGLERSGHRFLWVLRGLPADTSHGARHPSDGDLAELLPEGFLGE : 340
XP_0024607 : GSVG-FFTAPQAHELAHGLERSGHRFLWVLRGPPAP---GERHPSDANLDELLPDGFLER : 331
ScGT1_1 : GGKG-CFTAPQAHEIAHGLDRSGHRFLWVLRGPPEP---GMKMPTDGNLAELLPAGFLER : 336
 G g f pQaHE A gL RSGhrFLWVLRG P G p D 1L ELLP GFLe

 * 440 * 460 * 480
XP_0024607 : TKDRGLVWPTKAPQKEILAHAAVGGFVTHGGWNSILESLWHGVPMVPWPLGAEQHYNAFT : 398
XP_0049577 : TKGRGLVWPAKAPQKEILAHAAVGGFVTHCGWNSILESLWHGVPMVPWPLGAEQHYNAFT : 398
XP_0345786 : TKGRGLVWPAKAPQKEILAHAAVGGFVTHCGWNSILESLWHGVPMVPWPLGAEQHYNAFT : 398
XP_0345787 : TKGRGLVWPTRAPQKEILAHAAVGGFVTHCGWNSSVESLWFGVPTLPWPQAAEQHLNAFV : 408
XP_0049577 : TKGRGLVWPTRAPQKEILAHAAVGGFVTHCGWNSSVESLWFGVPTLPWPQAAEQHLNAFV : 408
XP_0024449 : TKGRGLVWPKWAPQKEILAHAAVGGFVTHCGWNSVLESLWFGVPMLPWPWAAEQHYNAFT : 425
XP_0147525 : TKGKGLVWPTKAPQKEILAHAAVGGFVTHGGWNSVLESLWFGVPMVPWPLYAEQHFNAFT : 392
RLN34493.1 : TKDRGLVWPTWAPQREILAHAAVGGFVTHGGWNSTLESLWHGVPLAPWPLYAEQHMNAFT : 449
EMS62308.1 : TEERGLVWPKRAPQKEILAHASVGGFVTHCGWNSVLESLWFGVPMLPWPLGAEQHFNAFT : 400
XP_0024607 : TKGRGLVWPTKAPQKEVLAHAAMGGFVTHGGWNSVLESLWFGVPMAPWPLYAEQHLNAFT : 391
ScGT1_1 : TEDRGFVWPTKAPQKEILAHAAVGGFVTHGGWNSVLESLWHGVPMVPWPLGAEQHYNAFT : 396
 Tk 4GlVWP APQ4E6LAHAa6GGFVTH GWNS 6ESLW GVP PWP AEQH NAFt

 * 500 * 520 * 540
XP_0024607 : LVADMGVAVAMGVERKRSNFVAAAELERAVKALMGDG-ETVRKVRDKVTEMKAACRKAVE : 457
XP_0049577 : LVADMGVAVPMEVDRKRNNFVGAGELERALKALMDDGGEAARKVRERAMEMKAACRKAVE : 458
XP_0345786 : LVADMGVAVPMEVDRKRNNFVGAGELERALKALMDDGGEAARKVRERAMEMKAACRKAVE : 458
XP_0345787 : LVAEMGVAVGMKVDKERRNFVEAAELERAVKALMGDG-EVARKIREKSAEMKAVCRKAVE : 467
XP_0049577 : LVAEMGVAVGMKVDKERRNFVEAAELERAVKALMGDG-EVARKIREKSAEMKAVCRKAVE : 467
XP_0024449 : LVAGMGVAVAMEVCRKEDNFVEAAELERAVRALMG-G-AEGTAAREKAREMKAACRRAVE : 483
XP_0147525 : LVAYMGVAVAMEVDRKRKNFVRASDLERAVVALMGDS-DEGRKAREKATEMKAACRSAVE : 451
RLN34493.1 : LVAAMGVAVAMKVDRKRDNFVEAAELEREVRELMGGG-EEGRKAREKAMEMKAACRNAVE : 508
EMS62308.1 : LVCDMGVAVPLTVDRKRDNFVEAAELERAVRSLMGGG-EEGKKAKEKAMEMMVVCRKAVD : 459
XP_0024607 : LVAYMGVAVAMKVDRKRNNFVEAAELERAVKELMGGG-EEGRKAREKAMEMKAAFRNAVE : 450
ScGT1_1 : LVADMGVAVAMDVERKRNNFVAAAELERAVKALMGDG-ETARKVRDKVMEMKAACRKAVE : 455
 LVa MGVAV 6 V 4kr NFV A eLERa6 LMg g e k 4e4 EMka cR AVe

 * 560 *
XP_0024607 : EGGSSNVSLQRLCDALVKGAVHPRK------ : 482
XP_0049577 : EGGSSSVELQRLCEALVVGAVLPTK------ : 483
XP_0345786 : EGGSSSVELQRLCEALVVGAVLPTK------ : 483
XP_0345787 : EGGSSVVSLQRLYDALIQGAVEPKV------ : 492
XP_0049577 : EGGSSVVSLQRLYDALIQGAVEPKV------ : 492
XP_0024449 : EGGSSDASLKRLCDALHQGAVLPARKFPQDT : 514
XP_0147525 : EGGSSYSALGSLAEEMIKGVNQRSPH----- : 477
RLN34493.1 : EGGSSDAALHRLAEELCKGAVVSTGK----- : 534
EMS62308.1 : HSGSSCASVRRLSEDLLRGAAVLPTV----- : 485
XP_0024607 : EGGSSYAALRRLSEEIMC--KVDTNQ----- : 474
ScGT1_1 : EGGSSNGSLQSLCHALVEGAVHPRK------ : 480
 egGSS 6 rL 6 ga

### (B). Molecular Phylogenetic analysis by Maximum Likelihood method (ScGT1_1)


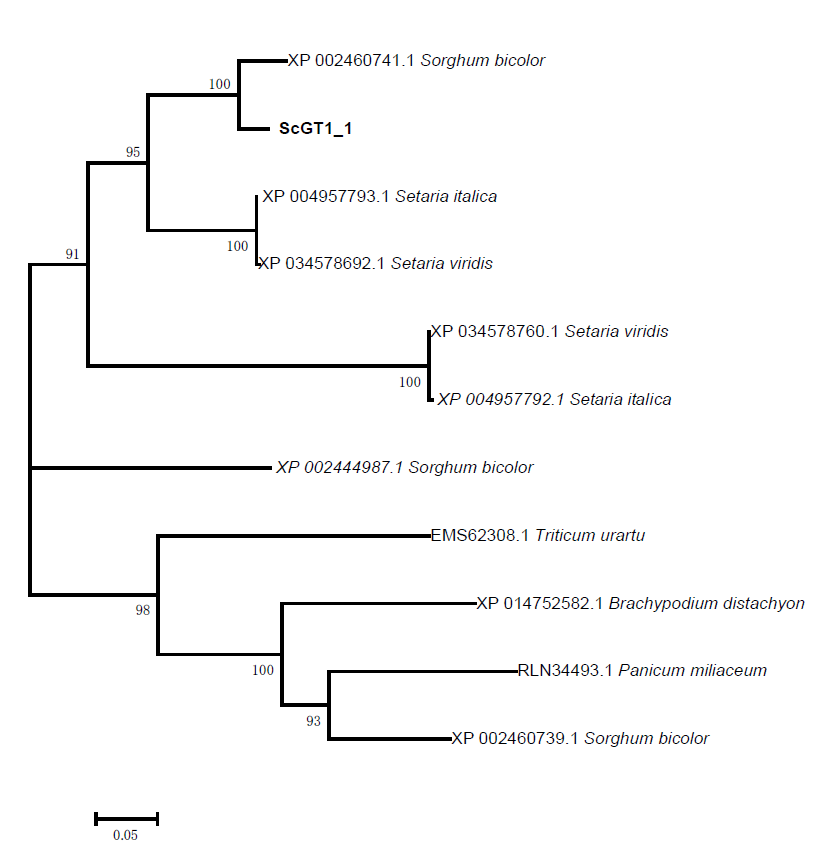


### (C). Multiple sequence alignment of ScGT1_2 gene.

* 20 * 40 * 60
XP_0024563 : ------------------------------------------------------------ : -
XP_0213136 : ------------------------------------------------------------ : -
PWZ32989.1 : ------------------------------------------------------------ : -
RLN24822.1 : ------------------------------------------------------------ : -
XP_0049698 : ------------------------------------------------------------ : -
RLM92047.1 : -------------------------------------------------MWFAGKAVAQQ : 11
XP_0049698 : ---------------KSTLLASLGLPFPISHHKWPSRSEEYSITCGASSKSSRHINKSST : 45
XP_0345947 : MEKTIMKANPTCCKIWSTLLASLGLPFPISHHKWPSRSEEYSITCGASSKSSRHINKSST : 60
RLN24208.1 : ------------------------------------------------------------ : -
ONM38427.1 : ------------------------------------------------------------ : -
ScGT1_2 : ------------------------------------------------------------ : -


 * 80 * 100 * 120
XP_0024563 : ----------------------------------------MDSVV-VSAKQTVVLYPGGG : 19
XP_0213136 : ----------------------------------------MDN-V-VSSKQTVVLYPGGG : 18
PWZ32989.1 : ----------------------------------------MDDVVPATAKQTVVLYPGGG : 20
RLN24822.1 : ----------------------------------------MDN---GSAKQTVVLFPSGG : 17
XP_0049698 : ----------------------------------------MDN---GPAKQTVVLYPGGG : 17
RLM92047.1 : RRYKSWALQSAASHLHSSSEATATVLSFPILPSGWAATAPMDN---GSAKQTVVLFPGGG : 68
XP_0049698 : ASPTSSKAVRRSIEVARRSQRQPCPRQSIFLPPNSLATAQMES---ASVKQIVVLYPVGG : 102
XP_0345947 : ASPTSSKAVRRSIEVARRSQRQPCPRQSIFLPPNSLATAQMES---ASVKQIVVLYPVGG : 117
RLN24208.1 : ----------------------------------------MDN---GSVKQLVVLYPVGG : 17
ONM38427.1 : ----------------------------------------MDD---VSVKQIAVLYPVGG : 17
ScGT1_2 : ----------------------------------------MDS---GSVKQTVVLYPGGG : 17
 Md KQ vVL5P GG

 * 140 * 160 * 180
XP_0024563 : AGHVGPMTQLAKVFLHHGYDVTMVLLEPPVKSIASSSGFIEGLAAANPSITFHLLPPVPP : 79
XP_0213136 : AGHVGPMTQLAKVFLHHGYDVTMVLLEPPVKSTASGASFVESLAASNPSVVFHALPPIPL : 78
PWZ32989.1 : AGHVAPMTQLAKVFVRHGYDVTMVLLEPPIKSNASGASFVESFAASNPSITFHLLPPIPP : 80
RLN24822.1 : VGHVGPMTQLAKVFLDHGFDVTMVIIEPPIKSTDSGAGFIERVAASNPSITFHVLPPIPP : 77
XP_0049698 : VGHVGPMTELAKVFLDHGHDVTMVLIEPPIKSTDSGAGFIERVAASNPSITFHVLPSIPP : 77
RLM92047.1 : VGHVGPMTQLAKVFLDHGYDVTMVIIEPPIKSTDSGAGFIERVAASNPSITFHVLPPIPP : 128
XP_0049698 : IGHVGPMTQLAKVFLDHGYDVTMVLIEPPIKSTDSGADFIERVAAPYPSITFHVLPPIPP : 162
XP_0345947 : IGHVGPMTQLAKVFLDHGYDVTMVLIEPPIKSTDSGADFIERVAAPYPSITFHVLPPIPP : 177
RLN24208.1 : VGHVGPMTQLAKVFLGHGYDVTMVLIEPPIKSTDSGAGFIERVAASNPSITFHILPSIPT : 77
ONM38427.1 : VGHVGPMTQLAKVFLHHGYDVTMVLIEPPIKSTDSGAGFIERVAASNPSITFHVLPPTPA : 77
ScGT1_2 : AGHVGPMTQLAKVFLHHGYDVNMVLLEPPIKSIASGASFIEGLAASNPSITFHLLPPIPP : 77
 GHVgPMT2LAKVF6 HG DVtMV66EPP6KS Sga F6E AA nPS6tFH LPp P

 * 200 * 220 * 240
XP_0024563 : PDFASATKHSFLFMMELLGQYNDKLESFLRS-IPRERLHSLVIDMFCTDAIDVAAKVGVP : 138
XP_0213136 : PDFASSTKHPFLLLQELLAQYNDKLENFLRS-IPRERLHSLVIDMFCTDAIDVAAKVGVP : 137
PWZ32989.1 : PDLASSTKHPFLVVLELLGQYNDKLESFLRT-IPRERLHSLVIDMFCTDAIDVAAKVGVP : 139
RLN24822.1 : PDFDSSRKHPFLLMLELMRQYNEKLESFLRS-IPRARLHSLVVDMFCTQAIDVAAKLGVP : 136
XP_0049698 : PDFASSPKHPFLLMLELMRLYNEKLESFLRS-IPRERLHSLVIDMFCTQAIDVATKLGVP : 136
RLM92047.1 : PDFASSRKHPFLLMMEFMRQYNEKLESFLRSIVPRARLHSLVIDMFCTQAIDVAAKLGVP : 188
XP_0049698 : PDLTSSTKHPFLLILELMRRYNDKLESFLRS-IPRERLHSLVIDLFCTHAIDIATKVGVP : 221
XP_0345947 : PDLTSSTKHPFLLILELMRRYNDKLESFLRS-IPRERLHSLVIDLFCTHAIDIATKVGVP : 236
RLN24208.1 : PDPATSPKHPFLLILELMRQYNENLESFLRS-IPRARLHSLVIDLFCTHAIDVAAKLGVP : 136
ONM38427.1 : PDVASSTKHPFLLILELMRQYNDKLESFLRS-IPRERLHSLVIDLFCTHAIDVATRLGVP : 136
ScGT1_2 : PDFASATKHPFLLMQELLGQYNDKLESFLRS-IPRERLHSLVIDMFCTAAIDVAAKVGVP : 136
 PD 3s KHpFL 6 El6 YN kLEsFLR3 6PR RLHSLV6D6FCT AID6A 46GVP

 * 260 * 280 * 300
XP_0024563 : VYTFFAASAGALSVLTQTAALLAGRKTGLKELGDTPIEFLGVPPMPASHILRDMLEDPED : 198
XP_0213136 : VYTFFSANAGALAVLTQTAALLAGRQTGLKELGDTPIEFLGVPPMPASHIIREMLEDPED : 197
PWZ32989.1 : VYTFFAANAGALAVLTQTVALLDGRQTGLKELGDTPIEFLGVPPIPASHIIREMLEDAED : 199
RLN24822.1 : VYTFFASGAGVLAVFTQLPALLAGRRTGLKELGDTPLEFLGVPPMPASHLIRELLEDPED : 196
XP_0049698 : VYTFFASGAGVLAVLTQLPALLAGRQSGLKELGDTPLEFLGVPPMPASHLIRELLEDPED : 196
RLM92047.1 : VYTFYASGAGVLAVFTQLPALLAGRRTGLKELGDTPLEFLGVPPMPASHLVRELLEDPED : 248
XP_0049698 : VYKFFASGAGTLAVFTQLPALLSCRQTGLKELGDTPLEFLGVPPMPASHLVKSLLENPED : 281
XP_0345947 : VYKFFASGAGTLAVFTQLPALLSCRQTGLKELGDTPLEFLGVPPMPASHLVKSLLENPED : 296
RLN24208.1 : VYKFFASGAGTLAVFTQLPALLAGRRTGLKELGDTPLEFLGVPPMPASHLVKSLLEHPDD : 196
ONM38427.1 : VFKFFASGAGTLAIFTQLPALLAGRLTGLKELGDKPLQFLGVPPMPASHLATSLLESPED : 196
ScGT1_2 : VYTFFAANAGALAVLTQTAALLAGRQTGLTELGDTPIDFLGVPPMPASHILREMLEDPDD : 196
 V5 F5a AG La6 TQ ALL gR 3GLkELGDtP6 FLGVPP6PASH6 6LE peD

 * 320 * 340 * 360
XP_0024563 : EVCKAMAEIWKRNTDTRGVLINTFYSLESPALQAFSDPLCVPGKVLPPVYSIGPLVGEGG : 258
XP_0213136 : KVCRAMAEIWKRDTDTRGVLINTFYSLEASALEAFRDPLCVPGKVLPPVYSIGPLVGEGG : 257
PWZ32989.1 : EVRTAMAKIWKRDTDTRGVLINTFYSLEAQALQAFRDPLCVPGKVLPPVYPIGPLVGKGG : 259
RLN24822.1 : ELCRTMMNIWKRNTDTHGVLVNTFCSLESRALQAFRDPLCVPGRVLPPVYSIGPLVGKGG : 256
XP_0049698 : ELCKTMVNIWKRNTDTQGVLVNTFYSLESRALQAFRDPLCVPGEVLPPVYSVGPLVGKG- : 255
RLM92047.1 : ELCRTMMNIWKRNTDTLGVLVNTFWSLESRALQAFRDPLCVPGRVLPPVYSIGPLVGKGG : 308
XP_0049698 : ELCRTMMKILKRNADTHGVLVNTFESLESRALQALRDPLCVPGQILPPVYPIGPLVGKG- : 340
XP_0345947 : ELCRTMMKILKRNADTHGVLVNTFESLESRALQALRDPLCVPGQILPPVYPIGPLVGKG- : 355
RLN24208.1 : ELCGAMMKILKRNAGTHGVLVNTFESLESRALQGLRDPHCVPGQVPPPVYAIGPLTGKA- : 255
ONM38427.1 : ELCRTSMEILERNAGTHGVLVNTFESLEQRALQALRDPLCVPGQALPPVYPIGPLVGTGT : 256
ScGT1_2 : EVCKTMAEIWKRNTDTRGVLINTFYSLECRALQAFGDPLCVPGKVLPPVYSIGPLVGEGG : 256
 e6c m I kR1 dT GVL6NTF SLE AL2a rDPlCVPG lPPVY 6GPLvG g

 * 380 * 400 * 420
XP_0024563 : --THGGEG-ER-HECLAWLDAQPERSVVFLCWGSRGLLSGEQLKDIAAGLDKSGQRFLWV : 314
XP_0213136 : --TETEEAGKR-HECLTWLDAQPERSVVFLCWGSKGLLSGEQLKEIAVGLEKSGQRFLWV : 314
PWZ32989.1 : SGTDGGEAAERPHECLAWLDAQPERSVVFLCWGSRGLLSEEQLKEIAAGLEKSGQRFLWV : 319
RLN24822.1 : IGADEEEA-ER-PECLAWLDAQPERSVVFLCWGSKGSLPEEQIKEIAAGLEKSEQRFLWV : 314
XP_0049698 : -GADKEEA-ER-HECVAWLDAQPERSVVFLCWGSKGSLSEEQIKEIAAGLEKSGQRFLWV : 312
RLM92047.1 : SGADEEEA-ER-PECLVWLDAQPERSVVLLSWGSKGSLPEEQIKEIAAGLEKSGQRFLWV : 366
XP_0049698 : -----GTDKEK-HECLAWLDAQQERSVVFLCWGSKGALPKEQLMEIAVGLENSGQRFLWV : 394
XP_0345947 : -----GTDKEK-HECLAWLDAQQERSVVFLCWGSKGALPKEQLMEIAVGLENSGQRFLWV : 409
RLN24208.1 : -----GTGREG-LECLAWLDAQPERSVVFLCWGSKGALPKQQLEEIASGLESSGHRFLWV : 309
ONM38427.1 : -GRQEGDGGPQ-HECLAWLDAQPERSVAFLCWGSKGALPKEQLKETAVGLERCGQRFLWV : 314
ScGT1_2 : --THREEA-ER-HECLTWLDAQPERSVVFLCWGSRGLLSGEQLKEIAAGLDKSGQRFLWV : 312
 e EC6 WLDAQpERSVvfLcWGS4G L 2Q6 eiA GLe sgqRFLWV

 * 440 * 460 * 480
XP_0024563 : VRTPAS--DPKRRFEPRPEPDLGALLPEGFLERTRDRGLVLKSWAPQVDVLHNPAIGAFV : 372
XP_0213136 : VRTPAAS-DPKRLWETRPEPDLDTLLPEGFLERTRDRGLVIKSWVPQVDVLNNPAIGAFV : 373
PWZ32989.1 : VRTPASSDDPKRFWLPRPEPDLDALLPEGFLERTKDRGLVIKSWAPQVDVLSNPAVGAFV : 379
RLN24822.1 : VRTPAGSDDPIRYLEQLPEPDLDALLPEGFLERTRGRGFVIKSWAPQGDILTHPATGAFV : 374
XP_0049698 : VRTPAGTDDPKRYLEKRPEPDLDALLPEGFLERTKGRGFVVKSWAPQVDVLMHPATGAFV : 372
RLM92047.1 : VRTPAGSDDPKRYLEQRPEPDLDALLPEGFLERTKGRGFVIKSWAPQADILTHPATGAFV : 426
XP_0049698 : VRTPAGSDEPKRYWEQRAEADLGALLPEGFLERIKGRGLVIKSWAPQVDVLGHRATGAFV : 454
XP_0345947 : VRTPAGSDEPKRYWEQRAEADLGALLPEGFLERIKGRGLVIKSWAPQVDVLGHRATGAFV : 469
RLN24208.1 : VRTPAGSDDPKRYWERRGEADLGALLPEGFSERTKDRGLVIRSWAPQVDVLNHPATGAFV : 369
ONM38427.1 : VRTPAGRDGPGRYWEQRAEADLDALLPEGFVERTKDRGLVVTSWAPQVDVLNHPATGVFV : 374
ScGT1_2 : VRTPPS--DPKRRWEPRPEPDLDTLLPEGFLERTRDRGLVIKSWAPQVDVLNNPAIGAFV : 370
 VRTPa PkR e r E DL aLLPEGF ERt4 RG V6 SWaPQvD6L pA GaFV

 * 500 * 520 * 540
XP_0024563 : THCGWNSALEAITAGVPMLCWPLD-AEQKTNKVLMTEA-MGIGLELEGYNTGFIKAEEIE : 430
XP_0213136 : THCGWNSTLEAIAAGVPMLCWPLG-AEQKINKVLMVEA-MGIGLELEGYNTGFIKAEEVE : 431
PWZ32989.1 : THCGWNSSLEAITAGVPMLCWPQG-AEQKINKVLMTEA-MGIGLELEGYNTGFIKAEEIE : 437
RLN24822.1 : THCGWNSTLEAIVAGVPMLCWPLG-AEQKMNKVLMTED-MGVGVELEGYKAGLVKAEEVE : 432
XP_0049698 : THCGWNSTLEAIVAGVPMLCWPLG-AEQKMNKVLMVDESMSIGVELEGYNTGFVKAEEIE : 431
RLM92047.1 : THCGWNSTLEAIAAGVPMLCWPLG-AEQKMNKVLMTAD-MGVGVELEGYKAGLIKAEEVE : 484
XP_0049698 : THCGWNSTLEAVAAGVPMLCWPLAAAEQKMNKVFITDD-MGIGVEMEGYKAGFIRAEEIE : 513
XP_0345947 : THCGWNSTLEAVAAGVPMLCWPLAAAEQKMNKVFITDD-MGIGVEMEGYKAGFIKAEEIE : 528
RLN24208.1 : THCGWNSTLEAVAAGVPMLCWPLAAAEQKMNKVFVTDD-MGVGVEMEGYRDGFITAEEIE : 428
ONM38427.1 : THCGWNSTLEAIAAGVPMLCWPLAGAEQRMNKVFITED-MGVGMEMEGYMTGLIKAEEIE : 433
ScGT1_2 : THCGWNSTLEAIAAGVPMLCWPLD-AEQMINKVLMTEA-MGIGLELEGYNTGFIKAEEIE : 428
 THCGWNS LEA6 AGVPMLCWPl AEQ NKV 6t Mg6G6E6EGY G 6 AEE6E

 * 560 * 580 *
XP_0024563 : TKVRLMLE-SEEGREIRTRAAELKKEAHEALEDGGSSQAAFLQFLSDVKNISE- : 482
XP_0213136 : TKVRLVLE-SELRREIRMRAAELKKEAHEALEDGGSSQAAFLQFLSDVKNITSA : 484
PWZ32989.1 : TKVRLVLE-SEEGREIRTRAAEVKKEAHAALEDGGSSEAAFLQFLSDVKNIN-- : 488
RLN24822.1 : AKVRLVLE-SEEGRELRERAAERKKEAEAALEDGGSSRAAFLQFLSDVENLRA- : 484
XP_0049698 : AKVKLVLE-SGEGRELRERAAELKKEAEAAMEDGGSSRMAFLQFLSDVKNLRG- : 483
RLM92047.1 : AKVRLVLE-SEEGRELRERAAERKKEAEEALEDGGSSRAAFLQFLSDVKNLRA- : 536
XP_0049698 : AKVRLVLE-CKEGRELRKRAIELKKAAQEAMEDGGSSRAAFLQFLSDVKNLRE- : 565
XP_0345947 : AKVRLVLE-CKEGRELRKRAIERKKAAQEAMEDGGSSRAAFLQFLSDVKNLRE- : 580
RLN24208.1 : AKVRLVLD-CGEGRKLRERAMELKKEAGEAVEDGGSSRAAFLQFLSDVRNLRE- : 480
ONM38427.1 : GKLRLALE-SEEGTRLKKRALQLKKETEEAMEDGGSSEAAFLRFLSDVANVRG- : 485
ScGT1_2 : TKVRLVLQESEEGREIRTRAAEMKKEAHAALEDGGSSQAAFLQFLSDVKNINE- : 481
 K64L L egre64 RA 2 KKea A6EDGGSS aAFLqFLSDV N6

### (D). Molecular Phylogenetic analysis by Maximum Likelihood method (ScGT1_2)


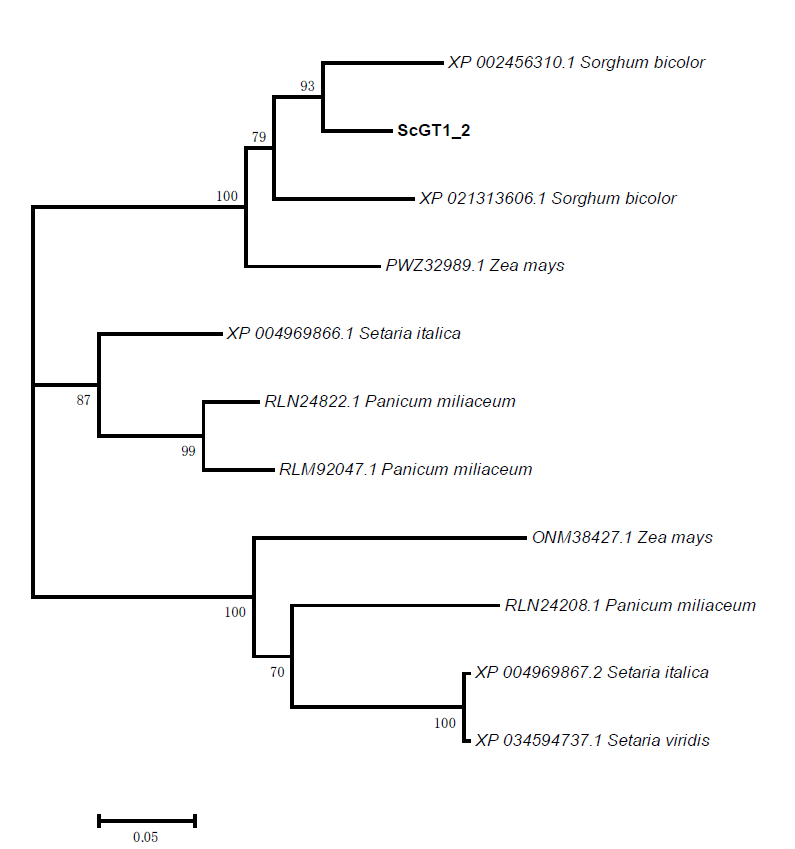


### (E). Multiple sequence alignment of ScGT1_3 gene.

* 20 * 40 * 60
XP_0213136 : ------------------------------------------------------------ : -
XP_0024563 : ------------------------------------------------------------ : -
PWZ32989.1 : ------------------------------------------------------------ : -
XP_0049698 : ------------------------------------------------------------ : -
RLN24822.1 : ------------------------------------------------------------ : -
RLM92047.1 : -----------------------------MWFAGKAVAQQRRYKSWALQSAASHLHSSSE : 31
PVH38071.1 : ------------------------------------------------------------ : -
XP_0049698 : ---------------KSTLLASLGLPFPISHHKWPSRSEEYSITCGASSKSSRHINKSST : 45
XP_0345947 : MEKTIMKANPTCCKIWSTLLASLGLPFPISHHKWPSRSEEYSITCGASSKSSRHINKSST : 60
XP_0258141 : ------------------------------------------------------------ : -
ScGT1_3 : -------------------------PFPLMQRAFHQASSPARQHTAISINNWLVPFRASK : 35


 * 80 * 100 * 120
XP_0213136 : -----------------------------------MDNVV----SSKQTVVLYPGGGAGH : 21
XP_0024563 : -----------------------------------MDSVV---VSAKQTVVLYPGGGAGH : 22
PWZ32989.1 : -----------------------------------MDDVVP--ATAKQTVVLYPGGGAGH : 23
XP_0049698 : ----------------------------------------MDNGPAKQTVVLYPGGGVGH : 20
RLN24822.1 : ----------------------------------------MDNGSAKQTVVLFPSGGVGH : 20
RLM92047.1 : ATATVLSFP--------------------ILPSGWAATAPMDNGSAKQTVVLFPGGGVGH : 71
PVH38071.1 : ----------------------------------------MDNGCAKQTVVLFPGGGVGH : 20
XP_0049698 : ASPTSSKAVRRSIEVARRSQRQPCPRQSIFLPPNSLATAQMESASVKQIVVLYPVGGIGH : 105
XP_0345947 : ASPTSSKAVRRSIEVARRSQRQPCPRQSIFLPPNSLATAQMESASVKQIVVLYPVGGIGH : 120
XP_0258141 : ---------------------------------------------MKQTVILYPAAGVGH : 15
ScGT1_3 : AISSDAEIGSSSPASN------------LLVSTFHMDKEFVPTTMKQQTVVLYPFPGVGH : 83
 kQtV6L5P gG GH

 * 140 * 160 * 180
XP_0213136 : VGPMTQLAKVFLHHGYDVTMVLLEPPVKSTASGASFVESLAASNPSVVFHALPPIPLPD- : 80
XP_0024563 : VGPMTQLAKVFLHHGYDVTMVLLEPPVKSIASSSGFIEGLAAANPSITFHLLPPVPPPD- : 81
PWZ32989.1 : VAPMTQLAKVFVRHGYDVTMVLLEPPIKSNASGASFVESFAASNPSITFHLLPPIPPPD- : 82
XP_0049698 : VGPMTELAKVFLDHGHDVTMVLIEPPIKSTDSGAGFIERVAASNPSITFHVLPSIPPPD- : 79
RLN24822.1 : VGPMTQLAKVFLDHGFDVTMVIIEPPIKSTDSGAGFIERVAASNPSITFHVLPPIPPPD- : 79
RLM92047.1 : VGPMTQLAKVFLDHGYDVTMVIIEPPIKSTDSGAGFIERVAASNPSITFHVLPPIPPPD- : 130
PVH38071.1 : VGPMTQLAKVFLDHGYDVTMVIIEPPIKSTDSGAGFIERVAASNPSITFHVLPPIPPPD- : 79
XP_0049698 : VGPMTQLAKVFLDHGYDVTMVLIEPPIKSTDSGADFIERVAAPYPSITFHVLPPIPPPD- : 164
XP_0345947 : VGPMTQLAKVFLDHGYDVTMVLIEPPIKSTDSGADFIERVAAPYPSITFHVLPPIPPPD- : 179
XP_0258141 : VVPMAELARVFLSHGYDVTMVIVPPPFKSSALGASQIEQIAAANPSISFHVLPPIPAPD- : 74
ScGT1_3 : VVPMVQLAKVFIRHGYDVTIVLVQPPSGLPDFGASVIDRIAASNPVISFHVLPPISEPDT : 143
 V PMt2LA4VF6 HG DVT6V66 PP ks sga f6e AA nPs6 FH LPp6p PD

 * 200 * 220 * 240
XP_0213136 : --FASS--TKHPFLLLQELLAQYNDKLENFLR-SIPRERLHSLVIDMFCTDAIDVAAKVG : 135
XP_0024563 : --FASA--TKHSFLFMMELLGQYNDKLESFLR-SIPRERLHSLVIDMFCTDAIDVAAKVG : 136
PWZ32989.1 : --LASS--TKHPFLVVLELLGQYNDKLESFLR-TIPRERLHSLVIDMFCTDAIDVAAKVG : 137
XP_0049698 : --FASS--PKHPFLLMLELMRLYNEKLESFLRS-IPRERLHSLVIDMFCTQAIDVATKLG : 134
RLN24822.1 : --FDSS--RKHPFLLMLELMRQYNEKLESFLRS-IPRARLHSLVVDMFCTQAIDVAAKLG : 134
RLM92047.1 : --FASS--RKHPFLLMMEFMRQYNEKLESFLRSIVPRARLHSLVIDMFCTQAIDVAAKLG : 186
PVH38071.1 : --FASS--RKHPFLLMIEFMRQYNEKLESFLRSIVPRARLHSLVIDMFCTQAIDVAAKLG : 135
XP_0049698 : --LTSS--TKHPFLLILELMRRYNDKLESFLRS-IPRERLHSLVIDLFCTHAIDIATKVG : 219
XP_0345947 : --LTSS--TKHPFLLILELMRRYNDKLESFLRS-IPRERLHSLVIDLFCTHAIDIATKVG : 234
XP_0258141 : --FAGS--PKHPFLLMLQMLRQYNDKLEGFLRS-IPRQRLHSLVIDMFCVDAIDVAAKLG : 129
ScGT1_3 : DQLAGSGKKKNPLPLLLQMMRRYNDALETFLRSTVPRERLHAVVTSMFTAHAVDVAAKLS : 203
 ss Khpfl 6 2 6 YN kLE FLR 6PR RLHs6V d6Fct A6D6A K6g

 * 260 * 280 * 300
XP_0213136 : VPVYTFFSANAGALAVLTQTAALLAGRQTGLKELGDTPIEFLGVPPMPASHIIREMLEDP : 195
XP_0024563 : VPVYTFFAASAGALSVLTQTAALLAGRKTGLKELGDTPIEFLGVPPMPASHILRDMLEDP : 196
PWZ32989.1 : VPVYTFFAANAGALAVLTQTVALLDGRQTGLKELGDTPIEFLGVPPIPASHIIREMLEDA : 197
XP_0049698 : VPVYTFFASGAGVLAVLTQLPALLAGRQSGLKELGDTPLEFLGVPPMPASHLIRELLEDP : 194
RLN24822.1 : VPVYTFFASGAGVLAVFTQLPALLAGRRTGLKELGDTPLEFLGVPPMPASHLIRELLEDP : 194
RLM92047.1 : VPVYTFYASGAGVLAVFTQLPALLAGRRTGLKELGDTPLEFLGVPPMPASHLVRELLEDP : 246
PVH38071.1 : VPVYTFFASGAGVLAVFTQLPALLAGRRTGLKELGDTPLEFLGVPPMPASHLVRELLEDP : 195
XP_0049698 : VPVYKFFASGAGTLAVFTQLPALLSCRQTGLKELGDTPLEFLGVPPMPASHLVKSLLENP : 279
XP_0345947 : VPVYKFFASGAGTLAVFTQLPALLSCRQTGLKELGDTPLEFLGVPPMPASHLVKSLLENP : 294
XP_0258141 : VPAYTFVPSGASSLAVVTQLPTLLAGRQTGLEELGDTPLEFLGVPPVPASHLIAELLAHP : 189
ScGT1_3 : VPVYTFFAANAGALAVLTQTAALLAGRQTGLTELGDTPIDFLGVPPMPASHILREMLEDP : 263
 VPvYtF a Ag LaV TQ aLL gR 3GLkELGDTP6eFLGVPP6PASH66 6Le p

 * 320 * 340 * 360
XP_0213136 : EDKVCRAMAEIWKRDTDTRGVLINTFYSLEASALEAFRDPLCVPGKVLPPVYSIGPLVGE : 255
XP_0024563 : EDEVCKAMAEIWKRNTDTRGVLINTFYSLESPALQAFSDPLCVPGKVLPPVYSIGPLVGE : 256
PWZ32989.1 : EDEVRTAMAKIWKRDTDTRGVLINTFYSLEAQALQAFRDPLCVPGKVLPPVYPIGPLVGK : 257
XP_0049698 : EDELCKTMVNIWKRNTDTQGVLVNTFYSLESRALQAFRDPLCVPGEVLPPVYSVGPLVGK : 254
RLN24822.1 : EDELCRTMMNIWKRNTDTHGVLVNTFCSLESRALQAFRDPLCVPGRVLPPVYSIGPLVGK : 254
RLM92047.1 : EDELCRTMMNIWKRNTDTLGVLVNTFWSLESRALQAFRDPLCVPGRVLPPVYSIGPLVGK : 306
PVH38071.1 : DDELCRTMMNIWKRNTDTLGVLS--------RALQAFRDPLCVPGRVLPPVYSIGPLVGK : 247
XP_0049698 : EDELCRTMMKILKRNADTHGVLVNTFESLESRALQALRDPLCVPGQILPPVYPIGPLVGK : 339
XP_0345947 : EDELCRTMMKILKRNADTHGVLVNTFESLESRALQALRDPLCVPGQILPPVYPIGPLVGK : 354
XP_0258141 : EEEQCKTMVSIFERGMNSRGVLVNTFESFESRAVRALRDPLCFPGKVLPPVYCVGPLVSG : 249
ScGT1_3 : DDEVCKTMAEIWKRNTDTRGVLINTFYSLECRALQAFGDPLCVPGKVLPPVYSIGPLVGE : 323
 ede c M I kR 13 GVL ntf sle A6 A rDPLCvPG 6LPPVY 6GPLVg

 * 380 * 400 * 420
XP_0213136 : GG--TETEEAGKR-HECLTWLDAQPERSVVFLCWGSKGLLSGEQLKEIAVGLEKSGQRFL : 312
XP_0024563 : GG--THGGEGER--HECLAWLDAQPERSVVFLCWGSRGLLSGEQLKDIAAGLDKSGQRFL : 312
PWZ32989.1 : GGSGTDGGEAAERPHECLAWLDAQPERSVVFLCWGSRGLLSEEQLKEIAAGLEKSGQRFL : 317
XP_0049698 : G--GADKEEAER--HECVAWLDAQPERSVVFLCWGSKGSLSEEQIKEIAAGLEKSGQRFL : 310
RLN24822.1 : GGIGADEEEAER--PECLAWLDAQPERSVVFLCWGSKGSLPEEQIKEIAAGLEKSEQRFL : 312
RLM92047.1 : GGSGADEEEAER--PECLVWLDAQPERSVVLLSWGSKGSLPEEQIKEIAAGLEKSGQRFL : 364
PVH38071.1 : GGSGADEEEAER--PECIVWLDAQPERSVVLLSWGSKGSLPEEQIKEIAAGLEKSGQRFL : 305
XP_0049698 : G--GTDKE---K--HECLAWLDAQQERSVVFLCWGSKGALPKEQLMEIAVGLENSGQRFL : 392
XP_0345947 : G--GTDKE---K--HECLAWLDAQQERSVVFLCWGSKGALPKEQLMEIAVGLENSGQRFL : 407
XP_0258141 : GG--RNGEKTER--HECLAWLDAQPERSVVFLCFGSMGTFSVEQLKEIAVGLEKSGQRFL : 305
ScGT1_3 : GG--THREEAER--HECLTWLDAQPERSVVFLCWGSRGLLSGEQLKEIAAGLDKSGQRFL : 379
 G e EC6 WLDAQpERSVVfLc5GS G l EQ6keIA GLekSgQRFL

 * 440 * 460 * 480
XP_0213136 : WVVRTPAAS-DPKRLWETRPEPDLDTLLPEGFLERTRDRGLVIKSWVPQVDVLNNPAIGA : 371
XP_0024563 : WVVRTPAS--DPKRRFEPRPEPDLGALLPEGFLERTRDRGLVLKSWAPQVDVLHNPAIGA : 370
PWZ32989.1 : WVVRTPASSDDPKRFWLPRPEPDLDALLPEGFLERTKDRGLVIKSWAPQVDVLSNPAVGA : 377
XP_0049698 : WVVRTPAGTDDPKRYLEKRPEPDLDALLPEGFLERTKGRGFVVKSWAPQVDVLMHPATGA : 370
RLN24822.1 : WVVRTPAGSDDPIRYLEQLPEPDLDALLPEGFLERTRGRGFVIKSWAPQGDILTHPATGA : 372
RLM92047.1 : WVVRTPAGSDDPKRYLEQRPEPDLDALLPEGFLERTKGRGFVIKSWAPQADILTHPATGA : 424
PVH38071.1 : WVVRTPAGSDDPKRYLEQRPEPDLDALLPEGFLERTKGRGFVIKSWAPQADILTHPATGA : 365
XP_0049698 : WVVRTPAGSDEPKRYWEQRAEADLGALLPEGFLERIKGRGLVIKSWAPQVDVLGHRATGA : 452
XP_0345947 : WVVRTPAGSDEPKRYWEQRAEADLGALLPEGFLERIKGRGLVIKSWAPQVDVLGHRATGA : 467
XP_0258141 : WVVRVPANINDPKRLLDKPCEPDLDALLPEGFLERTKDRGLVIKSWAPQVDVLNHPATGA : 365
ScGT1_3 : WVVRTPPS--DPKRRWEPRPEPDLDTLLPEGFLERTRDRGLVIKSWAPQVDVLNNPAIGA : 437
 WVVRtPa dPkR e r EpDL aLLPEGFLERt4 RG V6KSWaPQ D6L pA GA

 * 500 * 520 * 540
XP_0213136 : FVTHCGWNSTLEAIAAGVPMLCWPLG-AEQKINKVLMVEA-MGIGLELEGYNTGFIKAEE : 429
XP_0024563 : FVTHCGWNSALEAITAGVPMLCWPLD-AEQKTNKVLMTEA-MGIGLELEGYNTGFIKAEE : 428
PWZ32989.1 : FVTHCGWNSSLEAITAGVPMLCWPQG-AEQKINKVLMTEA-MGIGLELEGYNTGFIKAEE : 435
XP_0049698 : FVTHCGWNSTLEAIVAGVPMLCWPLG-AEQKMNKVLMVDESMSIGVELEGYNTGFVKAEE : 429
RLN24822.1 : FVTHCGWNSTLEAIVAGVPMLCWPLG-AEQKMNKVLMTED-MGVGVELEGYKAGLVKAEE : 430
RLM92047.1 : FVTHCGWNSTLEAIAAGVPMLCWPLG-AEQKMNKVLMTAD-MGVGVELEGYKAGLIKAEE : 482
PVH38071.1 : FVTHCGWNSTLEAIAAGVPMLCWPLG-AEQKMNKVLMTAD-MGVGVELEGYKAGLIKAEE : 423
XP_0049698 : FVTHCGWNSTLEAVAAGVPMLCWPLAAAEQKMNKVFITDD-MGIGVEMEGYKAGFIRAEE : 511
XP_0345947 : FVTHCGWNSTLEAVAAGVPMLCWPLAAAEQKMNKVFITDD-MGIGVEMEGYKAGFIKAEE : 526
XP_0258141 : FVTHCGWNSTMEGIMAGVTMLCWPLY-AEQKMNKIFMTED-MGVGVEIEGYRTGFIKAEE : 423
ScGT1_3 : FVTHCGWNSTLEAIAAGVPMLCWPLD-AEQKINKVLMTEA-MGIGLELEGYNTGFIKAEE : 495
 FVTHCGWNS 6Ea6 AGVpMLCWPl AEQK NK6 6t Mg6G6E6EGY G 64AEE

 * 560 * 580 *
XP_0213136 : VETKVRLVLE-SELRREIRMRAAELKKEAHEALEDGGSSQAAFLQFLSDVKNITSA--- : 484
XP_0024563 : IETKVRLMLE-SEEGREIRTRAAELKKEAHEALEDGGSSQAAFLQFLSDVKNISE---- : 482
PWZ32989.1 : IETKVRLVLE-SEEGREIRTRAAEVKKEAHAALEDGGSSEAAFLQFLSDVKNIN----- : 488
XP_0049698 : IEAKVKLVLE-SGEGRELRERAAELKKEAEAAMEDGGSSRMAFLQFLSDVKNLRG---- : 483
RLN24822.1 : VEAKVRLVLE-SEEGRELRERAAERKKEAEAALEDGGSSRAAFLQFLSDVENLRA---- : 484
RLM92047.1 : VEAKVRLVLE-SEEGRELRERAAERKKEAEEALEDGGSSRAAFLQFLSDVKNLRA---- : 536
PVH38071.1 : VEAKVRLVLE-SEEGRELRERAAERKKEAEEALEDGGSSRAAFLQFLSDVKNLRA---- : 477
XP_0049698 : IEAKVRLVLE-CKEGRELRKRAIELKKAAQEAMEDGGSSRAAFLQFLSDVKNLRE---- : 565
XP_0345947 : IEAKVRLVLE-CKEGRELRKRAIERKKAAQEAMEDGGSSRAAFLQFLSDVKNLRE---- : 580
XP_0258141 : VEAKVRLVME-SEEGRELQARVVARRKEAEAALEVGGSSHAAFVQFLLDVENLGEELAH : 481
ScGT1_3 : IETKVRLVLQESEEGREIRTRAAELKKEAHAALEDGGSSQAAFLQFLSDVKNINE---- : 550
 6E KV4L662 s egRE6r Ra e 4KeA A6EdGGSS aAF6QFLsDVkN6

### (F). Molecular Phylogenetic analysis by Maximum Likelihood method (ScGT1_3)


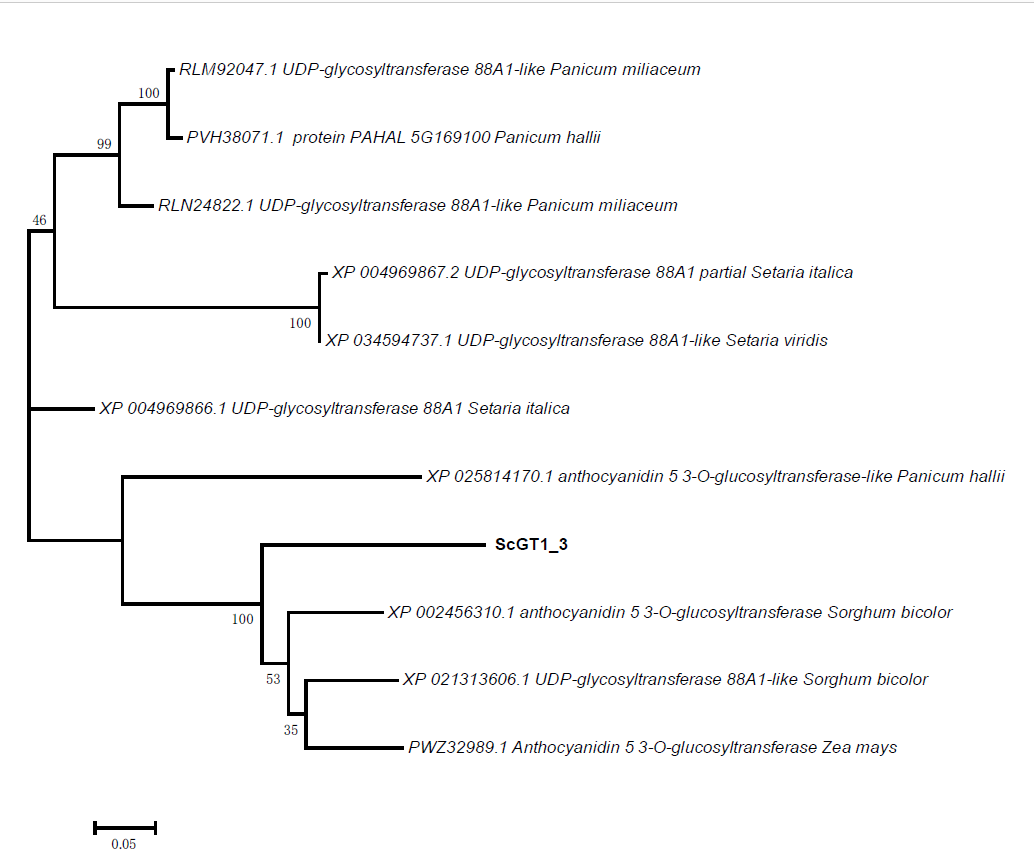


## Fig S16. Sequence alignment of Malonyl-CoA: anthocyanidin 5-O-glucoside-6''-O-malonyltransferase (5MaT) proteins from sugarcane and various other plants, and phylogenetic relationships of Malonyl-CoA: anthocyanidin 5-O-glucoside-6''-O-malonyltransferase.

### (A). Multiple sequence alignment of Sc5MaT_1 gene.

* 20 * 40 * 60
XP_0024561 : MAMAAAPDQQQ--LPAGA------SSSSPRLR--IHDTTLVPPSPSPPETSLPLTYFDIF : 50
XP_0049695 : --MAVAPDQKQ--RPAGS------SSASPHLR--VHDTILVAPSPSPPETSLPLTFFDII : 48
XP_0345977 : --MAVAPDQKQ--RPAGS------SSASPHLR--VHDTILVAPSPSPPQTSLPLTFFDII : 48
XP_0049695 : --MAVAPEQHQ--QPAG-------ASSSPRIR--VHDTTLVSPSPSPREASLPLTFFDIF : 47
OEL30804.1 : --MAAAPDQEQ-----LA------SSASPRFRV-LDDTTLVPPSPSPPETSLPLTFFDII : 46
RLN22214.1 : MAMAVAPDQQQ--VPAGA------SSAPPRLR--VHDTTLVPPSPSPPETSLPLTFFDII : 50
RLM92687.1 : MAMAVAPDQQQ--VPAGA------SSAPPRLLC-VHDTMLVPPSPSPPETSLPLTFFDII : 51
XP_0024614 : --MAVTPDQQQPPPPAGA------SSTPPRLLVHVHETTLVPPSPSPPETSLPLTFLDVF : 52
RLN23247.1 : --MAAASEQQQ--LHAGA------SLSSPRLR--VHVTTLVQPSPSPPEISLPLTFFDSF : 48
XP_0024389 : --MAMAPDQQQPPAGAGAGASSTTGSTSPRLRVHVHDTTLVPPSPSPPETSLPLTFFDVF : 58
CL1Contig2 : --MAAAPDQQQQ-LPAGA------ASSSPRLR--AHDTTLVPPSPSPPETSLPLTFFDIF : 49
 MA apdQ Q ag s Pr r hdT LV PSPSPp2tSLPLT5fD

 * 80 * 100 * 120
XP_0024561 : WLHSPPVERLLFYRLA-ADADVATIISNLRDSLHQAVRAFYPLAGRIRLTPGTSDRYELH : 109
XP_0049695 : WLNSPPVERLLFYRLA-PDADVATIISNLKDSLHKAFRVFYPLAGRLRLTPGTSDRYELY : 107
XP_0345977 : WLNSPPVERLLFYRLA-PDADVATIISNLKDSLHKAFRVFYPLAGRLRLTPGTSDRYELY : 107
XP_0049695 : WLNSPPVERLFFYRLS-PDADVATIVSNLKKSLQQAVRAFYPLAGRLRLIPGTSDRYELY : 106
OEL30804.1 : WLNSPPVERLFFYRLA-PDADVATIISNLKDSLRQAVRAFYPLAGRLRLSPGTSDRYELY : 105
RLN22214.1 : WLHSPPVERLFFYRLA-PDADVAAIVSNLKDSLHQAVRAFYPLAGRLRRTPGTSDRYELY : 109
RLM92687.1 : WLHSPPVERLFFYRLA-PDADIDNIVSNLKDSFHQAVRAFYPLAGRLRLTPGASDRYELY : 110
XP_0024614 : FLHSTPVKRLFLYRLAGPDADVAAIISSLRDSLHQALRAFYPLAGRVRLTPGTSDRYELH : 112
RLN23247.1 : WLHSPPVERLFFYRLA-PDADVAAIVSNLKDSLHHAVSAFYPLAGHLCLSPGTSGRYELY : 107
XP_0024389 : WLQSHPVERLFLYRLA-HDADVEAIISNLRSSLHKALAAFYPLAGRVRLTPGTSDRYELH : 117
CL1Contig2 : WLQSPPVERLLFYRLA-PDADVATIISSLRDSLHQAVRAFYPLAGRIRLTPGTSDRYELH : 108
 5L SpPVeRL fYRLa pDAD6a I6SnL4dSlh A raFYPLAGr6rl PGtSdRYEL

 * 140 * 160 * 180
XP_0024561 : YQPGDAVTFTVAEYDDDD-ADADIDGLTADEPREVAKIATLVPPLPEGGGLFALQATLLS : 168
XP_0049695 : YSPGDAVTFTVAECDDG---DADIDGLAAGDPREVAKIGTLVPPLAEGGGLFALQATLLS : 164
XP_0345977 : YSPGDAVTFTVAECDDG---DADIDGLAAGDPREVAKIGTLVPPLAEGGGLFALQATLLS : 164
XP_0049695 : YRPGDAVTFTVAECAD----DEDIDSLTTDDPREVSKIAPLVPALPEGGGLLALQATLLS : 162
OEL30804.1 : YRPGDGVAFTVAECNDD----ADIDGLATDEPREVAKIATLVPPLPDGGGLLALQATLLS : 161
RLN22214.1 : YRPGDAVTFTVAECDDD---DADIDGLATDDPRELAKIAALVPPLPEGGRLLALQATLLS : 166
RLM92687.1 : YRPGDAVTFTVAECDDD---DADIDGLATDDPREVAKIAALVPPLPEGGRLLALQATLLS : 167
XP_0024614 : YRPGDAVTFTVAECDD----DMHFDGLTTDEPREVAKIAALVPPLPDGGKLLAVQATLLP : 168
RLN23247.1 : YCPGDAVTFTVAECED----NVDIDGLATDDPREVTKIVPLVPPLTEGGGLLALQATLLS : 163
XP_0024389 : YRPGDAVTFTVAECDDDVDGDAHFDALATDEPREVAKIAALVPTLPRGGRLLAVRATLLP : 177
CL1Contig2 : YQPGDAVTFTVAEYDDD---DADIDGLTTDEPREVAKIATLVPPLPEGGGLFALQATLLS : 165
 Y PGDaVtFTVAEc D diDgL d PRE6aKI LVPpL GG L A6qATLLs

 * 200 * 220 * 240
XP_0024561 : ARRGLAIGVTVHHAACDGSGSTHFLHTWAAACISGAEAP-----PPPPPPPPVIDRTLLP : 223
XP_0049695 : ARRGLAIGVTVHHAACDGSNSTHFLHTWAAACS-GTEAP-----PPP-----VIDRTLLA : 213
XP_0345977 : ARRGLAIGVTVHHAACDGSNSTHFLHTWAAACS-GTEAP-----PPP-----VIDRTLLA : 213
XP_0049695 : ASRALAIGVTLHHAACDGSNSTHFLHTWAAACS-GTEAP-----PPP-----VIDRTLLA : 211
OEL30804.1 : ARRSLAIGVTVHHAACDGSGSTHFLHTWAAACT-GVEAP-----PPPP----VIDRTLLT : 211
RLN22214.1 : ARRGLAIGVTVHHAACDGSGSTHFLHTWAAACI-GAEAP-----PPPP----VIDRTLLT : 216
RLM92687.1 : ARRGLAIGVTVHHAACDGSASTHFLHTWTAACI-GAETP-----PPPP----VIDRTLLT : 217
XP_0024614 : ARRGLAIGVTVHHAACDGSGSTHFLHTWAAACRGGAAEPSLPPPPPPP----VIDRTLLP : 224
RLN23247.1 : ARRGLAIGVTAHHVAFDGSGSTHFLHTWAAACK-GADAR-----PLPP----VIDRALLP : 213
XP_0024389 : ARRGLAIGVTLHHAACDGSGSTHFLHTWAATCRGGGAE-----SPPPP----VIDRTLLA : 228
CL1Contig2 : ARRGLAIGVTVHHAACDGSGSTHFLHTWAAACS-GAEAP-----PPPP----VIDRTLIP : 215
 ArRgLAIGVT HHaAcDGS STHFLHTWaAaC G p PpP VIDRtL6

 * 260 * 280 * 300
XP_0024561 : DPRGLYDVFFQGAPSTDELEFVKMSADQLIATFVLSKDDLERVKEAVADEAARRRVAPPR : 283
XP_0049695 : DPRGLYNVFYQEAPSTDEMEFAKMSADQLFATFTLSKDDLQRIKEVVADEAARRGVAPPR : 273
XP_0345977 : DPRGLYNVFYQEAPSTDEMEFAKMSADQLFATFTLSKDDLQRIKEVVADEAARRGVAPPR : 273
XP_0049695 : DPRGLYNVFYQEAPSTDEMEFAKMSADQLFATFTLSKDDLQRIKEVVADEAARRGVAPPR : 271
OEL30804.1 : DPRGLYYIFFQGAPRTDEMEFVNMSADQLFATFTLCKEDLRRVKDAVADEATRRGVAPPR : 271
RLN22214.1 : DARGLYDIFCQAAPSTDEMEFVKMSADQLFATFTLSKDDLQRVKDIVADEAMKRGVAPPR : 276
RLM92687.1 : DPRGLYDIFCQAAPSTDEMEFVKMSADQLFATFTLSKDDLQRVKDIVADEAMRRGVAPPR : 277
XP_0024614 : DPRRLYDVFVQAAPSSQELEFIKVSADQLLATFVLSKDDLTRVKDVVADEATRRGVAPPR : 284
RLN23247.1 : DPRGLYDIISQAAPSTDDLKFVKMSADQILATFTLSKDDLLRVKDVVADEAAKRGVAPPR : 273
XP_0024389 : DPRRLYDAFVQTAPSSEEYEFVKMSADQLFATFTLSKDDLKRVKDAVADEAARRGVAPPR : 288
CL1Contig2 : DPRGLYDVFFQGALSTDEMEFVKMSADQLIATFVLSKDDLKRVKEAVADEAARRRVAPPR : 275
 DpRgLY f Q Aps3de eF k6SADQ6 ATF LsKdDL R6K VADEA 4RgVAPPR

 * 320 * 340 * 360
XP_0024561 : CSSLVATFGLVWSCY-QRAKDEESISGGAGAGP--MACLLFAVDHRSRVKPPLPDKYLGN : 340
XP_0049695 : CSSLVATFGFVWSCY-QRAKESCGSG----EGP--MTCILFPVNHRSRMKPPLPERYLGN : 326
XP_0345977 : CSSLVATFGFVWSCY-QRAKESCGSG----EGP--MTCILFPVNHRSRMKPPLPERYLGN : 326
XP_0049695 : CSSLVATFGFVWSCY-QRAKESCGSG----EGP--MTCILFPVNHRSRMKPPLPERYLGN : 324
OEL30804.1 : CSSLVAAFGFAWSCY-QRAKENC--S--AGESP--MTCMIFPVDHRTRMKPPLPDKYLGN : 324
RLN22214.1 : CSSLVATLGFVWSCYLQRAKESCGSDD---EGS--MTYLLFPVDHRSRMKPPLPDKYLGN : 331
RLM92687.1 : CSSLVATFGFVWSCYLQRAKESCGSD----EGS--MSCLLFPVDHRSRMKPPLPDKYLGN : 331
XP_0024614 : CSSLVATFGFVWSCY-QRAKNG-GGG----EGP-MMTCMVIAVDHRSRMKPPLPDKYLGN : 337
RLN23247.1 : CTSLVATFGFVWWCY-QRAKESGGAD----EDP--TAYMVFPVDHRSRMKPRLPDEYLGN : 326
XP_0024389 : CSSLVATFGLVWSCY-QRGKEGSGGG----AGEGSMACMAFPVDHRSRMKPPLPEKYLGN : 343
CL1Contig2 : CSSLVATFGFVWSCY-QRAKDKESISG-AGAGP--MPCLLFAVDHRSRVKPPLPDKYLGN : 331
 C3SLVAtfGfvWsCY QRaK g m c6 f V1HR3R6KPpLP YLGN

 * 380 * 400 * 420
XP_0024561 : CVGPAFALAPQGELAVAGAAGIFSACAAVASSIDEAVRDIETSAMDVWLDRVKEAGTKGT : 400
XP_0049695 : CVGPAFGMAPKSELAVAGVGGLFTACAAVASAIDEAVRDIGTSSMDAWLDRIKEASANG- : 385
XP_0345977 : CVGPAFGMAPKSELAVAGVGGLFTACAAVASAIDEAVRDIGTSSMDAWLDRIKEASANG- : 385
XP_0049695 : CVGPAFGMAPKSELAVAGVGGLFTACAAVASAIDEAVRDIGTSSMDAWSDRIKEASANG- : 383
OEL30804.1 : CVGPAFAMAPKGELAAAGAGGLFSACSAVASAIDEAVRDIGTSNMDVWDDRVREAMAMG- : 383
RLN22214.1 : CVGPAVGMAPKGELAAAGAGGLFSACAAVVSAIDEAVRGIGTSSMDAWVDRIMEAAAAG- : 390
RLM92687.1 : CVVPAFGMAPKGELAAAGAGGLFSACTAVASAIDEAVRDIGTPSMDAWLDRIREASAKG- : 390
XP_0024614 : CVGPAFALAPTGELAVAGAGGLFSACAAVAASIDEAVRDIGTSSMEAWMDRIKEVLLMD- : 396
RLN23247.1 : CVGPAFVVAHKGELAAAGAGGLFTACAAVASAIDEAVRGIGTSNMDAWVRRIMEAAAAG- : 385
XP_0024389 : CVGPAFALAPTGELAAAGAGGLFSACAAVASAIDEAVRDIGTSSMDAWMDRIREVLPMG- : 402
CL1Contig2 : CVGPAFALAPQGELAAAGAVGLFSACAAVASSIDEAVRDIGTSAMDVWLDRIKEAGATGT : 391
 CVgPAf 6Ap ELA AG gG6F3ACaAVas IDEAVRdIgTs Md W dR6 Ea g

 * 440 * 460 * 480
XP_0024561 : LLSVAGSPRFRVYDLDFGFGRPAKVDIVSVARTGALAMAESRSRSSTGGMEVGVSLQPAG : 460
XP_0049695 : ILSVAGSPRFRVYELDFGFGRPLKVDIVSVARTGAVAVAESR--SCIGGMEVGVSLQPAG : 443
XP_0345977 : ILSVAGSPRFRVYELDFGFGRPLKVDIVSVARTGAVAVAESR--SCIGGMEVGVSLQPAG : 443
XP_0049695 : ILSVAGSPRFRVYELDFGFGRPLKVDIVSVARTGAMAVAESR--SSAGGMEIGVSLQPAD : 441
OEL30804.1 : ILTVAGSPRFRVYELDFGFGRPEKVDIVSVARTGAVAVAESR--GSASGMEVGVSLQPAG : 441
RLN22214.1 : LLSVAGSPRFRVYELDFGFGRPAKVDIVSVARTGAVAVAESR--SSTGGMEVGISLQPAG : 448
RLM92687.1 : VLSVAGSPRFRVYELDFGFGRPAKVYIVSVARTGAVAVAESR--RSIGGMEVGVSLQPAG : 448
XP_0024614 : LLTVSGSPRFRVYDLDFGFGRPAKVEVVPVTRTGVVAVAESR--VGDGGIEVGVSLQPAA : 454
RLN23247.1 : VLSVAGSPRFRVYELDFGFGRLAKVDIVSVARTGEVAVAESR--SSAGGMEVGVSLQPAG : 443
XP_0024389 : LLTVAGSPRFRVYDLDFGFGRPAKVDIVSVARTGAVAVAESR--SGDGGIEVGVSLQPAA : 460
CL1Contig2 : LLSVAGSPRFRVYDLDFGFGRPAKVDIVSVARTGALGMAESR--SSTGGMEVGVSLQPAG : 449
 6L3VaGSPRFRVY LDFGFGRp KVd6VsVaRTGa6a6AESR gG6E6G6SLQPA

 * 500
XP_0024561 : MERFRKCFVDAIAWLHLHQSRVEQS- : 485
XP_0049695 : MDRYRKCFTDGIAWLHQRS------- : 462
XP_0345977 : MDRYRKCFTDGIAWLHQRS------- : 462
XP_0049695 : MDRFRKCMADAIAWLHNHNHQS---- : 463
OEL30804.1 : MERYRKCFADAIAWLHQRT------- : 460
RLN22214.1 : MDRYRKCFADCIAWLHQRS------- : 467
RLM92687.1 : MERYRKCFADGIAWLHQPS------- : 467
XP_0024614 : MGSYKKCFADAIAWLHQRRHESDDSF : 480
RLN23247.1 : MDRYRKCFADGIAWIHNHQS------ : 463
XP_0024389 : MERYRKCFADATLWLHQKT------- : 479
CL1Contig2 : MERYRKCFVDAIAWLHQRP--AEQS- : 472
 M r54KCf D iaW6H

### (B). Molecular Phylogenetic analysis by Maximum Likelihood method (Sc5MaT_1)


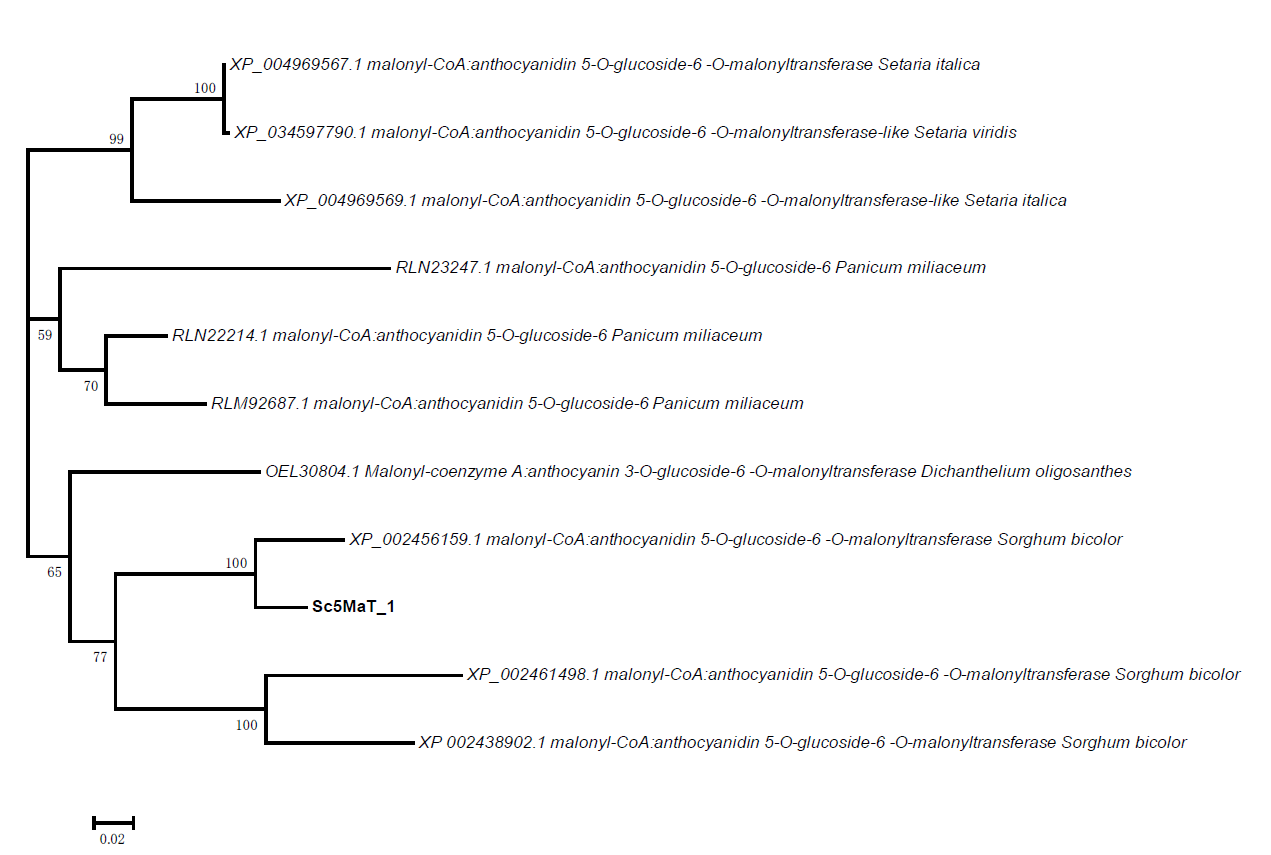


### (C). Multiple sequence alignment of Sc5MaT_2 gene.

* 20 * 40 * 60
XP_0024389 : ------------------------------------------------------------ : -
XP_0049695 : ------------------------------------------------------------ : -
XP_0345977 : ------------------------------------------------------------ : -
XP_0024614 : ------------------------------------------------------------ : -
XP_0024561 : ------------------------------------------------------------ : -
XP_0049695 : ------------------------------------------------------------ : -
RLN22214.1 : ------------------------------------------------------------ : -
OEL30804.1 : ------------------------------------------------------------ : -
RLM92687.1 : ------------------------------------------------------------ : -
RLN23247.1 : ------------------------------------------------------------ : -
Sc5MaT_2 : MDDADEAHSNKPGRQEEANFQQPSKATTDPVTPISTSHNGMAFHLASADLRQSNSHPVPA : 60


 * 80 * 100 * 120
XP_0024389 : ----------MAMAPDQQQPPAGAGAGASSTTGSTSPRLRVHVHDTTLVPPSPSPPETSL : 50
XP_0049695 : ----------MAVAPDQKQRPAGSS--------SASPHLR--VHDTILVAPSPSPPETSL : 40
XP_0345977 : ----------MAVAPDQKQRPAGSS--------SASPHLR--VHDTILVAPSPSPPQTSL : 40
XP_0024614 : ----------MAVTPDQQQPPPPAG--A----SSTPPRLLVHVHETTLVPPSPSPPETSL : 44
XP_0024561 : --------MAMAAAPDQQQLPAGAS--------SSSPRLR--IHDTTLVPPSPSPPETSL : 42
XP_0049695 : ----------MAVAPEQHQQPAG-A--------SSSPRIR--VHDTTLVSPSPSPREASL : 39
RLN22214.1 : --------MAMAVAPDQQQVPAGAS--------SAPPRLR--VHDTTLVPPSPSPPETSL : 42
OEL30804.1 : ----------MAAAPDQEQLASS-----------ASPRFRV-LDDTTLVPPSPSPPETSL : 38
RLM92687.1 : --------MAMAVAPDQQQVPAGAS--------SAPPRLLC-VHDTMLVPPSPSPPETSL : 43
RLN23247.1 : ----------MAAASEQQQLHAGAS--------LSSPRLR--VHVTTLVQPSPSPPEISL : 40
Sc5MaT_2 : GCRELELAAAMAGAPDQQQPPAGAG--------SSSPSSRLRVHDTTLVPASPSPPETSL : 112
 MA apdQ Q pag s P r 6hdT LV pSPSPp2tSL

 * 140 * 160 * 180
XP_0024389 : PLTFFDVFWLQSHPVERLFLYRLA-HDADVEAIISNLRSSLHKALAAFYPLAGRVRLTPG : 109
XP_0049695 : PLTFFDIIWLNSPPVERLLFYRLA-PDADVATIISNLKDSLHKAFRVFYPLAGRLRLTPG : 99
XP_0345977 : PLTFFDIIWLNSPPVERLLFYRLA-PDADVATIISNLKDSLHKAFRVFYPLAGRLRLTPG : 99
XP_0024614 : PLTFLDVFFLHSTPVKRLFLYRLAGPDADVAAIISSLRDSLHQALRAFYPLAGRVRLTPG : 104
XP_0024561 : PLTYFDIFWLHSPPVERLLFYRLA-ADADVATIISNLRDSLHQAVRAFYPLAGRIRLTPG : 101
XP_0049695 : PLTFFDIFWLNSPPVERLFFYRLS-PDADVATIVSNLKKSLQQAVRAFYPLAGRLRLIPG : 98
RLN22214.1 : PLTFFDIIWLHSPPVERLFFYRLA-PDADVAAIVSNLKDSLHQAVRAFYPLAGRLRRTPG : 101
OEL30804.1 : PLTFFDIIWLNSPPVERLFFYRLA-PDADVATIISNLKDSLRQAVRAFYPLAGRLRLSPG : 97
RLM92687.1 : PLTFFDIIWLHSPPVERLFFYRLA-PDADIDNIVSNLKDSFHQAVRAFYPLAGRLRLTPG : 102
RLN23247.1 : PLTFFDSFWLHSPPVERLFFYRLA-PDADVAAIVSNLKDSLHHAVSAFYPLAGHLCLSPG : 99
Sc5MaT_2 : PLTFFDVFWLLSPPVERLFLYRLA-PDADVAAIISNLRNSLHQAVRAFYPLAGRVRLTPG : 171
 PLT5fD 5L SpPVeRL YRLa pDAD6a I6SnL4 Slh A raFYPLAGr6rl PG

 * 200 * 220 * 240
XP_0024389 : TSDRYELHYRPGDAVTFTVAECDDDVDGDAHFDALATDEPREVAKIAALVPTLPRGGRLL : 169
XP_0049695 : TSDRYELYYSPGDAVTFTVAECDDG---DADIDGLAAGDPREVAKIGTLVPPLAEGGGLF : 156
XP_0345977 : TSDRYELYYSPGDAVTFTVAECDDG---DADIDGLAAGDPREVAKIGTLVPPLAEGGGLF : 156
XP_0024614 : TSDRYELHYRPGDAVTFTVAECDD--D--MHFDGLTTDEPREVAKIAALVPPLPDGGKLL : 160
XP_0024561 : TSDRYELHYQPGDAVTFTVAEYDDDDA-DADIDGLTADEPREVAKIATLVPPLPEGGGLF : 160
XP_0049695 : TSDRYELYYRPGDAVTFTVAECAD----DEDIDSLTTDDPREVSKIAPLVPALPEGGGLL : 154
RLN22214.1 : TSDRYELYYRPGDAVTFTVAECDDD---DADIDGLATDDPRELAKIAALVPPLPEGGRLL : 158
OEL30804.1 : TSDRYELYYRPGDGVAFTVAECND----DADIDGLATDEPREVAKIATLVPPLPDGGGLL : 153
RLM92687.1 : ASDRYELYYRPGDAVTFTVAECDDD---DADIDGLATDDPREVAKIAALVPPLPEGGRLL : 159
RLN23247.1 : TSGRYELYYCPGDAVTFTVAECED----NVDIDGLATDDPREVTKIVPLVPPLTEGGGLL : 155
Sc5MaT_2 : TSNRYELYYRPGDAVTFTVAECDDD-DADAHFDALVTDEPREVSKIATLVPPLPGGGRLF : 230
 tS RYEL Y PGDaVtFTVAEc D D L d PRE6 KI LVPpL GG L

 * 260 * 280 * 300
XP_0024389 : AVRATLLPARRGLAIGVTLHHAACDGSGSTHFLHTWAATCRGGGAE-----SPPPP---- : 220
XP_0049695 : ALQATLLSARRGLAIGVTVHHAACDGSNSTHFLHTWAAACS-GTEA-----PPPP----- : 205
XP_0345977 : ALQATLLSARRGLAIGVTVHHAACDGSNSTHFLHTWAAACS-GTEA-----PPPP----- : 205
XP_0024614 : AVQATLLPARRGLAIGVTVHHAACDGSGSTHFLHTWAAACRGGAAEPSLPPPPPPP---- : 216
XP_0024561 : ALQATLLSARRGLAIGVTVHHAACDGSGSTHFLHTWAAACISGAEA-----PPPPPPPPP : 215
XP_0049695 : ALQATLLSASRALAIGVTLHHAACDGSNSTHFLHTWAAACS-GTEA-----PPPP----- : 203
RLN22214.1 : ALQATLLSARRGLAIGVTVHHAACDGSGSTHFLHTWAAACI-GAEA-----PPPPP---- : 208
OEL30804.1 : ALQATLLSARRSLAIGVTVHHAACDGSGSTHFLHTWAAACT-GVEA-----PPPPP---- : 203
RLM92687.1 : ALQATLLSARRGLAIGVTVHHAACDGSASTHFLHTWTAACI-GAET-----PPPPP---- : 209
RLN23247.1 : ALQATLLSARRGLAIGVTAHHVAFDGSGSTHFLHTWAAACK-GADA-----RPLPP---- : 205
Sc5MaT_2 : AVQVTLLPARRGLAIGVTVHHAACDGSGSTHFLHTWAAACRGGAEP-----PPPPP---- : 281
 A6qaTLL ArRgLAIGVT HHaAcDGS STHFLHTWaAaC G pPpP

 * 320 * 340 * 360
XP_0024389 : VIDRTLLADPRRLYDAFVQTAPSSEEYEFVKMSADQLFATFTLSKDDLKRVKDAVADEAA : 280
XP_0049695 : VIDRTLLADPRGLYNVFYQEAPSTDEMEFAKMSADQLFATFTLSKDDLQRIKEVVADEAA : 265
XP_0345977 : VIDRTLLADPRGLYNVFYQEAPSTDEMEFAKMSADQLFATFTLSKDDLQRIKEVVADEAA : 265
XP_0024614 : VIDRTLLPDPRRLYDVFVQAAPSSQELEFIKVSADQLLATFVLSKDDLTRVKDVVADEAT : 276
XP_0024561 : VIDRTLLPDPRGLYDVFFQGAPSTDELEFVKMSADQLIATFVLSKDDLERVKEAVADEAA : 275
XP_0049695 : VIDRTLLADPRGLYNVFYQEAPSTDEMEFAKMSADQLFATFTLSKDDLQRIKEVVADEAA : 263
RLN22214.1 : VIDRTLLTDARGLYDIFCQAAPSTDEMEFVKMSADQLFATFTLSKDDLQRVKDIVADEAM : 268
OEL30804.1 : VIDRTLLTDPRGLYYIFFQGAPRTDEMEFVNMSADQLFATFTLCKEDLRRVKDAVADEAT : 263
RLM92687.1 : VIDRTLLTDPRGLYDIFCQAAPSTDEMEFVKMSADQLFATFTLSKDDLQRVKDIVADEAM : 269
RLN23247.1 : VIDRALLPDPRGLYDIISQAAPSTDDLKFVKMSADQILATFTLSKDDLLRVKDVVADEAA : 265
Sc5MaT_2 : VIDRTLLADPRRLYDIFVQTAPSTEEYEMAEMSADQLFATFALSKDDLKRIKDVVADEAA : 341
 VIDRtLL DpR LY f Q APs3 e ef k6SADQ6 ATF LsKdDL R6K VADEA

 * 380 * 400 * 420
XP_0024389 : RRGVAPPRCSSLVATFGLVWSCY-QRGKEGS---GGGAG-EGSMACMAFPVDHRSRMKPP : 335
XP_0049695 : RRGVAPPRCSSLVATFGFVWSCY-QRAKE-----SCGSG-EGPMTCILFPVNHRSRMKPP : 318
XP_0345977 : RRGVAPPRCSSLVATFGFVWSCY-QRAKE-----SCGSG-EGPMTCILFPVNHRSRMKPP : 318
XP_0024614 : RRGVAPPRCSSLVATFGFVWSCY-QRAKNG----GGGEG-P-MMTCMVIAVDHRSRMKPP : 329
XP_0024561 : RRRVAPPRCSSLVATFGLVWSCY-QRAKDEESI-SGGAG-AGPMACLLFAVDHRSRVKPP : 332
XP_0049695 : RRGVAPPRCSSLVATFGFVWSCY-QRAKE-----SCGSG-EGPMTCILFPVNHRSRMKPP : 316
RLN22214.1 : KRGVAPPRCSSLVATLGFVWSCYLQRAKE-----SCGSDDEGSMTYLLFPVDHRSRMKPP : 323
OEL30804.1 : RRGVAPPRCSSLVAAFGFAWSCY-QRAKE-----NCSAG-ESPMTCMIFPVDHRTRMKPP : 316
RLM92687.1 : RRGVAPPRCSSLVATFGFVWSCYLQRAKE-----SCGSD-EGSMSCLLFPVDHRSRMKPP : 323
RLN23247.1 : KRGVAPPRCTSLVATFGFVWWCY-QRAKE-----SGGAD-EDPTAYMVFPVDHRSRMKPR : 318
Sc5MaT_2 : RRGVAPPRCSSLVATFGFVWSCY-QRAKDKESISGAGAG-P--MPCLLFAVDHRSRVKPP : 397
 4RgVAPPRC3SLVAtfGfvWsCY QRaK g m c6 f V1HR3R6KPp

 * 440 * 460 * 480
XP_0024389 : LPEKYLGNCVGPAFALAPTGELAAAGAGGLFSACAAVASAIDEAVRDIGTSSMDAWMDRI : 395
XP_0049695 : LPERYLGNCVGPAFGMAPKSELAVAGVGGLFTACAAVASAIDEAVRDIGTSSMDAWLDRI : 378
XP_0345977 : LPERYLGNCVGPAFGMAPKSELAVAGVGGLFTACAAVASAIDEAVRDIGTSSMDAWLDRI : 378
XP_0024614 : LPDKYLGNCVGPAFALAPTGELAVAGAGGLFSACAAVAASIDEAVRDIGTSSMEAWMDRI : 389
XP_0024561 : LPDKYLGNCVGPAFALAPQGELAVAGAAGIFSACAAVASSIDEAVRDIETSAMDVWLDRV : 392
XP_0049695 : LPERYLGNCVGPAFGMAPKSELAVAGVGGLFTACAAVASAIDEAVRDIGTSSMDAWSDRI : 376
RLN22214.1 : LPDKYLGNCVGPAVGMAPKGELAAAGAGGLFSACAAVVSAIDEAVRGIGTSSMDAWVDRI : 383
OEL30804.1 : LPDKYLGNCVGPAFAMAPKGELAAAGAGGLFSACSAVASAIDEAVRDIGTSNMDVWDDRV : 376
RLM92687.1 : LPDKYLGNCVVPAFGMAPKGELAAAGAGGLFSACTAVASAIDEAVRDIGTPSMDAWLDRI : 383
RLN23247.1 : LPDEYLGNCVGPAFVVAHKGELAAAGAGGLFTACAAVASAIDEAVRGIGTSNMDAWVRRI : 378
Sc5MaT_2 : LPDKYLGNCVGPAFALAPTGELAAAGAGGLFSACAAVASSIDEAVRDIGTSSMDAWMDRI : 457
 LP YLGNCVgPAf 6Ap ELA AG gG6F3ACaAVas IDEAVRdIgTs MdaW dR6

 * 500 * 520 * 540
XP_0024389 : REVLPMG-LLTVAGSPRFRVYDLDFGFGRPAKVDIVSVARTGAVAVAESRS--GDGGIEV : 452
XP_0049695 : KEASANG-ILSVAGSPRFRVYELDFGFGRPLKVDIVSVARTGAVAVAESRS--CIGGMEV : 435
XP_0345977 : KEASANG-ILSVAGSPRFRVYELDFGFGRPLKVDIVSVARTGAVAVAESRS--CIGGMEV : 435
XP_0024614 : KEVLLMD-LLTVSGSPRFRVYDLDFGFGRPAKVEVVPVTRTGVVAVAESRV--GDGGIEV : 446
XP_0024561 : KEAGTKGTLLSVAGSPRFRVYDLDFGFGRPAKVDIVSVARTGALAMAESRSRSSTGGMEV : 452
XP_0049695 : KEASANG-ILSVAGSPRFRVYELDFGFGRPLKVDIVSVARTGAMAVAESRS--SAGGMEI : 433
RLN22214.1 : MEAAAAG-LLSVAGSPRFRVYELDFGFGRPAKVDIVSVARTGAVAVAESRS--STGGMEV : 440
OEL30804.1 : REAMAMG-ILTVAGSPRFRVYELDFGFGRPEKVDIVSVARTGAVAVAESRG--SASGMEV : 433
RLM92687.1 : REASAKG-VLSVAGSPRFRVYELDFGFGRPAKVYIVSVARTGAVAVAESRR--SIGGMEV : 440
RLN23247.1 : MEAAAAG-VLSVAGSPRFRVYELDFGFGRLAKVDIVSVARTGEVAVAESRS--SAGGMEV : 435
Sc5MaT_2 : REVIPLG-VLTVAGSPRFRVYDLDFGFGQPAKVDVVSVARTGAVAVAESRG--GDGGIEV : 514
 E g 6L3VaGSPRFRVY LDFGFGrp KVd6VsVaRTGa6A6AESR gG6E6

 * 560 *
XP_0024389 : GVSLQPAAMERYRKCFADATLWLHQKT------- : 479
XP_0049695 : GVSLQPAGMDRYRKCFTDGIAWLHQRS------- : 462
XP_0345977 : GVSLQPAGMDRYRKCFTDGIAWLHQRS------- : 462
XP_0024614 : GVSLQPAAMGSYKKCFADAIAWLHQRRHESDDSF : 480
XP_0024561 : GVSLQPAGMERFRKCFVDAIAWLHLHQSRVEQS- : 485
XP_0049695 : GVSLQPADMDRFRKCMADAIAWLHNHNHQS---- : 463
RLN22214.1 : GISLQPAGMDRYRKCFADCIAWLHQRS------- : 467
OEL30804.1 : GVSLQPAGMERYRKCFADAIAWLHQRT------- : 460
RLM92687.1 : GVSLQPAGMERYRKCFADGIAWLHQPS------- : 467
RLN23247.1 : GVSLQPAGMDRYRKCFADGIAWIHNHQS------ : 463
Sc5MaT_2 : GVSLRPAAMERYRKCFADAVAWLHQKK------- : 541
 G6SLqPA M r54KCf D aW6H

### (D). Molecular Phylogenetic analysis by Maximum Likelihood method (Sc5MaT_2)


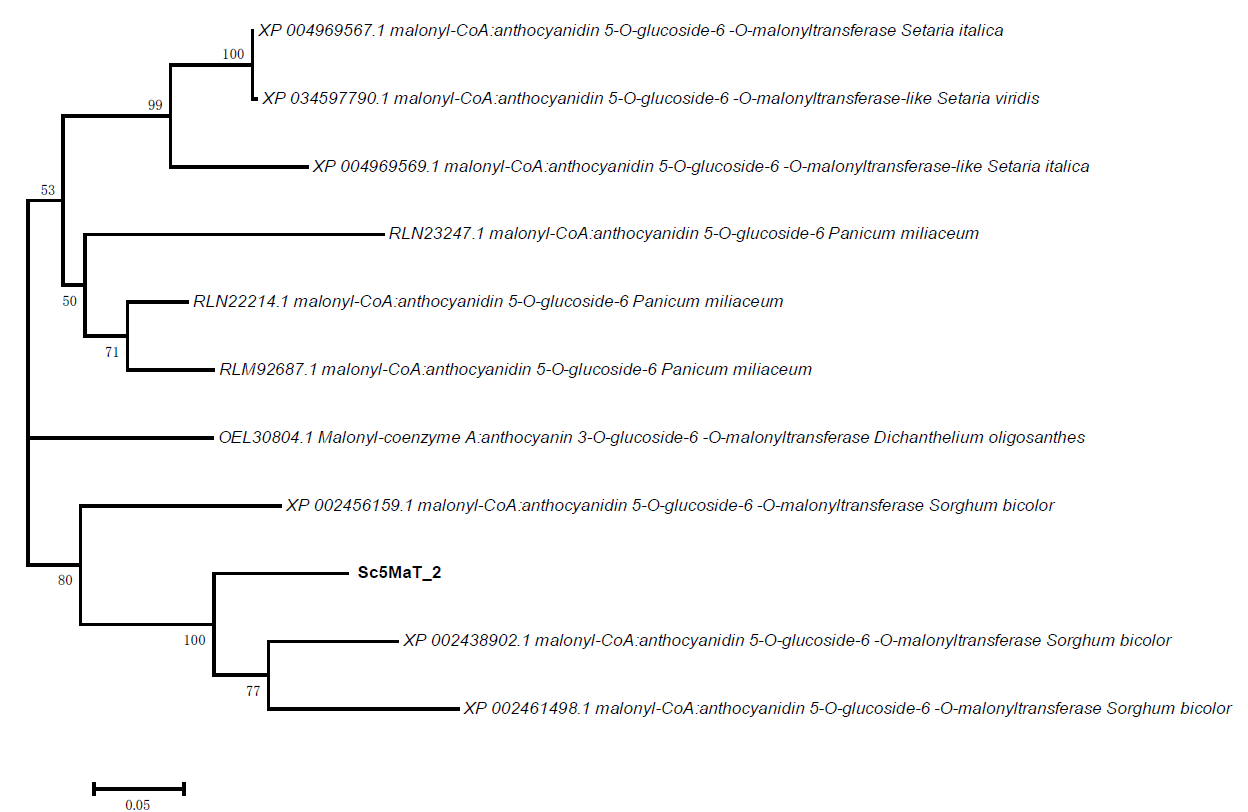


### (E). Multiple sequence alignment of Sc5MaT_3 gene.

* 20 * 40 * 60
XP_002459867 : ------------------------------------------------------------ : -
XP_034581770 : MNSLYNYISLHVISGSGCGAWAVTLPFAKLASLKQPSSVLSKSGRSGTCPTCQCPPLLLI : 60
XP_004956266 : ------------------------------------------------------------ : -
XP_025802054 : ------------------------------------------------------------ : -
RLN34265.1 : ---------------------------------MQQLGLLCP-----TCQSSLCPHLLTC : 22
OEL24084.1 : ------------------------------------------------------------ : -
RLM84313.1 : ------------------------------------------------------------ : -
PWZ40298.1 : ------------------------------------------------------------ : -
Sc5MaT_3 : -----------------------------------------------LGRSSNVSSACDS : 13


 * 80 * 100 * 120
XP_002459867 : ------------------------------------------MAPPPSPLP-RVRVLERI : 17
XP_034581770 : SLPSWLPRLLSSILNRSRAEQSGAEEATQQLQTRAEQRRRADQMAPPSSPP-DVRLVDRI : 119
XP_004956266 : -------------------------------------------MAPPSSPP-DVRLVDRI : 16
XP_025802054 : -------------------------------------------MAPPSSSP-GVRVVDRI : 16
RLN34265.1 : LLPSCLPPFLN-----PQAEQSRVEQRRPLNNCTLGQSSGADQMAPPSSSP-GVHVVDRF : 76
OEL24084.1 : -------------------------------------------MAAPSSQS-GVHVLDRI : 16
RLM84313.1 : -------------------------------------------MAPPSSSP-GVRMVDRI : 16
PWZ40298.1 : -------------------------------------------MAPPSPQPPLVRVLDRI : 17
Sc5MaT_3 : PATADSPSLFT--LSQLQKRHGGGRSRAERIAEDREQSTRSDQMAPPSPQP-RVRVLERI : 70
 mapPS p Vr66dRi

 * 140 * 160 * 180
XP_002459867 : RVEPQP-SDSEQEESALPLTFFDVAWLFTGPVERLFFFRHPEPASTLPLLRSSLSLALRR : 76
XP_034581770 : RVSPAP-SDSAQE-HALPLTFFDVAWLFTGPVERLFFFRHPDPSSALPLLRSSLPVALRR : 177
XP_004956266 : RVSPAP-SDSAQE-HALPLTFFDVAWLFTGPVERLFFFRHPDPSSALPLLRSSLPVALRR : 74
XP_025802054 : RVSPPPPSNSARE-PALPLTFFDVAWLFTGPVERLFFFRHPDPASVLPLLRSSLSVTLRR : 75
RLN34265.1 : RVSPPPPSDSARE-PALPLTFFDVAWLFTGPVERLFFFRHPDPASALPQLRASLSVALRR : 135
OEL24084.1 : RLSPPP-SDSAQE-PALPLTFFDAAWLFTGPVERLFFFRHPDPASTLPLLRSSLSVALRR : 74
RLM84313.1 : RVSPPPPSNSARE-PALPLTFFDVAWLFTGPVERLFFFRHPDPASVLPLLRSSLSVVLRC : 75
PWZ40298.1 : RVSPPP-ADSEQE-PALPLTFFDVAWLFTGPVERLFFFRHPEPASTLPLLRSSLSLALRR : 75
Sc5MaT_3 : RVEPQP-SGSEQEEPALPLTFFDVAWLFTGPVERLFFFRHPEPASTLPLLRSSLSLALHR : 129
 R6sP P s S E ALPLTFFDvAWLFTGPVERLFFFRHP PaS LPlLRsSLs6aLrr

 * 200 * 220 * 240
XP_002459867 : FYPLAGTIRPHAPFLCSYTRGTDALTMVVAESDSPEDFDRLVARSPRDLALIRPLVPQLP : 136
XP_034581770 : FYPLAGTIRPHAPFLCSYARGADALTLVVAEFDSPDDFDHLVARAPRDLSRIRALVPQLP : 237
XP_004956266 : FYPLAGTIRPHAPFLCSYARGADALTLVVAEFDSPDDFDHLVARAPRDLSRIRALVPQLP : 134
XP_025802054 : FYPLAGTIRPHPPFLCSYTPGADALTLVVAESDSPDDFDRLVARSPRDLAGIRPLVPQLP : 135
RLN34265.1 : FYPLAGTIRPHPPFLCSYTPGADALTLVIAESDSPDDFDRLVARSPRDLDGIHPLVPQLP : 195
OEL24084.1 : FYPLAGTIRPQAPILCSYTRGADALTLVVAESDSPDDFDRLVARSPRDLTGIRPLVPQLP : 134
RLM84313.1 : FYPLAGTIRPHPPFLCSYTPGADALTLVVAESDSPDDFDRLVARSPRDLAGIRPLVPQLP : 135
PWZ40298.1 : FYPLAGTIRPHAPFLCSYTHGTDALTMVVAESNSPDDFDSLVARSPRDLDGIRPLVPQLP : 135
Sc5MaT_3 : FYPLAGTIRPHAPFLCSYTRGTDALTMVVAESDSPDDFDRLVARSPRDLALIRPLVPQLP : 189
 FYPLAGTIRPh PfLCSYt G DALT6V6AEs1SPdDFD LVARsPRDL IrpLVPQLP

 * 260 * 280 * 300
XP_002459867 : PPGNDGAFALAAVQVTVFPGRGLCLGVSVHHAACDDASTMHFVRTWAAACRLGLENDEGS : 196
XP_034581770 : PPGDDGAFALAALQVTVFPGRGLCLGVSVHHAACDDASTMHFVRTWAAACRLGLESGDGS : 297
XP_004956266 : PPGDDGAFALAALQVTVFPGRGLCLGVSVHHAACDDASTMHFVRTWAAACRLGLESGDGS : 194
XP_025802054 : PPGEDGAFSLAAVQATVFPGRGICLGVSVHHAACDDTSTMHFVRTWAAACRLGLESDDGS : 195
RLN34265.1 : PPGDDGAFALAAVQATVFPGHGLCLGVSVHHAACDDASTMHFVRTWAAACRLGLESDDGS : 255
OEL24084.1 : PPGDDGAFALAAVQVTVFPGRGLCLGVSVHHAACDDASTMHFVRTWATAGRLGLESDDGS : 194
RLM84313.1 : PPREDGAFALAAVQATVFPGRGICLGVSVHHAACDDASTMHFVRTWAAACRLGLESDDGS : 195
PWZ40298.1 : RPGEDGAFALAAVQATVFPGRGLCLGVSVHHAACDDASTMHFVRTWAATCRLGLENDDGS : 195
Sc5MaT_3 : PPGDDGAFALAAVQVTVFPGRGLCLGVSVHHAACDDASTMHFVRTWAAACRLGLENDEGS : 249
 pPg DGAFaLAA6Q TVFPGrG6CLGVSVHHAACDDaSTMHFVRTWAaacRLGLE ddGS

 * 320 * 340 * 360
XP_002459867 : EDAVLPPPPVLDRSVVADPDDLRGKTLAGMARLAPPPPPT-PPPPQQEEKTPMVMASFLL : 255
XP_034581770 : GDATLPPPPVLDRSLVADPDDLRGKTLAGMARLAPPPPPP--PQQQQEEQTPMVMASFLL : 355
XP_004956266 : GDATLPPPPVLDRSLVADPDDLRGKTLAGMARLAPPPPPP--PQQQQEEQTPMVMASFLL : 252
XP_025802054 : EDAALPPPPVLYRSLVADPDDLRGKTLAGMARLAPPPPPQ---EEEEEEKAPMVMASFLL : 252
RLN34265.1 : EDAALPPRPVLDRSLVADPDDLRGKTLAGMTRLAPPPPPPSPPQHQEEEKAPMVMASFLL : 315
OEL24084.1 : EDATLPPPPVMDRSLVADPDDLRGKTLAGMARLAPPPPPP---PQQQEEKTLMVMASFLL : 251
RLM84313.1 : EDAALPPPPVLDRSLVADPDDLRGKTLAGMARLAPPPPPQ---EEEEEEKAPMVMASFLL : 252
PWZ40298.1 : EDAVLPPRPVLDRSVVADPDDLRGKTLAGMARLAPP--PS-PPPQQQEEKTPMVMASFLL : 252
Sc5MaT_3 : EDTVLPPPPVLDRSVVADPDDLRGKTLAGMARLAPPPPPT-PPPPQQEEKTPMVMASFLL : 308
 eDa LPPpPV6dRS6VADPDDLRGKTLAGMaRLAPPppP 22EEk pMVMASFLL

 * 380 * 400 * 420
XP_002459867 : PREQIDRIKEGAAAKA----SSFVAASALAWVCLLKCGSAGVAGAQRSHMLFSAECRSRL : 311
XP_034581770 : PRHQIDRIKEGAAARSDAKASSFVAASALAWVCLLKSGSAGVAGAERSHMLFSAECRSRL : 415
XP_004956266 : PRHQIDRIKEGAAARSDAKASSFVAASALAWVCLLKSGSAGVAGAERSHMLFSAECRSRL : 312
XP_025802054 : PRHQIDRIKEGAAAKSDAKPSSFVAASALAWVCLLKSGSSGVAGAGRSHMLFSAECRTRL : 312
RLN34265.1 : PRHQIDRIKEGAAPKADAKPSSFVAASALAWVCLLKSGSAGVAGAERSHMLFSAECRARL : 375
OEL24084.1 : LRHQIDRIKEGAAAKADAKASSFVAASALAWVCLLKSGSTGVAGAERSHMLFSAECRSRL : 311
RLM84313.1 : PRHQIDRIKEGAAAKSDAKPSSFVAASALAWVCLLKSGSSGVAGAERSHMLFSADCRTRL : 312
PWZ40298.1 : GREQIDRIKEGAAAKA----SSFVAASALAWVCLLKCGSVGVAGARRSHMLFSAECRSRL : 308
Sc5MaT_3 : PREQIDRIKEGAAAKADAKASSFVAASALAWVCLLKSGSAGVAGAQRSHMLFSAECRSRL : 368
 pR QIDRIKEGAAa4 dak SSFVAASALAWVCLLKsGS GVAGA RSHMLFSAeCR RL

 * 440 * 460 * 480
XP_002459867 : TPPLQAEYFGNCLRPCFVEAATADLLSGETADGVAAAAAAIGSAIREMEQGVLDGAEGWL : 371
XP_034581770 : APPLPAEYFGNCLRPCFVEAATADLLSGDTADGVAAAASAIGSAIREMEQGVLEGAEGWL : 475
XP_004956266 : APPLPAEYFGNCLRPCFVEAATADLLSGDTADGVAAAASAIGSAIREMEQGVLEGAEGWL : 372
XP_025802054 : TPPLPAEYFGNCLRPCFVEAATADLLSGETADGVAAAASAIRSAIREMEQGVLEGAEGWL : 372
RLN34265.1 : TPPLSAEYFGNCLRPCFVEAATADLLSGETADGIAAAASAIGSAIREMEQGVLEGAEGWL : 435
OEL24084.1 : TPPLPAEYFGNCLRPCFVEATTADLLSGNTADGVAAAASAIGSAIRDMEQGVLEGAEGWL : 371
RLM84313.1 : TPPPPAEYFGNCLRPCFVEAATADLLSGETADGVAAAASAIGSAIREMEQGVLEGAEGWL : 372
PWZ40298.1 : TPPLPAEYFGNCLRPCFVEAATADLLSGETEDGVAAAASAIGSAIQEMEQGVLEGAEGWL : 368
Sc5MaT_3 : TPPLPAEYFGNCLRPCFVEAATVDLLSGETAAGVAAAAAAIGSAIREMEQGVLEGAEGWL : 428
 tPPlpAEYFGNCLRPCFVEAaTaDLLSG TadG6AAAAsAIgSAIreMEQGVLeGAEGWL

 * 500 * 520 * 540
XP_002459867 : GSVLSVLPERPMSAGGSPRHGVYETADFGWGRPARVEMVSVEKTPGTVALAESPEGDGGI : 431
XP_034581770 : GRVLSVLPERPMSVGGSPRHGVYETTDFGWGRPARVEMVSVEKTPGTVALADSPEGDGGI : 535
XP_004956266 : GRVLSVLPERPMSVGGSPRHGVYETTDFGWGRPARVEMVSVEKTPGTVALADSPEGDGGI : 432
XP_025802054 : GRVLSVLPERPMSVGGSPRHGVYETADFGWGRPARVEMVSVEKTPGTVALADSPEGDGGI : 432
RLN34265.1 : GRVLSVLPQRPMSVGGSPRHGVYETADFGWGRPARVEMVSVEKTPGTVALADCPEGDGGI : 495
OEL24084.1 : GRVLSVLPERPMSVGGSPRHGVYEATDFGWGRPARVEMVSVEKTPGTVALADSPEGDGGI : 431
RLM84313.1 : GRVLSVLPERPMSVGGSPRHGVYETADFGWGRPARVEMVSVEKTPGTVALADSPEGDGGI : 432
PWZ40298.1 : GRVLSVLPERPMSVGGSPRHGVYETTDFGWGRPARVEMVSVEKTPGTVALADSPEGDGGI : 428
Sc5MaT_3 : GRVLSVLPERPMSVGGSPRHGVYETTDFGWGWPARVEMVSVEKTPGTVALAESPEGDGGI : 488
 GrVLSVLP2RPMSvGGSPRHGVYEt DFGWGrPARVEMVSVEKTPGTVALAdsPEGDGGI

 * 560
XP_002459867 : ELGVVLPPDAMDAFASCFAHALG-ATV : 457
XP_034581770 : ELGVVLPPDAMDAFASCFADALAGATI : 562
XP_004956266 : ELGVVLPPDAMDAFASCFADALGGATI : 459
XP_025802054 : ELGVVLPTDAMDAFASCFADALGGATI : 459
RLN34265.1 : ELGVVLPTDAIDAFASCFADALGGTTV : 522
OEL24084.1 : ELGVVLPPDAMDAFASCFANALG-ATI : 457
RLM84313.1 : ELGVVLPTDAMDAFASCFADALGGATI : 459
PWZ40298.1 : ELGVVLPPDAMDAFASCFAHAVG-ANV : 454
Sc5MaT_3 : ELGVVLPPDAMDAFASCFAHAVG-ATV : 514
 ELGVVLP DA6DAFASCFA A6g at6

### (F). Molecular Phylogenetic analysis by Maximum Likelihood method (Sc5MaT_3)


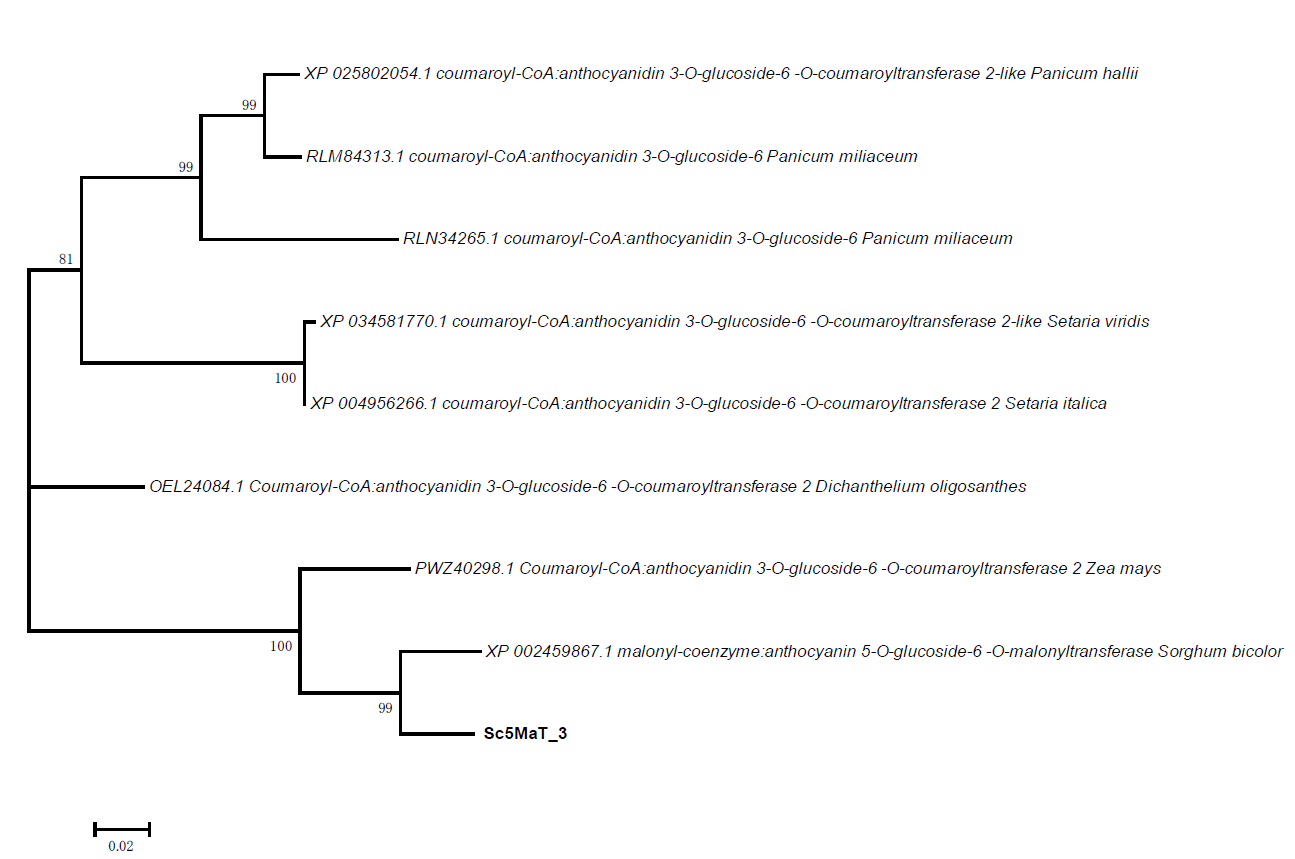


## Fig S17. Sequence alignment of Malonyl-coenzyme A: anthocyanin 3-O-glucoside-6''-O-malonyltransferase (3MaT) proteins from sugarcane and various other plants, and phylogenetic relationships of Malonyl-coenzyme A: anthocyanin 3-O-glucoside-6''-O-malonyltransferase.

### (A). Multiple sequence alignment of Sc3MaT_1 gene.

* 20 * 40 * 60
XP_002454794.2 : MAAATKVLDRLKVGASPPAPGGALPLTFFDVPWLFTGPVERVFFYHYAHTAEHFAAHLLP : 60
OEL36677.1 : --MATKVLDRVKVAASPPAPGGVLPLTFFDVPWLFTGPVERVFFYPYPHTAEHFTAHLLP : 58
XP_034599996.1 : --MATKILDRLTVAASPPAPGGVLPLTFFDVPWLFTGPVERVFFYPYPHTAEHFTAHLLP : 58
XP_004954417.1 : --MATKILDRLTVAASPPAPGGVLPLTFFDVPWLFTGPVERVFFYPYPHTAEHFTAHLLP : 58
RLN07180.1 : --MATKVLDRLTVSASPPAPGGVLPLSFFDVPWLFTGPVERVFFYPYPHTAEQFTARLLP : 58
RLM79758.1 : --MATKVLDRLTVAALPPAPGGVLPLTFFDVPWLFTGPVERVFFYPYPHTAEQFTARLLP : 58
Sc3MaT_1 ：MAATTKVLDRLKVGASPPAPGGVLPLTFFDVPWLFTGPVERVFFYPYAHTAEHFAAHLLP : 60
 aTK6LDR6 V AsPPAPGGvLPL3FFDVPWLFTGPVERVFFYpY HTAE F A LLP

 * 80 * 100 * 120
XP_002454794.2 : SLVSSLSATLHAFYPLLGRVRPCPDGSSGGYEFFCSAGGEGGEAVELTVAESSDDFDELA : 120
OEL36677.1 : PLVSSLSAALHGFYPLVGRVCPCPDGG-GGYEFCS-AGEDAGEGVELTVAESSDDFDELS : 116
XP_034599996.1 : HLVSSLSAALHRFYPLVGRVRPCPDGG-GGYEFCS-AGGDAGEGVELTVAESSDDFDELS : 116
XP_004954417.1 : HLVSSLSAALHRFYPLVGRVRPCPDGG-GGYEFCS-AGGDAGEGVELTFAESSDDFDELS : 116
RLN07180.1 : PLVSSLSAALHWFYPLLGRVRPCPDGG-GGYEFFS-AGGDAGEGVELTVAESSDDFDELS : 116
RLM79758.1 : PLMSSLSAALHWFYPLLGRVRPCPDGG-GGYEFFS-AGGDAGEGVELTVAESSDDFDEVS : 116
Sc3MaT_1 ：SLVSSLSATLHAFYPMLGRVRPSPDGG-GGYEFFCSAGGEDGEAIELTVAESSDDFDELS : 119
 L6SSLSA LH FYP66GRVrPcPDGg GGYEF AGg GE 6ELTvAESSDDFDE6s

 * 140 * 160 * 180
XP_002454794.2 : GGGPRDVARLYALVPRLPPPEVDGSFALAAAQVTVFPACGVAVGVSIHHVACDDSSYMHF : 180
OEL36677.1 : GGGPRDVSRLYALVPQLPR-TEDGTFSLAAAQVTVFAGRGIAVGVSIHHVACDDSSYMHF : 175
XP_034599996.1 : GCGPRDVARLYALVPQLPR-AEDGTFALAAAQVTVFAGRGIAVGVSIHHVACDDSSYMHF : 175
XP_004954417.1 : GCGPRDVARLYALVPQLPR-AEDGTFALAAAQVTVFAGRGIAVGVSIHHVACDDSSYMHF : 175
RLN07180.1 : GGSPRDVARLYALVPQLPR-TEDGTFALAATQVTVFAGRGIAVGISIHHVACDDSSYMHF : 175
RLM79758.1 : GGAPRDVARLYALVPQLPR-TEDGTFALAAAQVTVFAGRGIAVGVSIHHVACDDSSYMHF : 175
Sc3MaT_1 ：SDGSRDVARLYALVPRLPPPEADGSFALAAAQVTVFPARGVAVGVSIHHVACDDSSYMHF : 179
 g pRDVaRLYALVP LP DG3FaLAAaQVTVF rG6AVG6SIHHVACDDSSYMHF

 * 200 * 220 * 240
XP_002454794.2 : VKTWAARCRVAVTGADADADAVPVPFFDRGVVADPEGLAARTLDEMRQLAANGPPPPPPA : 240
OEL36677.1 : VKTWAGQCRVASGEESTEGALPPPPFLDRGVVADPEGLAARTLDEMRQLAANGPPPPPPP : 235
XP_034599996.1 : VKTWAGQCRVAAGEEHAEGALPPPPFLDRGVVADPEGLAARTLDEMRQLAANGPPPPPPP : 235
XP_004954417.1 : VKTWAGQCRVAAGEEHAEGALPPPPFLDRGVVADPEGLAARTLDEMRQLAANGPPPPPPP : 235
RLN07180.1 : VKTWAGQCRVAAGEESAEGALPPPPFLDRGVVADPEGLAARTLDEMRQLAANAPPPPPPP : 235
RLM79758.1 : VKTWAGQCRVAAGEEFAEGALPPPPFLDRGVVADPEGLAARTLDEMRQLAANAPPPPPPP : 235
Sc3MaT_1 ：VKTWAARCREAVGGADAVPVPPPPPFLDRGVVADPEGLAARTLDEMRQLAANGPPPPPP- : 238
 VKTWA CRvA g a pPpPFlDRGVVADPEGLAARTLDEMRQLAAN PPPPPP

 * 260 * 280 * 300
XP_002454794.2 : APAGPPPKLVIASFALTRDRIDGLKQRVTSKFADGGGTERVHCSAFTVACALAWACLART : 300
OEL36677.1 : --TGPPPKLVIASFALTRDRIDKLKQRVVAEGANGG--GRVHCSAFTVACAFAWACLARV : 291
XP_034599996.1 : PPTGPPPKLVIASFALTRDRIDRLKQRVVADGG-----ERVHCSAFTVACAFAWACLARV : 290
XP_004954417.1 : PPTGPPPKLVIASFALTRDRIDRLKQRVVADGG-----ERVHCSAFTVACAFAWACLARV : 290
RLN07180.1 : PPAGPPPKLVIASFALTRDRIDKLKQRVVAD----------HCSAFTVACAFAWACLVRV : 285
RLM79758.1 : R-TGPPPKLVIASFALTRDRIDKLKQRVVAEGG-----GRFHCSAFTVACAFAWACLARV : 289
Sc3MaT_1 ：APAGPPPKLVIASFALTRDRIDALKRRVAAKVADGGGTGRVHCSAFTVACALAWACLARV : 298
 GPPPKLVIASFALTRDRID LKqRV a r HCSAFTVACA AWACLaRv

 * 320 * 340 * 360
XP_002454794.2 : GSG--GDERPRAHLLFSVECRRRLAPPIPQEYLGNCLRPCFVEVGATELLGGDGVAAAAA : 358
OEL36677.1 : DGGGCADTKRRAHLLFSVECRRRLAPPIPQEYLGNCLRPCFVEVGLGELLGADGVAAAAS : 351
XP_034599996.1 : DGG-RADAERRAHLLFSVECRRRLAPPIPQEYLGNCLRPCFVEVGLGELLGGDGVVAAAS : 349
XP_004954417.1 : DGG-RADAERRAHLLFSVECRRRLAPPIPQEYLGNCLRPCFVEVGLGELLGGDGVVAAAS : 349
RLN07180.1 : DGG-CADTERRAHLLFSVECRRRLAPPIPQEYLGNCLRPCFVEVGLGELLGADGVVAAAA : 344
RLM79758.1 : DGG-CADAERRAHLLFSVECRRRLAPPIPQEYLGNCLRPCFVEVGLGELLGGDGVVAAAA : 348
Sc3MaT_1 ：GGG--GTERPRAHLLFSVECRRRLAPPIPQEYLGNCLRPCFVEVGAAELLGGDGVAAAAA : 356
 gG d RAHLLFSVECRRRLAPPIPQEYLGNCLRPCFVEVG ELLG DGV AAA

 * 380 * 400 * 420
XP_002454794.2 : AIGAAVAGLDGGVLDGAGGWFHKILSLVPERPMSVGGSPRYGVYETDFGLGRPAKVELVS : 418
OEL36677.1 : AIGASIRALDEGVLEGAGGWFHKILSLVPERPMSVGGSPRYGVYETDFGLGRPSKVELVS : 411
XP_034599996.1 : AIGASVRALDDGVLAGAGGWFHKILSLVPERPMSVGGSPRYGVYETDFGLGRPIKVELVS : 409
XP_004954417.1 : AIGASVRALDDGVLAGAGGWFHKILSLVPERPMSVGGSPRYGVYETDFGLGRPIKVELVS : 409
RLN07180.1 : AIGASIRALDDGVLDGADGWFHKILSLVPQRPMSVGGSPRYGVYETDFGLGRPEKVELVS : 404
RLM79758.1 : AIGASIRALDDGVLAGAGGWFHKILSLVPERPMSVGGSPRYGVYETDLGLGLPKKVELVS : 408
Sc3MaT_1 ：AIGAAVAGLDGGVLDGAGGWFHKILSLVPERPMSVGGSPRYGVYETDFGLGRPGKVELVS : 416
 AIGA 6 LD GVL GAgGWFHKILSLVP2RPMSVGGSPRYGVYETDfGLGrP KVELVS

 * 440 * 460 *
XP_002454794.2 : IDKTPGTVSLAEGRDADGAQAAGVEIGVVLPEAEMARFSSCFADALEELCD : 469
OEL36677.1 : IDKTPGTVXLAEGRDAQ----AGIEIGVVLPEAEMARFXSCFSDGLEQL-- : 456
XP_034599996.1 : IDKTPGTVSLAEGREKL----AGIEIGVVLPEADMARFSSCFADGLEQLL- : 455
XP_004954417.1 : IDKTPGTVSLAEGRDKL----AGIEIGVVLPEADMARFSSCFADGLEQLL- : 455
RLN07180.1 : IDKTPGTVSLAEGRDAQ----AGIEIGVVLPEAEMARFSSCFSDGLEQL-- : 449
RLM79758.1 : IDKTPGTVSLAEGRDAQ----AGIETGVVLPEAEMARFSSCFSDGLGQL-- : 453
Sc3MaT_1 : IDKTPGTVSLAEGRDAD-AHAAGVEIGVVLPEADMARFSSCFADALEELCD : 466
 IDKTPGTVsLAEGRd AG6EiGVVLPEA MARFsSCF D Le2L

### (B). Molecular Phylogenetic analysis by Maximum Likelihood method (Sc3MaT_1)


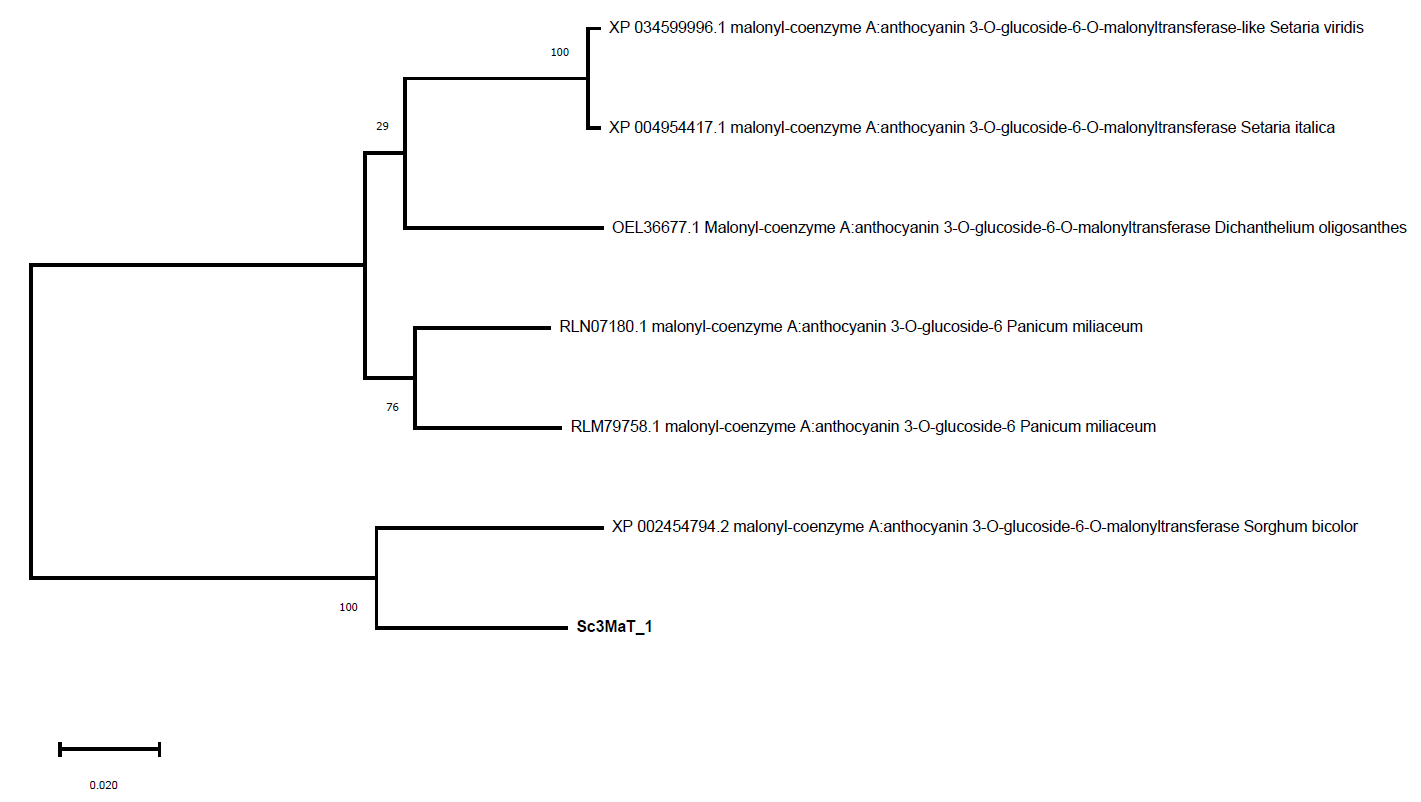

Supplement: Supplementary file 1 — Additional file 1. [file 12870_2021_2986_MOESM1_ESM.docx]
